# Lentiviral vectors for precise expression to treat X-linked lymphoproliferative disease

**DOI:** 10.1016/j.omtm.2024.101323

**Published:** 2024-08-20

**Authors:** Paul G. Ayoub, Julia Gensheimer, Lindsay Lathrop, Colin Juett, Jason Quintos, Kevin Tam, Jack Reid, Feiyang Ma, Curtis Tam, Grace E. McAuley, Devin Brown, Xiaomeng Wu, Ruixue Zhang, Kathryn Bradford, Roger P. Hollis, Gay M. Crooks, Donald B. Kohn

**Affiliations:** 1 Department of Molecular & Medical Pharmacology, University of California, Los Angeles, Los Angeles, CA 90095, USA; 2 Department of Microbiology, Immunology & Molecular Genetics, University of California, Los Angeles, Los Angeles, CA 90095, USA; 3 David Geffen School of Medicine, University of California, Los Angeles, Los Angeles, CA 90095, USA; 4 Division of Pediatric Hematology-Oncology, University of California, Los Angeles, Los Angeles, CA 90095, USA; 5 Eli & Edythe Broad Center for Regenerative Medicine & Stem Cell Research, University of California, Los Angeles, Los Angeles, CA 90095, USA; 6 Jonsson Comprehensive Cancer Center, University of California, Los Angeles, Los Angeles, CA 90095, USA; 7 Department of Pathology & Laboratory Medicine, University of California, Los Angeles, Los Angeles, CA 90095, USA

**Keywords:** HSC, gene therapy, lentiviral vector, XLP, SAP, SH2D1A, regulated, enhancer, stem cell

## Abstract

X-linked lymphoproliferative disease (XLP1) results from *SH2D1A* gene mutations affecting the SLAM-associated protein (SAP). A regulated lentiviral vector (LV), XLP-SMART LV, designed to express SAP at therapeutic levels in T, NK, and NKT cells, is crucial for effective gene therapy. We experimentally identified 34 genomic regulatory elements of the *SH2D1A* gene and designed XLP-SMART LVs to emulate the lineage and stage-specific control of SAP. We screened them for their on-target enhancer activity in T, NK, and NKT cells and their off-target enhancer activity in B cell and myeloid populations. In combination, three enhancer elements increased SAP promoter expression up to 4-fold in on-target populations *in vitro*. NSG-Tg(Hu-IL15) xenograft studies with XLP-SMART LVs demonstrated up to 7-fold greater expression in on-target cells over a control EFS-LV, with no off-target expression. The XLP-SMART LVs exhibited stage-specific T and NK cell expression in peripheral blood, bone marrow, spleen, and thymic tissues (mimicking expression patterns of SAP). Transduction of XLP1 patient CD8+ T cells or BM CD34+ cells with XLP-SMART LVs restored restimulation-induced cell death and NK cytotoxicity to wild-type levels, respectively. These data demonstrate that it is feasible to create a lineage and stage-specific LV to restore the XLP1 phenotype by gene therapy.

## Graphical abstract

**Figure:**
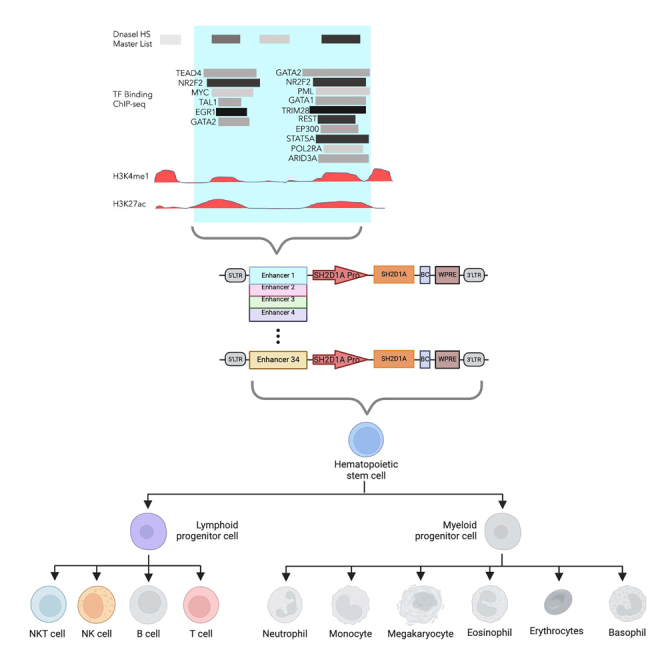

## Teaser

Kohn and colleagues develop a potential gene therapy treatment for X-linked lymphoproliferative disease (XLP1), caused by SH2D1A gene mutations affecting SAP, using a lentiviral vector (LV) expressing therapeutic SAP. Through experimental identification and rational design of regulatory elements, XLP-SMART LVs demonstrate enhanced on-target expression in T, NK, and NKT cells.

## Introduction

X-linked lymphoproliferative disease (XLP1), also known as Duncan disease, is an inborn error of immunity caused by mutations in the *SH2D1A* gene, affecting 1 in 1 million males.^1^^,^^2^^,^^3^^,^^4^^,^^5^ The *SH2D1A* gene encodes the SLAM-associated protein (SAP), an adaptor molecule involved in the signaling of immune cell receptors of the SLAM family.^1^^,^^2^^,^^3^^,^^4^^,^^5^ SAP binds to the intracellular domain of SLAM family signaling receptors, and supports the activation or inhibition of immune cell signaling.^2^^,^^4^^,^^6^^,^^7^^,^^8^ SAP mRNA and protein expression are predominately expressed in human thymocytes, T cells, NK cells, and NKT cells.^2^^,^^4^^,^^6^^,^^7^^,^^8^^,^^9^^,^^10^^,^^11^^,^^12^ Patients with XLP1 suffer from impairments in CD4+ T cell function, CD8+ T cell cytotoxicity, NK cell cytotoxicity, plasma cell and memory B cell generation, and NKT cell development.^13^^,^^14^^,^^15^^,^^16^

In over 90% of XLP1 cases, Epstein-Barr virus (EBV) primary infection is the major cause for clinical presentations of the disease.^1^^,^^2^^,^^17^ After EBV infection, XLP1 patients mount a dysregulated immune response, with nearly 60% of patients developing hemophagocytic lymphohistiocytosis (HLH).^1^^,^^8^ HLH treatment is highly immune suppressive and can be significantly toxic.^18^ As such, the mortality associated with HLH presentation is greater than 60%.^1^ Those that survive the EBV infection may develop malignant lymphoma, hypogammaglobulinemia, and lymphoproliferation, and are thus treated with continuous immunoglobulin replacement therapy (IRT) and immune suppression.^9^ The expense and inconvenience of life-long IRT, with administration requiring intravenous or subcutaneous injections, are drawbacks, but are accepted due to the important clinical benefits IRT confers. The only curative treatment includes the use of an allogeneic hematopoietic stem cell (HSC) transplantation, in which CD34+ HSCs are taken from a healthy suitable-matched donor and transplanted into the patient to give rise to a fully functionally immune system.^19^ Many patients, unfortunately, lack this treatment as a viable option due to poor donor availability and immunologic complications. Thus, a more effective treatment for XLP1 remains an unmet need, and—given the severe nature of the disease—an autologous HSC transplantation is a viable approach.^11^ An autologous HSC transplant treats a patient’s own HSCs to integrate a stable copy of the *SH2D1A ex vivo* with a lentiviral vector (LV).^11^ This method of gene therapy may provide the same benefit as an allogeneic transplant, while eliminating any risks of graft rejection or graft-versus-host disease, since each patient serves as their own donor. A successful treatment by autologous HSC transplantation requires an LV capable of transducing a functional copy of *SH2D1A* into long-lived multipotent HSCs expressing within target leukocyte lineages.^1^^,^^11^

A LV was developed previously for gene therapy of XLP1 through LV-mediated gene transfer into autologous HSCs.^11^ However, this LV did not utilize the endogenous regulatory elements of the encoded transgene like many of the current clinical LVs, thereby it did not recapitulate the precise expression pattern of the native *SH2D1A* gene.^11^^,^^20^ Instead, a ubiquitously expressed promoter known as the elongation factor 1 α short promoter (EFS) was used. With this approach, the endogenous promoter alone is insufficient to drive proper physiological expression and regulation of the gene, as additional regulatory elements such as distant enhancer and silencers are also required.^21^ Since SAP expression is tightly regulated within T, NK, and NKT cells, gene expression in off-target HSC populations may pose safety concerns including skewing of hematopoietic potential and dysregulated lymphocyte development and function.^11^^,^^22^^,^^23^

Not all groups utilize ubiquitously expressed promoters within LV gene therapies. Instead, some have employed natural regulatory elements to control promoter activity. This approach has been applied in developing LV gene therapies for conditions such as hemoglobinopathies and IPEX syndrome.^24^^,^^25^ In these specific instances, decades of research were dedicated to identifying key native promoter/enhancer regions. This led to the discovery of the locus control region (LCR) and the conserved noncoding sequence (CNS) areas. The LCR was used to regulate the β-globin promoter in hemoglobinopathy treatments, and the CNS was applied to the FoxP3 promoter for IPEX syndrome therapies.^24^^,^^25^^,^^26^ However, the elucidation of these regions was backed by extensive research, including studying the regulation of the globin genes (specifically *HBB*) and using mouse knockout models to explore and define the regulatory CNS elements within the FoxP3 locus (pertaining to *FOXP3*).^24^^,^^25^^,^^26^ In contrast, our group has advanced this approach utilizing bioinformatics-assisted design to quickly identify and construct LVs driven by the endogenous promoter and regulatory elements necessary to treat inborn errors of immunity such as X-linked chronic granulomatous disease (X-CGD) and Wiskott-Aldrich syndrome.^27^ The approach leveraged the publicly available bioinformatic tool GeneHancer database, which pools data from ENCODE, Ensembl, FANTOM, and VISTA. By integrating data, including histone modification, chromatin accessibility, bound transcription factors, and Hi-C interactions across multiple datasets, we identified various putative enhancer regions that assist with regulating their target gene.^27^ Using this methodology, we recently described an LV with a superior lineage-specific expression pattern compared with an LV currently in clinical trials for X-CGD that uses non-endogenous regulatory elements from other myeloid lineage genes.^27^

We have since optimized this methodology for the efficient identification and testing of endogenous regulatory elements to generate rationally designed bioinformatics-assisted lentiviruses for the treatment of XLP1. Transduction of autologous CD34+ hematopoietic stem and progenitor cells (HSPCs) using LVs that contain regulatory elements of the *SH2D1A* gene can achieve lineage- and stage-specific expression of SAP for maximal therapeutic benefit, with minimal off-target expression in inappropriate cell types. Rationally designed LVs with *SH2D1A* gene-specific promoter/enhancers (XLP-SMART LVs) may achieve functional restoration of humoral and cytotoxic defects in XLP1 patients through gene therapy.

## Results

**Figure 1:**
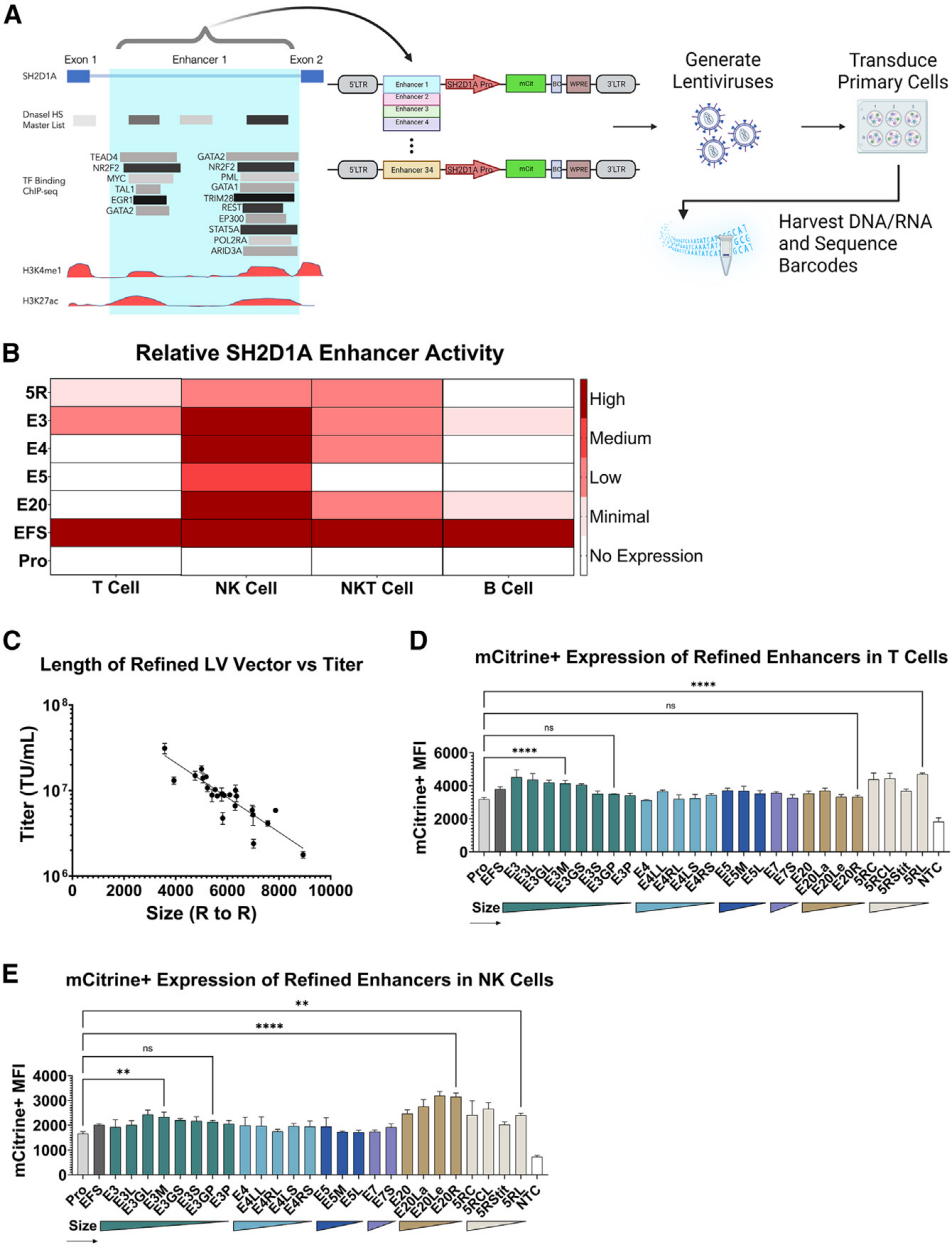
Functional Characterization and Optimization of SH2D1A Enhancers Across T, NK, and B Cell Lineages (A) UCSC Genome Browser interface of the *SH2D1A* locus encoding the SAP protein. Blue shaded columns indicate putative regulatory regions. Distinctive DNase I hypersensitive sites (DHSs) are shown across different cell lineages. Transcription factor (TF) binding, along with peaks of bound H3K4me1, H3K27ac, and DHSs can be used to define the presence and boundaries of putative enhancer elements. Proviral maps of a series of XLP1 “SMART” lentiviral vector constructs each contain a putative regulatory element upstream of the endogenous *SH2D1A* promoter (*SH2D1A* Pro) that drive expression of an mCitrine (mCit) reporter cassette. A unique barcode (BC) is located upstream of the woodchuck hepatitis virus post-transcriptional regulatory element (WPRE) to identify the ability for each element to drive lineage and stage specific expression of the mCit reporter. We transduced primary T, NKT, and NK cells with a pool of raw viral supernatant containing each of the 34 candidate XLP-SMART LVs, in duplicate with different BC, and the EFS-SAP vector as control. B-LCLs were transduced to measure off-target expression in the B lymphocyte lineage. Fourteen days post-transduction, cells were harvested for their gDNA and RNA fractions to measure BC expression and presence in gDNA via next-generation sequencing. (B) Relative *SH2D1A* enhancer activity of the highest-expressing elements. The relative enhancer expression measured by next-generation sequencing of vector barcodes is shown. The highest-expressing 5 enhancers are depicted (see supplemental figures for the remaining 29), with the lowest level of expression (white) denoted as that of the *SH2D1A* promoter only (Pro). Elements of interest contain increased expression over the *SH2D1A* Pro (red). Minimal = 1- to 1.25-fold increase; low = 1.25- to 1.5-fold increase; medium = 1.5- to 2-fold increase; high = >2-fold increase. (C) Proviral size of refined XLP-SMART LV versus titer. Putative enhancers were cloned into the plasmid backbone of a lentiviral vector (pCCL-c-MNDU3-X [Addgene, plasmid no. 81071]) with the MNDU3 promoter first removed, and the vectors were packaged and titered head-to-head. The quantities of infectious particles were plotted as a function of proviral length (bp). Each point in the plot represents an average of three individual 10-cm plates of virus titered on HT-29 cells. Proviral length is defined as sequence length from the beginning of the 5′ long terminal repeat (LTR) U3 through the end of the 3′ LTR U5. *n* = 3 per arm. Linear regression analyses were used to determine the correlation between titer and proviral size (R^2^ = 0.78). (D and E) Expression from LV with refined enhancer elements (via GFP mean fluorescence intensity [MFI]) in T and NK cells. Healthy donor CD3+ T cells or CD56+ NK cells were isolated from peripheral blood mononuclear cells (PBMCs). CD3+ T cells and CD56+ NK cells were transduced with each XLP-SMART LV to achieve a VCN ranging from 0.1 to 0.2. 14 days post-transduction (based on pre-determined titers), T cells were assessed for relative expression driven by each enhancer via mCitrine+ MFI using flow cytometry. Each enhancer was compared with basal *SH2D1A* promoter-driven expression (Pro) and the control LV (EFS). Data are represented as mean ± SD of biological triplicates from two experiments. Statistical significance was analyzed using a one-way ANOVA followed by multiple paired comparisons for normally distributed data (Tukey test). Statistical analysis was performed on all arms, but selected arms are shown. All statistical tests were two-tailed and a *p* value of <0.05 was deemed significant (ns, non-significant; ∗*p* < 0.05, ∗∗*p* < 0.01, ∗∗∗*p* < 0.001, ∗∗∗∗*p* < 0.0001).

To elucidate the putative elements responsible for the lineage- and stage-specific expression of the endogenous *SH2D1A* locus, we employed a bioinformatics-guided approach utilizing GeneHancer, a bioinformatic tool that links over 285,000 candidate enhancer elements across the human genome to their respective target genes.^28^ GeneHancer integrates four genome-wide enhancer databases (ENCODE, Ensembl, FANTOM, and VISTA) to generate a comprehensive list of putative regulatory elements for each gene. By integrating data, including histone modification, chromatin accessibility, bound transcription factors, and Hi-C interactions, we identified 34 potential regulatory elements of *SH2D1A*, located within a 200 kb window of the *SH2D1A* transcription start site (Table S1). We entered each genomic coordinate into the UCSC Genome Browser to examine the corresponding regulatory elements (Figure 1A).

We designed a series of LVs (XLP1-SMART LVs) to test each element’s enhancer capability independently (Figure 1A). The elements were inserted upstream of a 600 bp *SH2D1A* promoter—identified via the Eukaryotic Promoter Database —to drive expression of an mCitrine (mCit) reporter cassette. The mCitrine gene is a variant of eGFP, with increased fluorescent intensity over the traditional GFP fluorophore.^27^ Furthermore, each vector was designed with the woodchuck hepatitis virus post-transcriptional regulatory element (WPRE) replacing the endogenous 3′ UTR of *SH2D1A* to enhance expression of the transgene cassette. To multiplex the series of vectors, unique 20 bp barcodes were cloned directly upstream of the WPRE (Figure 1A). At this location, the barcode remains within the transcript and vector provirus genome for identification and normalization by RNA or DNA, respectively, but it will not be translated to affect any protein function. The barcodes were designed, each with a Hamming distance of 10, to tolerate accidental mutations.^29^ Each of the 34 vectors was cloned twice—each with its own unique barcode—to further assess any potential biases from transduction, recombination, or PCR amplification. An LV used in pre-clinical XLP1 gene therapy studies, EFS-SAP, was cloned in duplicate as a control.^11^ The *SH2D1A* gene was replaced with an mCit reporter and termed EFS-mCit.

To mimic the endogenous expression of SAP, we tested each LV construct for its ability to drive high-level expression in primary T cells, NKT cells, and NK cells, each isolated from healthy donor peripheral blood mononuclear cells (PBMCs). B lymphoblastoid cell lines (B-LCLs) were utilized to assess off-target B lymphoid expression of each regulatory element. We transduced each cell population with the 34 candidate vectors and the EFS control. Fourteen days post-transduction, cells were harvested to isolate their gDNA and mRNA fractions. Bulk vector copy number (VCN) of the transduced populations was assessed via droplet digital PCR (ddPCR). The barcode-containing DNA regions were PCR amplified from the gDNA fraction and assessed via next-generation sequencing. Furthermore, the barcode-containing regions were PCR amplified from the RNA fraction after cDNA conversion and assessed via next-generation sequencing. The RNA barcode counts are proportionate to the transcriptional activity of the enhancers within each lineage. The genomic (DNA) barcode count is used to normalize each barcode in the transcript to account for potential differences in transduction efficiency of the vectors. The number of RNA barcode reads normalized to frequency of gDNA barcodes within each cell type determined the relative expression directed by each element (Figure 1A).

Of the 34 regulatory elements tested, elements 3 and 5R demonstrated T cell-specific enhancer expression (Figures 1B and S1A–S1C). Elements 3, 4, 5, 7, 20, and 5R demonstrated NK and NKT cell-specific enhancer expression (Figures 1B, and S1D–S1E). All 34 regulatory elements tested demonstrated minimal to no off-target expression in B-LCLs (Figures 1B and S1F).

Enhancers 3, 4, 5, 7, 20, and 5R were refined to decrease their size (thereby increasing the vector titers and gene transfer) with the goal of retaining high enhancer-driven function (Figures 1C–1E). LV plasmids were designed with the refined enhancer fragments and the 600 bp *SH2D1A* promoter driving expression of an mCit cassette. LV plasmids were packaged and titered head-to-head using methods previously described by our laboratory (Figure 1C).^26^^,^^27^^,^^30^ Primary T and NK cells were transduced with the refined LVs to achieve equivalent VCNs ranging from ∼0.10 to 0.20 to increase the probability of each transduced cell containing a single integrant.^27^ The mean fluorescence intensity (MFI) of the mCit+ populations were assessed via flow cytometry to compare the ability of each refined enhancer element to drive mCit expression in T cells (Figure 1D) and NK cells (Figure 1E). Enhancers E3M and 5RL retained 90% and 100% of the full-length enhancer activity in T cells, while reducing the enhancer sizes by 3.5 and 4 kb, respectively, from the original elements (Figure 1D). Enhancers 3M, 3GP, 20R, and 5RL retained 120%, 110%, 130%, and 100% of the full-length enhancer activity in NK cells, while reducing the enhancer sizes by 3.5, 4.2, 1, and 4 kb, respectively (Figure 1E). Enhancers 4, 5, and 7 and their shortened iterations did not significantly increase expression in T cells or NK cells when compared with the promoter-only control and were thus removed from subsequent analyses (Figures 1D and 1E). After refining the enhancers, the elements were combined to assess additive and/or synergistic effects on expression.

**Figure 2:**
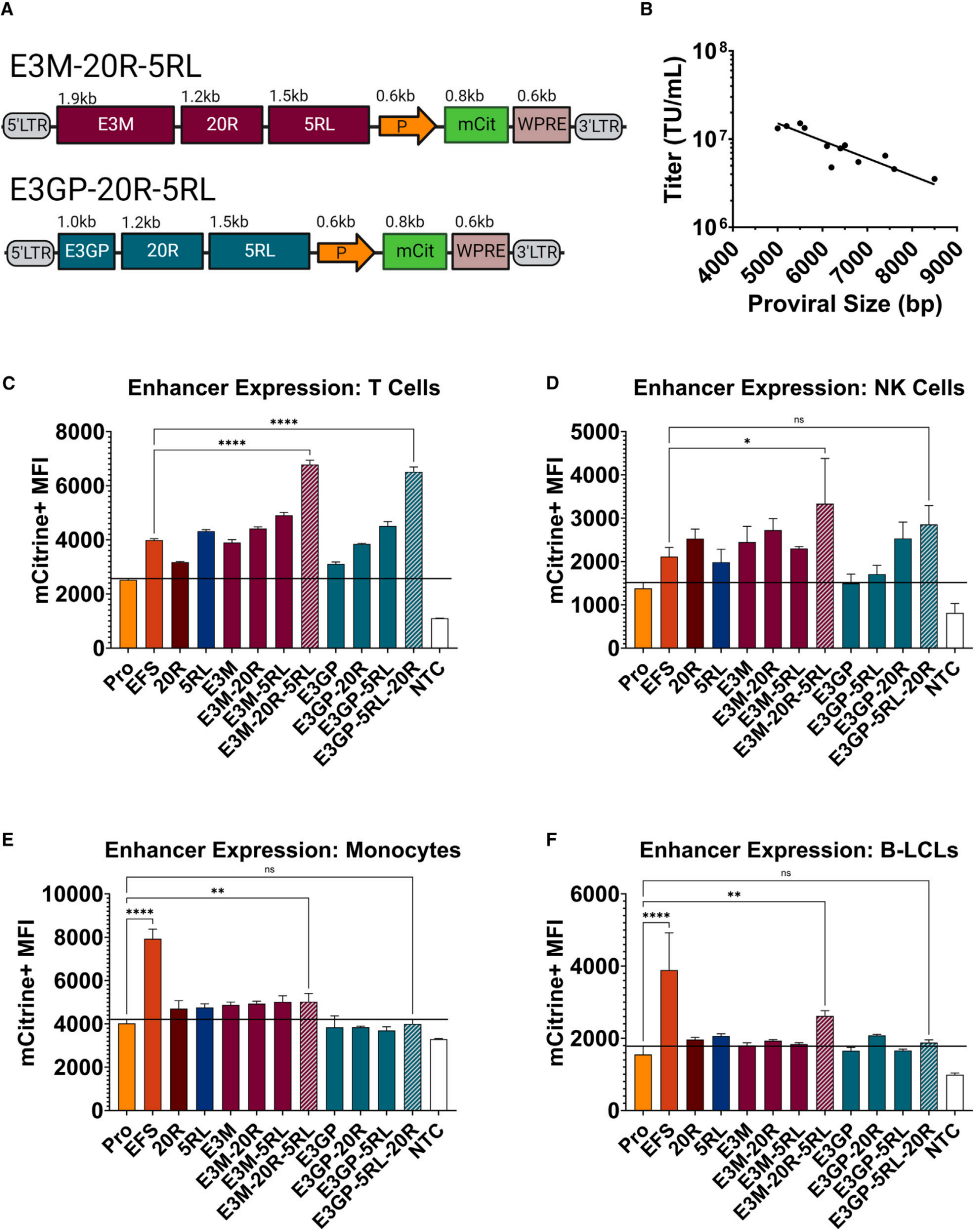
In Vitro Characterization of Composite XLP1-SMART Lentiviral Vectors: Proviral Size, Titer, and Cell Lineage-Specific Expression (A) Schematic of composite XLP1-SMART lentiviral vectors. Diagrams of the two XLP-SMART LV composite constructs are shown with their sequence lengths (kb). 5′ LTR and 3′ LTR designate the 5' and 3′ viral long terminal repeats (LTRs), respectively; E3M, E3GP, 20R, and 5RL are *SH2D1A* enhancer elements; P designates the *SH2D1A* promoter; mCit, mCitrine reporter cassette; WPRE, woodchuck hepatitis virus post-transcriptional regulatory element. (B) Proviral size of composite XLP-SMART LVs versus titer. Enhancers were cloned into the plasmid backbone of a therapeutic lentiviral vector (pCCL-c-MNDU3-X [Addgene, plasmid no. 81071]) (with the MNDU3 promoter removed), packaged, and titered head-to-head. The quantities of infectious particles were plotted as a function of proviral length (bp). Each point in the plot represents an average of three individual 10-cm plates of virus titered on HT-29 cells. Proviral length is defined as sequence length from the beginning of the 5′ long terminal repeat (LTR) U3 through the end of the 3′ LTR U5. *n* = 3 per arm. Linear regression analyses were used to determine the correlation between titer and proviral size (R^2^ = 0.79). (C) On-target expression in T cells *in vitro* of composite XLP-SMART LVs. Healthy donor CD3+ T cells were isolated from PBMCs. CD3+ T cells were transduced with each XLP-SMART LV to achieve a VCN ranging from 0.1 to 0.2. At 14 days post-transduction, T cells were assessed for the relative expression driven by each enhancer via mCitrine+ MFI using flow cytometry. Each enhancer was compared with basal *SH2D1A* promoter expression (Pro), the control LV (EFS), and a non-transduced control (NTC). Data are represented as mean ± SD of biological triplicates from three experiments. Statistical significance was analyzed using a one-way ANOVA followed by multiple paired comparisons for normally distributed data (Tukey test). Statistical analysis was performed on all arms, but selected arms are shown. All statistical tests were two-tailed and a *p* value of <0.05 was deemed significant (ns, non-significant; ∗*p* < 0.05, ∗∗*p* < 0.01, ∗∗∗*p* < 0.001, ∗∗∗∗*p* < 0.0001). (D) On-target expression in NK cells *in vitro* of composite XLP-SMART LVs. Healthy donor CD56+ NK cells were isolated from PBMCs. CD56+ NK Cells were transduced with each XLP-SMART LV to achieve a VCN ranging from 0.1 to 0.2. At 14 days post-transduction, NK cells were assessed for the relative expression driven by each enhancer via mCitrine+ MFI using flow cytometry. Each enhancer was compared with basal *SH2D1A* promoter expression (Pro) and the control LV (EFS). Data are represented as mean ± SD of biological triplicates from three experiments. Statistical significance was analyzed using a one-way ANOVA followed by multiple paired comparisons for normally distributed data (Tukey test). Statistical analysis was performed on all arms, but selected arms are shown. All statistical tests were two-tailed and a *p* value of <0.05 was deemed significant (ns, non-significant; ∗*p* < 0.05, ∗∗*p* < 0.01, ∗∗∗*p* < 0.001, ∗∗∗∗*p* < 0.0001). (E) Off-target expression in CB CD34+ differentiated monocytes cells by composite XLP-SMART LVs. Healthy donor CB CD34+ cells were differentiated into monocytes as described.^31^ Prior to differentiation, CB CD34+ cells were transduced with each XLP-SMART LV to achieve a VCN ranging from 0.1 to 0.2. At 14 days post-transduction and differentiation, CD14+CD16+ monocytes were assessed for the relative expression driven by each enhancer via mCitrine+ MFI using flow cytometry. Each enhancer was compared with basal *SH2D1A* promoter expression (Pro) and the control LV (EFS). Data are represented as mean ± SD of biological triplicates from one experiment. Statistical significance was analyzed using a one-way ANOVA followed by multiple paired comparisons for normally distributed data (Tukey test). Statistical analysis was performed on all arms, but selected arms are shown. All statistical tests were two-tailed and a *p* value of <0.05 was deemed significant (ns, non-significant; ∗*p* < 0.05, ∗∗*p* < 0.01, ∗∗∗*p* < 0.001, ∗∗∗∗*p* < 0.0001). (F) Off-target expression in B-LCLs by composite XLP-SMART LVs. B-LCLs, cultured in R10, were transduced with each XLP-SMART LV to achieve a VCN ranging from 0.1 to 0.2. At 14 days post-transduction, B-LCLs were assessed for the relative expression driven by each enhancer via mCitrine+ MFI using flow cytometry. Each enhancer was compared with basal *SH2D1A* promoter expression (Pro) and the control LV (EFS). Data are represented as mean ± SD of biological triplicates from three experiments. Statistical significance was analyzed using a one-way ANOVA followed by multiple paired comparisons for normally distributed data (Tukey test). Statistical analysis was performed on all arms, but selected arms are shown. All statistical tests were two-tailed and a *p* value of <0.05 was deemed significant (ns, non-significant; ∗*p* < 0.05, ∗∗*p* < 0.01, ∗∗∗*p* < 0.001, ∗∗∗∗*p* < 0.0001).

Enhancer E3M was combined with either 20R, 5RL or both 20R and 5RL, and a 600 bp *SH2D1A* promoter to drive expression of an mCit cassette (Figure 2A). Similarly, LV plasmids were packaged and titered head-to-head using methods previously described by our lab (Figure 2B).^26^^,^^27^^,^^30^ We transduced primary T and NK cells for evaluating on-target expression (Figures 2C and 2D). Transduced cord blood (CB) CD34+ cells were differentiated into monocytes and, along with B-LCLs, were used to determine off-target expression levels (Figures 2E and 2F).

Enhancers E3M/E3GP, 20R, and 5RL demonstrated additive expression levels when combined and evaluated in primary T and NK cells (Figures 2C and 2D). The combination vectors (XLP1-SMART LVs) increased mCit expression in T cells up to 4-fold compared with the promoter-only control and up to 1.8-fold greater than the control EFS vector (Figure 2C). Furthermore, the combination vectors increased mCit expression in NK cells 2-fold greater than the promoter-only control and 1.5-fold greater than the EFS preclinical vector (Figure 2D). XLP1-SMART LVs that included E3M showed minor levels of off-target expression, with the E3M combination XLP-SMART LV (E3M-20R-5RL-mCit) harboring a 1.25- or 1.67-fold increase in mCit expression levels in monocytes and B cells, respectively, over the promoter-only control. However, the E3GP combination XLP-SMART LV (E3GP-20R-5RL-mCit) showed no significant levels of off-target expression (Figures 2E and 2F).

To determine the lineage- and stage-specific expression of the XLP1-SMART LVs, we tested the ability of each LV to drive mCit reporter expression *in vivo* in NSG-Tg(Hu-IL15)-immunodeficient mice. Healthy human donor CB CD34+ HSCs were pre-stimulated for 24 h in 50 ng/mL of human hSCF, hTPO, and hFT3L before transduction with 2e–7 TU/mL of our XLP-SMART LVs. The E3M-20R-5RL and E3GP-20R-5RL LVs were compared with both mock transduced cells and to CB CD34+ HSCs transduced with 2e–7 TU/mL of the control EFS-mCit vector. We transplanted the transduced cells via intra-hepatic injection into sub-lethally irradiated NOD.Cg-*Prkdc*^*scid*^ *Il2rg*^*tm1Wjl*^ Tg(IL15)1Sz/SzJ (NSG-Tg[HuIL15]) neonatal pups receiving 150 rads of irradiation. These NSG-Tg(HuIL15) mice express human IL-15 to help enhance the development of human NK cells in mice engrafted with human CD34+ cells.^32^ Furthermore, the transplantation of NSG neonatal pups supports more efficient human T cell development after HSC injection than adult NSG mice.^33^

**Figure 3:**
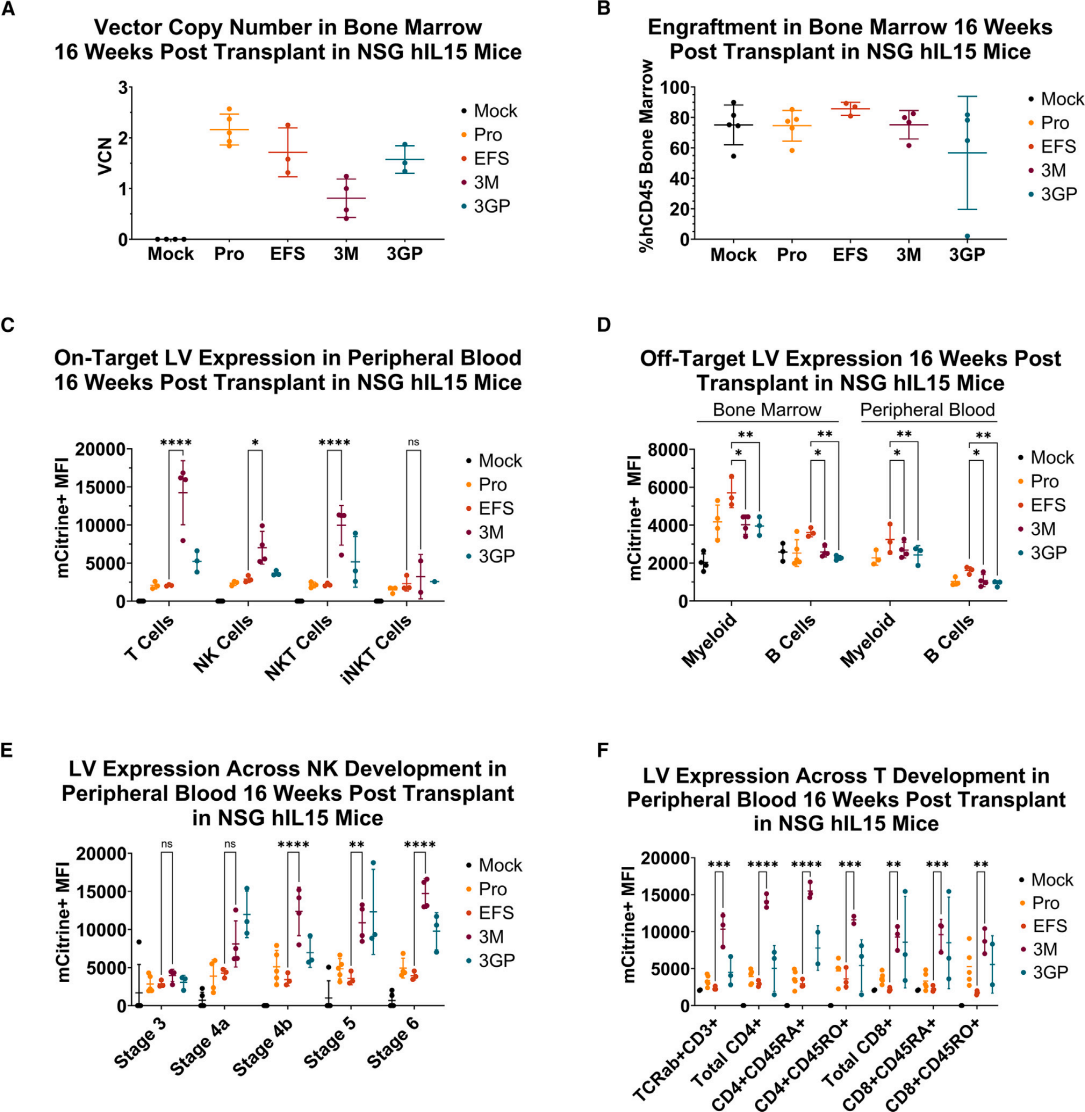
Lineage-Specific Expression of XLP-SMART Lentiviral Vectors in NSG-Tg(Hu-IL15) Mice (A) Vector copy number 16 weeks post-transplant from NSG-Tg(Hu-IL15) mouse bone marrow (BM). Whole BM was taken from each mouse at time of euthanasia and processed into a single-cell suspension. Genomic DNA was extracted from the BM suspension and analyzed for vector copy number by ddPCR. *n* = 4, mock; *n* = 5, promoter only (Pro); *n* = 3, EFS-mCitrine (EFS); *n* = 4, E3M-20R-5RL-mCitrine (3M); *n* = 3, E3GP-20R-5RL-mCitrine (3GP). Data are represented as mean ± SD of biological replicates from one experiment. (B) Engraftment 16 weeks post-transplant from NSG-Tg(Hu-IL15) mouse BM. Whole BM was taken from each mouse at time of euthanasia and analyzed for engraftment by flow cytometry using an anti-hCD45 antibody. *n* = 5, Mock; *n* = 5, promoter only (Pro); *n* = 3, EFS-mCitrine (EFS); *n* = 4, E3M-20R-5RL-mCitrine (3M); *n* = 4, E3GP-20R-5RL-mCitrine (3GP). (C) On-target XLP-SMART LV expression in peripheral blood 16 weeks post-transplant in NSG-Tg(Hu-IL15) mice. Mice were bled at 16 weeks post-transplant to analyze peripheral blood for XLP-SMART LV expression. Lysed red blood cells were stained for various on-target lineages within the hCD45+ gate (T cells: hCD33–, hCD19–, hCD3+; NK cells: hCD33-, hCD3-, hCD19-, hCD56+; NKT cells: hCD33-, hCD19-, hCD3+, hCD56+; and iNKT cells: hCD33–, hCD19–, hCD3+, hCD56+, hVα24+). Each LV’s relative expression was measured in on-target lineages via mCitrine+ MFI using flow cytometry. Each enhancer was compared with basal *SH2D1A* promoter-driven expression (Pro) and the control LV (EFS). Data are represented as mean ± SD of biological triplicates from one experiment. Statistical significance was analyzed using a two-way ANOVA followed by multiple paired comparisons for normally distributed data (Tukey test). Statistical analysis was performed on all arms, but selected arms are shown. All statistical tests were two-tailed and a *p* value of <0.05 was deemed significant (ns, non-significant; ∗*p* < 0.05, ∗∗*p* < 0.01, ∗∗∗*p* < 0.001, ∗∗∗∗*p* < 0.0001). (D) Off-target XLP-SMART LV expression in BM and peripheral blood (PB) 16 weeks post-transplant in NSG-Tg(Hu-IL15) mice. Whole BM was taken from each mouse at time of euthanasia and mice were bled at 16 weeks post-transplant to analyze PB for XLP-SMART LV expression. Whole BM, processed into a single-cell suspension, and red blood cell-lysed PB were stained for various off-target lineages within the hCD45+ gate (myeloid cells: hCD33+; B cells: hCD33–, hCD19+, hCD3–). Each LV’s relative expression was measured in off-target lineages via mCitrine+ MFI using flow cytometry. Each enhancer was compared with basal *SH2D1A* promoter expression (Pro) and the control LV (EFS). Data are represented as mean ± SD of biological triplicates from one experiment. Statistical significance was analyzed using a two-way ANOVA followed by multiple paired comparisons for normally distributed data (Tukey test). Statistical analysis was performed on all arms, but selected arms are shown. All statistical tests were two-tailed and a *p* value of <0.05 was deemed significant (ns, non-significant; ∗*p* < 0.05, ∗∗*p* < 0.01, ∗∗∗*p* < 0.001, ∗∗∗∗*p* < 0.0001). (E) XLP-SMART LV expression across NK cell development in PB 16 weeks post-transplant in NSG-Tg(Hu-IL15) mice. Mice were bled at 16 weeks to analyze PB for XLP-SMART LV expression after red blood cell lysis. Cells were stained for various stages of NK cell differentiation within the hCD45+CD33– gate (stage 1 [data not shown]: hCD34+; stage 2a [data not shown]: hCD34+, hCD117+, hCD122–; stage 2b [data not shown]: hCD34+, hCD117+, hCD122+; stage 3: hCD34–, hCD117+, hCD122+, hCD56–; stage 4a: hCD34–, hCD117+, hCD122+, hCD56+, hCD94+; stage 4b: hCD34–, hCD117–, hCD122+, hCD56+, hCD94+, hNKp80+; stage 5: hCD34–, hCD117–, hCD122+, hCD56+, hCD94+, hNKp80+, hCD16+; and stage 6: hCD34–, hCD117–, CD122+, hCD56+, hCD94+, hNKp80+, hCD16+, hCD57+). Each LV’s relative expression was measured across NK cell subpopulations via mCitrine+ MFI using flow cytometry. Each enhancer was compared with basal *SH2D1A* promoter expression (Pro) and the control LV (EFS). Data are represented as mean ± SD of biological triplicates from one experiment. Statistical significance was analyzed using a two-way ANOVA followed by multiple paired comparisons for normally distributed data (Tukey test). Statistical analysis was performed on all arms, but selected arms are shown. All statistical tests were two-tailed and a *p* value of <0.05 was deemed significant (ns, non-significant; ∗*p* < 0.05, ∗∗*p* < 0.01, ∗∗∗*p* < 0.001, ∗∗∗∗*p* < 0.0001). (F) XLP-SMART LV expression across T cell development in PB 16 weeks post-transplant in NSG-Tg(Hu-IL15) mice. Mice were bled at 16 weeks to analyze PB for XLP-SMART LV expression after red blood cell lysis. Cells were stained for various stages of mature T cell populations within the hCD45+ hCD34– hCD14– hCD19– hCD56– hCD5+ hCD7+ TCRab+ CD3+ gate (total CD4: hCD4+, hCD8–; CD4+CD45RA+: hCD4+, hCD8–, hCD45RA+, hCD45RO–; CD4+CD45RO+: hCD4+, hCD8–, hCD45RA–, hCD45RO+; total CD8: hCD4–, hCD8+; CD8+CD45RA+: hCD4–, hCD8+, hCD45RA+, hCD45RO–; CD8+CD45RO+: hCD4–, hCD8+, hCD45RA–, hCD45RO+). Each LV’s relative expression was measured in mature T cell subsets via mCitrine+ MFI using flow cytometry. Each enhancer was compared with basal *SH2D1A* promoter expression (Pro) and the control LV (EFS), both harboring an mCitrine reporter cassette. Data are represented as mean ± SD of biological triplicates from one experiment. Statistical significance was analyzed using a two-way ANOVA followed by multiple paired comparisons for normally distributed data (Tukey test). Statistical analysis was performed on all arms, but selected arms are shown. All statistical tests were two-tailed and a *p* value of <0.05 was deemed significant (ns, non-significant; ∗*p* < 0.05, ∗∗*p* < 0.01, ∗∗∗*p* < 0.001, ∗∗∗∗*p* < 0.0001).

Mice were sacrificed 16 weeks post-transplantation to assess engraftment and vector-mediated gene expression in multiple human hematopoietic cell lineages. Bone marrow, thymus, and spleen were processed into single-cell suspensions and peripheral blood was collected. The VCN was determined for the transplanted cells within the bone marrow of each mouse (Figure 3A) and the bone marrow engraftment (Figure 3B) of each mouse was quantified via the percent of hCD45+ human cells via flow cytometry. Average VCNs for promoter-only (Pro), EFS-mCit (EFS), E3M-20R-5RL-mCit (3M), and E3GP-20R-5RL-mCit (3GP) in the bone marrow compartment of NSG-Tg(Hu-IL15) mice were 2.17, 1.72, 0.81, and 1.57, respectively. With exception of one mouse that did not engraft in the E3GP-20R-5RL-mCit group, average human cell engraftment was 75% for all groups.

We evaluated lineage-specific expression of the XLP-SMART LVs by analyzing the MFI of mCit+ expression in different on- and off-target populations of hCD45+ cells. Mice transplanted with CB CD34+ HSCs transduced with the E3M-20R-5RL-mCit LV contained 7.1-, 2.8-, and 4.3-fold brighter mCit+ expression than the EFS-mCit vector in T cells, NK cells, and NKT cells, respectively (Figure 3C). Mice transplanted with CB CD34+ HSCs transduced with the E3GP-20R-5RL-mCit LV contained 2.0-, 1.6-, and 2.0-fold brighter mCit+ expression than the EFS-mCit vector in T cells, NK cells, and NKT cells, respectively (Figure 3C). Furthermore, mice transplanted with CB CD34+ HSCs transduced with E3M-20R-5RL-mCit or E3GP-20R-5RL-mCit LVs contained no off-target expression in myeloid cells or B cells in both the bone marrow and peripheral blood compartments, unlike the EFS-mCit counterpart (Figure 3D).

To evaluate the stage-specific expression of the XLP1-SMART LVs, we stained the bone marrow, thymus, and spleen cell suspensions with an NK cell or T cell differentiation antibody panel and evaluated their mCit+ expression via flow cytometry (Figures 3E, 3F, and S2A–2D). Mice transplanted with CB CD34+ HSCs transduced with the E3M-20R-5RL-mCit LV contained 1.7- to 3.7-fold brighter mCit+ expression than the EFS-mCit vector in mature NK lineages (stage 4a to stage 6; Figure 3E). Mice transplanted with CB CD34+ HSCs transduced with the E3GP-20R-5RL-mCit LV contained 2.1- to 3.5-fold brighter mCit+ expression than the EFS-mCit vector in mature NK lineages (stage 4a to stage 6; Figure 3E). Furthermore, the mCit+ expression in mature NK lineages from mice treated with E3M-20R-5RL-mCit LV mirrors the natural SAP expression seen in mature NK lineages of a healthy donor’s peripheral blood (Figures S3B and S3D). The mice with the E3M-20R-5RL-mCit LV had 4.7- to 5.0-fold brighter mCit+ expression than cells in mice receiving the EFS-mCit vector in mature T lineages (Figures 3F, S2A, and S2B). The mice with the E3GP-20R-5RL-mCit LV had 2.0- to 4.4-fold brighter mCit+ expression than cells in mice receiving the EFS-mCit vector in mature T lineages (Figures 3F, S2A, and S2B). In addition, the mCit+ expression measured across mature T lineages in the thymus of mice with the E3M-20R-5RL-mCit LV mirrored the pattern of SAP expression across T development from a heathy donor thymus (Figures S3A and S3C).

To further test the stage specificity of the XLP1-SMART LVs, we utilized the artificial thymic organoid (ATO) system, which fully recapitulates thymopoiesis from multiple stem cell sources.^34^^,^^35^ Human CD34+ mobilized peripheral blood (mPB) cells were transduced with E3M-20R-5RL-mCit LV, E3GP-20R-5RL-mCit LV, and EFS-mCit at equivalent VCN. Transduced mPB cells and a mock non-transduced control were combined with the MS5-hDLL4 stromal cell line which constitutively expresses human Notch delta-like ligand 4 (DLL4). Cell count and T cell differentiation kinetics were measured at weeks 3, 7, and 12 using flow cytometry, and the percentage of mCitrine expression was compared with endogenous SAP expression to assess the temporal specificity of the XLP1-SMART LVs across multiple stages of T cell differentiation. Thymocytes from ATOs with XLP1-SMART LVs expressed mCitrine at all stages of T cell development, consistent with the presence of SAP expression throughout T cell differentiation from uncommitted to mature thymocytes (Figures S4A and S4B).

To assess both the clonogenic potential of human hematopoietic progenitor cells after transduction with the LV and the functional restoration of the XLP1 phenotype, the mCitrine open reading frame of the XLP1-SMART LVs was replaced with the coding region of human *SH2D1A*, codon optimized using the JCat codon optimization algorithm (Figures S5A and S5B).^36^ Both JCat and GeneArt algorithms were tested, changing the codons of wild-type *SH2D1A* with synonymous human codon changes to thus increase protein production (Table S2). The algorithms differ based on their considerations of codons that assist in ribosome stalling, mRNA translation, mRNA stability, and premature termination of translation.^36^^,^^37^ Both optimizations were tested in *SH2D1A−/−* Jurkat cells for their relative protein production via western blotting, with JCat producing the most protein per VCN (Figures S5A and S5B).

After determining the increased *SH2D1A* expression using a JCat codon optimization, in comparison with wild-type *SH2D1A,* codon optimized XLP1-SMART LV plasmids were packaged and titered head-to-head using methods described previously.^26^^,^^27^^,^^30^

To measure the efficacy of XLP1-SMART LVs to produce SH2D1A protein, we conducted a dose response in the *SH2D1A−/−* Jurkat cells, transducing the cells with the JCat codon optimized XLP1-SMART LVs to achieve VCNs of 1, 3, and 5. At equal protein concentrations, XLP1-SMART LVs harboring the E3M enhancer demonstrated detectable levels of SH2D1A protein at VCNs of 3 and 5, reaching approximately 23% and 34% wild-type SAP protein, respectively (Figures S5C and S5D; Table S3). Furthermore, XLP1-SMART LVs harboring the E3GP enhancer demonstrated detectable levels of SH2D1A protein at VCNs of 3 and 5, reaching approximately 10% and 31% wild-type SAP protein, respectively (Figures S5C and S5D; Table S3).

To assess the viability of XLP1-SMART LVs as a gene therapy tool for XLP1 patients, we tested the XLP1-SMART LVs for their functional correction of XLP1 patient cells. Bone marrow (BM) CD34+ cells were obtained from XLP1 patients after their informed consent (UCLA IRB no. 10-001399). XLP1 patient CD34+ cells were pre-stimulated for 24 h in 50 ng/mL of human hSCF, hTPO, and hFT3L before transduction with the XLP1-SMART LVs. Cells were transduced to achieve equivalent VCNs of ∼1.1 and ∼2.4 (EFS = 1.1 and 3.0; E3M = 0.9 and 1.7; E3GP = 1.2 and 2.52) (based on prior dose-response testing of VCN produced by each LV across a range of concentrations). Transduced CD34+ cells were used to conduct a colony-forming unit (CFU) assay for progenitor cell clonogenic potential and were also differentiated to NK cells to assess XLP1 functional restoration via an NK cell cytotoxicity assay.

**Figure 4:**
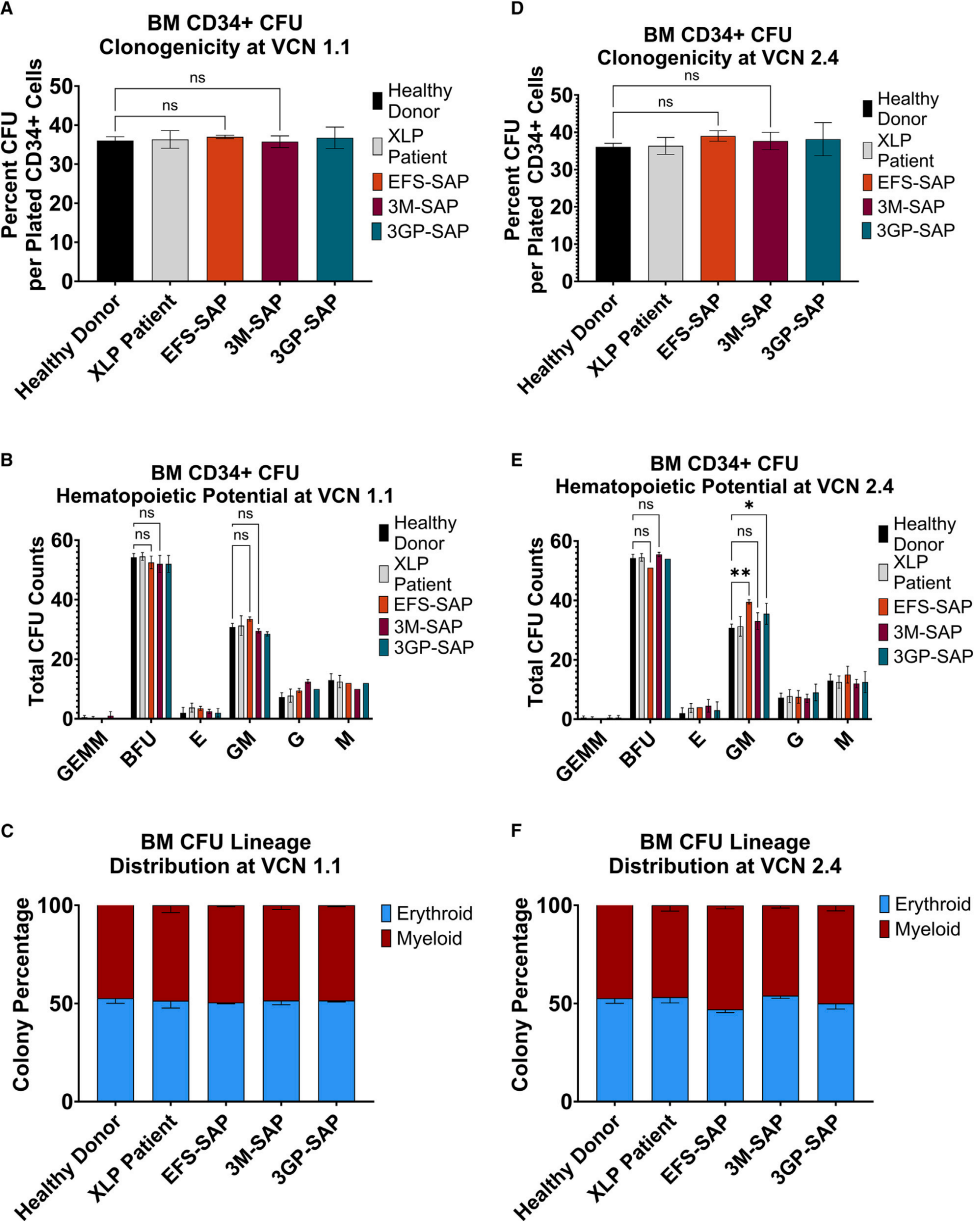
Colony Forming Unit (CFU) Assay of XLP-SMART Lentiviral Vectors in Healthy Donor and XLP1 Patient Bone Marrow CD34+ Cells BM CD34+ cells from a healthy donor and an XLP1 patient were prestimulated for 24 h with 50 ng/mL each of human stem cell factor (hSCF), human thrombopoietin (hTPO), and human FMS-like tyrosine kinase 3 ligand (hFlt3-L) before transduction with XLP1-SMART LVs. 24 h after transduction, 100, 300, and 900 BM CD34+ HSPCs per replicate were plated in MethoCult. After 14 days of culture at 5% CO_2_, 37°C, and humidified atmosphere, the number of mature colonies were scored under the microscope for total colony-forming units (CFU) at a VCN of 1.1 (A) or 2.4 (D); total hematopoietic progenitor cell counts at a VCN of 1.1 (B) or 2.4 (E), denoted as myeloid (G/M/GM), erythroid (BFU/E), or mixed (GEMM); and, finally, percentage of total myeloid or erythroid lineage distribution for cells at a VCN of 1.1 (C) or 2.4 (F). Data are represented as mean ± SD of biological duplicates from one experiment. Clonogenicity was analyzed for statistical significance using a one-way ANOVA followed by multiple paired comparisons for normally distributed data (Tukey test). CFU hematopoietic potential was analyzed for statistical significance using a two-way ANOVA followed by multiple paired comparisons for normally distributed data (Tukey test). Statistical analysis was performed on all arms, but selected arms are shown. All statistical tests were two-tailed and a *p* value of <0.05 was deemed significant (ns, non-significant;∗*p* < 0.05, ∗∗*p* < 0.01, ∗∗∗*p* < 0.001, ∗∗∗∗*p* < 0.0001).

Transduced XLP1 patient cells were seeded in semi-solid methylcellulose medium and cultured for 14 days before quantifying vector effect on clonogenicity and generation of hematopoietic progenitor colonies (Figures 4A–4C). At a VCN of 1, XLP1-SMART LVs demonstrated no changes to clonogenicity or hematopoietic lineage skewing in comparison with a healthy donor control (Figures 4A–4C). Similarly, patient cells transduced with the EFS-SAP vector, at a VCN of 1.1, demonstrated no significant skewing (*p* < 0.05) into the myeloid lineage when compared with a healthy donor control (Figures 4B and 4C). At a VCN of 1.7, E3M-20R-5RL-SAP (3M-SAP) XLP1-SMART LVs still demonstrated no changes to clonogenicity or hematopoietic lineage skewing in comparison with a healthy donor control (Figures 4D–4F). Conversely, patient cells transduced with the EFS-SAP vector at a VCN of 3.0 demonstrated significant skewing (*p* < 0.05) into the myeloid lineage, with a 1.3-fold increase in granulocyte-macrophage (GM) colonies compared with a healthy donor control (Figures 4E and 4F). At a VCN of 2.52, E3GP-20R-5RL-SAP (3GP-SAP) also demonstrated significant skewing into the myeloid lineage with a 1.15-fold increase in GM colonies compared with a healthy donor control (Figures 4E and 4F).

**Figure 5:**
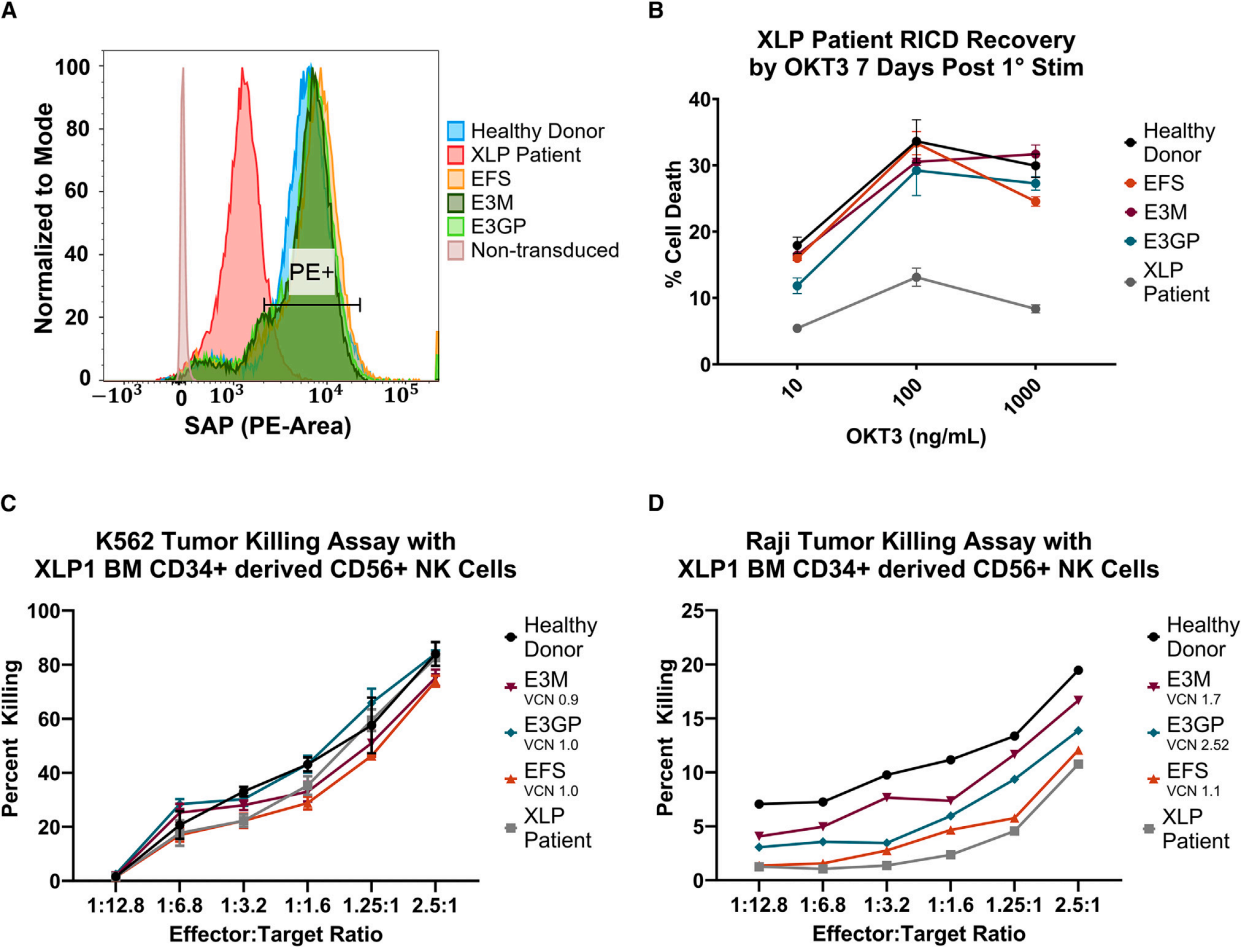
Functional Restoration and Cytotoxic Activity of XLP1-SMART Lentiviral Vectors in CD8+ T Cells and NK Cells from XLP1 Patients (A) FACS representation of SAP protein restoration in XLP1 patient CD8+ T cells. CD8+ T cells from a healthy donor (HD) and an XLP1 patient were isolated from PBMCs and transduced with XLP-SMART LVs. Ten days after transduction, cells were fixed, permeabilized, and stained for SAP protein using an anti-SAP monoclonal antibody. Stained cells were then assessed for their SAP expression via total SAP MFI within each target subpopulation using flow cytometry. (B) T cell restimulation-induced cell death (RICD) assay of XLP1 patient CD8+ T cells transduced with XLP-SMART LVs. CD8+ T cells from a HD and an XLP1 patient were isolated from PBMCs and transduced with XLP-SMART LVs. Ten days after transduction, cells were plated for RICD assay in OKT3 at final concentrations of 1,000, 100, and 10 ng/mL. After 24 h, XLP-SMART LV transduced cells were taken to measure the recovery of RICD in comparison with an EFS-SAP transduced condition and an HD control. The number of live cells (PI–) in stimulated controls were compared with unstimulated controls to measure the percent cell loss = [1 – (no. of PI– restimulated cells/no. of PI– untreated cells)] × 100. Data are represented as mean ± SD of biological triplicates from one experiment. Statistical significance was analyzed using a two-way ANOVA followed by multiple paired comparisons for normally distributed data (Tukey test). All statistical tests were two-tailed and a *p* value of <0.05 was deemed significant (ns, non-significant; ∗*p* < 0.05, ∗∗*p* < 0.01, ∗∗∗*p* < 0.001, ∗∗∗∗*p* < 0.0001). Compared with SH2D1A knockout (KO) T cells, the EFS, E3M, and E3GP conditions were deemed significant with a *p* value < 0.0001 (data not shown). (C) K562 NK cell cytotoxicity assay of XLP1 patient BM CD34+ cells transduced with XLP-SMART LVs. BM CD34+ cells from a HD and an XLP1 patient were transduced with XLP-SMART LVs and differentiated into CD56+ NK cells using the StemSpan NK Cell Generation Kit. On day 28 of differentiation, CD56+ NK cells were enriched using magnetic bead isolation and serially diluted with target cells at various effector to target (K562) ratios: 2.5:1, 1.25:1, 1:1.6, 1:3.2, 1:6.8, and 1:12.8. After 18 h of incubation, GFP+ tumor cells were counted via FACS to assess NK cytotoxicity and normalized to target only control wells. Due to limited XLP1 patient cells, data are represented as single replicates from one experiment. Statistical significance was analyzed using a two-way ANOVA followed by multiple paired comparisons for normally distributed data (Tukey test). All statistical tests were two-tailed and a *p* value of <0.05 was deemed significant (ns non-significant; ∗*p* < 0.05, ∗∗*p* < 0.01, ∗∗∗*p* < 0.001, ∗∗∗∗*p* < 0.0001). Compared with XLP1 patient samples, Healthy donor was deemed significant at a *p* value < 0.05; EFS was deemed significant with a *p* value < 0.001; E3M was deemed not significant; and E3GP was deemed significant with a *p* value < 0.0001. (D): Raji NK cell cytotoxicity assay of XLP1 patient BM CD34+ cells transduced with XLP-SMART LVs. BM CD34+ cells from a HD and an XLP1 patient were transduced with XLP-SMART LVs and differentiated into CD56+ NK cells using the StemSpan NK Cell Generation Kit. On day 28 of differentiation, CD56+ NK cells were enriched using magnetic bead isolation and serially diluted with target cells at various effector to target (Raji) ratios: 2.5:1, 1.25:1, 1:1.6, 1:3.2, 1:6.8, and 1:12.8. After 18 h of incubation, 7AAD+ CFSE+ tumor cells were counted via FACS to assess NK cytotoxicity and normalized to target only control wells. Due to insufficient patient cells, data are represented as single replicates from one experiment. Statistical significance was analyzed using a two-way ANOVA followed by multiple paired comparisons for normally distributed data (Tukey test). Compared with XLP1 patient samples, EFS was deemed not significant; E3M was deemed significant with a *p* value < 0.0001; and E3GP was deemed significant with a *p* value < 0.0001.

To measure levels of rescued SAP expression in XLP1 patient cells, we compared SAP expression after transduction by XLP1-SMART LVs to wild-type SAP expression from healthy donor CD8+ T cells via flow cytometry (Figure 5A). In brief, transduced XLP1 patient CD8+ T cells were fixed, permeabilized, and stained for SAP protein using an anti-SAP monoclonal antibody to assess the rescue of SAP protein expression via flow cytometry. At a VCN of 1, XLP1-SMART LVs restored SAP expression in XLP1 patient CD8+ T cells to healthy donor levels (Figure 5A).

It has also been demonstrated that patients with XLP1 are resistant to apoptosis-mediated T cell receptor (TCR) restimulation, as SAP expression is required for TCR-induced apoptosis.^38^ As such, XLP1 patient peripheral blood CD8+ T cells were transduced with XLP1-SMART LVs at equivalent VCNs to assess functional restoration using a restimulation-induced cell death (RICD) assay.^39^^,^^40^ Fourteen days post-transduction, transduced patient CD8+ T cells were quantified for recovery of RICD by flow cytometry. Transduced XLP1 patient T cells with the EFS-SAP, E3M-SAP, and E3GP-SAP vectors restored T cell RICD activity to healthy donor levels at an equal VCN of 1 (Figure 5B).

Healthy donor human CD8+ cells were also transfected using the CRISPR-Cas9 system to knock out the *SH2D1A* gene. Knockout T cells were transduced with XLP1-SMART LVs to assess their RICD activity. The E3M-20R-5RL-SAP LV at a VCN of 2 restored RICD activity to healthy donor levels. Conversely, EFS-SAP and E3GP-20R-5RL-SAP LV transduced *SH2D1A* knockout T cells did not restore T cell RICD activity to healthy donor levels at an equivalent VCN of 2 (Figure S6).

Transduced XLP1 patient BM CD34+ cells were also differentiated into NK cells and tested for functional recovery of NK cell cytotoxicity in a tumor killing assay with NK-sensitive target cells that express SLAM family receptors (Raji) and those that do not (K562).^41^^,^^42^ As expected, MHC-independent killing was observed in K562 cells, with equal levels of killing of K562 by healthy donor and non-transduced/transduced XLP1 patient cells (VCN: EFS = 6, E3M = 0.9, and E3GP = 1.2) (Figure 5C). In a parallel assay with SLAM-expressing Raji cells, E3M-20R-5RL-SAP-transduced XLP1 patient BM CD34+ cells restored NK cell cytotoxicity to near healthy donor levels (VCN: EFS = 1, E3M = 1.7, E3GP = 2.52) (Figure 5D). Conversely, E3GP-20R-5RL-SAP- and EFS-SAP-transduced XLP1 patient BM CD34+ cells did not fully restore NK cell cytotoxicity to healthy donor levels (Figure 5D).

## Discussion

Autologous HSC gene therapy could provide a safe and effective curative treatment for XLP1 if methods can achieve precise, regulated SAP expression in the correct lineages, at therapeutic levels, at the right developmental stage. There are also far fewer risks with autologous gene therapy that hamper allogeneic hematopoietic stem cell transplantation (HSCT), such as graft versus host disease and graft rejection. For the treatment of XLP1, a gene therapy candidate that can restore appropriate *SH2D1A* gene expression after an autologous HSCT in the appropriate hematopoietic cell lineages is required since SAP expression is tightly regulated in T, NK, and NKT cells. There are various gene therapy efforts underway to target XLP1 including gene editing and viral vector-mediated approaches. Recently, a group from the University College London has attempted to combat XLP1 through gene editing, specifically through the use of TALEN, CRISPR-Cas9, and CRISPR-12a nucleases.^39^ The benefit of gene editing for XLP1 is the ability to use endogenous regulatory elements of the *SH2D1A* gene to ensure the tight control necessary for appropriate SAP expression. However, the delivery of these gene-editing reagents utilizes an adeno-associated virus serotype 6-based vector, which can cause issues with immunogenicity and toxicity in treated patients.^43^^,^^44^ Furthermore, the utilization of nucleases and the induction of double-stranded breaks (DSBs) is a safety-profile concern, specifically due to off-target effects, chromosomal translocation, and chromothripsis.^45^^,^^46^ Even newer nuclease-based technologies that overcome these safety concerns such as base-editing and prime editing have disadvantages.^47^ Base-editing is restricted to single-nucleotide changes, so diseases such as XLP1 with multiple mutations are ineligible for this treatment. Prime editing, although capable of inserting or deleting larger fragments of DNA without the need for DSBs, lacks the repair efficiency required to provide a therapeutic advantage. Consequently, the utilization of an LV-based therapy for XLP1 is a promising alternative that may offer a stronger safety profile, so long as the designed LV maintains the tight regulation of *SH2D1A*.

The use of LV gene therapy to treat XLP1 has been attempted previously.^11^ However, this method utilized a ubiquitously active elongation factor 1 α short promoter to drive expression of the SAP protein (EFS-SAP). One concern with this approach lies in the non-physiological expression of SAP in all hematopoietic cell populations. Since SAP expression is tightly regulated within T, NK, and NKT cells, gene expression in off-target cell populations such as myeloid and B cells may pose safety concerns, including improper signaling of factors associated with autoimmune diseases and an elevated apoptotic response to DNA damage.^23^^,^^48^ Previous researchers have demonstrated significant skewing induced by the EFS-SAP vector of hematopoietic potential in the bone marrow compartment via a CFU assay.^8^^,^^11^ As a result, we rationalized that an XLP1 LV therapy requires the incorporation of endogenous enhancer elements to regulate the expression of the *SH2D1A* transgene.

We implemented a bioinformatics-guided approach to develop highly regulated LVs driven by endogenous regulatory elements of the *SH2D1A* gene. Analysis of the topologically associated domain of *SH2D1A* revealed three genomic elements responsible for the physiological expression pattern of the *SH2D1A* gene. Element 5R, located 3 kb directly downstream of the *SH2D1A* promoter, was shown to be a T-, NK-, and NKT-specific enhancer, lacking activity in B cell and myeloid cell lineages. Chromatin immunoprecipitation sequencing (ChIP-seq) data from ENCODE revealed various lymphoid-associated transcription factor binding sites such as MEIS2, TAL1, and SPI1 within element 5R.^28^ These transcription factors are known to regulate expression of NK cell-activating ligands, T cell decision fate, and homeostasis of lymphoid cells.^49^^,^^50^ Element 3, located 125 kb upstream of the *SH2D1A* promoter, presented as a T-, NK-, and NKT-specific enhancer, lacking activity in B cell and myeloid cell lineages. ChIP-seq transcription factor analysis revealed various lymphoid-associated transcription factor binding sites within element 3, such as FOXA1, RUNX3, GATA2, and JUND. These transcription factors are important in T cell development, expression of T and NK cytotoxic lymphocytes, proliferation and maintenance of HSPCs, and modulation of expression of T cell differentiation and activation.^51^ Finally, element 20, located within intron 1 of the *SH2D1A* gene, presented as an NK- and NKT-specific enhancer lacking activity in T cell, B cell, and myeloid cell lineages. ChIP-seq transcription factor analysis revealed various lymphoid-associated transcription factor binding sites within element 20, such as SPI1, GATA2, and BRD4. These transcription factors are known to regulate lymphocyte homeostasis and HSPC differentiation, proliferation, and maintenance.^49^^,^^51^^,^^52^ Taken together, these three enhancers can recapitulate the expression of SAP in all physiologically expressed lineages.

The therapeutic potential of an LV is greatly influenced by both gene transfer and expression. Enhancing gene transfer not only increases the percentage of transduced cells, but also results in more integrated LV copies within each cell, amplifying the overall cellular expression. Conversely, boosting expression directly results in a higher production of the therapeutic protein for every integrated copy. Previous studies have also demonstrated a negative correlation between titer/gene transfer and proviral length.^26^ To address this, we made systematic deletions in each of the genomic elements to reduce the proviral length of our vector. As a result, we observed a 5- to 10-fold enhancement in titer and gene transfer.^26^^,^^27^ Enhancer E3M was further reduced in size from 1.9 to 1 kb to generate the E3GP enhancer. These reductions in enhancer element lengths increased titer by 2-fold and gene transfer by 1.5-fold. Such improvements in titer and gene transfer are of significant value for the clinical application of gene therapy because they substantially decrease the cost of vector production and reduce the volume of LV production lots needed for each patient’s treatment. With these modifications, there is a potential concern about the regulation and expression being compromised. However, even after these adjustments, over 90% of the intended MFI was maintained, and any unintended activity remained minimal. Further modifications to refine the enhancer elements and retain vector expression can be made.

We next strung multiple enhancers together within a single LV to measure the additive effects on mCitrine expression in on-target and off-target cells. While enhancer 3 demonstrated minimal off-target expression in B-LCLs and monocytes, when combined with enhancer 5RL alone or 5RL-20R, off-target expression was abrogated, most likely a result of repressor elements within the 5R enhancer region. This result was further confirmed in our *in vivo* studies, in which the XLP1-SMART LVs contained no significant off-target expression when compared with the promoter-only control in both the bone marrow and peripheral blood compartments. The effects of this decrease in off-target expression warrants further studies in SAP-deficient mouse models.

Using reporter gene experiments in transduced human CB CD34+ cells that were then transplanted into NSG-Tg(Hu-IL15) neonates, we observed that the XLP1-SMART LVs were predominantly expressed in mature human T, NK, and NKT lineages. Importantly, there was no detectable expression in the B and myeloid cell lineages. Furthermore, the E3GP-20R-5RL XLP1-SMART LV exhibited lower levels of mCitrine expression in T, NK, and NKT cells compared with the E3M-20R-5RL XLP1-SMART LV.

We next assessed if the XLP1-SMART LVs will affect hematopoietic skewing. While the E3M-20R-5RL-SAP XLP-SMART LV demonstrated no lineage skewing at VCNs of 1 and 1.7, the EFS-SAP LVs illustrated significant HSC skewing into the myeloid lineage, further indicating the potential adverse effects of ectopic SAP expression within hematopoietic cells. The lineage skewing demonstrated in the CFU assay may be attributed to the increased SAP expression within off-target lineages. The skewing into the myeloid lineage of the E3GP-20R-5RL XLP-SMART LV may be attributed to the differences in enhancer sequences of enhancer 3. Alternatively, the lack of skewing in the E3M-20R-5RL-SAP XLP-SMART LV may be due to VCN differences between that sample and the EFS and E3GP counterparts (EFS = 3, E3M = 1.7, E3GP = 2.52). Further studies are necessary to determine the cause of this skewing and its effects on the XLP1 phenotype.

The dysregulated immune response seen in XLP1 patients can be attributed to their reductions in T cell function and NK cell cytotoxicity. We began by assessing the XLP-SMART LVs efficacy in an alternative disease model: SH2D1A −/− Jurkat cell lines. Our dose response, ranging from a VCN of 1 to 5, demonstrated that at VCN of 3 and 5, the E3M and E3GP vectors reached ∼30% of wild-type SH2D1A protein levels. Future studies are required to determine if this decrease in SH2D1A protein expression will affect function and treatment outcomes. That said, it is possible that wild-type levels are not necessary to alleviate the XLP1 phenotype, as evidenced by the fact that female carriers of XLP are healthy.^53^

We then looked to assess the ability of XLP1 patient cells transduced with XLP-SMART LVs to restore T cell and NK cell function to healthy donor levels. E3M-20R-5RL-SAP (E3M)- and EFS-SAP-transduced XLP1 patient CD8+ T cells restored RICD activity to HD levels at an average VCN of 1; in contrast, studies conducted in healthy donor samples—in which the *SH2D1A* was knocked out with CRISPR-Cas9 technology—demonstrated larger discrepancies of RICD restoration between the EFS, E3M, and E3GP vectors at equal VCN (e.g., EFS and E3GP vectors were not capable of restoring RICD levels to healthy donor levels at a VCN of 2). While these variations may be due to donor variability, further studies in XLP1 patient T cells or in a viable XLP1 *in vivo* model are necessary to confirm the E3M or EFS vector’s ability to restore T cell RICD function.

When assessing restoration of NK cytotoxic activity in XLP1 patient cells, the XLP1-SMART LVs demonstrated greater cytolytic activity than the EFS-SAP vector. This phenotype is likely due to the increased SAP expression in NK cells with the E3M/GP, E5RL, and E20R enhancers. The reduction of NK expression in the E3GP sample compared with E3M may be due to core transcription factor binding sites and epigenetic modifications that were removed in the interest of decreasing vector size for an increased titer and gene transfer. The lack of EFS-SAP vector NK cytolytic activity is potentially a result of reduced SAP expression within NK cells due to a lower VCN seen in the EFS-transduced populations compared with the E3M and E3GP counterparts (VCNs were EFS = 1.1, E3M = 1.7, E3GP = 2.52). The limited access to more XLP1 patient CD34+ cells and PBMCs prevented repeated studies at equal VCNs. Future *in vivo* studies utilizing the C57BL/6 *SH2D1A*−/− mouse model will help elucidate the ability for XLP-SMART LVs to restore the humoral defects and the NKT developmental block seen in XLP1 patients.

While future studies are needed to assess the safety and efficacy of the XLP-SMART LVs (e.g., *in vitro* immortalization assay and C57BL/6 *SH2D1A*−/− mouse studies), the outcomes of this study have elucidated a streamlined approach for the identification and incorporation of key elements into a vector cassette that can properly regulate a target gene (SMART LVs). The rational generation of regulated LVs offers a novel, effective, and efficient approach for enhanced expression and regulation of transgenes required to correct various inborn errors of immunity, as evidenced by these highly specific XLP-SMART LVs for the treatment of XLP1. In conjunction with these studies, the regulated expression by enhancers 3, 20, and 5R within the T, NK, and NKT lineages may provide a useful approach toward a lentiviral gene therapy for hemophagocytic lymphohistiocytosis (HLH) disorders, such as perforin deficiency, which share similar expression profiles and regulation to that of XLP1. Ultimately by iterative process, we developed two candidate vectors that express SAP protein in a lineage- and stage-specific manner at levels similar to the endogenous *SH2D1A* gene.

## Materials and methods

### Elucidation of putative enhancer elements

Genomic regions containing putative regulatory elements of the *SH2D1A* gene were compiled using data from ENCODE, Ensembl, FANTOM, and VISTA. Functional boundaries of the putative enhancer elements were defined using lineage-specific DNase I accessibility, transcription factor binding, epigenetic histone modification, and vertebrate sequence conservation. Primers were then designed to amplify these putative enhancer regions from human genomic DNA for downstream cloning into plasmid lentiviral transgene cassettes.

### Vector packaging and titration

Lentiviruses were packaged by transient transfection of PKR−/− 293T cells with fixed amounts of HIV Gag/Pol, Rev, and VSV-G envelope expression plasmids and equimolar amounts of transfer plasmid using TransIT-293 (Mirus Bio, Madison, WI) as described in Cooper et al.^30^^,^^54^ Viral supernatants were then directly used for titer determination or concentrated by tangential flow filtration. To titer the lentivirus, 1 × 10^5^ HT-29 cells per sample were plated in 2 mL of culture medium in 6-well plates (no. 3516; Corning, Corning, NY). Twenty-four hours after plating, cells were transduced with a 1:10 dilution of viral supernatant in 1 mL of culture medium. Twenty-four hours after transduction, culture medium was refreshed on all wells. Seventy-two hours after transduction, cells were harvested to determine VCNs by ddPCR. Vector titer (TU/mL) was calculated as TU = VCN × (cell count at day of transduction) × virus dilution. Cell counts were measured with a Vi-CELL XR automated cell counter (Beckman Coulter, Brea, CA).

### LV transduction

Primary human T, NK, and NKT cells (5 × 10^4^ per sample) isolated from PBMCs were plated on retronectin (20 μg/mL) (Takara Bio, Kusatsu, Shiga, Japan)-coated plates with XVIVO15 medium (Lonza Biosciences, Basel, Switzerland) supplemented with 1 mg/mL poloxamer synperonic F108 (Kolliphor P338; BASF Pharma, Ludwigshafen, Germany) for 24 h during lentiviral transduction. Cell counts were measured with a Vi-CELL XR automated cell counter. For transduction of CD34+ cells, cells were prestimulated with 50 ng/mL each of human stem cell factor (hSCF), human thrombopoietin (hTPO), and human FMS-like tyrosine kinase 3 ligand (hFlt3-L) (Peprotech, Rocky Hill, NJ) for 24 h before lentiviral transduction. During LV transduction of CD34+ cells, 10 μM prostaglandin E2 (PGE2) was also added for 24 h. LV supernatant (raw or concentrated) was added to the culture medium for 24 h at various concentrations to achieve the necessary VCN.

### ddPCR for VCN and titer quantification

Genomic DNA from transduced cells was extracted using a PureLink Genomic DNA Mini Kit (K182002; Invitrogen, Waltham, MA). VCN was calculated by using the vector HIV-1 PSI gene primers (oPAF-PSI, oPAR-PSI) and probes (oPAP-PSI) and an endogenous human diploid gene control (SCD4; Human Syndecan 4) primers (oPAF-SDC4 and oPAR-SDC4) and probe (oPAP-SDC4) as a reference (Table S4). Reaction mixtures of 22 μL volume, comprising 1× ddPCR Master Mix (no. 1863010; Bio-Rad, Hercules, CA), 400 nmol/L primers, and 100 nmol/L probe for each set, 40 U DraI (R0129S; New England Biolabs, Ipswich, MA) and 30–100 g of the gDNA were prepared and incubated at 37°C for 1 h. Droplet generation was performed as described in Hindson et al. with 20 μL of each reaction mixture.^55^ The droplet emulsion was then transferred with a multichannel pipette to a 96-well twin.tec real-time PCR Plate (Eppendorf, Hamburg, Germany), heat sealed with foil, and amplified in a conventional thermal cycler (T100 Thermal Cycler; Bio-Rad). Thermal cycling conditions consisted of 95°C for 10 min (1 cycle), 94°C for 30 s, and 60°C for 1 min (55 cycles), 98°C for 10 min (1 cycle), and 12°C hold. After PCR, the 96-well plate was transferred to a droplet reader (Bio-Rad). Acquisition and analysis of the ddPCR data was performed with the QuantaSoft software (Bio-Rad), provided with the droplet reader. Vector titer (TU/mL) was calculated as TU = VCN × (cell count at day of transduction) × virus dilution.

### NSG-Tg(hu-IL15) xenografts

Transduced human CB CD34+ cells were washed and incubated with 1 μg/100 μL of OKT3 (Tonbo Biosciences, San Diego, CA) for 30 min at 4°C to prevent contaminating T cell-derived graft-versus-host disease. Immediately before transplant, 1- to 3-day-old neonatal NSG-Tg(hu-IL-15) mice (NOD.Cg-*Prkdc*^*scid*^ *Il2rg*^*tm1Wjl*^ Tg(IL15)1Sz/SzJ, strain no. 030890; The Jackson Laboratory, Bar Harbor, ME) were irradiated at a dose of 150 rads with a cesium-137 source. Each mouse was injected intrahepatically with 1 × 10^5^ to 5 × 10^5^ cells. At 16 weeks post-transplant, mouse bone marrow, spleen, thymus, and peripheral blood were harvested into single-cell suspensions for downstream flow cytometry analysis. The mice were maintained at UCLA under an approved protocol by the UCLA Animal Research Committee under the Division of Laboratory Medicine.

### Plasmid generation

All LVs were cloned into an empty pCCL backbone.^56^ Fragments of transcriptional regulatory elements were synthesized as gBlocks (Integrated DNA Technologies, Coralville, IA) or amplified from genomic DNA by polymerase chain reaction with compatible ends to be cloned using an NEBuilder HiFi DNA Assembly Kit (New England Biolabs). Enhancer elements were inserted upstream of a 600 bp *SH2D1A* promoter to drive expression of the SAP or an mCit reporter cassette. Furthermore, each vector contains the WPRE in replacement of the endogenous *SH2D1A* 3′ UTR.

### Cell culture

CD34+ cells were cultured in X-VIVO15 medium (Lonza Biosciences) supplemented with 1× penicillin-streptomycin-glutamine (P/S/G), with 50 ng/mL each of hSCF, hTPO, and hFlt3-L for 24 h before LV transduction. CD3+ T cells were isolated from PBMCs through CD3+-positive magnetic selection (Miltenyi Biotec, Bergisch Gladbach, Germany). Isolated cells were then activated using anti-CD3/CD28 Immunocult (STEMCELL Technologies, Vancouver, Canada) and cultured in X-VIVO15 medium (Lonza Biosciences), 5% human serum, and 100 U/mL of human recombinant IL-2. NKT cells were isolated from PBMCs through iNKT Vα24+-positive magnetic selection (Miltenyi Biotec). NKT cells were cultured in RPMI 1640, 1% P/S, 10% fetal bovine serum (FBS), 1% MEM non-essential amino acids, 10 mM HEPES, 1 mM sodium pyruvate, and 50 μM 2-mercaptoethanol. NKT cells were stimulated at a ratio of 1:1 with autologous PBMCs loaded with 5 ng/mL of α-GalCer (Cayman Chemical, Ann Arbor, MI, cat, no. 158021-47-7) and irradiated at 6,000 rpm. The cocultured cells were subsequently cultured with 10 ng/mL of IL-15 and IL-7. NK cells were enriched from PBMCs using the CD56+-positive magnetic selection (Miltenyi Biotec). Isolated NK cells were cultured in NK MACS medium (Miltenyi Biotech) supplemented with 5% human AB serum, 1% P/S, 10 ng/mL hIL-15, and 500 U/mL of hIL-2 at 5% CO_2_ and 37°C humidified atmosphere.

### ATO generation

ATOs were generated from human mPB stem cells as described previously.^34^^,^^35^ Previously frozen MS5-hDLL4 cells were thawed and resuspended in serum-free ATO culture medium (RB27) composed of RPMI 1640 (Corning, Manassas, VA), 4% B27 supplement (Thermo Fisher Scientific, Grand Island, NY), 30 μM L-ascorbic acid 2-phosphate sesquimagnesium salt hydrate (Sigma-Aldrich, St. Louis, MO) reconstituted in PBS, 1% P/S (GeminiBio products, West Sacramento, CA), and 2% GlutaMAXx (Thermo Fisher Scientific). RB27 lasts 3 weeks at 4°C. MS5-hDLL4 cells and CD34+ mPB cells edited with XLP1-SMART LVs or mock control were combined in Eppendorf tubes at a concentration of 150k MS5-hDLL4 and 5,000 mPB per ATO to make 24 ATOs per group. Cells were centrifuged at 300 × *g* for 5 min at 4°C in a swinging bucket centrifuge. Supernatants were carefully removed, and the cell pellet was resuspended in 5 μL RB27 per ATO and mixed by brief vortexing. ATOs were plated on 0.4 μm Millicell transwell inserts (EMD Millipore, Billerica, MA, cat. no. PICM0RG50) in 6-well plates containing 1 mL complete RB27 (RB27 with the addition of 5 ng/mL rhFLT3L, 2.5 ng/mL rhIL-7, and 5 ng/mL hSCF (Peprotech) per well. Two ATOs were plated per insert. Medium was changed completely every 3–4 days by aspiration from around the cell insert followed by replacement with 1 mL of fresh RB27/cytokines. hSCF is only added for the first week of culture. ATOs are kept in an incubator at 37°C with 5% CO_2_. At weeks 3, 7, and 12, six ATOs (three inserts) per group per time point were harvested by adding FACS buffer (PBS/0.5% bovine serum albumin/2 mM EDTA) to each well and briefly disaggregating the ATO by pipetting, followed by passage through a 50 μm nylon cell strainer. Cells were stained in 96-well plates. At weeks 3 and 7, 200k cells per ATO were stained without fixation to determine mCitrine levels and T cell differentiation kinetics, and the remaining cells (no more than 2 million cells per stained sample) were fixed and permeabilized to assess intracellular SAP expression and T cell differentiation. At week 12, half of the ATO cells were stained without fixation and half were stained with fixation (no more than 2 million cells per stained sample).

### Acquisition of XLP1 patient cells

XLP1 patient cells were acquired after patient and parental informed consent (UCLA IRB no. 10-001399). Collection of BM CD34+ cells after bone marrow aspirate and acquisition of PBMCs from peripheral blood were procured under Institutional Review Board-approved protocol at the David Geffen School of Medicine at UCLA, protocol no. 10-001399. The XLP1 patient harbored a pathogenic missense mutation in exon 1 of the *SH2D1A* gene.

### CFU assay

One hundred, 300, and 900 BM CD34+ HSPCs per replicate were plated in MethoCult (cat. no. 04445; STMECELL Technologies) 24 h after LV transduction. After 14 days of culture at 5% CO_2_, 37°C, and humidified atmosphere, the number of mature colonies were counted and scored under the microscope based on their specific morphology.

### Generation of SH2D1A knockout cell lines

To generate an XLP1 model cell line, Jurkat T cells were modified to knockout *SH2D1A* by electroporation of SpCas9 recombinant protein (QB3 Macrolab; UC Berkeley, Berkeley, CA) complexed to sgRNA (Table S5) (Synthego, Redwood City, CA) and FACS single-cell sorted and cultured in R20 (RPMI 1640 [Gibco, Grand Island, NY]/20% FBS [Gibco]/1× P/S/G [GeminiBio products]). Primers for amplification of the *SH2D1A* locus were used to confirm knockout (oPAF605 [TCCTATGAATGCAATGACACCA] and oPAR340 [TGTGGCAATTTTCAGGAGTTCAC]) by Synthego ICE. Absence of SAP expression was confirmed by western blot analysis. Cells were cultured in R10 at 37°C with 5% CO_2_.

### Western blot

For immunoblots, cells were lysed in RIPA lysis and extraction buffer (cat. no. 89901; Thermo Fisher Scientific) with added HALT protease inhibitor (cat. no. 87786; Thermo Fisher Scientific) at a 1× concentration following the manufacturer’s protocols. Lysate concentrations were determined using the Pierce BCA protein assay (cat. no. 23227; Thermo Fisher Scientific) following the manufacturer’s protocol. Samples were treated for sodium dodecyl sulfate-polyacrylamide gel electrophoresis with NuPAGE LDS Sample Buffer (cat. no. NP0007; Thermo Fisher Scientific) and NuPAGE Sample Reducing Agent (cat. no. NP0009; Thermo Fisher Scientific), each to a 1× concentration. Lysates were diluted to contain equivalent amounts of total protein for immunoblot gel loading, using lysate from the SAP-deficient Jurkat cells to keep the total amount of protein loaded per lane constant to allow for valid loading controls. Wild-type Jurkat lysate was used as a control to indicate the relative expression levels of the *SH2D1A* codon optimized XLP1 LVs. SAP levels were detected using Abnova monoclonal antibody, clone 1C9 (cat. no. H00004068-M01). Protein quantification was assessed through densitometry via the ImageJ software. SAP protein levels were normalized to the actin protein levels after quantification.

### Flow cytometry

Intracellular staining of SAP was performed using the eBioscience Foxp3/Transcription Factor Staining Buffer Set (Invitrogen) using the manufacturer’s protocol. The primary antibody was rat anti-human SAP antibody, PE (cat. no. 12-9787-42; Invitrogen). To discern the various hematopoietic cell lineages, cell populations were gated as follows: NK cell: CD56+, CD16–; NKT: CD3+, CD56+; iNKT: CD3+, TCR Va24+, CD56+; B cell: CD19+, CD3–; T cell: CD3+, CD19–, CD4+/CD8+; monocytes: CD33–, CD16–, CD14+. To discern the various T cell subpopulations and thymocyte populations in ATOs, we used the following monoclonal antibodies for staining: hCD45, hCD56, hCD34, hCD5, hCD7, hTCRab, hCD4, hCD8a, hCD8b, hCD3, hCD45RA, and hCD45RO. To discern the various NK cell subpopulations, we used the following monoclonal antibodies for staining: hCD122, hCD16, hCD117, hCD45, hNKP80, hCD56, hCD34, hCD94, and hCD57. Live/dead dyes used for staining include DAPI and Zombie Fixable Viability Dye (BioLegend, San Diego, CA) for unfixed and fixed cells, respectively. See supplemental figures for more information (Table S6).

### Next-generation sequencing library preparation

Fourteen days after transduction, genomic DNA and mRNA were harvested from the primary T cells, primary NK cells, primary NKT cells, and the B-LCLs. For library preparation, an initial PCR was completed to amplify the barcodes (Table S7) using primers oPAF255 – XLP Barcode Amp for NGS (Table S8) and oPAR119 – XLP Barcode Amp for NGS (Table S8). A second PCR was completed to add Illumina adapters and indexes. Following Illumina barcoding, PCR products were pooled at equal concentrations, purified twice using AMPure XP beads (Beckman Coulter), and then quantified by ddPCR (QX 200; Bio-Rad). The high-throughput sequencing was performed at the UCLA Technology Center for Genomics & Bioinformatics (TCGB) using an Illumina MiSeq instrument 2 × 150 paired-end reads (Illumina, San Diego, CA). For flow cytometry gating strategies, please see Figures S7–S17.

### RICD

RICD was assessed using an established protocol.^40^^,^^57^ LV modified or unmodified T cells were cultured for 10 days, before plating at 5 × 10^4^ cells/well in a 96-well plate in 100 μL medium. Dilutions of OKT3 antibody (Tonbo Biosciences, San Diego, CA) were prepared at 2,000 , 200, and 20 ng/mL, and 100 μL added to the cells to make final concentrations of 1,000, 100, and 10 ng/mL OKT3 in the wells. After 24 h, a final concentration of 1 μg/mL of propidium iodide (PI) was added before running a fixed volume of cell suspension from each well for flow cytometry. The numbers of live cells (PI–) in stimulated controls were compared with unstimulated controls to measure the percent cell loss = [1 – (no. of PI– restimulated cells/no. of PI– untreated cells)] × 100.

### NK differentiation

Untransduced or transduced healthy donor and XLP1 patient BM CD34+ cells were differentiated into CD56+ NK cells over 28 days of culture using the StemSpan NK Cell Generation Kit (cat. no. 09960; STEMCELL Technologies) following the manufacturer’s protocol. BM CD34+ cells (1 × 10^4^) were plated on non-tissue culture-treated plates coated with StemSpan Lymphoid Differentiation Coating Material. Half-volume medium changes were performed using StemSpan Lymphoid Progenitor Expansion Medium until day 14. The cells were then replated at 1 × 10^5^ cells/mL in StemSpan NK Cell Differentiation Medium and cultured for another 14 days. On day 28, the cells were harvested and enriched using CD56+-positive magnetic selection (Miltenyi Biotec). The differentiated NK cells were then assessed for cytotoxic capabilities in further assays.

### NK killing assay

BM CD34+ cells differentiated into CD56+ NK cells were counted and resuspended in NK medium at 5 × 10^4^ cells per 200 μL and added to row B on a 96-well plate. One hundred microliters of NK medium was added to rows C through G. Six serial dilutions were performed of the resuspended NK cells using 100 μL multichannel pipette from rows B to G (from 2.5 × 10^4^ cells per well to 800 cells per well). Target cells were either K562 cells expressing one copy of a GFP reporter cassette or Raji cells labeled with CellTrace CFSE (C34554; Invitrogen) resuspended in R10 (RPMI 1640, 10% FBS, 1% P/S/G) at 1 × 10^4^ cells per 100 μSL. Of the target cells, 100 μL was added to rows A through G to generate the following effector to target ratios: 2.5:1, 1.25:1, 0.625:1, 1:3, 1:6, and 1:12.5. The plate was incubated for 18 h at 5% CO_2_, 37°C. After incubation, GFP+ tumor cells were counted via FACS using a BD FACSCelesta Cell Analyzer (BD Biosciences, Franklin Lakes, NJ) and normalized to target only control wells (rows A and G) to normalize the killing percentage.^41^

### Monocyte differentiation

CB CD34+ HSCs were cultured in X-VIVO15 medium (Lonza Biosciences), 4% FBS (Gibco), 1× P/S/G (GeminiBio products), 50 ng hSCF, 15 ng/mL hTPO, 30 ng/mL hIL3, and 30 ng/mL hFlt3-L (all cytokines: Peprotech) for 9 days. After 9 days of culture, the CB CD34+ cells were differentiated in the following medium for 7 days: STEMSpan II (STEMCELL Technologies), 20% FBS (Gibco), 1× P/S/G (GeminiBio products), 25 ng hSCF, 30 ng/mL M-CSF, 30 ng/mL hIL3, and 30 ng/mL hFlt3-L (all cytokines: Peprotech). On day 16 of culture, cells were confirmed for NK differentiation and purity by flow cytometry using anti-CD14 and anti-CD16 antibodies.

### T cell electroporation to knock out SH2D1A

After isolation from PBMCs, CD3+ primary T cells were counted by hemocytometer via trypan blue exclusion prior to electroporation. Per condition, 1 × 10^6^ cells were centrifuged at 300 × *g* for 10 min at room temperature (RT), resuspended in 20 μL of P3 electroporation buffer (Lonza Biosciences). SpCas9 recombinant protein (100 pmol) (QB3 Macrolab, UC Berkeley) was combined with 120 pmol of each sgRNA to *SH2D1A* (sgRNA4: 5′-GACGCAGTGGCTGTGTATCA-3’; sgRNA7: 5′-AACAGGTTCTTGGAGTGCTG-3′ both from Synthego, Redwood City, CA) for 15 min at RT for RNP complex formation. The cell and RNP mixtures were combined and electroporated using the EH-100 setting on the Amaxa 4D Nucleofector X Unit (Lonza Biosciences). Cells were rested in 16-well electroporation strips (Lonza Biosciences) for 10 min at RT and then recovered with 480 μL of T cell medium. Twenty-four hours after electroporation, *SH2D1A* knockout T cells were transduced with XLP-SMART LVs and used for an RICD assay 10 days post-electroporation.

### Statistical analysis

All data are reported as mean ± SD unless otherwise stated. All statistical analyses were carried out using GraphPad Prism version 10.0.0 (GraphPad Software, San Diego, CA). The statistical significance between two averages was established using unpaired t tests. When the statistical significance between three or more averages was evaluated, a one-way ANOVA was applied, followed by multiple paired comparisons for normally distributed data (Tukey’s test). When the statistical significance between two or more categorical variables was evaluated, a two-way ANOVA was applied, followed by multiple paired comparisons for normally distributed data (Tukey’s test). Linear regression analyses were used to determine the correlation between titer and proviral size. All statistical tests were two-tailed and a *p* value of <0.05 was deemed significant (ns, non-significant; ∗*p* < 0.05, ∗∗*p* < 0.01, ∗∗∗*p* < 0.001, ∗∗∗∗*p* < 0.0001). Details of statistical tests used, including all *p* values, are indicated in the relevant figure legend.

## Data and code availability

The data generated in this study are available within the article and its supplemental information. Any additional materials and protocols will be distributed to researchers for non-commercial and academic purposes.

## Acknowledgments

Dr. Lili Yang (UCLA) and Charlie Yan-Ruide Li (UCLA) assisted with NKT expansion and activation. This work was funded through the use of unrestricted funds. J.G. is supported by the UCLA Tumor Cell Biology Training Program (NCI T32CA009056) and the UCLA-Caltech Medical Scientist Training Program (NIGMS T32GM008042). The Flow Cytometry Core of the UCLA Eli and Edythe Broad Center of Regenerative Medicine and Stem Cell Research, the Imaging Core of the UCLA Eli and Edythe Broad Center of Regenerative Medicine and Stem Cell Research, and the Virology Core of the UCLA Center for AIDS Research (CFAR) were used to support studies.

## Author contributions

P.G.A. conceived of the project, performed bioinformatic analyses to identify putative enhancer elements, designed the vectors, led the *in vitro* and *in vivo* studies of vector activity, and wrote the manuscript. R.P.H. participated in design and analysis of the SMART LV and edited the manuscript. J.G. performed the ATO experiments and edited the manuscript. L.L., C.J., K.T., J.R., C.T., J.Q., and G.E.M. performed laboratory work in support of the studies. K.B. provided the XLP1 patient cells after acquiring patient consent. D.B., X.W., and R.Z. performed murine transplant work. J.G. and G.M.C. designed and performed studies in the Artificial Thymic Organoid system. F.Y.M. performed bioinformatic analyses. D.B.K. oversaw the studies and edited the manuscript.

## Declaration of interests

The authors declare no competing interests.

## Supplemental information

Data S1. Tables S1–S8

Document S2. Article plus supplemental information
