# Supplementary material for: Lentiviral vectors for precise expression to treat X-linked lymphoproliferative disease
Source: Mol Ther Methods Clin Dev. 2024 Aug 20;32(4):101323. doi: 10.1016/j.omtm.2024.101323 (PMC11415656; doi:10.1016/j.omtm.2024.101323)
Supplement: Document S1. Figures S1–S17 [file mmc1.pdf]

**OMTM, Volume *32***

## Supplemental information

# Lentiviral vectors for precise expression to treat X-linked lymphoproliferative disease

**Paul G. Ayoub, Julia Gensheimer, Lindsay Lathrop, Colin Juett, Jason Quintos, Kevin Tam, Jack Reid, Feiyang Ma, Curtis Tam, Grace E. McAuley, Devin Brown, Xiaomeng Wu, Ruixue Zhang, Kathryn Bradford, Roger P. Hollis, Gay M. Crooks, and Donald B. Kohn**

**Figure S1:**

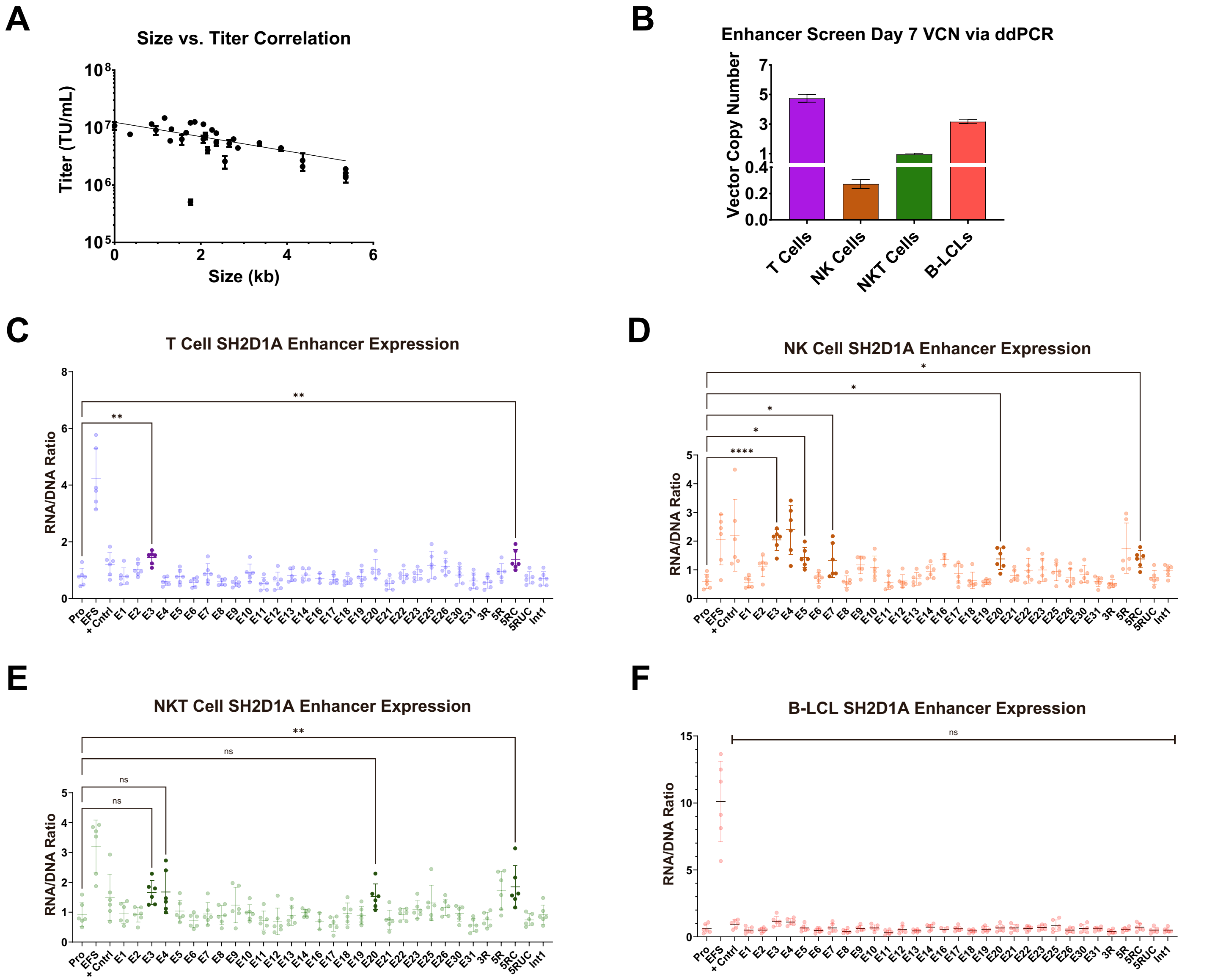


**A: Effect of Lentivirus Proviral Size on Titer.** Putative enhancers were cloned into the plasmid backbone of a therapeutic lentiviral vector (pCCL-c-MNDU3-X [Addgene Plasmid #81071]), packaged, titered head-to-head, and the quantity of infectious particles was plotted as a function of proviral length (bp). Each point in the plot represents an average of three individual 10-cm plates of virus titered on HT-29 cells. Proviral length is defined as sequence length from the beginning of the 5′ long terminal repeat (LTR) U3 through the end of the 3′ LTR U5. n = 3 per arm. Linear regression analyses were used to determine the correlation between titer and proviral size ($R^2$=0.51).

**B: Enhancer Screen Bulk Vector Copy Number.** We transduced primary T, NKT, and NK cells with a pool of raw viral supernatant containing $5x10^4$ TU/mL of the 34 candidate XLP1-SMART-LVs and the EFS-mCitrine vector. B-LCLs were transduced to measure off-target expression. 14 days post-transduction, cells were harvested for their gDNA fraction to measure vector copy number using digital droplet PCR. Data are represented as mean ± SD of biological triplicates from one experiment.

**C-F: Relative SH2D1A Enhancer Activity in T, NK, NKT, and B-LCL Cells.** We transduced primary T, NKT, and NK cells with a pool of raw viral supernatant containing each of the 34 candidate XLP1-SMART-LVs, in duplicate, and the EFS-mCit vector. Each candidate LV was cloned with two unique barcodes. B-LCLs were transduced to measure off-target expression. 14 days post-transduction, cells were harvested for their gDNA and RNA fractions to measure barcode expression via next-generation sequencing. The RNA barcode counts identify active enhancers within each lineage whereas each genomic barcode count is used normalize each barcode in the transcript to the genome. The number of RNA barcode reads, normalized to frequency of gDNA barcodes within each cell type, determined the relative expression of each element. Data are represented as mean ± SD of biological triplicates from two experiments. We analyzed statistical significance using a one-way ANOVA followed by multiple paired comparisons for normally distributed data (Tukey test). All statistical tests were two-tailed and a p value of < 0.05 was deemed significant (ns non-significant, *P < 0.05, **P < 0.01, ***P < 0.001, ****P < 0.0001).

**Figure S2:**

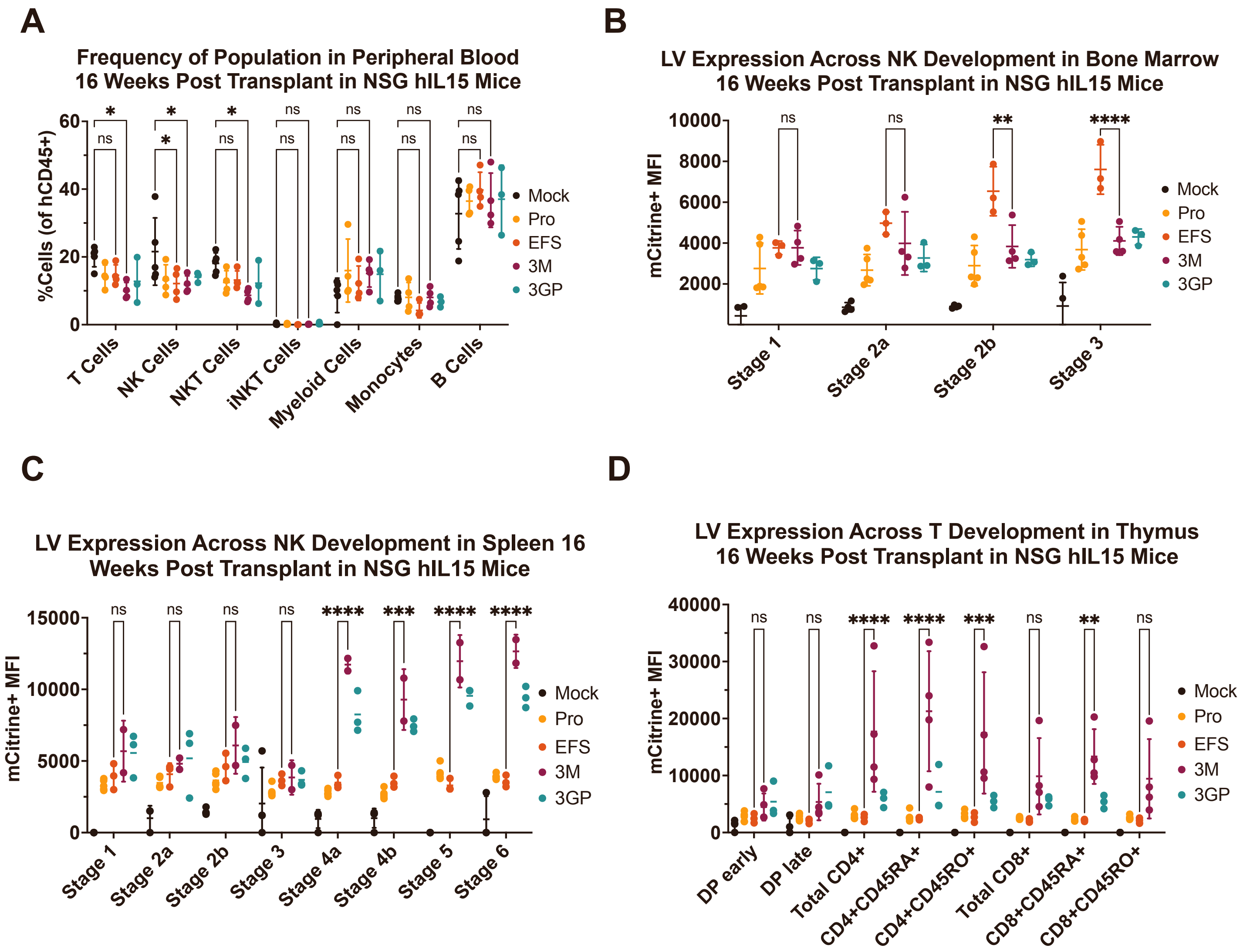


**A: Frequency of Hematopoietic Populations in Peripheral Blood 16 Weeks Post Transplant in hIL15 NSG Mice.** Mice were bled at 16 weeks to analyze peripheral blood for XLP1-SMART-LV expression. Lysed red blood cells were stained for various lineages within the hCD45+ gate (T Cells: hCD33- hCD19- hCD3+; NK Cells: hCD33- hCD3- hCD19- hCD56+; NKT Cells: hCD33- hCD19- hCD3+ hCD56+; iNKT cells: hCD33- hCD19- hCD3+ hCD56+ hVα24+; Myeloid Cells: hCD33+; Monocytes: hCD33+, CD14+, CD16-; B Cells: hCD33- hCD19+ hCD3-). Frequency of each population is plotted as a percentage of total hCD45+ cells using flow cytometry. Data are represented as mean ± SD of biological triplicates from one experiment. We analyzed statistical significance using a two-way ANOVA followed by multiple paired comparisons for normally distributed data (Tukey test). All statistical tests were two-tailed and a p value of < 0.05 was deemed significant (ns non-significant, *P < 0.05, **P < 0.01, ***P < 0.001, ****P < 0.0001).

**B: XLP1-SMART-LV Expression across NK cell Development in Bone Marrow 16 Weeks Post Transplant in hIL15 NSG Mice.** Whole bone marrow (BM) was taken from each mouse at time of euthanasia and processed into a single cell suspension. Single cells were stained for various stages of NK cell differentiation within the hCD45+hCD33- gate (Stage 1: hCD34+; Stage 2a: hCD34+ hCD117+ hCD122-; Stage 2b: hCD34+ hCD117+ hCD122+; Stage 3: hCD34- hCD117+ hCD122+ hCD56-). Each LV's relative expression was measured via mCitrine+ MFI using flow cytometry. Each enhancer was compared to basal SH2D1A promoter expression (Pro) and the preclinical vector (EFS). Data are represented as mean ± SD of biological triplicates from one experiment. We analyzed statistical significance using a two-way ANOVA followed by multiple paired comparisons for normally distributed data (Tukey test). All statistical tests were two-tailed and a p value of < 0.05 was deemed significant (ns non-significant, *P < 0.05, **P < 0.01, ***P < 0.001, ****P < 0.0001).

**C: XLP1-SMART-LV Expression across NK cell Development in Spleen 16 Weeks Post Transplant in hIL15 NSG Mice.** The spleen was taken from each mouse at time of euthanasia, processed into a single cell suspension, and lysed. Lysed splenic single cells were stained for various stages of NK cell differentiation within the hCD45+hCD33- gate (Stage 1: hCD34+; Stage 2a: hCD34+ hCD117+ hCD122-; Stage 2b: hCD34+ hCD117+ hCD122+; Stage 3: hCD34- hCD117+ hCD122+ hCD56-; Stage 4a: hCD34- hCD117+ hCD122+ hCD56+ hCD94+; Stage 4b: hCD34- hCD117- hCD122+ hCD56+ hCD94+ hNKp80+; Stage 5: hCD34- hCD117- hCD122+ hCD56+ hCD94+ hNKp80+ hCD16+; and Stage 6: hCD34- hCD117- CD122+ hCD56+ hCD94+ hNKp80+ hCD16+ hCD57). Each LV's relative expression was measured via mCitrine+ MFI using flow cytometry. Each enhancer was compared to basal SH2D1A promoter expression (Pro) and the preclinical vector (EFS). Data are represented as mean ± SD of biological triplicates from one experiment. We analyzed statistical significance using a two-way ANOVA followed by multiple paired comparisons for normally distributed data (Tukey test). All statistical tests were two-tailed and a p value of < 0.05 was deemed significant (ns non-significant, *P < 0.05, **P < 0.01, ***P < 0.001, ****P < 0.0001).

**D: XLP1-SMART-LV Expression across T cell Development in Thymus 16 Weeks Post Transplant in hIL15 NSG Mice.** The thymus was taken from each mouse at time of euthanasia and processed into a single cell suspension. The single cell suspension was stained for mature stages of T cell differentiation within the hCD45+ hCD34- hCD14- hCD19- hCD56- hCD5+ hCD7+ TCRab+ CD3+ gate (DP early: TCRab-CD3-CD8+CD4+; DP late: hCD4+ hCD8+; total CD4+: hCD4+ hCD8-; CD4+CD45RA+: hCD4+ hCD8- hCD45RA+ hCD45RO-; CD4+CD45RO+: hCD4+ hCD8- hCD45RA- hCD45RO+; total CD8+: hCD4- hCD8+; CD8+CD45RA+: hCD4- hCD8+ hCD45RA+ hCD45RO-; CD8+CD45RO+: hCD4- hCD8+ hCD45RA- hCD45RO+). Each LV's relative expression was measured via mCitrine+ MFI using flow cytometry. Each enhancer was compared to basal SH2D1A promoter expression (Pro) and the preclinical vector (EFS), both

harboring an mCitrine reporter cassette. Data are represented as mean ± SD of biological triplicates from one experiment. We analyzed statistical significance using a two-way ANOVA followed by multiple paired comparisons for normally distributed data (Tukey test). All statistical tests were two-tailed and a p value of $< 0.05$ was deemed significant (ns non-significant, *$P < 0.05$, **$P < 0.01$, ***$P < 0.001$, ****$P < 0.0001$.).

**Figure S3:**

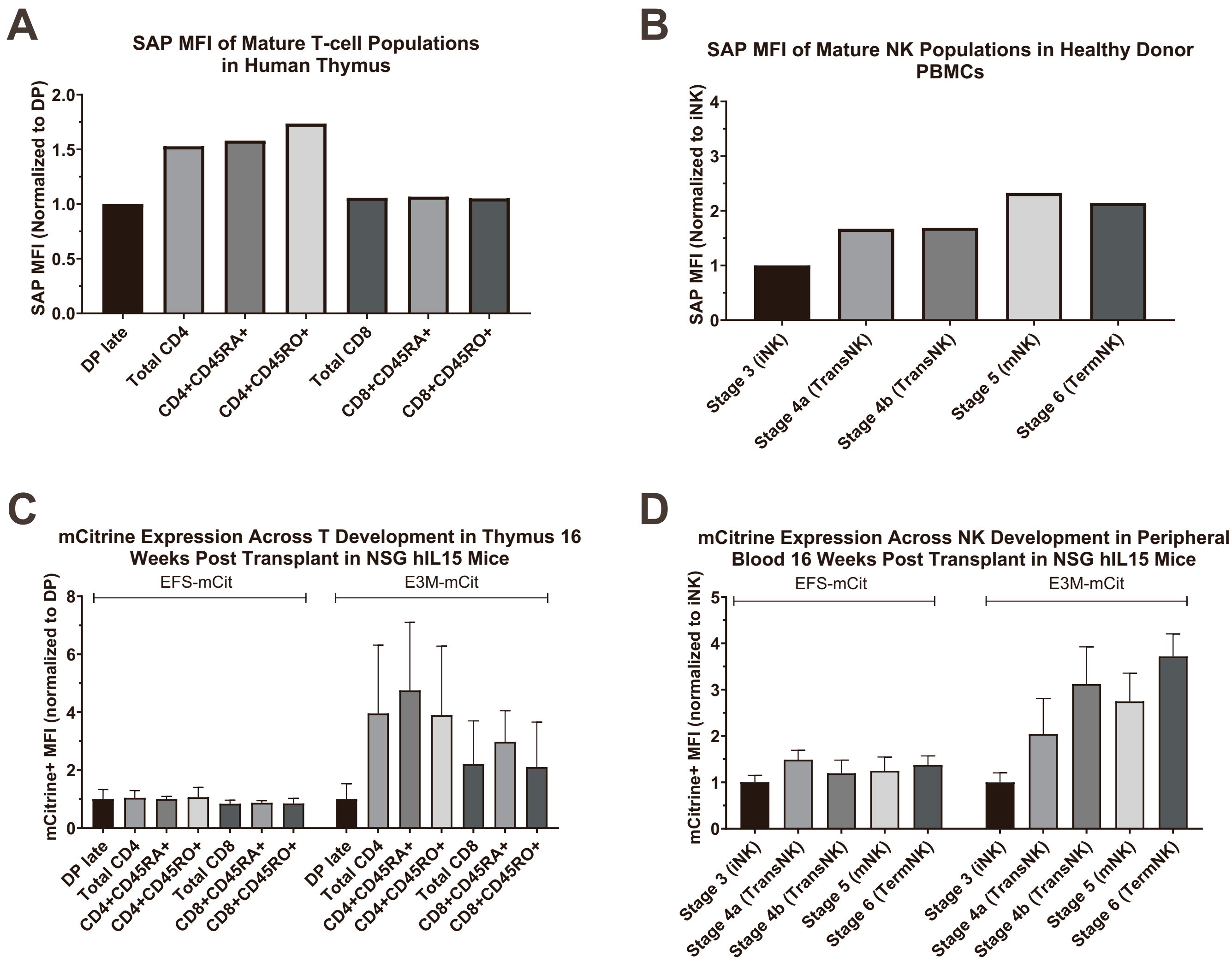


**A: SAP Mean Fluorescence Intensity of T cell Populations in Human Thymus:** Healthy donor human thymic tissue was processed into a single cell suspension. The single cell suspension was stained for mature stages of T cell differentiation within the hCD45+ hCD34- hCD14- hCD19- hCD56- hCD5+ hCD7+ TCRab+ CD3+ gate (DP: hCD4+ hCD8+; total CD4+: hCD4+ hCD8-; CD4+CD45RA+: hCD4+ hCD8- hCD45RA+ hCD45RO-; CD4+CD45RO+: hCD4+ hCD8- hCD45RA- hCD45RO+; total CD8+: hCD4- hCD8+; CD8+CD45RA+: hCD4- hCD8+ hCD45RA+ hCD45RO-; CD8+CD45RO+: hCD4- hCD8+ hCD45RA- hCD45RO+). Stained cells were then fixed, permeabilized and stained for SAP using an anti-SAP monoclonal antibody. SAP expression was determined via total SAP MFI within each target population using flow cytometry.

**B: SAP Mean Fluorescence Intensity of Mature NK cell Populations in Three Healthy Donor PBMC Samples:** CD56+ NK cells from (n=3) healthy donors (HD) were isolated from PBMCs. Cells were stained with monoclonal antibodies for various stages of NK development (Stage 3: hCD34- hCD117+ hCD122+ hCD56-; Stage 4a: hCD34- hCD117+ hCD122+ hCD56+ hCD94+; Stage 4b: hCD34- hCD117- hCD122+ hCD56+ hCD94+ hNKp80+; Stage 5: hCD34- hCD117- hCD122+ hCD56+ hCD94+ hNKp80+ hCD16+; and Stage 6: hCD34- hCD117- CD122+ hCD56+ hCD94+ hNKp80+ hCD16+ hCD57). Stained cells were then fixed, permeabilized and stained for SAP using an anti-SAP monoclonal antibody. SAP expression was determined via total SAP MFI within each target population using flow cytometry.

**C-D: XLP1-SMART-LV Expression across T cell and NK cell Development 16 Weeks Post Transplant in hIL15 NSG mice.** The following graphs are representative data shown in Figure 3, panels E and F. They depict XLP1-SMART-LV expression across T cell development (C) and NK cell development (D) 16 weeks post transplant in hIL15 NSG mice. mCitrine+ MFI was detected in T and NK cell subpopulations to assess relative XLP1-SMART-LV patterns of expression in comparison to patterns of SAP expression shown in panels A and B.

Figure S4:

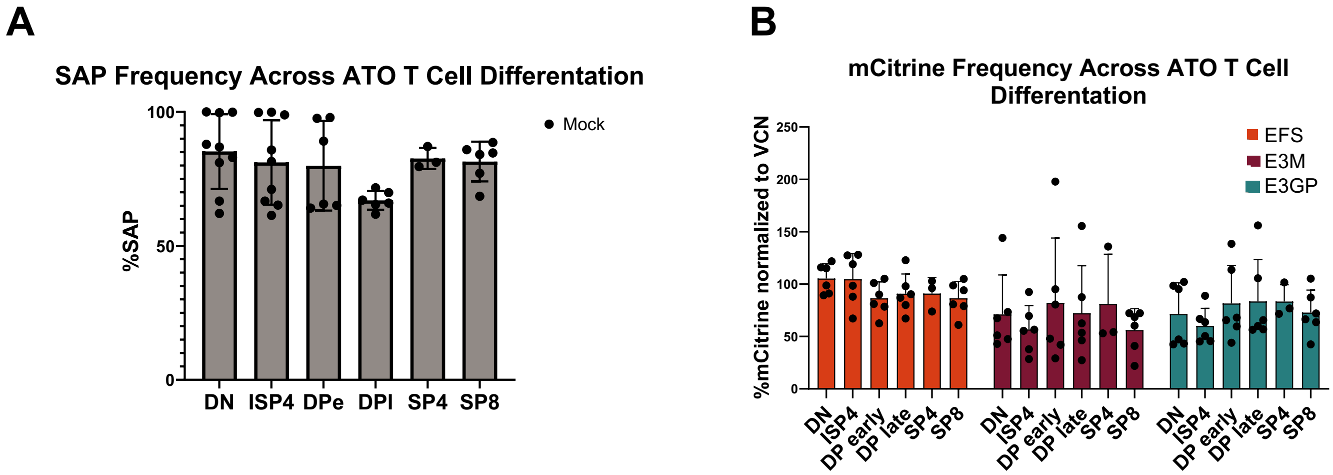


**A-B: Representative SAP and mCitrine Expression within Healthy Donor PBSC CD34+ Derived Artificial Thymic Organoid Cultures.** To determine SAP expression and SMART-LV expression across T cell maturation, healthy donor mobilized peripheral blood CD34+ cells were transduced with XLP1-SMART-LVs harboring an mCitrine reporter cassette and differentiated into T cells using the 3D artificial thymic organoid (ATO) system. At weeks 3, 7, and 12 of differentiation, ATOs were harvested and stained to measure their relative **(A)** SAP expression (after fixation and permeabilization) or **(B)** mCitrine expression using flow cytometry. ATOs were stained to differentiate the following stages of T cell development: double negative (DN): hTCRab-hCD3-hCD4-hCD8-; immature single positive 4 (ISP4): hTCRab-hCD3-hCD4+hCD8-; double positive (DP) early: hTCRab-hCD3-hCD4+hCD8+; DP late: hTCRab+hCD3+hCD4+hCD8+; single positive (SP) 4: hTCRab+hCD3+hCD4+hCD8-; and SP8: hTCRab+hCD3+hCD4-hCD8+. A representative plot of the frequency of SAP+ cells **(A)** and the frequency of mCitrine+ cells **(B)** across weeks 3, 7, and 12 is shown, in which data were normalized to the VCN of the bulk ATO populations. VCNs were as follows: EFS = 0.39, E3M = 0.184, E3GP = 0.25. Data are represented as mean ± SD of biological triplicates from one experiment across three timepoints (weeks 3, 7, and 12).

**Figure S5:**

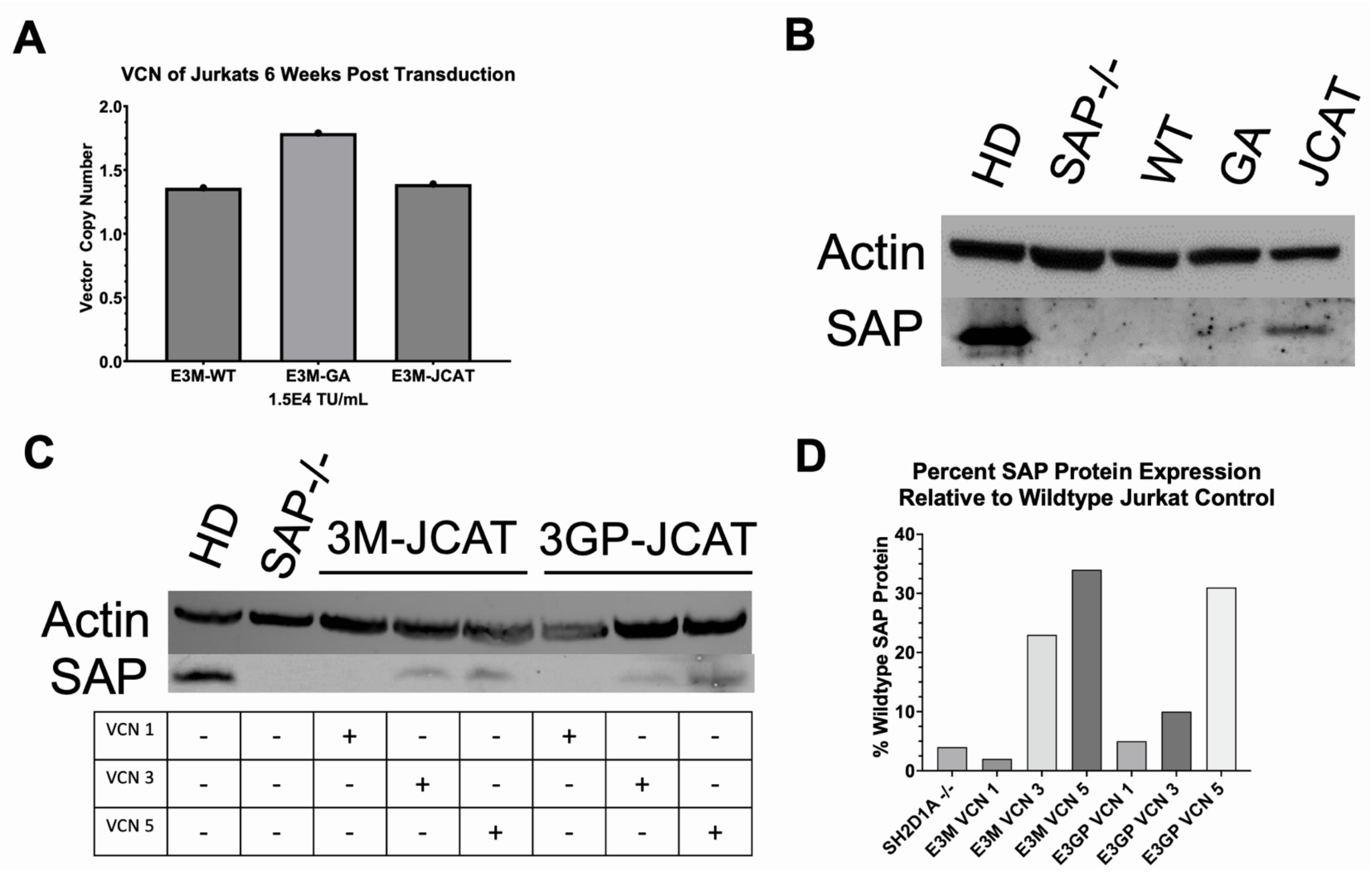


**A: VCN of Jurkat cells 6 weeks post transduction with XLP-SMART-LV.** SH2D1A -/- Jurkat cells were transduced at an equivalent vector copy number (VCN) with the E3M-E20R-5RL lentiviral vector harboring an SH2D1A cDNA cassette containing wildtype codons, GeneArt (GA), or Java Codon Adaptation Tool (JCAT) codon optimizations. 14 days after transduction, cells were harvested for VCN measurement by ddPCR.

**B: Western blot of cells transduced with XLP-SMART-LV.** Protein was extracted from the transduced populations shown in panel A. The reconstitution of SAP protein after transduction was measured by western blot using an anti-SAP antibody (clone 1C9; Abnova) with a 1:1000 dilution of primary antibody.

**C: Western blot for dose response transduction of Jurkat cells transduced with JCat codon optimized XLP1-SMART-LVs.** SH2D1A -/- Jurkat cells were transduced with the JCat codon optimized E3M-E20R-5RL or E3GP-E20R-5RL lentiviral vectors to achieve VCNs of 1,3, and 5. 14 days after transduction, cells were harvested for VCN

measurement by ddPCR. Protein was extracted from the transduced populations and measured by western blot using an anti-SAP antibody (clone 1C9; Abnova).

**D: Densitometry quantification of Western Blot dose response.** The western blot images produced in panel C were quantified for total Actin and SAP protein using densitometry via the ImageJ image processing and analysis tool. After quantification, SAP protein was normalized to the actin loading control. Relative SAP protein expression was determined by measuring the ratio of normalized SAP protein in the experimental condition to the normalized SAP protein of the wildtype Jurkat control.

**Figure S6:**

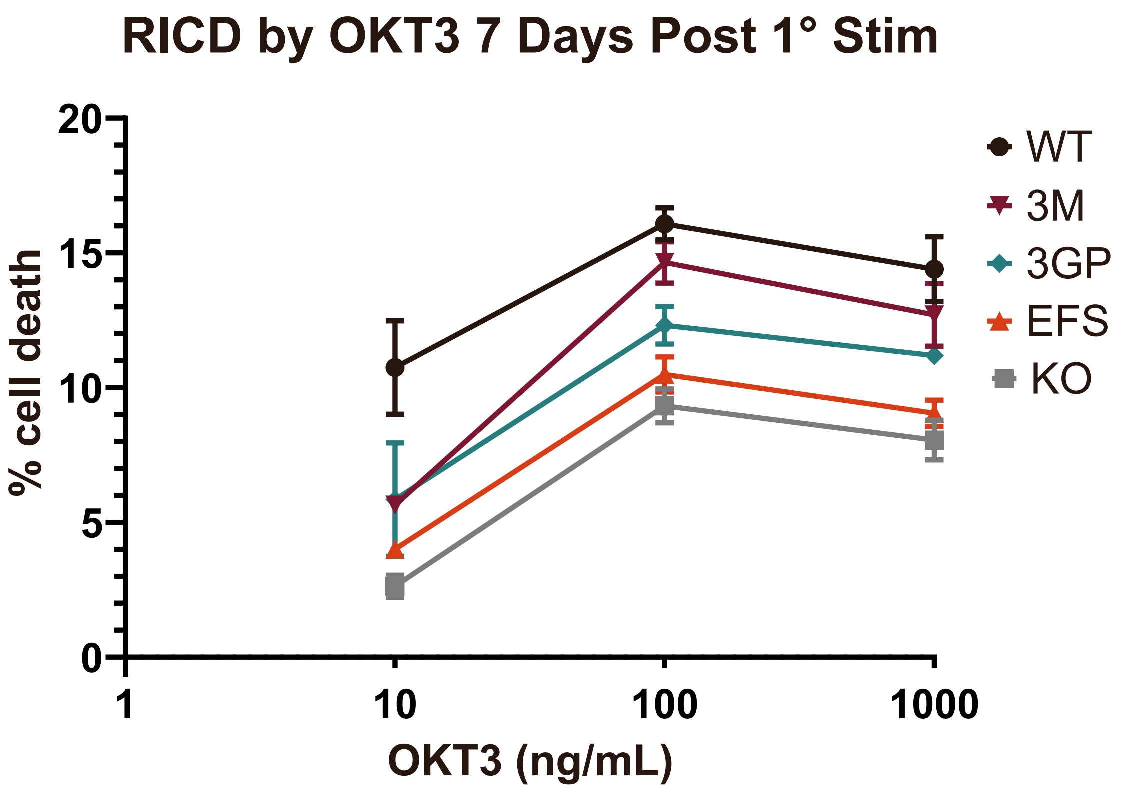


**A: T Cell Restimulation Induced Cell Death (RICD) Assay of SH2D1A-/- CD8+ T Cells Transduced with XLP1-SMART-LVs.** CD8+ T cells from a healthy donor (HD) and were isolated from PBMCs and transfected with sgRNAs targeting Exon 2 of the SH2D1A gene to knockout SAP expression. SH2D1A -/- T cells were transduced with XLP1-SMART-LVs (VCNs are EFS = 2.79, E3M = 2.5, E3GP = 2.46). 10 days after transduction, cells were plated for RICD in OKT3 at final concentrations of 1000 ng/ml, 100 ng/ml and 10 ng/ml. After 24 hours, the recovery of RICD in XLP1-SMART-LV transduced cells was compared to a HD control. The number of live cells (PI-) in stimulated controls were compared to unstimulated controls to measure the % cell loss = [1-(# PI- restimulated cells/# PI- untreated cells)]x100. Data are represented as mean ± SD of biological triplicates from one experiment. We analyzed statistical significance using a two-way ANOVA followed by multiple paired comparisons for normally distributed data (Tukey test). All statistical tests were two-tailed and a p value of < 0.05 was deemed significant (ns non-significant, *P < 0.05, **P < 0.01, ***P < 0.001, ****P < 0.0001). Compared to XLP knockout samples, EFS was deemed significant with a p value <0.05; E3M was deemed significant with a p value <0.0001; and E3GP was deemed significant with a p value <0.0001.

**Figure S7:**

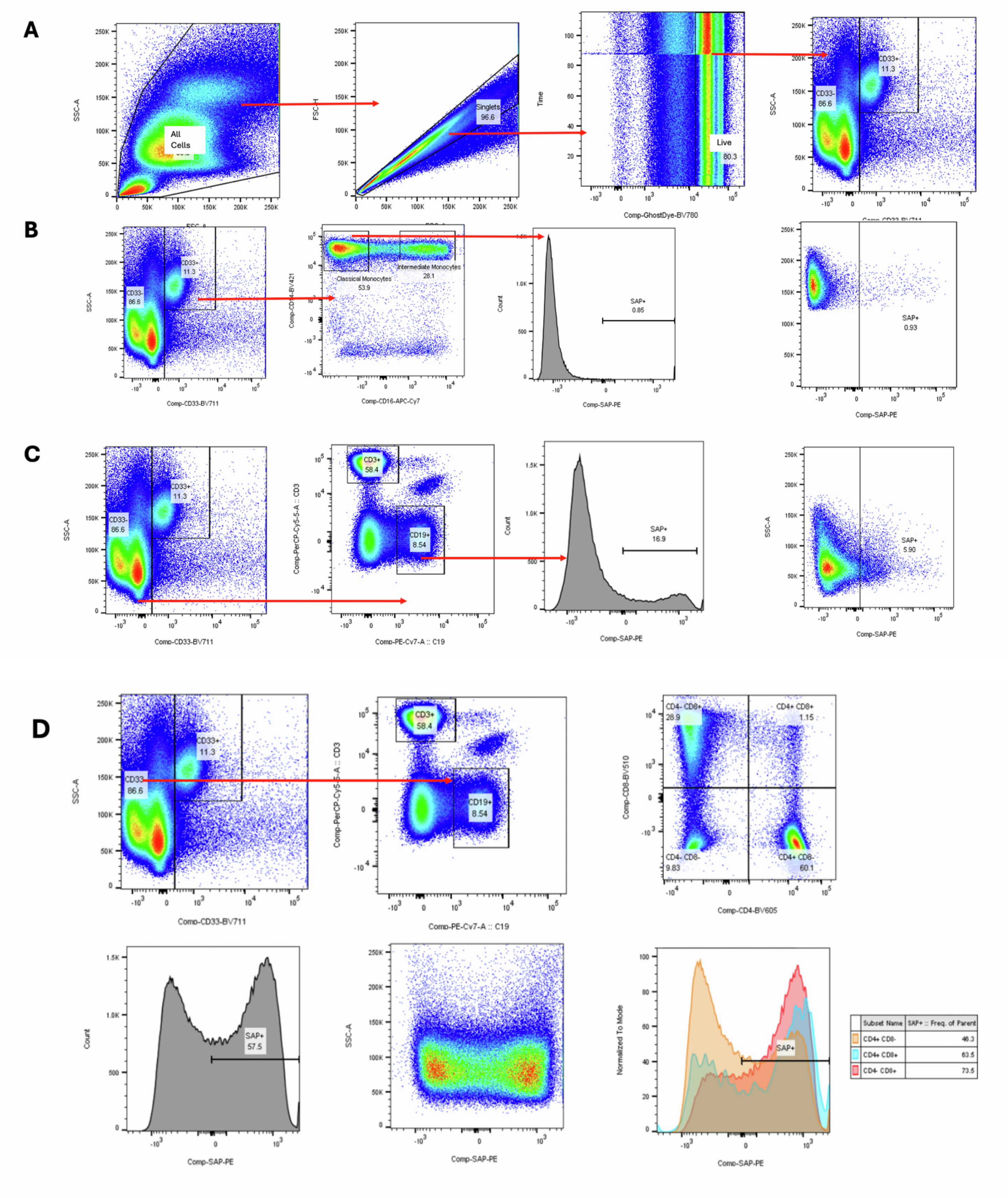
A
B
C
D
All Cells
Singlets 96.6
Live 80.3
CD33+ 11.3
CD33- 86.6
Classical Monocytes 53.9
Intermediate Monocytes 28.1
SAP+ 0.85
SAP+ 0.93
CD3+ 58.4
CD19+ 8.54
SAP+ 16.9
SAP+ 5.90
CD4- CD8+ 28.9
CD4+ CD8+ 1.15
CD4- CD8- 9.83
CD4+ CD8- 60.1
SAP+ 57.5
SAP+
SSC-A
FSC-H
Time
Count
Normalized To Mode
Comp-GhostDye-BV780
Comp-CD33-BV711
Comp-CD14-BV421
Comp-CD16-APC-Cy7
Comp-SAP-PE
Comp-PerCP-Cy5-5-A :: CD3
Comp-PE-Cy7-A :: C19
Comp-CD8-BV510
Comp-CD4-BV605
Subset Name
SAP+ :: Freq. of Parent
CD4+ CD8- 46.3
CD4+ CD8+ 63.5
CD4- CD8+ 73.5

E

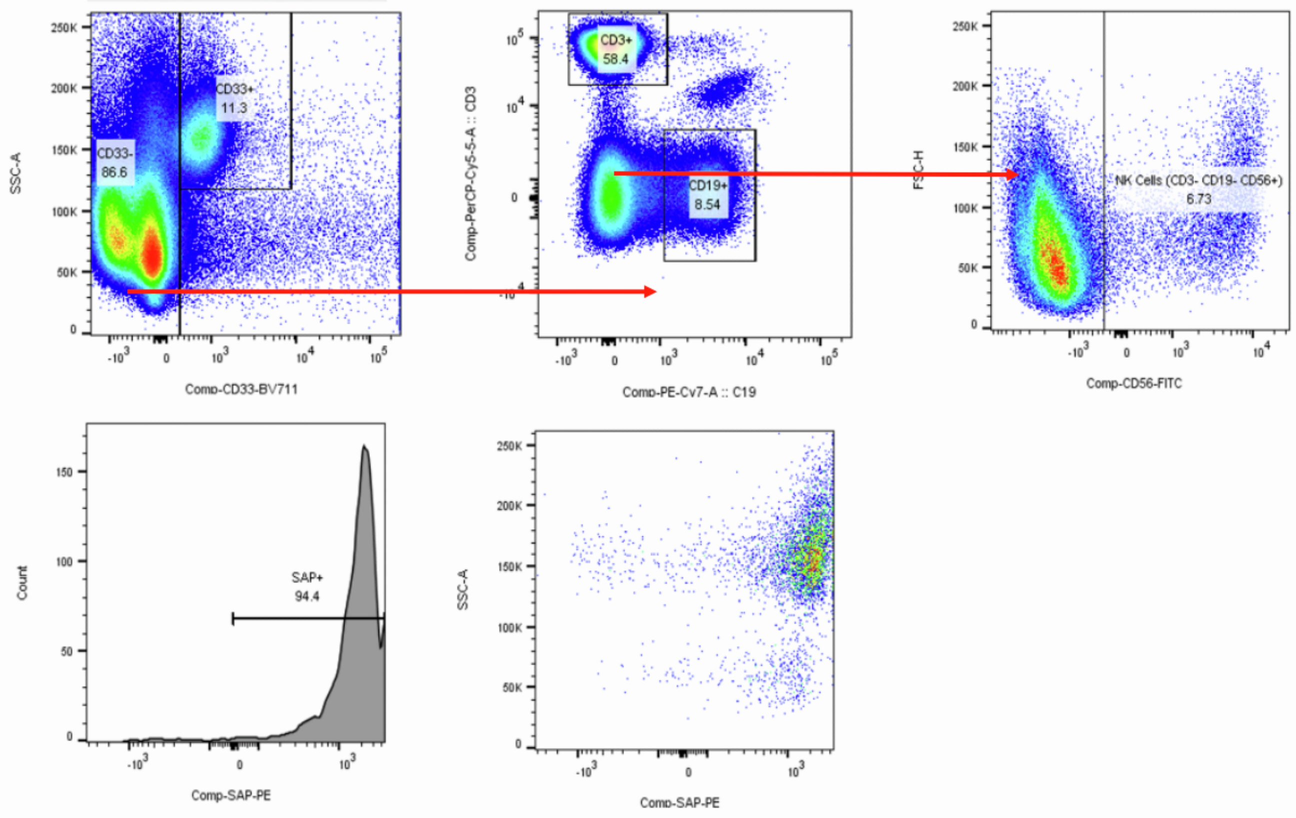

CD33+
11.3
CD33-
86.6
SSC-A
Comp-CD33-BV711
CD3+
58.4
CD19+
8.54
Comp-PerCP-Cy5-5-A :: CD3
Comp-PE-Cy7-A :: C19
NK Cells (CD3- CD19- CD56+)
6.73
FSC-H
Comp-CD56-FITC
SAP+
94.4
Count
Comp-SAP-PE
SSC-A
Comp-SAP-PE


F

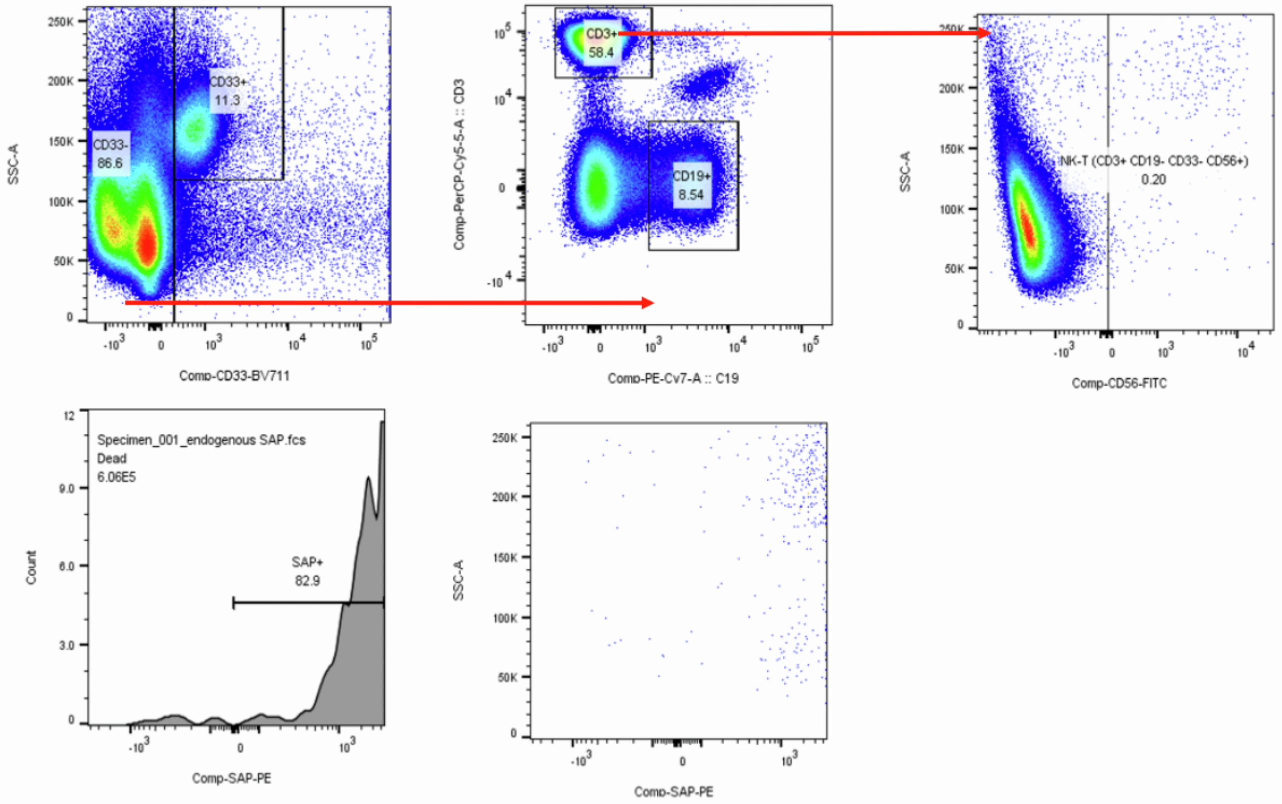

CD33+
11.3
CD33-
86.6
SSC-A
Comp-CD33-BV711
CD3+
58.4
CD19+
8.54
Comp-PerCP-Cy5-5-A :: CD3
Comp-PE-Cy7-A :: C19
NK-T (CD3+ CD19- CD33- CD56+)
0.20
SSC-A
Comp-CD56-FITC
Specimen_001_endogenous SAP.fcs
Dead
6.06E5
SAP+
82.9
Count
Comp-SAP-PE
SSC-A
Comp-SAP-PE

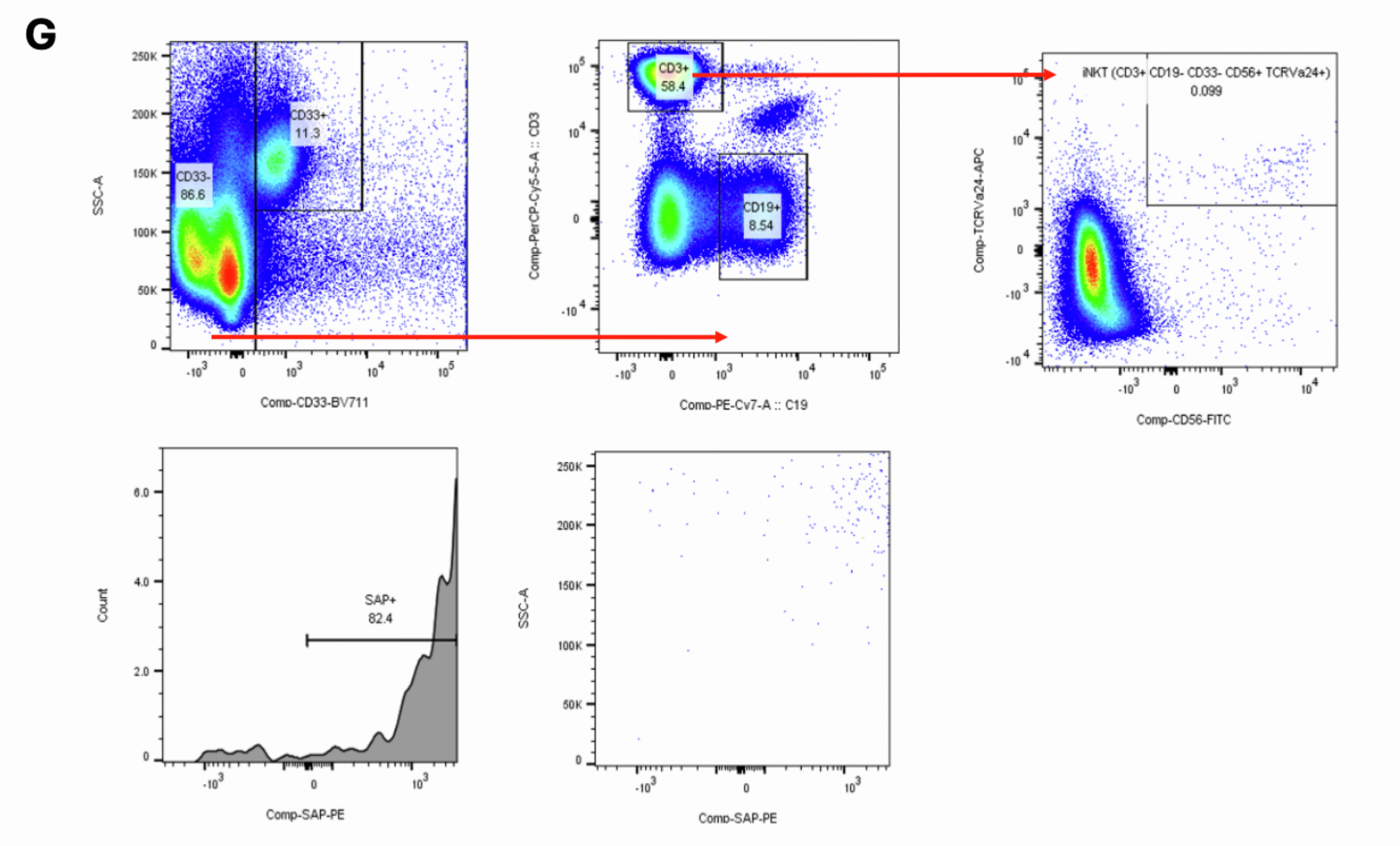


**Flow Gating for SAP quantification within PBMC Lineages.** (A) Parent Gating; (B) Monocytes; (C) B Cells; (D) T Cells; (E) NK Cells; (F) NKT Cells; (G) iNKT Cells.

**Figure S8:**

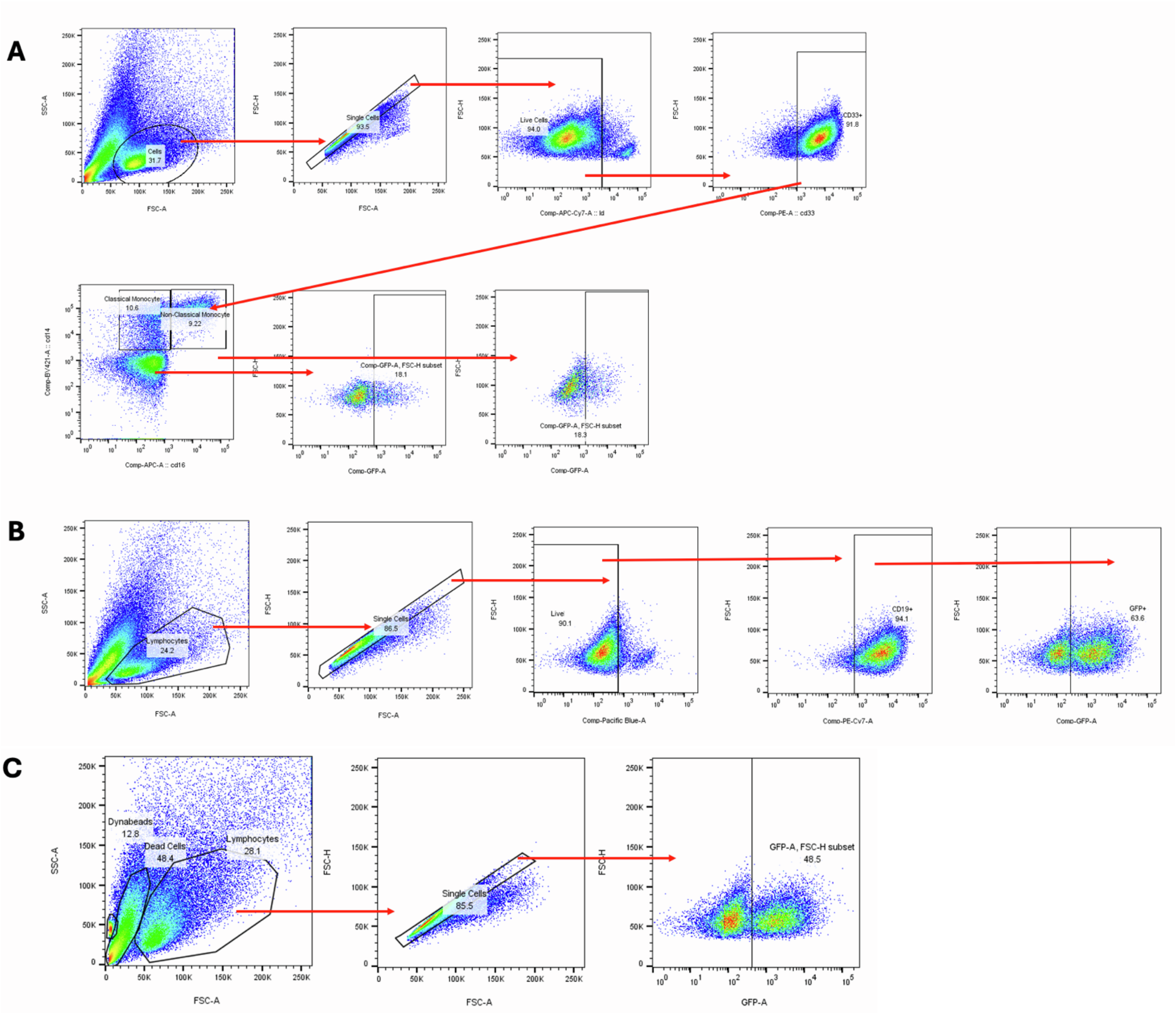


**mCitrine MFI Flow Cytometry Gating for Enhancers in T Cells, Monocytes, and B-LCLs.** (A) Monocyte gating; (B) B-LCL gating; (C) T Cell gating.

**Figure S9:**

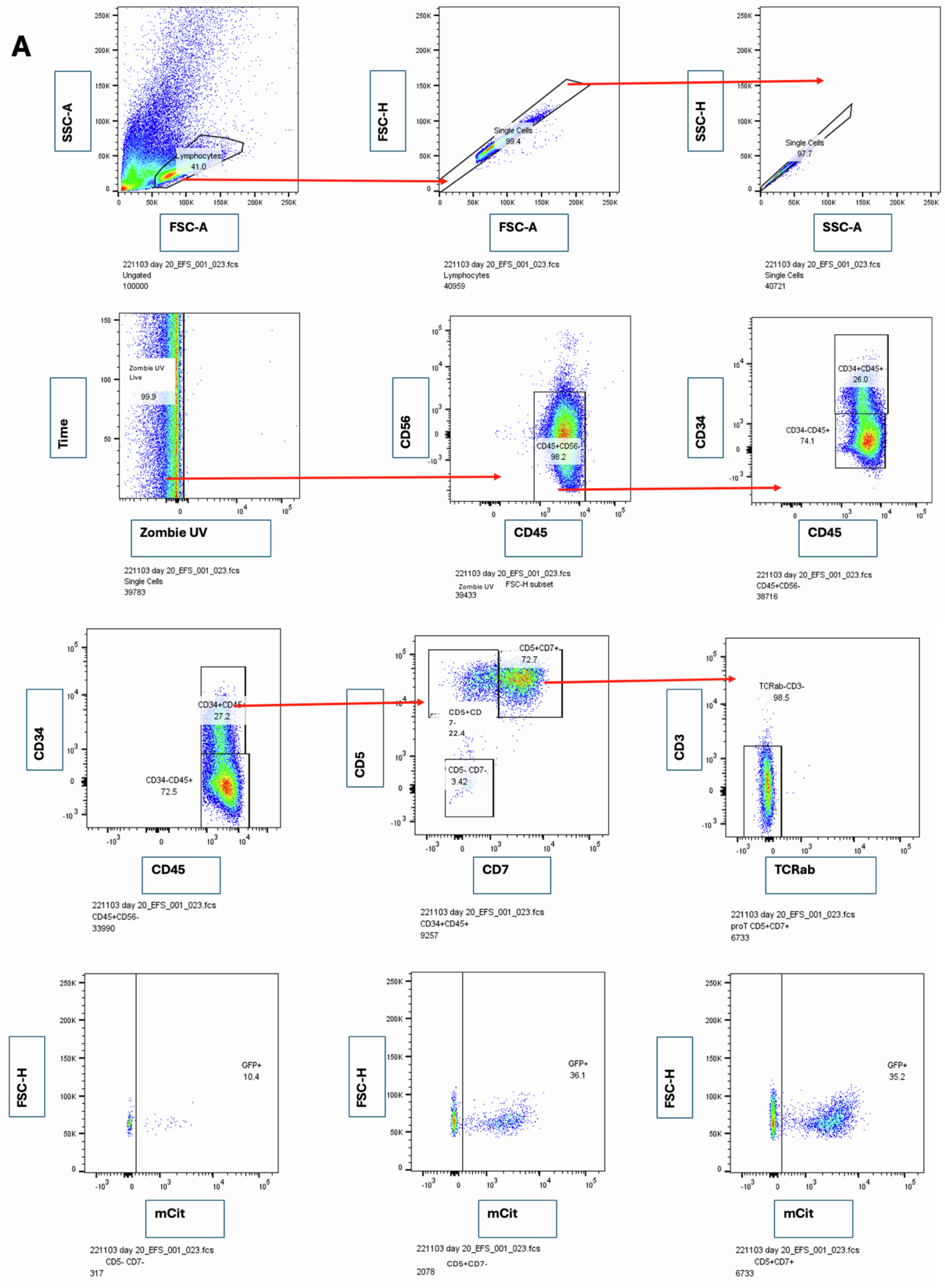
A
SSC-A
FSC-A
Lymphocytes
41.0
221103 day 20_EFS_001_023.fcs
Ungated
100000
FSC-H
FSC-A
Single Cells
99.4
221103 day 20_EFS_001_023.fcs
Lymphocytes
40959
SSC-H
SSC-A
Single Cells
97.7
221103 day 20_EFS_001_023.fcs
Single Cells
40721
Time
Zombie UV
Zombie UV Live
99.9
221103 day 20_EFS_001_023.fcs
Single Cells
39783
CD56
CD45
CD45+CD56-
98.2
221103 day 20_EFS_001_023.fcs
Zombie UV FSC-H subset
39433
CD34
CD45
CD34+CD45+
26.0
CD34-CD45+
74.1
221103 day 20_EFS_001_023.fcs
CD45+CD56-
38716
CD34
CD45
CD34+CD45+
27.2
CD34-CD45+
72.5
221103 day 20_EFS_001_023.fcs
CD45+CD56-
33990
CD5
CD7
CD5+CD7+
72.7
CD5+CD7-
22.4
CD5- CD7-
3.42
221103 day 20_EFS_001_023.fcs
CD34+CD45+
9257
CD3
TCRab
TCRab-CD3-
98.5
221103 day 20_EFS_001_023.fcs
proT CD5+CD7+
6733
FSC-H
mCit
GFP+
10.4
221103 day 20_EFS_001_023.fcs
CD5- CD7-
317
FSC-H
mCit
GFP+
36.1
221103 day 20_EFS_001_023.fcs
CD5+CD7-
2078
FSC-H
mCit
GFP+
35.2
221103 day 20_EFS_001_023.fcs
CD5+CD7+
6733

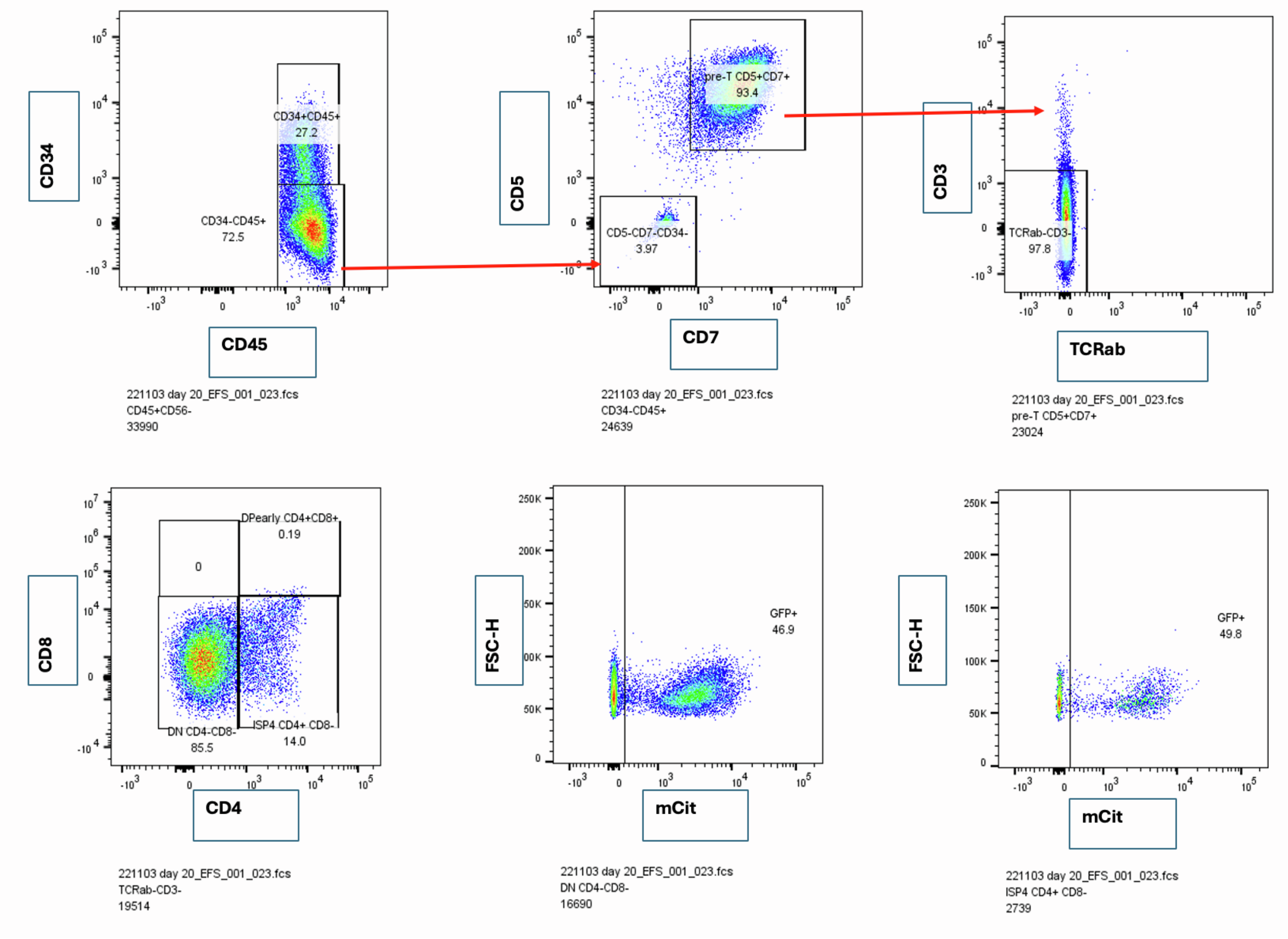
CD34
CD45
CD34+CD45+
27.2
CD34-CD45+
72.5
221103 day 20_EFS_001_023.fcs
CD45+CD56-
33990
CD5
CD7
pre-T CD5+CD7+
93.4
CD5-CD7-CD34-
3.97
221103 day 20_EFS_001_023.fcs
CD34-CD45+
24639
CD3
TCRab
TCRab-CD3-
97.8
221103 day 20_EFS_001_023.fcs
pre-T CD5+CD7+
23024
CD8
CD4
DPearly CD4+CD8+
0.19
0
DN CD4-CD8-
85.5
ISP4 CD4+ CD8-
14.0
221103 day 20_EFS_001_023.fcs
TCRab-CD3-
19514
FSC-H
mCit
GFP+
46.9
221103 day 20_EFS_001_023.fcs
DN CD4-CD8-
16690
FSC-H
mCit
GFP+
49.8
221103 day 20_EFS_001_023.fcs
ISP4 CD4+ CD8-
2739

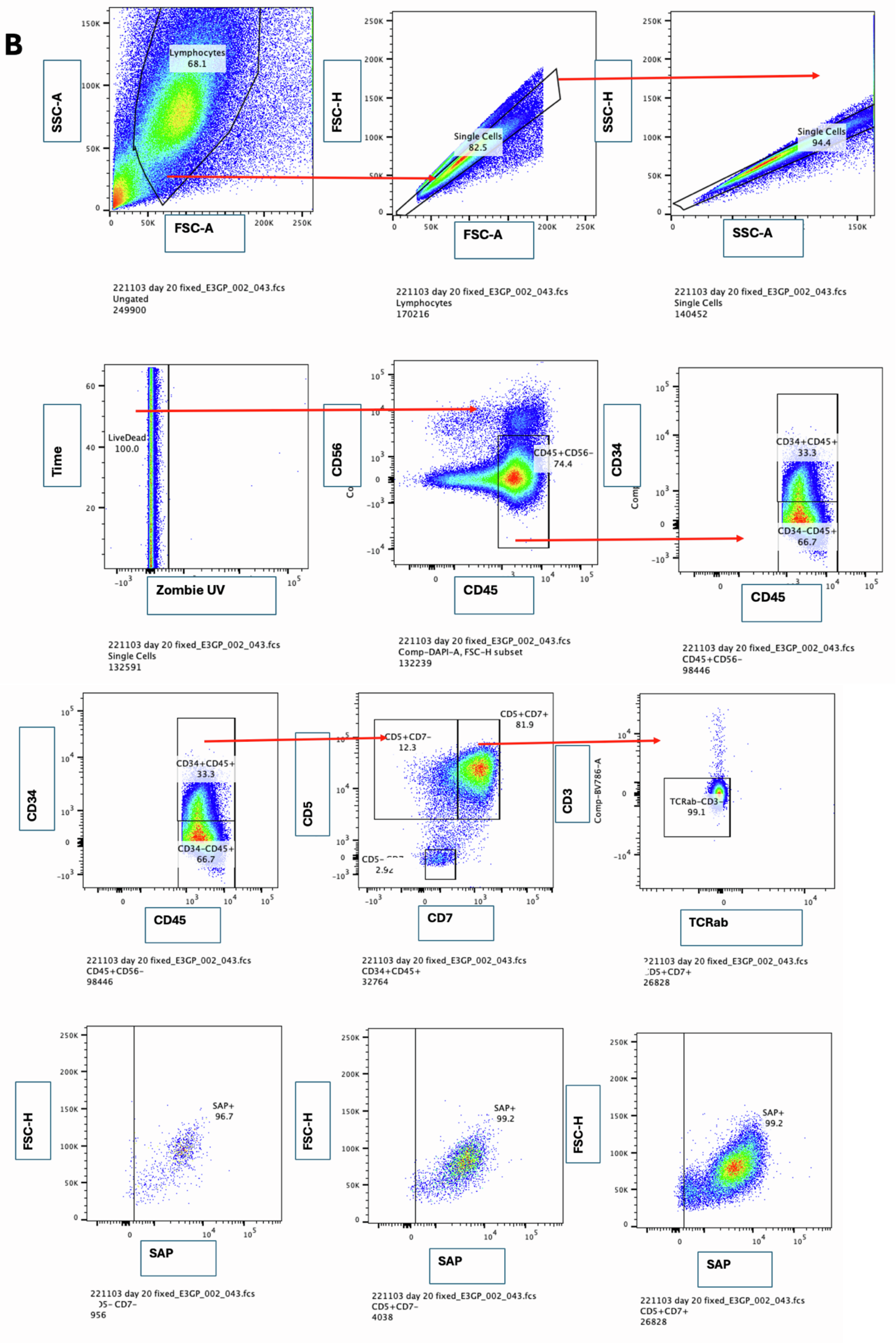
B
SSC-A
FSC-A
Lymphocytes 68.1
221103 day 20 fixed_E3GP_002_043.fcs
Ungated
249900
FSC-H
FSC-A
Single Cells 82.5
221103 day 20 fixed_E3GP_002_043.fcs
Lymphocytes
170216
SSC-H
SSC-A
Single Cells 94.4
221103 day 20 fixed_E3GP_002_043.fcs
Single Cells
140452
Time
Zombie UV
LiveDead 100.0
221103 day 20 fixed_E3GP_002_043.fcs
Single Cells
132591
CD56
CD45
CD45+CD56- 74.4
221103 day 20 fixed_E3GP_002_043.fcs
Comp-DAPI-A, FSC-H subset
132239
CD34
CD45
CD34+CD45+ 33.3
CD34-CD45+ 66.7
221103 day 20 fixed_E3GP_002_043.fcs
CD45+CD56-
98446
CD34
CD45
CD34+CD45+ 33.3
CD34-CD45+ 66.7
221103 day 20 fixed_E3GP_002_043.fcs
CD45+CD56-
98446
CD5
CD7
CD5+CD7- 12.3
CD5+CD7+ 81.9
2.92
221103 day 20 fixed_E3GP_002_043.fcs
CD34+CD45+
32764
CD3
Comp-BV786-A
TCRab
TCRab-CD3- 99.1
CD5+CD7+
26828
FSC-H
SAP
SAP+ 96.7
221103 day 20 fixed_E3GP_002_043.fcs
956
FSC-H
SAP
SAP+ 99.2
221103 day 20 fixed_E3GP_002_043.fcs
CD5+CD7-
4038
FSC-H
SAP
SAP+ 99.2
221103 day 20 fixed_E3GP_002_043.fcs
CD5+CD7+
26828

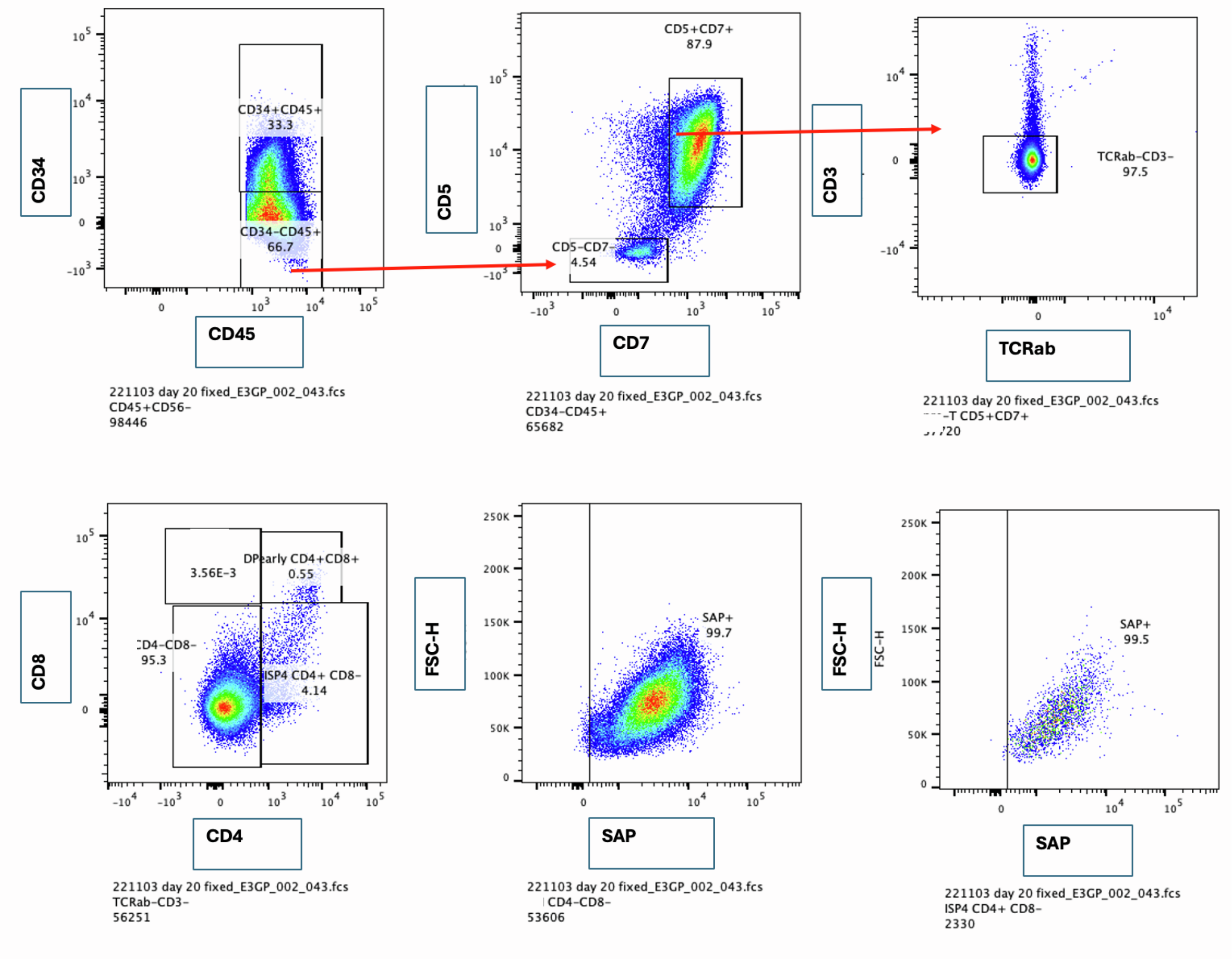
CD34
CD45
CD34+CD45+
33.3
CD34-CD45+
66.7
221103 day 20 fixed_E3GP_002_043.fcs
CD45+CD56-
98446
CD5
CD7
CD5+CD7+
87.9
CD5-CD7-
4.54
221103 day 20 fixed_E3GP_002_043.fcs
CD34-CD45+
65682
CD3
TCRab
TCRab-CD3-
97.5
221103 day 20 fixed_E3GP_002_043.fcs
CD8
CD4
3.56E-3
DPearly CD4+CD8+
0.55
95.3
ISP4 CD4+ CD8-
4.14
221103 day 20 fixed_E3GP_002_043.fcs
TCRab-CD3-
56251
FSC-H
SAP
SAP+
99.7
221103 day 20 fixed_E3GP_002_043.fcs
CD4-CD8-
53606
FSC-H
SAP
SAP+
99.5
221103 day 20 fixed_E3GP_002_043.fcs
ISP4 CD4+ CD8-
2330

**c**

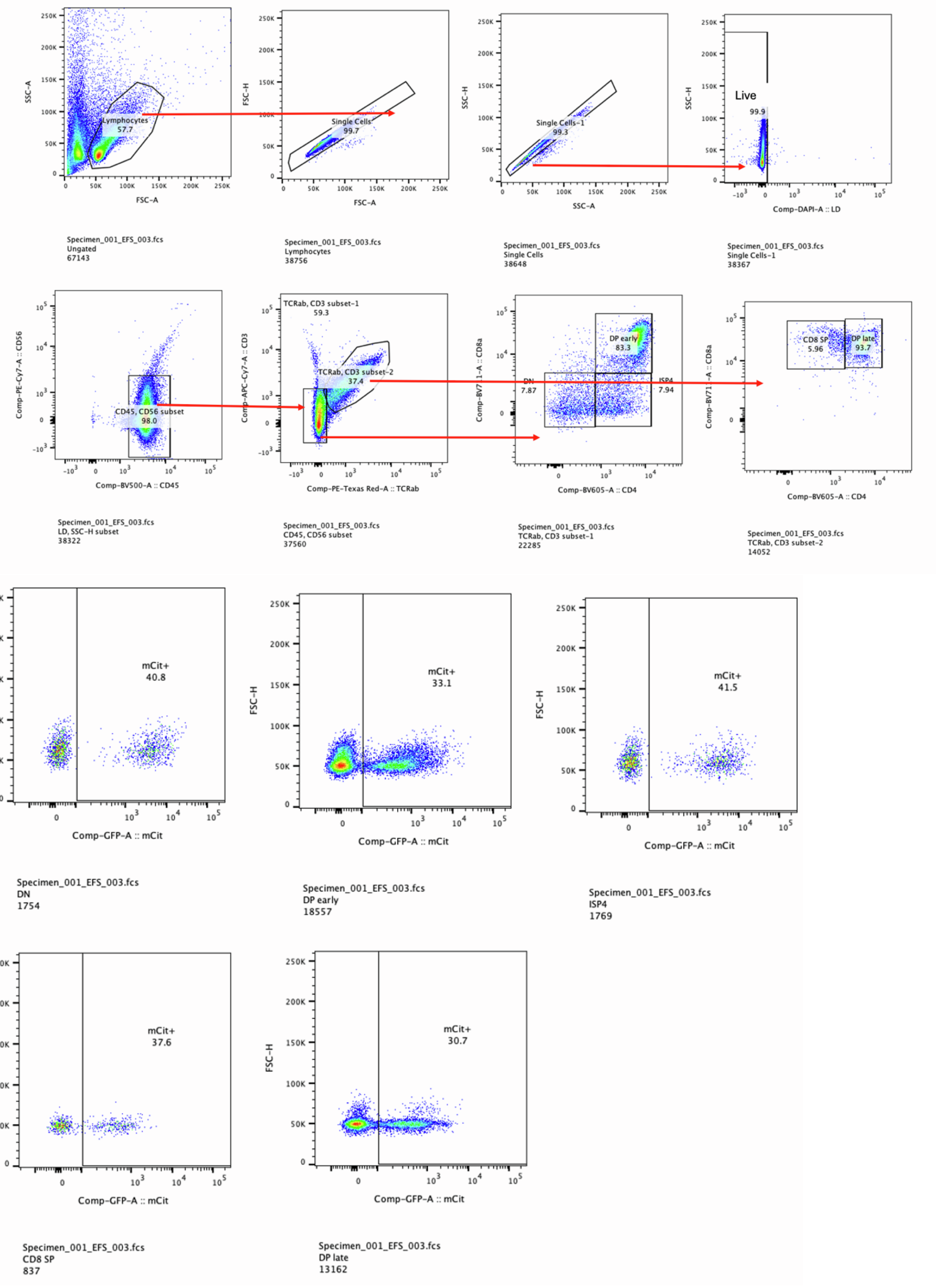
Lymphocytes
57.7
SSC-A
FSC-A
Specimen_001_EFS_003.fcs
Ungated
67143
Single Cells
99.7
FSC-H
FSC-A
Specimen_001_EFS_003.fcs
Lymphocytes
38756
Single Cells-1
99.3
SSC-H
SSC-A
Specimen_001_EFS_003.fcs
Single Cells
38648
Live
99.9
SSC-H
Comp-DAPI-A :: LD
Specimen_001_EFS_003.fcs
Single Cells-1
38367
CD45, CD56 subset
98.0
Comp-PE-Cy7-A :: CD56
Comp-BV500-A :: CD45
Specimen_001_EFS_003.fcs
LD, SSC-H subset
38322
TCRab, CD3 subset-1
59.3
TCRab, CD3 subset-2
37.4
Comp-APC-Cy7-A :: CD3
Comp-PE-Texas Red-A :: TCRab
Specimen_001_EFS_003.fcs
CD45, CD56 subset
37560
DP early
83.3
DN
7.87
ISP4
7.94
Comp-BV605-A :: CD4
Specimen_001_EFS_003.fcs
TCRab, CD3 subset-1
22285
CD8 SP
5.96
DP late
93.7
Comp-BV605-A :: CD4
Specimen_001_EFS_003.fcs
TCRab, CD3 subset-2
14052
mCit+
40.8
FSC-H
Comp-GFP-A :: mCit
Specimen_001_EFS_003.fcs
DN
1754
mCit+
33.1
FSC-H
Comp-GFP-A :: mCit
Specimen_001_EFS_003.fcs
DP early
18557
mCit+
41.5
FSC-H
Comp-GFP-A :: mCit
Specimen_001_EFS_003.fcs
ISP4
1769
mCit+
37.6
FSC-H
Comp-GFP-A :: mCit
Specimen_001_EFS_003.fcs
CD8 SP
837
mCit+
30.7
FSC-H
Comp-GFP-A :: mCit
Specimen_001_EFS_003.fcs
DP late
13162

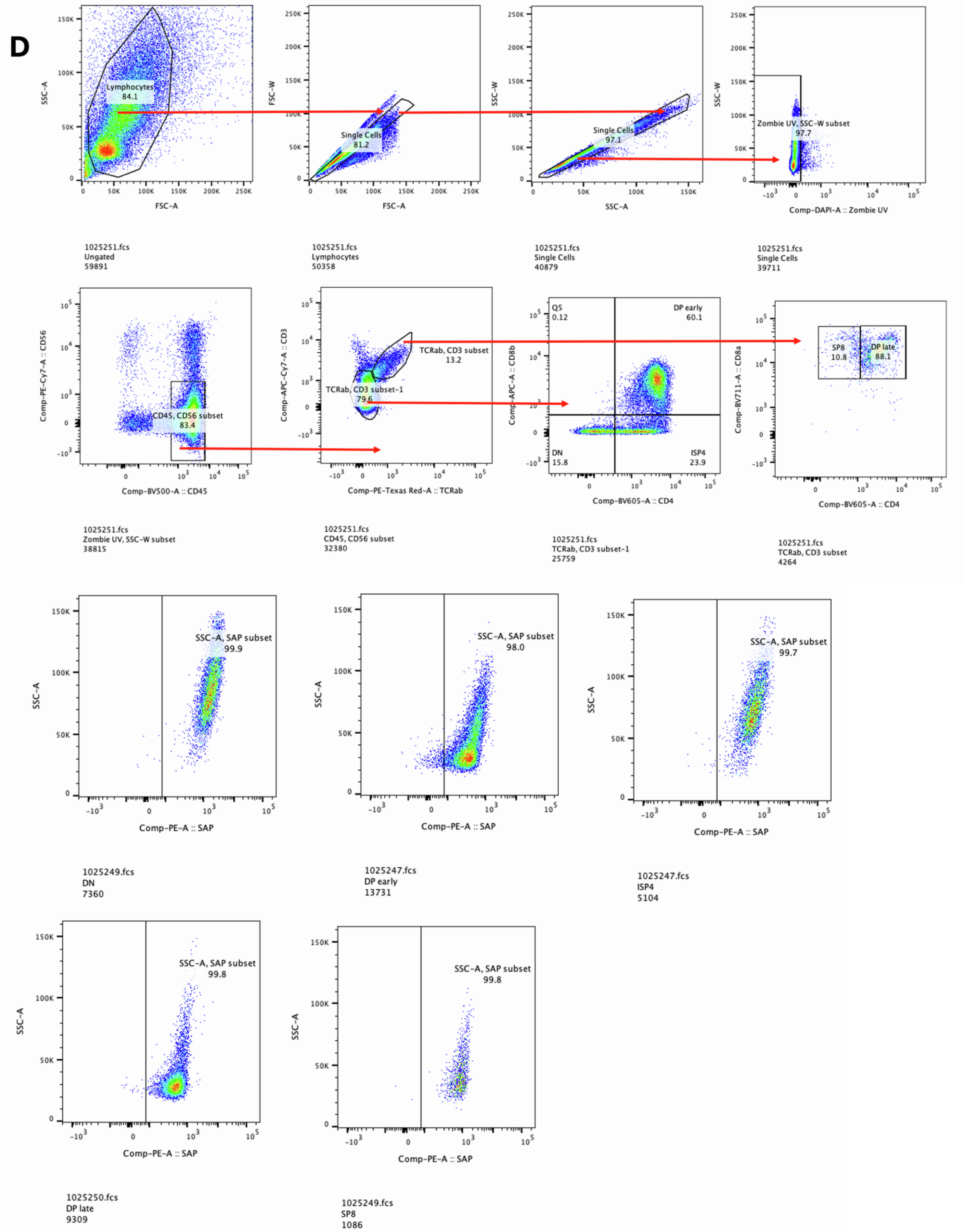
D
Lymphocytes
84.1
SSC-A
FSC-A
1025251.fcs
Ungated
59891
Single Cells
81.2
FSC-W
FSC-A
1025251.fcs
Lymphocytes
50358
Single Cells
97.1
SSC-W
SSC-A
1025251.fcs
Single Cells
40879
Zombie UV, SSC-W subset
97.7
SSC-W
Comp-DAPI-A :: Zombie UV
1025251.fcs
Single Cells
39711
CD45, CD56 subset
83.4
Comp-PE-Cy7-A :: CD56
Comp-BV500-A :: CD45
1025251.fcs
Zombie UV, SSC-W subset
38815
TCRab, CD3 subset
13.2
TCRab, CD3 subset-1
79.6
Comp-APC-Cy7-A :: CD3
Comp-PE-Texas Red-A :: TCRab
1025251.fcs
CD45, CD56 subset
32380
Q5
0.12
DP early
60.1
DN
15.8
ISP4
23.9
Comp-APC-A :: CD8b
Comp-BV605-A :: CD4
1025251.fcs
TCRab, CD3 subset-1
25759
SP8
10.8
DP late
88.1
Comp-BV711-A :: CD8a
Comp-BV605-A :: CD4
1025251.fcs
TCRab, CD3 subset
4264
SSC-A, SAP subset
99.9
SSC-A
Comp-PE-A :: SAP
1025249.fcs
DN
7360
SSC-A, SAP subset
98.0
SSC-A
Comp-PE-A :: SAP
1025247.fcs
DP early
13731
SSC-A, SAP subset
99.7
SSC-A
Comp-PE-A :: SAP
1025247.fcs
ISP4
5104
SSC-A, SAP subset
99.8
SSC-A
Comp-PE-A :: SAP
1025250.fcs
DP late
9309
SSC-A, SAP subset
99.8
SSC-A
Comp-PE-A :: SAP
1025249.fcs
SP8
1086

**E**

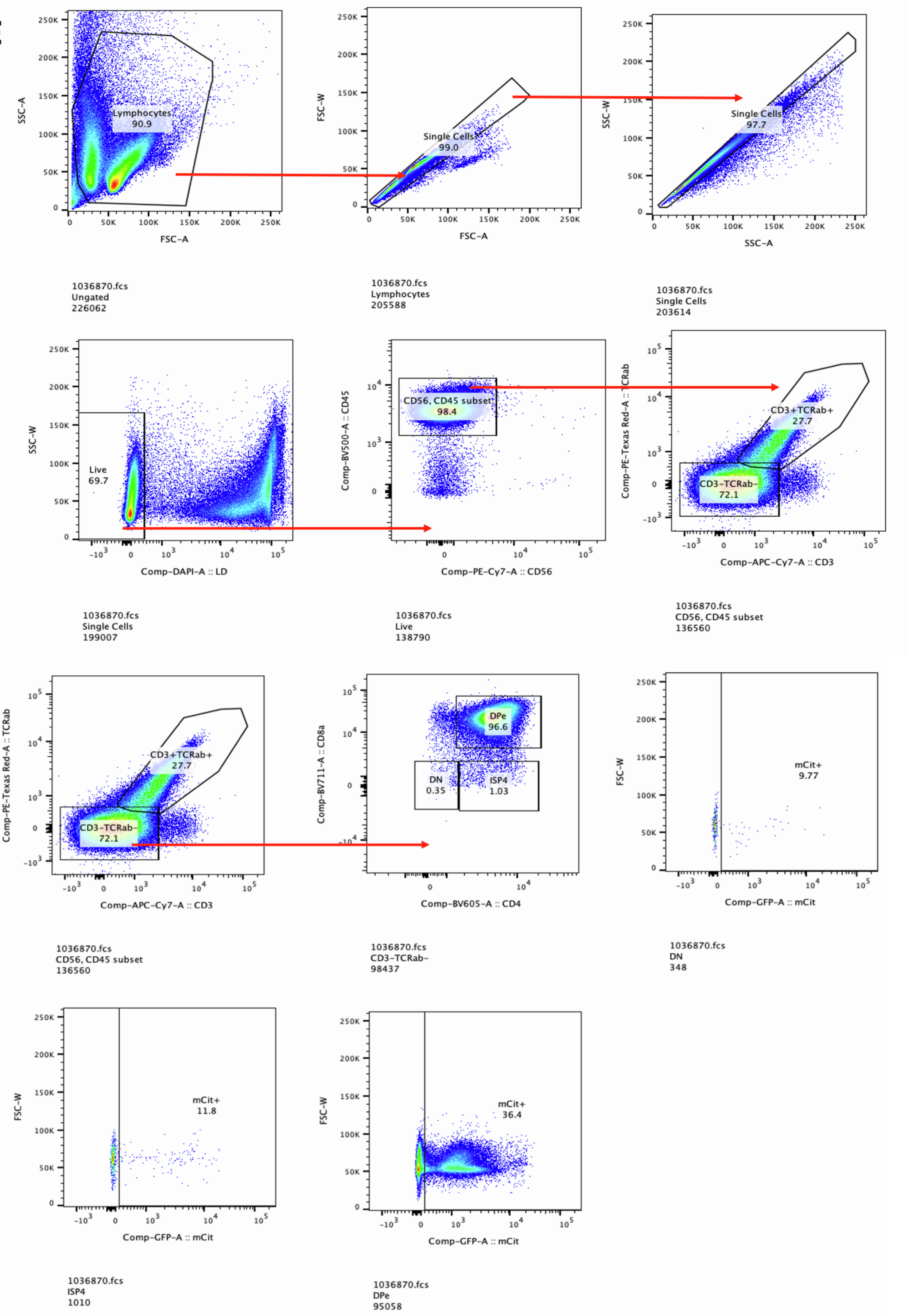
Lymphocytes
90.9
SSC-A
FSC-A
1036870.fcs
Ungated
226062
Single Cells
99.0
FSC-W
FSC-A
1036870.fcs
Lymphocytes
205588
Single Cells
97.7
SSC-W
SSC-A
1036870.fcs
Single Cells
203614
Live
69.7
SSC-W
Comp-DAPI-A :: LD
1036870.fcs
Single Cells
199007
CD56, CD45 subset
98.4
Comp-BV500-A :: CD45
Comp-PE-Cy7-A :: CD56
1036870.fcs
Live
138790
CD3+TCRab+
27.7
CD3-TCRab-
72.1
Comp-PE-Texas Red-A :: TCRab
Comp-APC-Cy7-A :: CD3
1036870.fcs
CD56, CD45 subset
136560
CD3+TCRab+
27.7
CD3-TCRab-
72.1
Comp-PE-Texas Red-A :: TCRab
Comp-APC-Cy7-A :: CD3
1036870.fcs
CD56, CD45 subset
136560
DPe
96.6
DN
0.35
ISP4
1.03
Comp-BV711-A :: CD8a
Comp-BV605-A :: CD4
1036870.fcs
CD3-TCRab-
98437
mCit+
9.77
FSC-W
Comp-GFP-A :: mCit
1036870.fcs
DN
348
mCit+
11.8
FSC-W
Comp-GFP-A :: mCit
1036870.fcs
ISP4
1010
mCit+
36.4
FSC-W
Comp-GFP-A :: mCit
1036870.fcs
DPe
95058

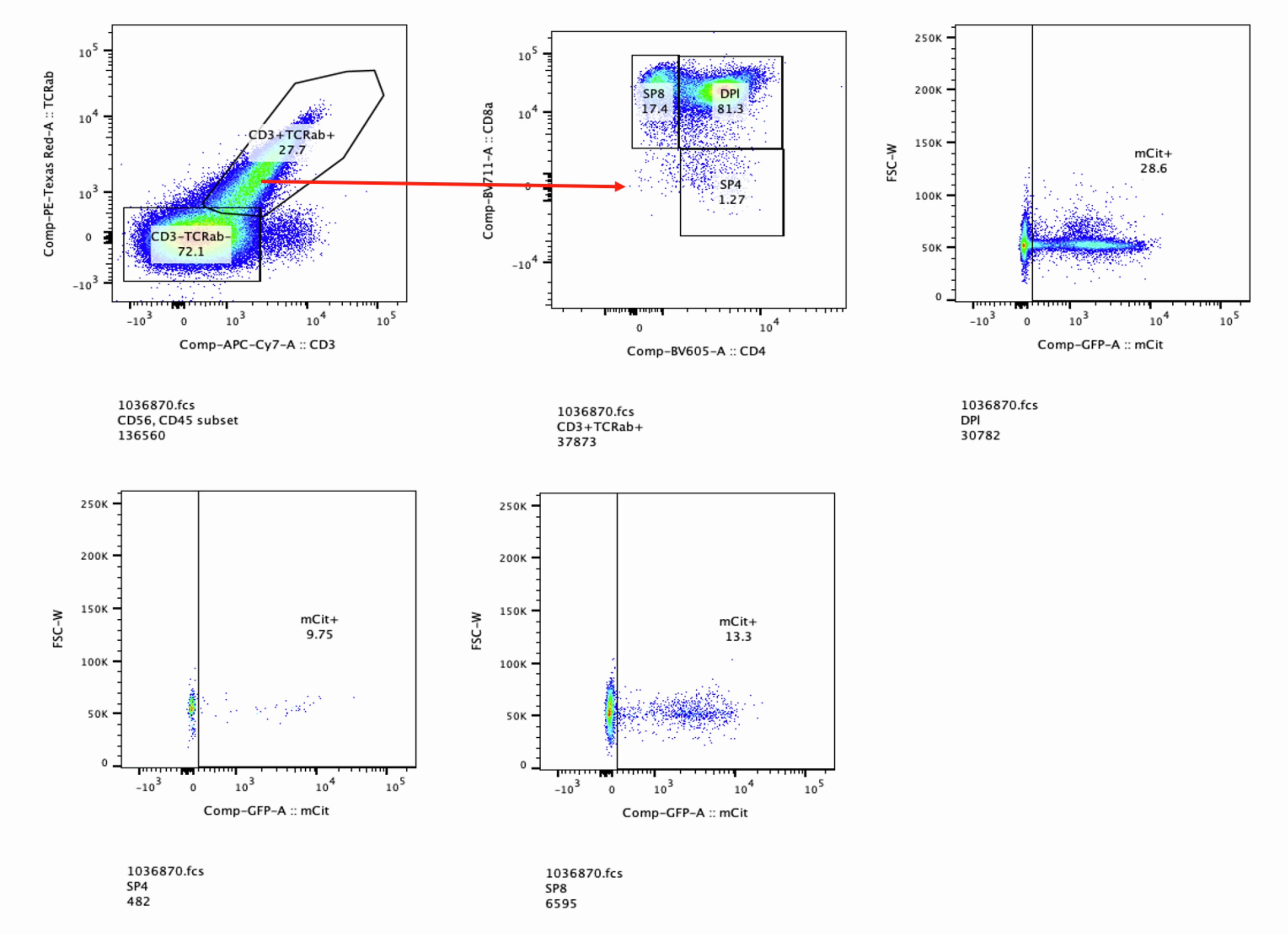
Comp-PE-Texas Red-A :: TCRab
Comp-APC-Cy7-A :: CD3
CD3+TCRab+
27.7
CD3-TCRab-
72.1
1036870.fcs
CD56, CD45 subset
136560
Comp-BV711-A :: CD8a
Comp-BV605-A :: CD4
SP8
17.4
DPl
81.3
SP4
1.27
1036870.fcs
CD3+TCRab+
37873
FSC-W
Comp-GFP-A :: mCit
mCit+
28.6
1036870.fcs
DPl
30782
FSC-W
Comp-GFP-A :: mCit
mCit+
9.75
1036870.fcs
SP4
482
FSC-W
Comp-GFP-A :: mCit
mCit+
13.3
1036870.fcs
SP8
6595

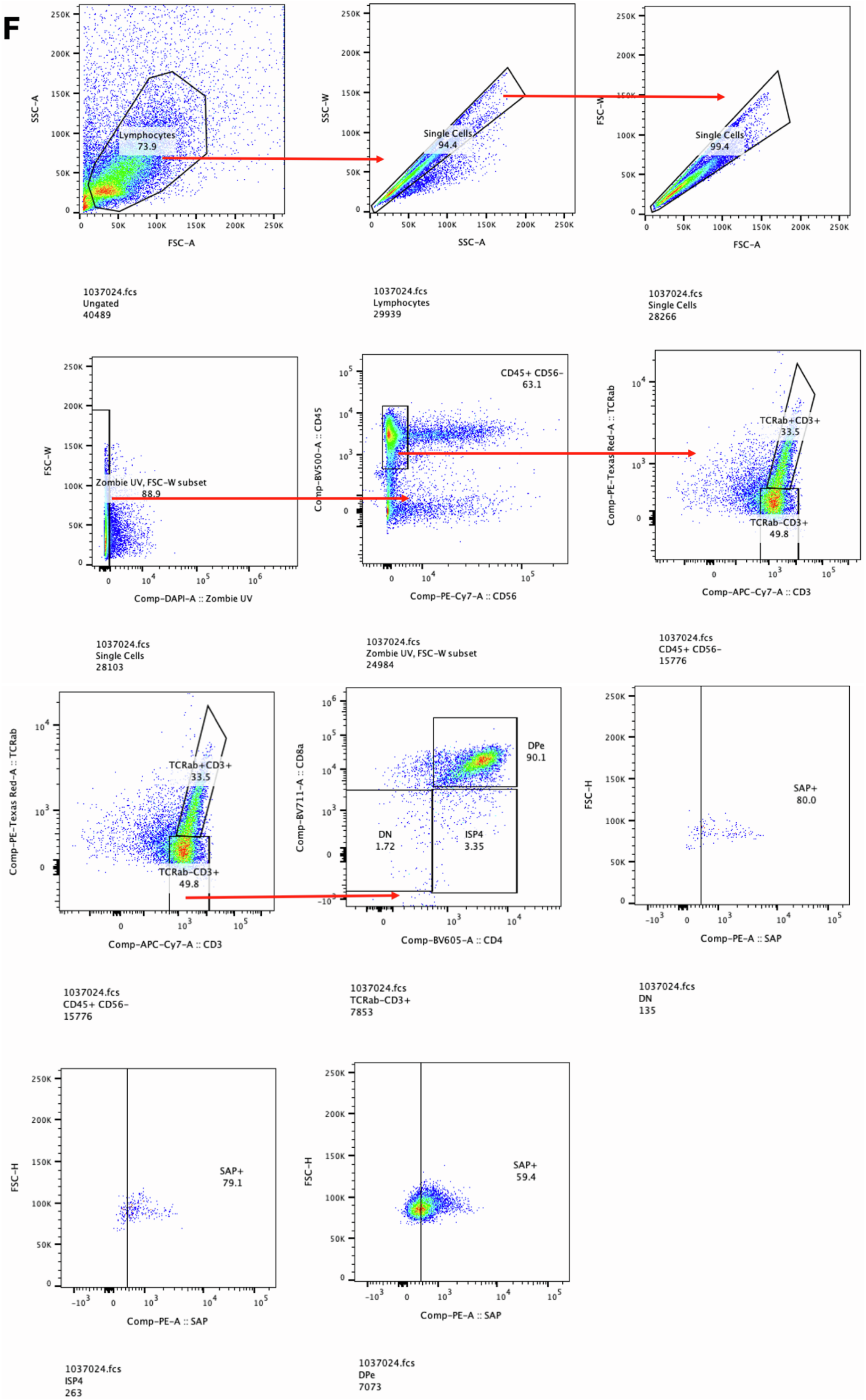
F
Lymphocytes
73.9
SSC-A
FSC-A
1037024.fcs
Ungated
40489
Single Cells
94.4
SSC-W
SSC-A
1037024.fcs
Lymphocytes
29939
Single Cells
99.4
FSC-W
FSC-A
1037024.fcs
Single Cells
28266
Zombie UV, FSC-W subset
88.9
FSC-W
Comp-DAPI-A :: Zombie UV
1037024.fcs
Single Cells
28103
CD45+ CD56-
63.1
Comp-BV500-A :: CD45
Comp-PE-Cy7-A :: CD56
1037024.fcs
Zombie UV, FSC-W subset
24984
TCRab+CD3+
33.5
TCRab-CD3+
49.8
Comp-PE-Texas Red-A :: TCRab
Comp-APC-Cy7-A :: CD3
1037024.fcs
CD45+ CD56-
15776
TCRab+CD3+
33.5
TCRab-CD3+
49.8
Comp-PE-Texas Red-A :: TCRab
Comp-APC-Cy7-A :: CD3
1037024.fcs
CD45+ CD56-
15776
DPe
90.1
DN
1.72
ISP4
3.35
Comp-BV711-A :: CD8a
Comp-BV605-A :: CD4
1037024.fcs
TCRab-CD3+
7853
SAP+
80.0
FSC-H
Comp-PE-A :: SAP
1037024.fcs
DN
135
SAP+
79.1
FSC-H
Comp-PE-A :: SAP
1037024.fcs
ISP4
263
SAP+
59.4
FSC-H
Comp-PE-A :: SAP
1037024.fcs
DPe
7073

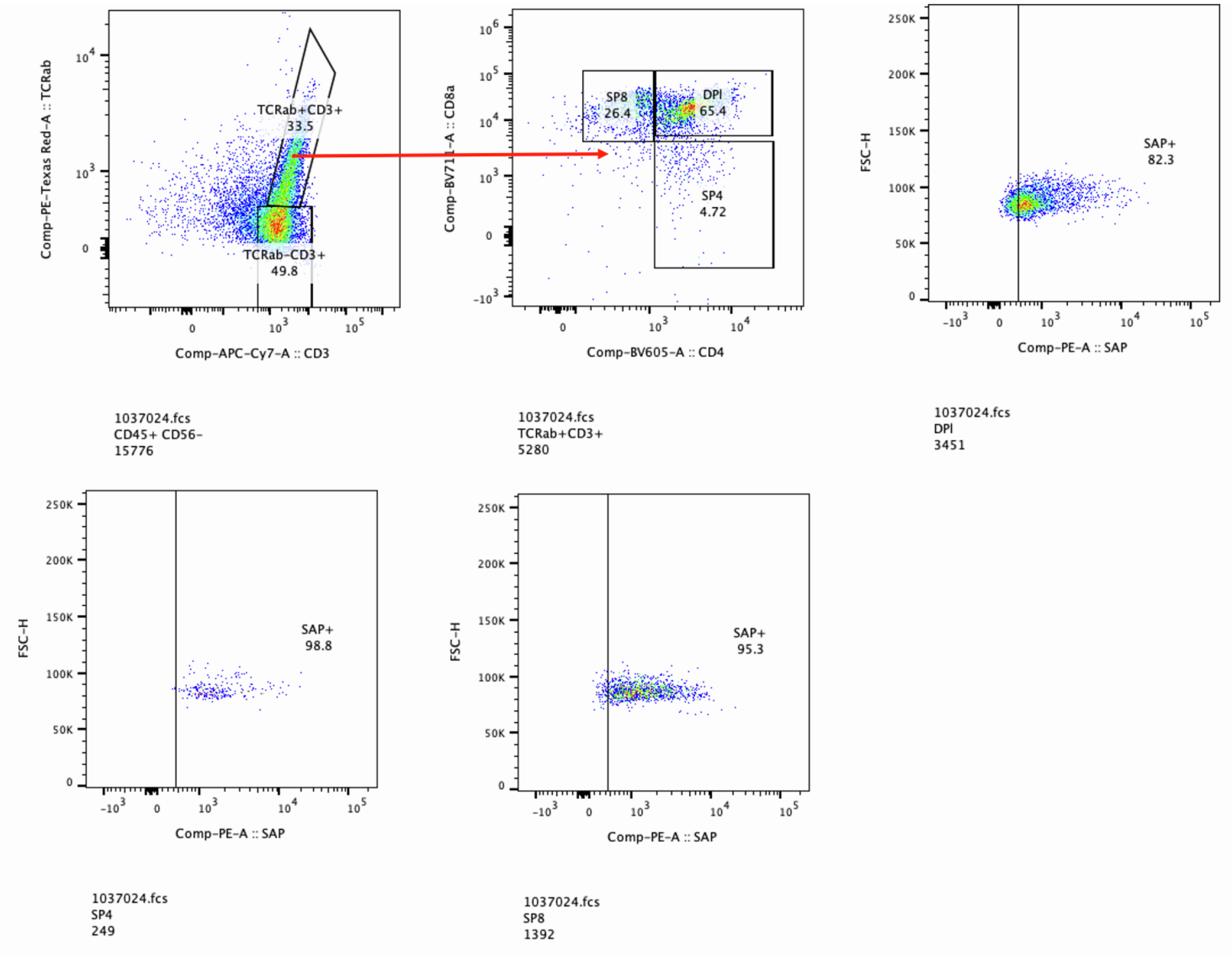


**ATO Flow Cytometry SAP and mCitrine staining across differentiation.** ATOs were stained to differentiate the following stages of T cell development: double negative (DN): hTCRab-hCD3-hCD4-hCD8-; immature single positive 4 (ISP4): hTCRab-hCD3-hCD4+hCD8-; double positive (DP) early: hTCRab-hCD3-hCD4+hCD8+; DP late: hTCRab+hCD3+hCD4+hCD8+; single positive (SP) 4: hTCRab+hCD3+hCD4+hCD8-; and SP8: hTCRab+hCD3+hCD4-hCD8+. Representative flow plots are shown above for mCitrine week 3 (A), SAP week 3 (B), mCitrine week 7 (C), SAP week 7 (D), mCitrine week 12 (E), and SAP week 12 (F).

**Figure S9:**

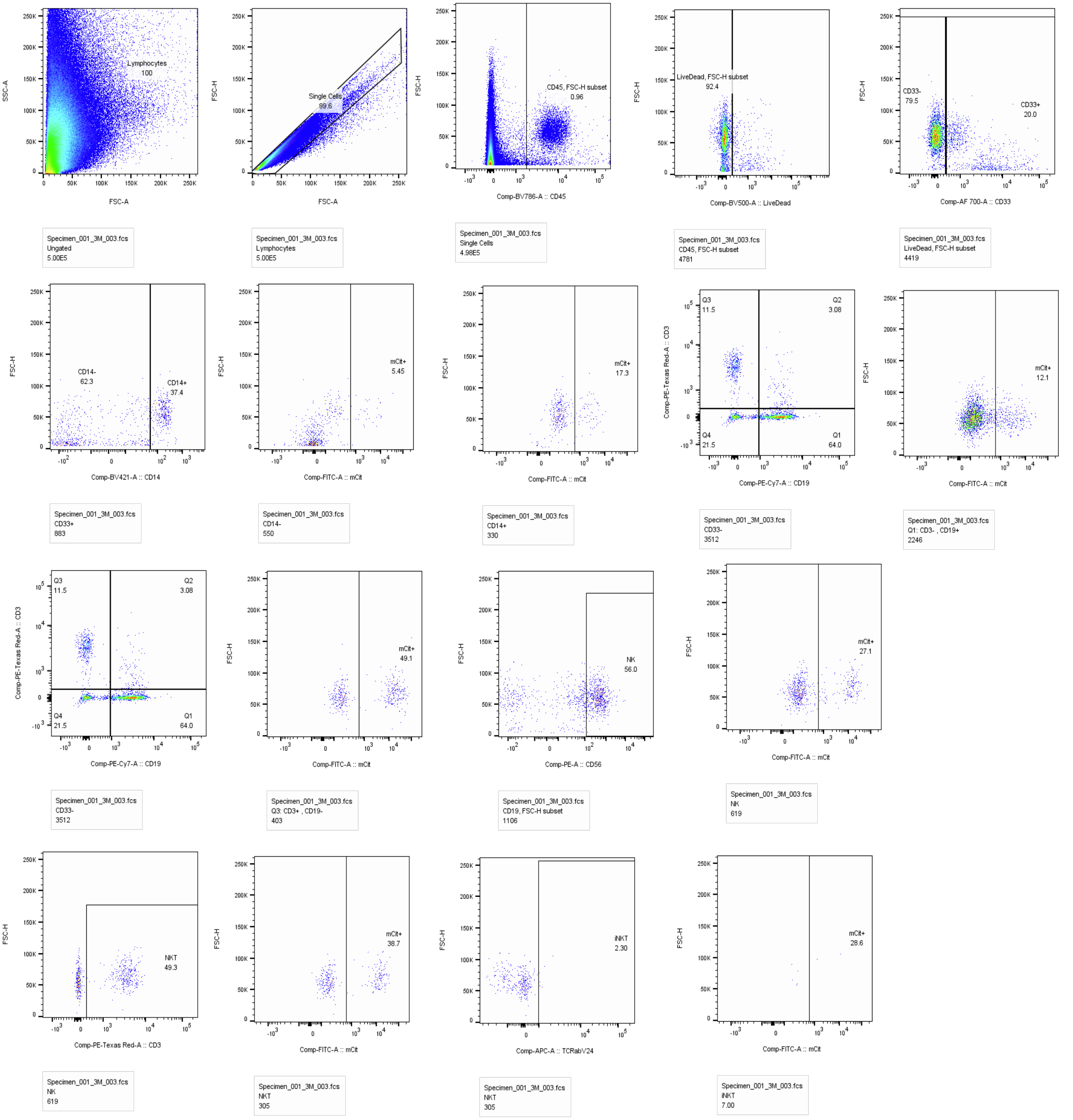


**Representative flow cytometry gating strategy of peripheral blood lineages in hIL15-NSG mice.**

**Figure S10:**

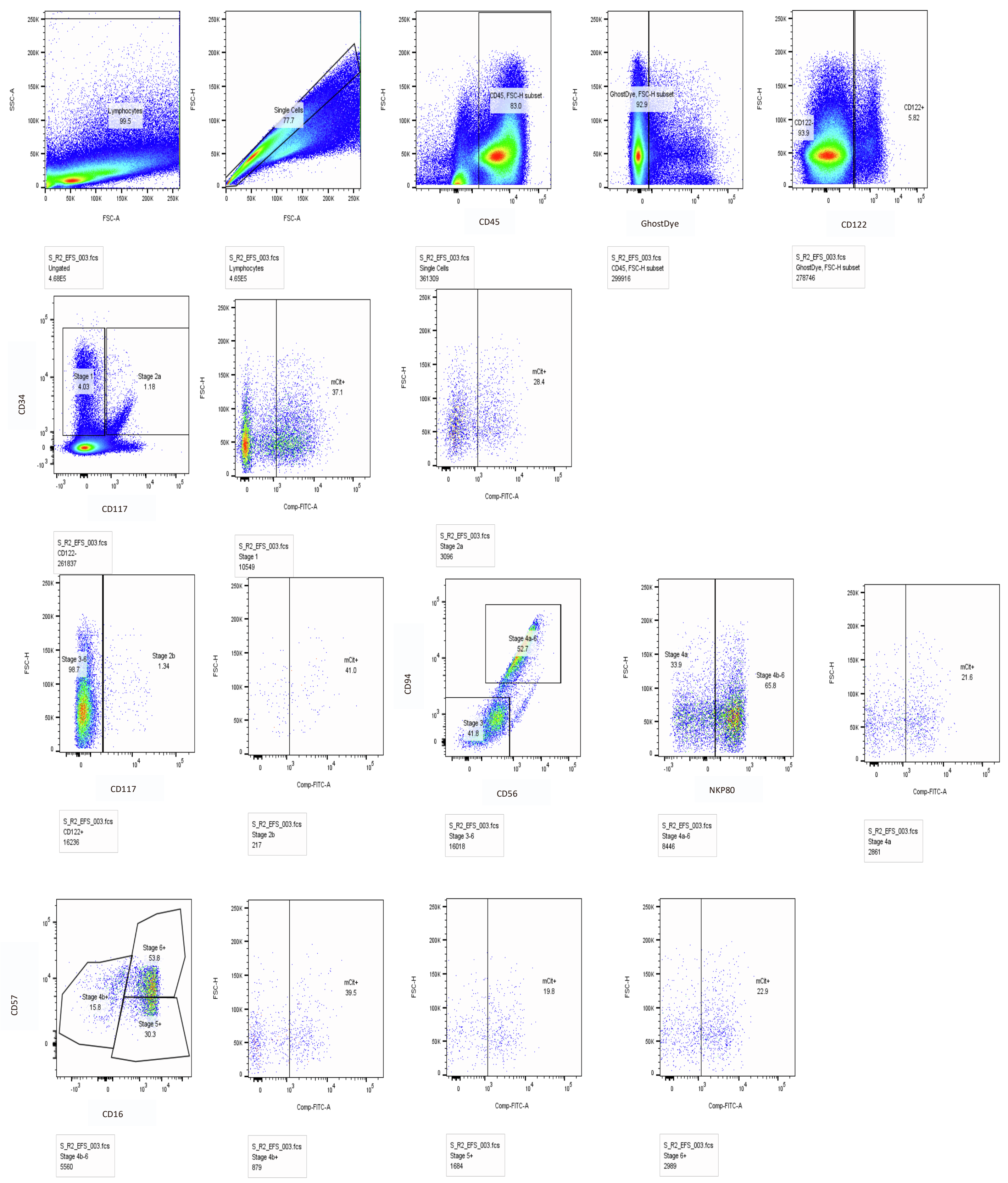


**Representative flow cytometry gating strategy of NK cells in the spleen of hIL15-NSG mice.**

**Figure S11:**

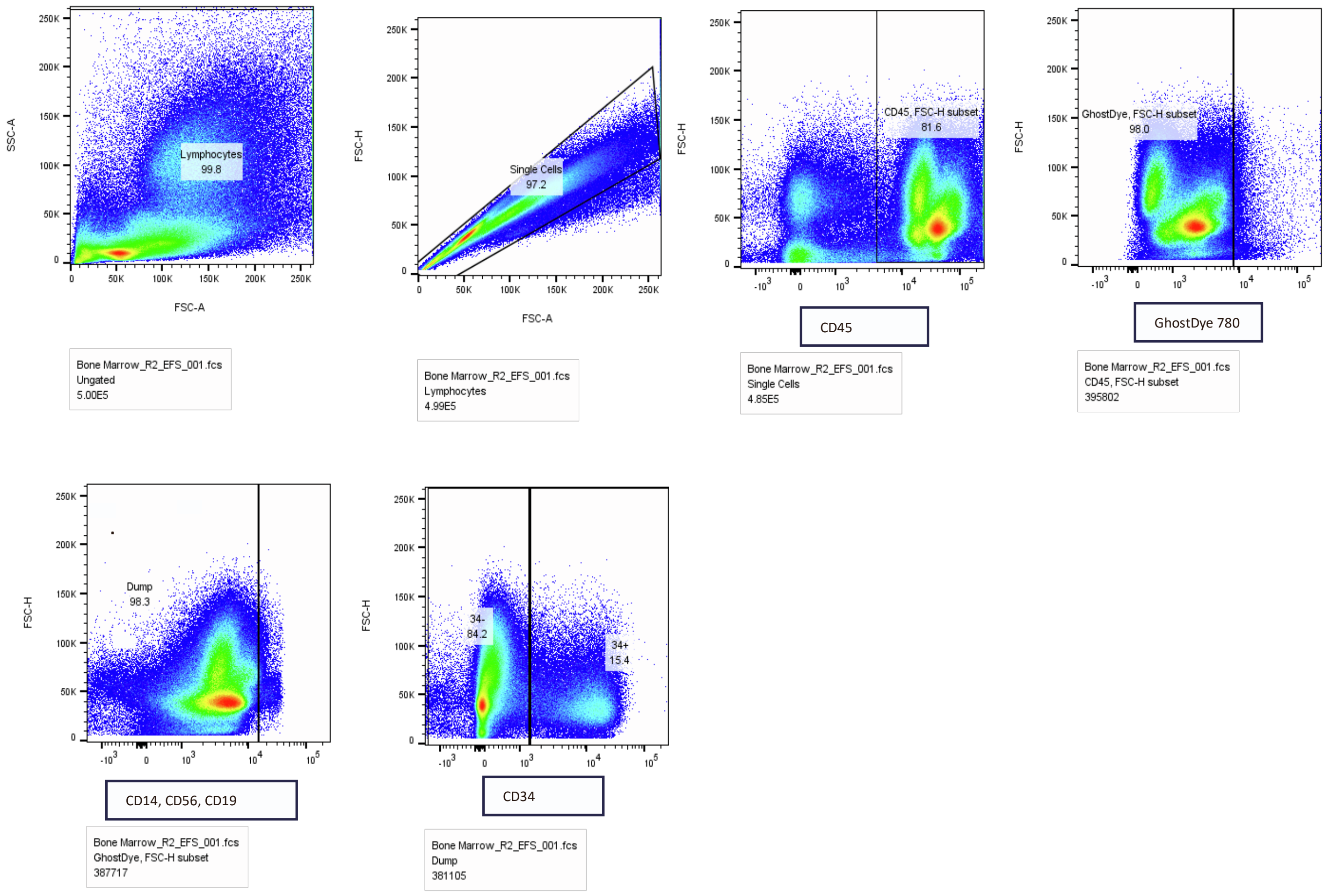
SSC-A
FSC-A
Lymphocytes
99.8
Bone Marrow_R2_EFS_001.fcs
Ungated
5.00E5
FSC-H
FSC-A
Single Cells
97.2
Bone Marrow_R2_EFS_001.fcs
Lymphocytes
4.99E5
FSC-H
CD45, FSC-H subset
81.6
CD45
Bone Marrow_R2_EFS_001.fcs
Single Cells
4.85E5
FSC-H
GhostDye, FSC-H subset
98.0
GhostDye 780
Bone Marrow_R2_EFS_001.fcs
CD45, FSC-H subset
395802
FSC-H
Dump
98.3
CD14, CD56, CD19
Bone Marrow_R2_EFS_001.fcs
GhostDye, FSC-H subset
387717
FSC-H
34-
84.2
34+
15.4
CD34
Bone Marrow_R2_EFS_001.fcs
Dump
381105

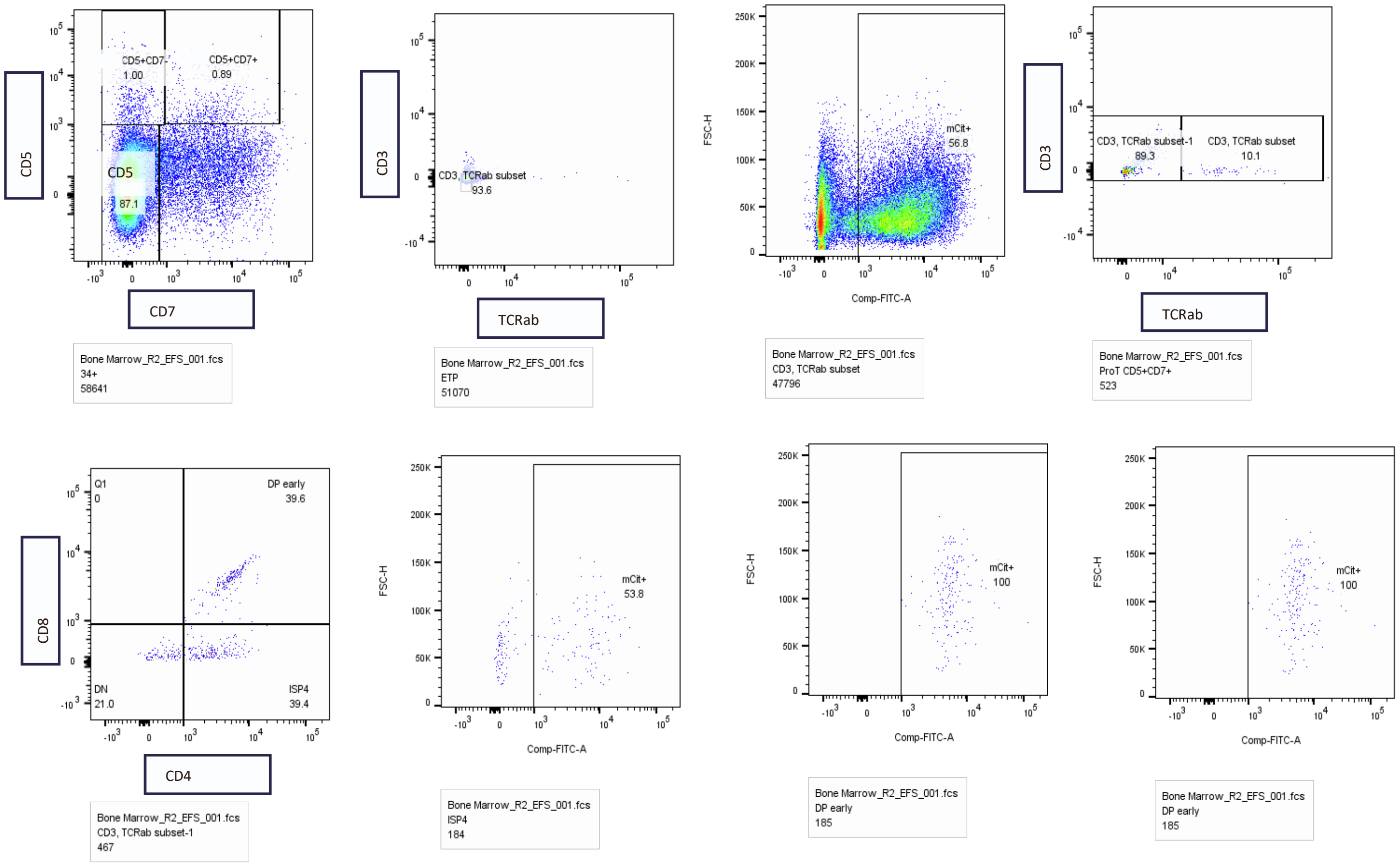
CD5
CD7
CD5+CD7-
1.00
CD5+CD7+
0.89
87.1
Bone Marrow_R2_EFS_001.fcs
34+
58641
CD3
TCRab
CD3, TCRab subset
93.6
Bone Marrow_R2_EFS_001.fcs
ETP
51070
FSC-H
Comp-FITC-A
mCit+
56.8
Bone Marrow_R2_EFS_001.fcs
CD3, TCRab subset
47796
CD3, TCRab subset-1
89.3
CD3, TCRab subset
10.1
Bone Marrow_R2_EFS_001.fcs
ProT CD5+CD7+
523
CD8
CD4
Q1
0
DP early
39.6
DN
21.0
ISP4
39.4
Bone Marrow_R2_EFS_001.fcs
CD3, TCRab subset-1
467
mCit+
53.8
Bone Marrow_R2_EFS_001.fcs
ISP4
184
mCit+
100
Bone Marrow_R2_EFS_001.fcs
DP early
185
mCit+
100
Bone Marrow_R2_EFS_001.fcs
DP early
185

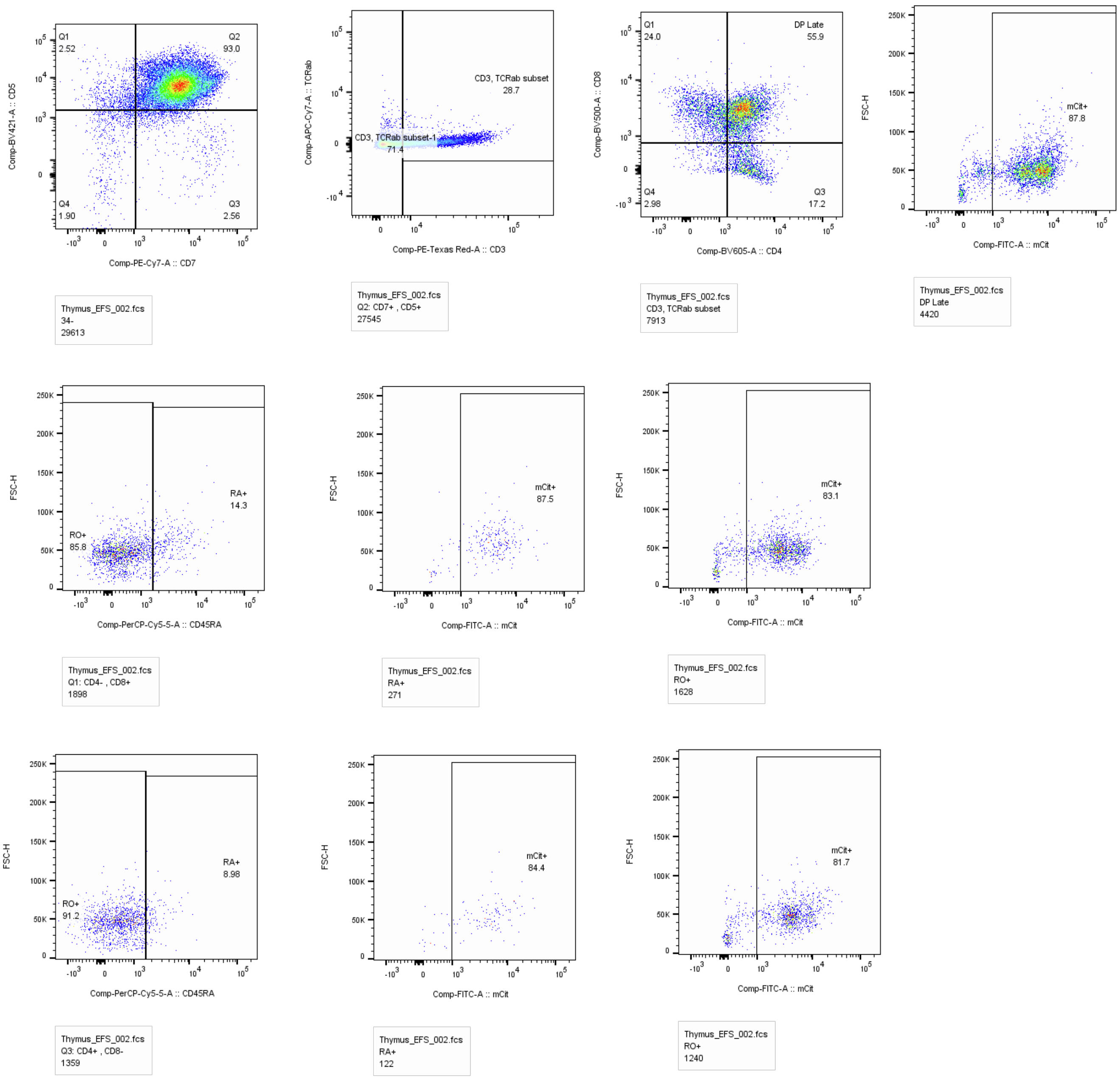


**Representative flow cytometry gating strategy of T cells in the bone marrow and thymus of hIL15-NSG mice.**

**Figure S12:**

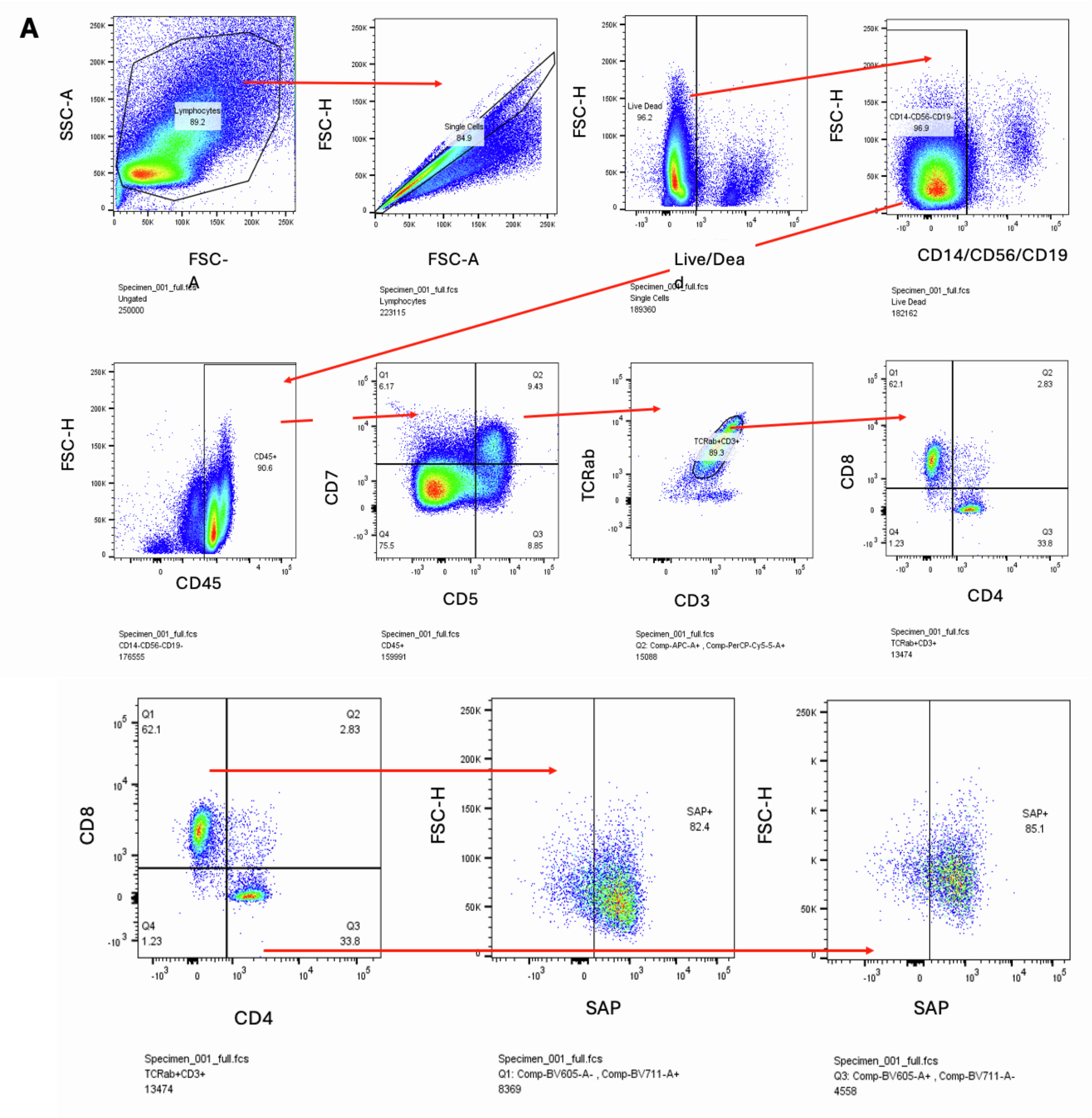
A
SSC-A
FSC-A
Lymphocytes 89.2
Specimen_001_full.fcs
Ungated
250000
FSC-H
FSC-A
Single Cells 84.9
Specimen_001_full.fcs
Lymphocytes
223115
FSC-H
Live/Dead
Live Dead 96.2
Specimen_001_full.fcs
Single Cells
189360
FSC-H
CD14/CD56/CD19
CD14-CD56-CD19- 96.9
Specimen_001_full.fcs
Live Dead
182162
FSC-H
CD45
CD45+ 90.6
Specimen_001_full.fcs
CD14-CD56-CD19-
176555
CD7
CD5
Q1 6.17
Q2 9.43
Q4 75.5
Q3 8.85
Specimen_001_full.fcs
CD45+
159991
TCRab
CD3
TCRab+CD3+ 89.3
Specimen_001_full.fcs
Q2: Comp-APC-A+ , Comp-PerCP-Cy5-5-A+
15088
CD8
CD4
Q1 62.1
Q2 2.83
Q4 1.23
Q3 33.8
Specimen_001_full.fcs
TCRab+CD3+
13474
FSC-H
SAP
SAP+ 82.4
Specimen_001_full.fcs
Q1: Comp-BV605-A- , Comp-BV711-A+
8369
SAP+ 85.1
Specimen_001_full.fcs
Q3: Comp-BV605-A+ , Comp-BV711-A-
4558

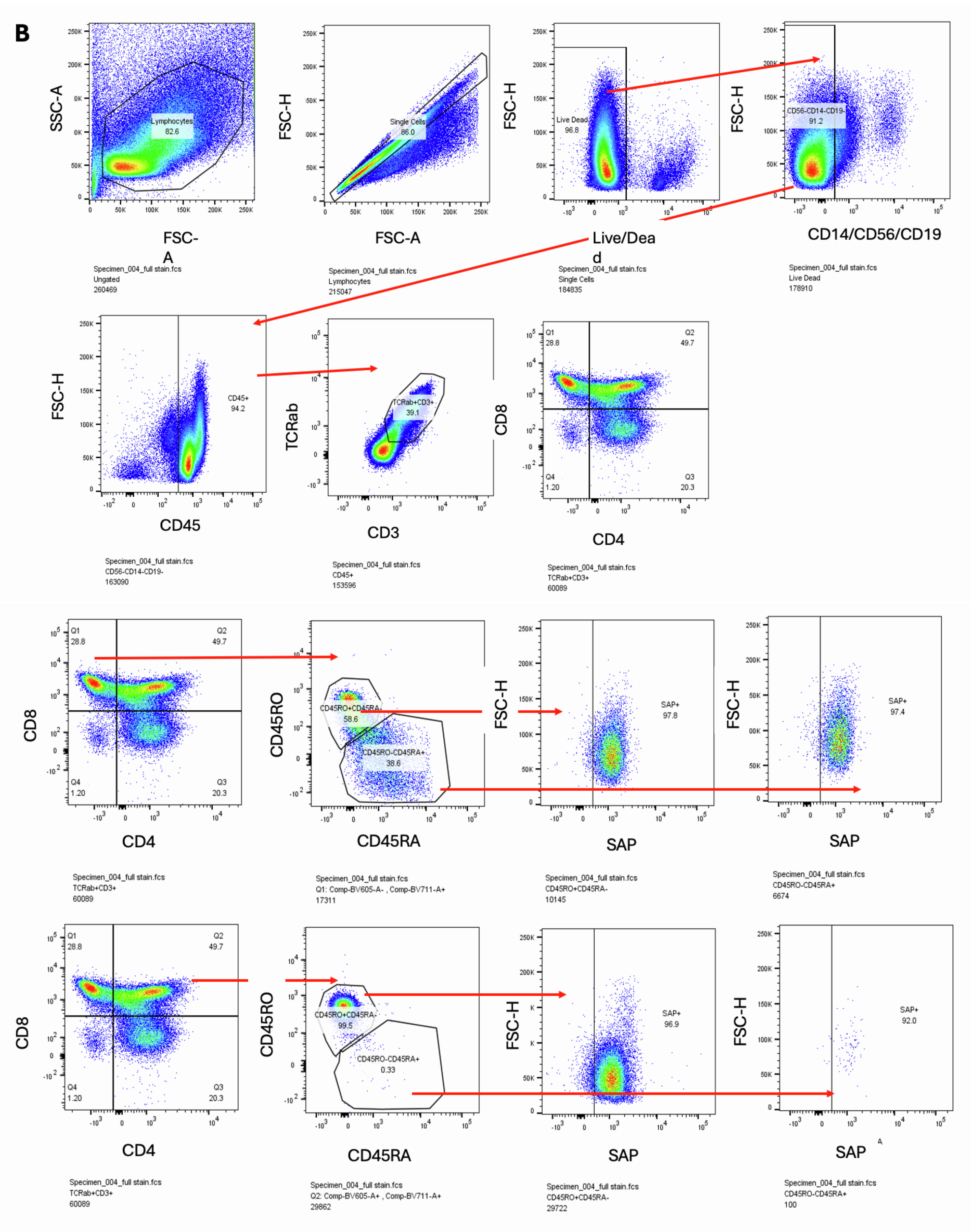
B
SSC-A
FSC-A
Lymphocytes
82.6
Specimen_004_full stain.fcs
Ungated
260469
FSC-H
FSC-A
Single Cells
86.0
Specimen_004_full stain.fcs
Lymphocytes
215047
FSC-H
Live/Dead
Live Dead
96.8
Specimen_004_full stain.fcs
Single Cells
184835
FSC-H
CD14/CD56/CD19
CD56-CD14-CD19-
91.2
Specimen_004_full stain.fcs
Live Dead
178910
FSC-H
CD45
CD45+
94.2
Specimen_004_full stain.fcs
CD56-CD14-CD19-
163090
TCRab
CD3
TCRab+CD3+
39.1
Specimen_004_full stain.fcs
CD45+
153596
CD8
CD4
Q1 28.8
Q2 49.7
Q4 1.20
Q3 20.3
Specimen_004_full stain.fcs
TCRab+CD3+
60089
CD8
CD4
Q1 28.8
Q2 49.7
Q4 1.20
Q3 20.3
Specimen_004_full stain.fcs
TCRab+CD3+
60089
CD45RO
CD45RA
CD45RO+CD45RA-
58.6
CD45RO-CD45RA+
38.6
Specimen_004_full stain.fcs
Q1: Comp-BV605-A- , Comp-BV711-A+
17311
FSC-H
SAP
SAP+
97.8
Specimen_004_full stain.fcs
CD45RO+CD45RA-
10145
FSC-H
SAP
SAP+
97.4
Specimen_004_full stain.fcs
CD45RO-CD45RA+
6674
CD8
CD4
Q1 28.8
Q2 49.7
Q4 1.20
Q3 20.3
Specimen_004_full stain.fcs
TCRab+CD3+
60089
CD45RO
CD45RA
CD45RO+CD45RA-
99.5
CD45RO-CD45RA+
0.33
Specimen_004_full stain.fcs
Q2: Comp-BV605-A+ , Comp-BV711-A+
29862
FSC-H
SAP
SAP+
96.9
Specimen_004_full stain.fcs
CD45RO+CD45RA-
29722
FSC-H
SAP
SAP+
92.0
Specimen_004_full stain.fcs
CD45RO-CD45RA+
100

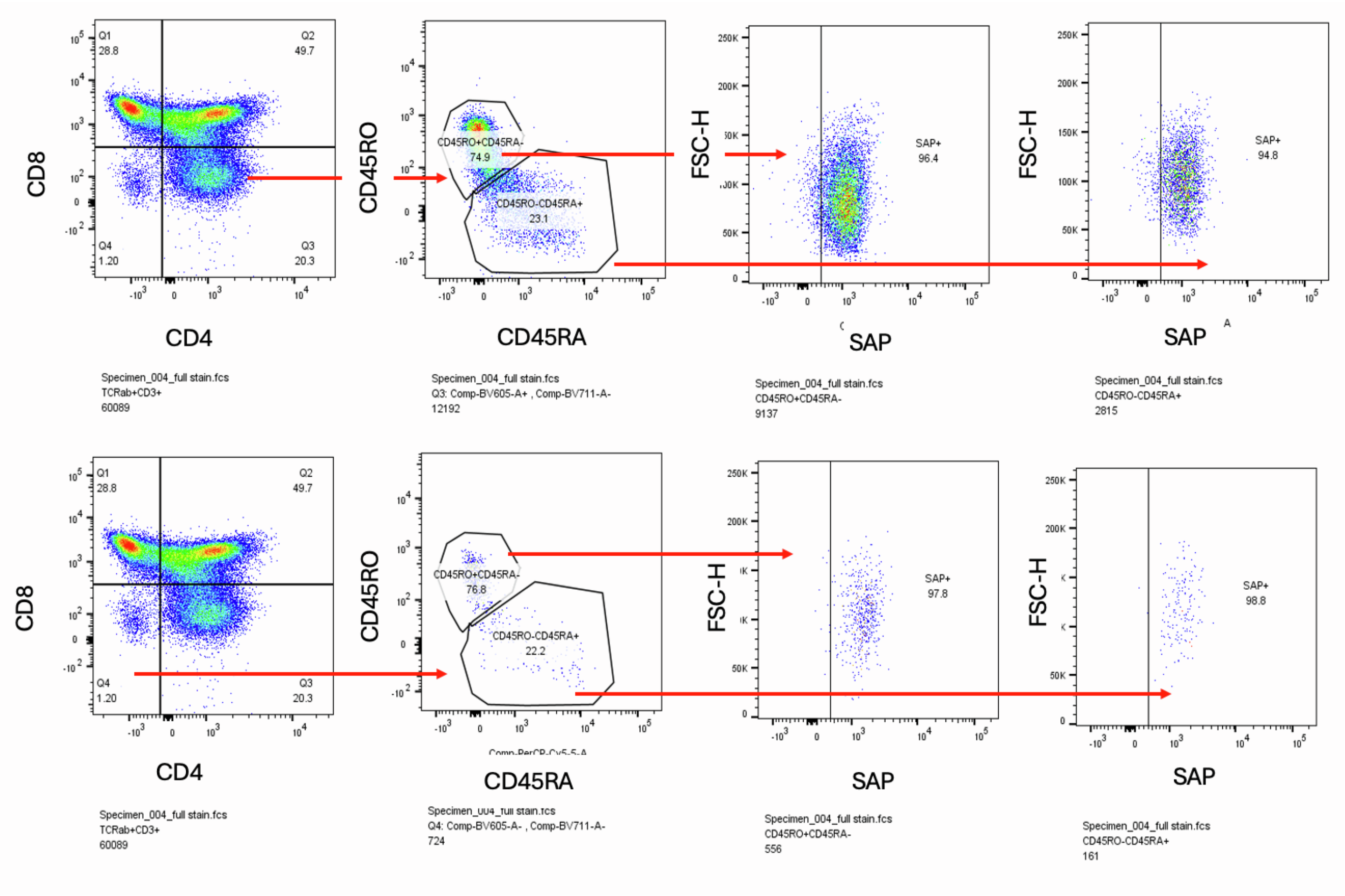


**Representative flow gating strategy of T cell populations in a human thymus as described in Figure S3.** Panel A illustrates flow gating strategy for human thymus within early development. Panel B illustrates the flow gating strategy for human thymus within mature development.

**Figure S13:**

**A**

| | | | | NKPs | Immature NKs | | Mature NKs | | | | |
|---|---|---|---|---|---|---|---|---|---|---|---|
| | | | | NKP | Pre-NK cell | | iNK cell | CD56bright NK Cell | | CD56dim NK Cell | |
| **Markers** | **Colors** | **Laser** | **Filter** | **Stage 1** | **Stage 2a** | **Stage 2b** | **Stage 3** | **Stage 4a** | **Stage 4b** | **Stage 5** | **Stage 6** |
| **CD34** | PE-Cy7 | Yellow | 780/60 | + | + | + | - | - | - | - | - |
| **CD117** | FITC | Blue | 530/30 | - | + | + | + | + (low) | - | - | - |
| **CD122** | BV650 | Violet | 610/20 | - | - | + | + | + | + | + | + |
| **CD56** | APC-Cy7 | Red | 780/60 | - | - | - (low) | - (low) | + (bright) | + (bright) | + (dim) | + (dim) |
| **CD94** | PE-Cy7 | Yellow | 575/26 | - | - | - | - | + | + | (-/+) | (-/+) |
| **NKp80** | APC-Cy7 | Red | 660/20 | - | - | - | - | - | + | + | + |
| **CD16** | BV421 | Bviolet | 450/40 | - | - | - | - | - | - | + | + |
| **CD57** | PerCP-Cy5.5 | Blue | 695/40 | - | - | - | - | - | - | - | + |
| **Ghost Dye** | UV | UV | 450/50 | | | | | | | | |

**B**

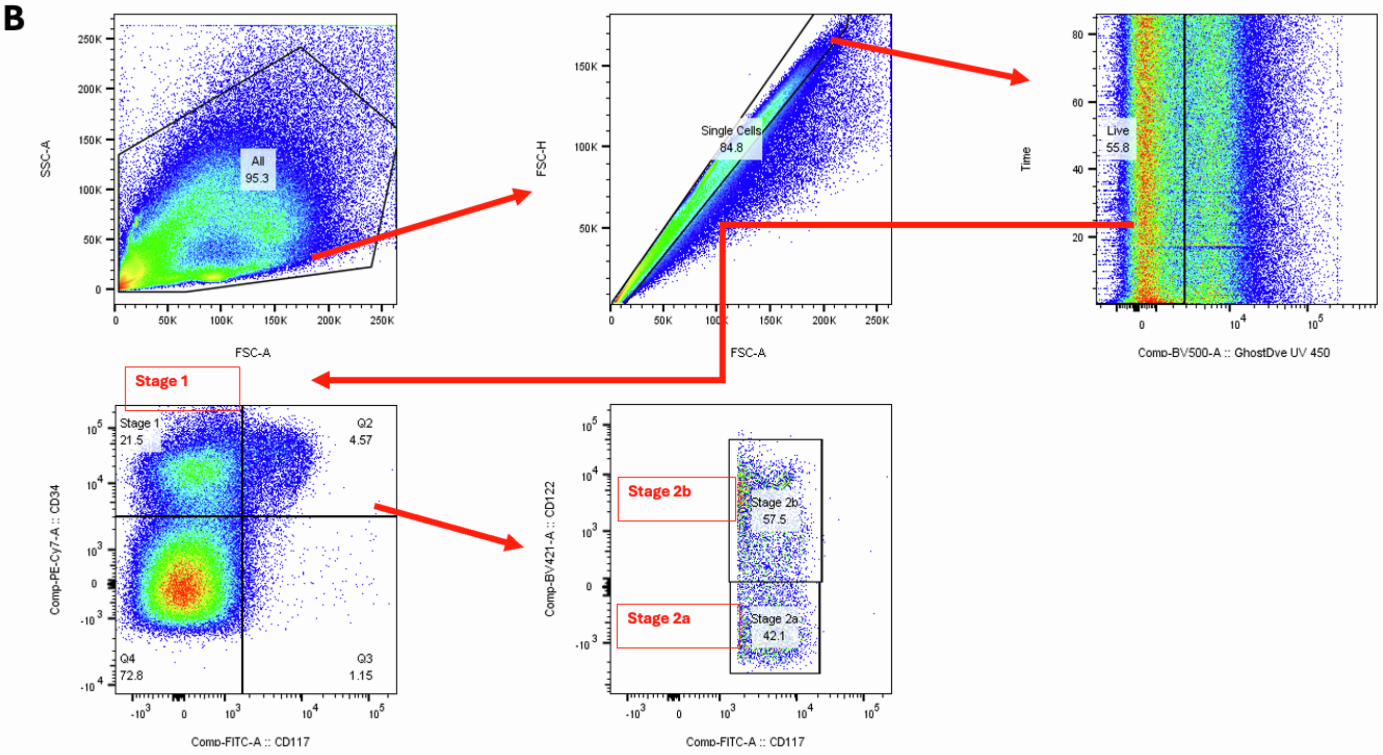

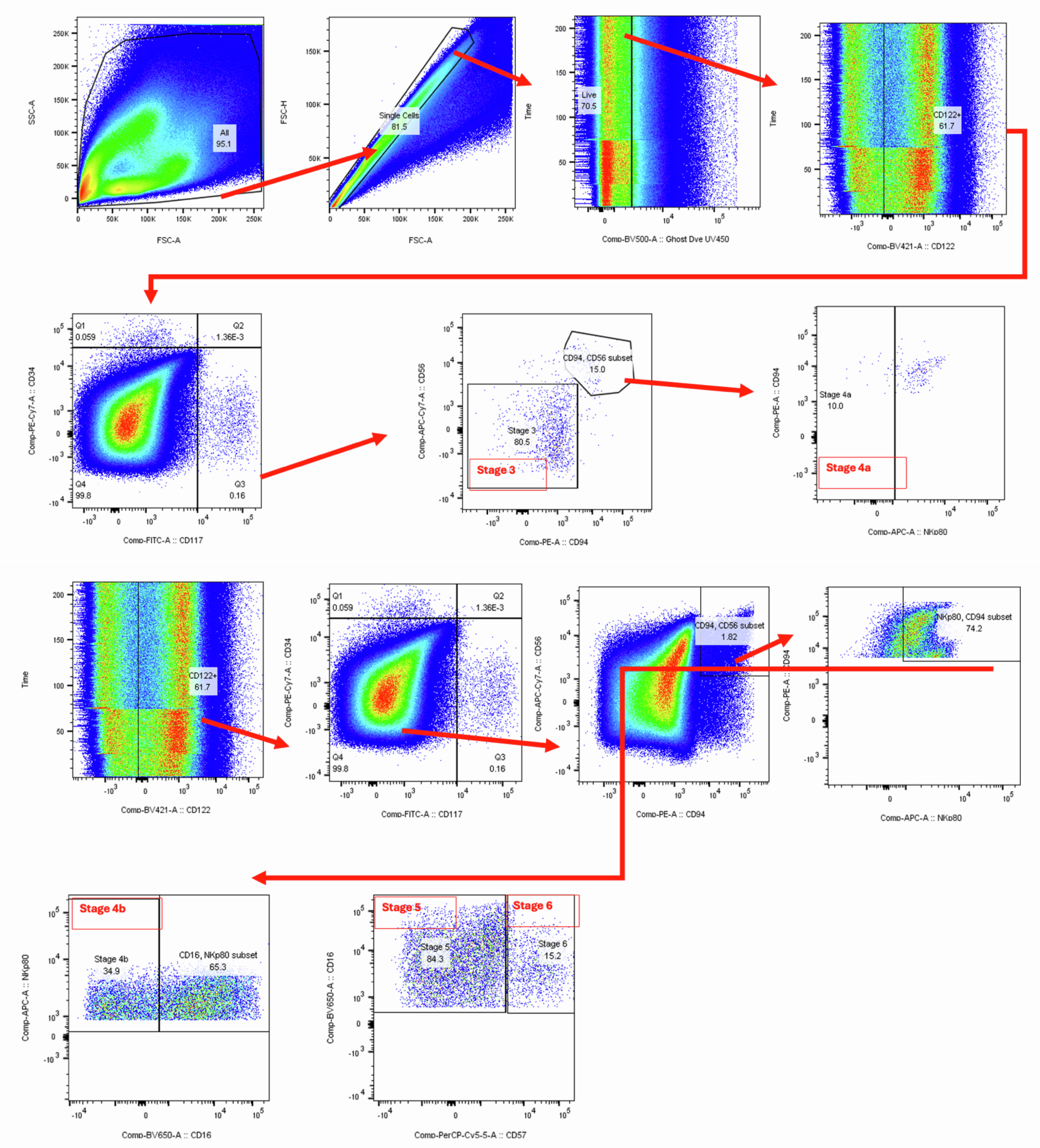


**Representative flow gating strategy of NK cell populations in human peripheral blood as described in Figure S3.** Panel A illustrates the flow panel used to differentiate NK developmental markers across development. Panel B illustrates the flow gating strategy for human NK cells across development in peripheral blood.

**Figure S14:**

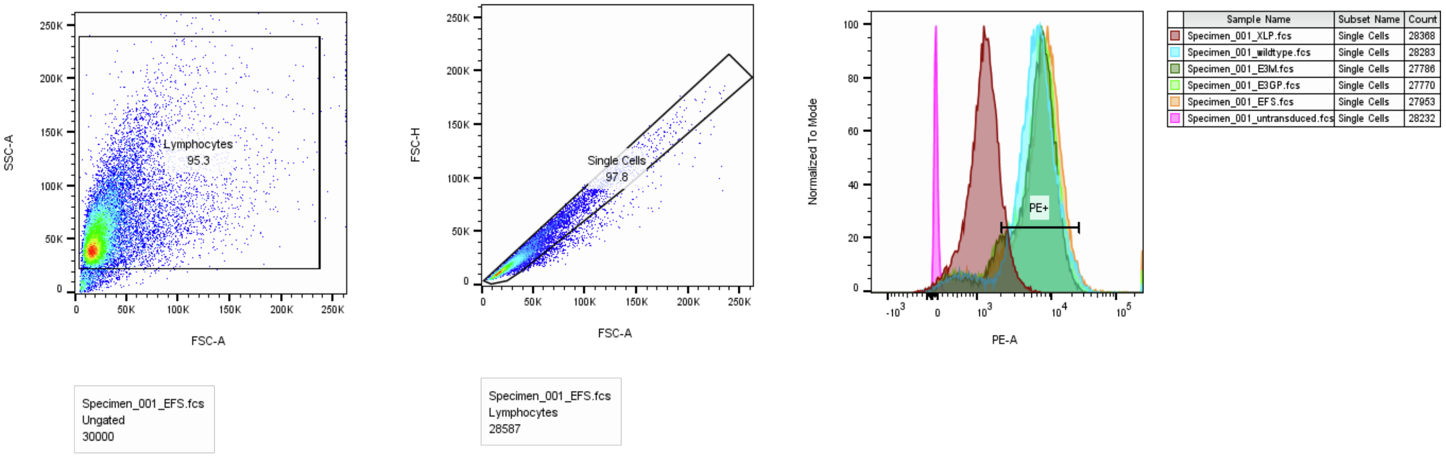


**Flow Cytometry gating strategy of SAP Expression of XLP1 Patient T cells transduced with XLP1-SMART LVs as described in Figure 5 panel A.**

**Figure S15:**

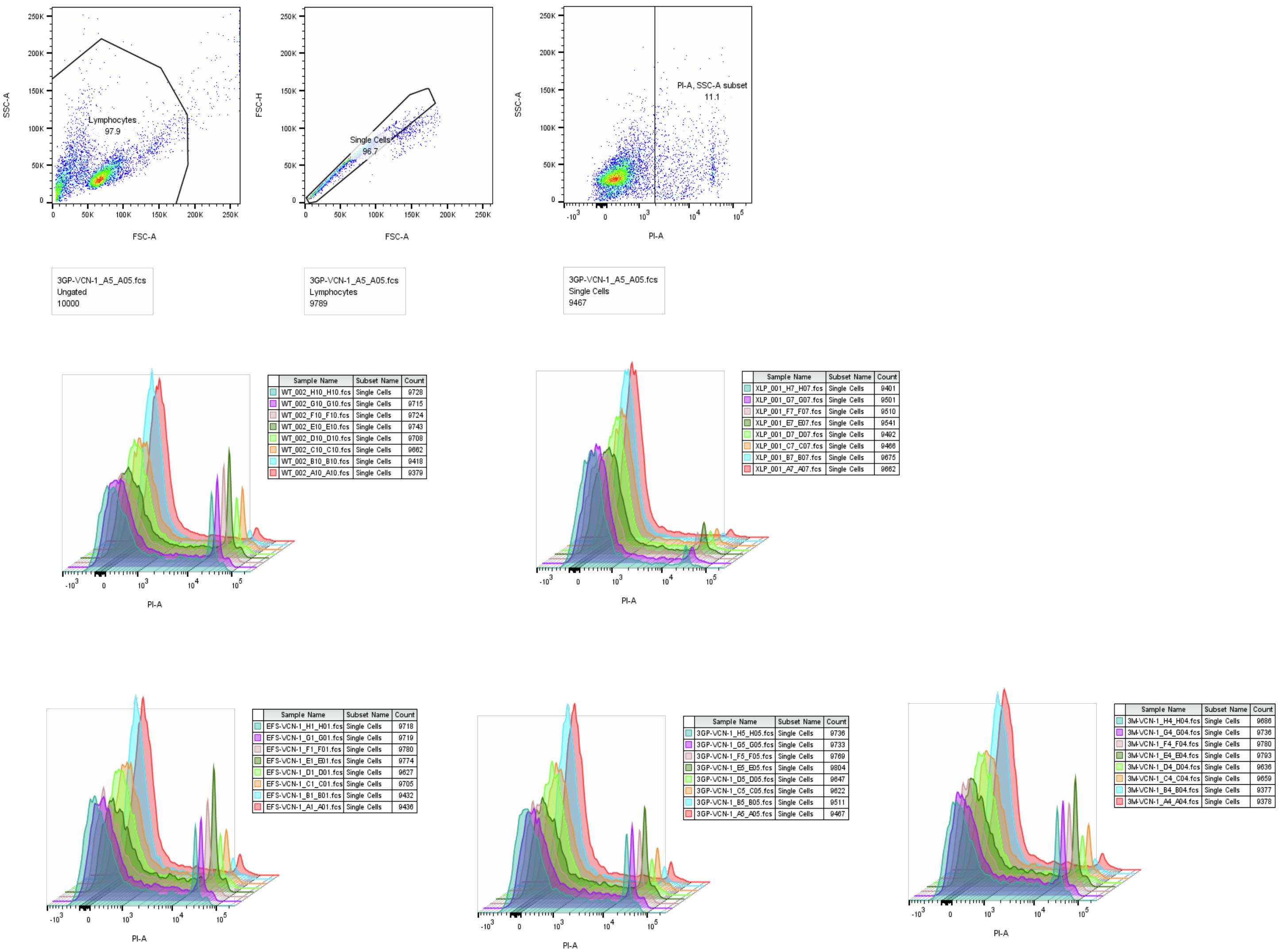


**Flow cytometry gating strategy of T Cell RICD as described in Figure 5 panel B.**

**Figure S16:**

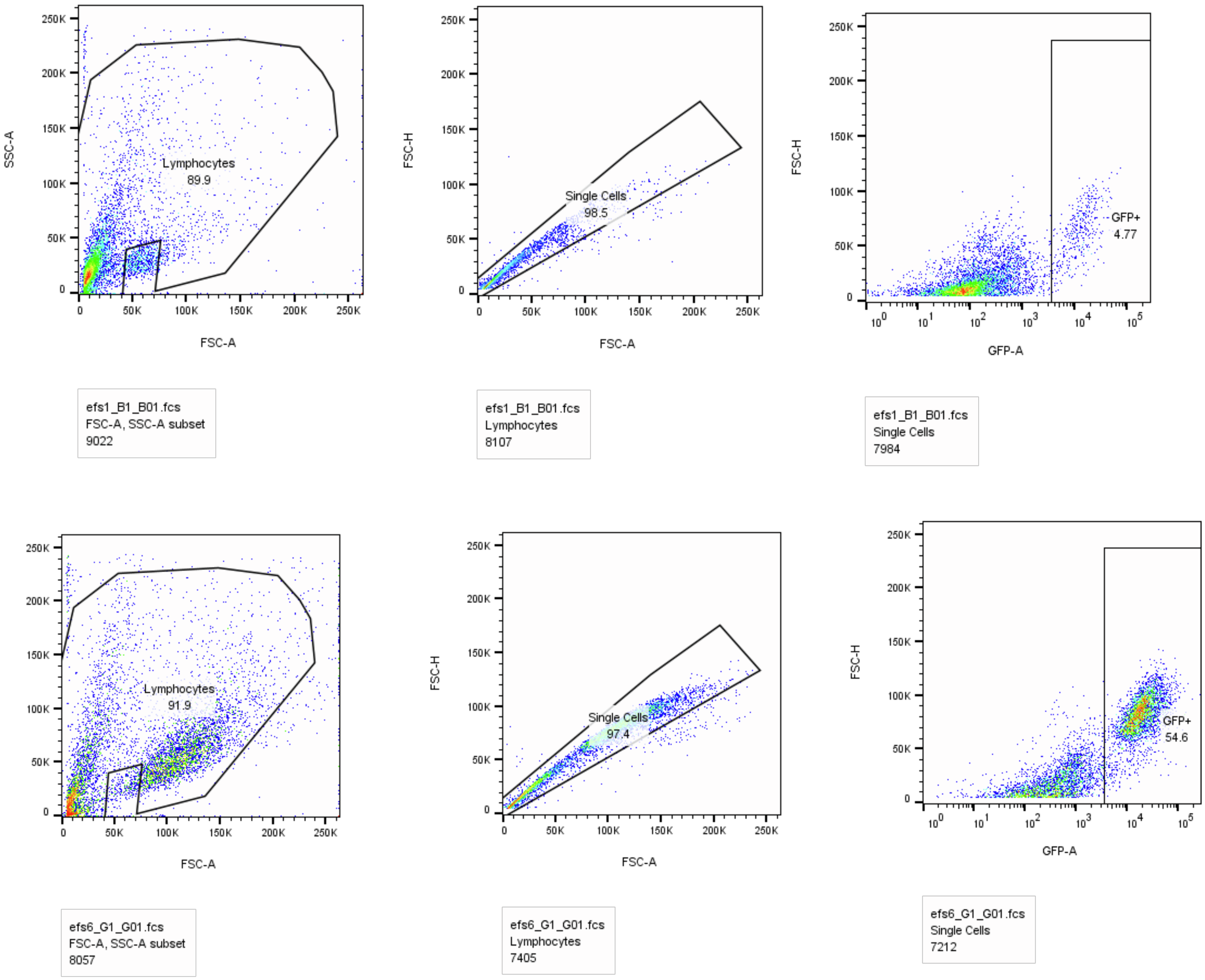

Lymphocytes
89.9
SSC-A
FSC-A
efs1_B1_B01.fcs
FSC-A, SSC-A subset
9022
Single Cells
98.5
FSC-H
FSC-A
efs1_B1_B01.fcs
Lymphocytes
8107
GFP+
4.77
FSC-H
GFP-A
efs1_B1_B01.fcs
Single Cells
7984
Lymphocytes
91.9
FSC-A
efs6_G1_G01.fcs
FSC-A, SSC-A subset
8057
Single Cells
97.4
FSC-H
FSC-A
efs6_G1_G01.fcs
Lymphocytes
7405
GFP+
54.6
FSC-H
GFP-A
efs6_G1_G01.fcs
Single Cells
7212

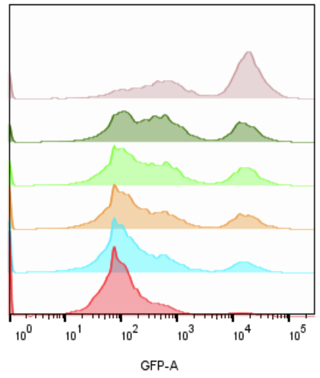


| | Sample Name | Subset Name | Count |
|---|---|---|---|
| | efs6_G1_G01.fcs | Single Cells | 7212 |
| | efs5_F1_F01.fcs | Single Cells | 7535 |
| | efs4_E1_E01.fcs | Single Cells | 7967 |
| | efs3_D1_D01.fcs | Single Cells | 7435 |
| | efs2_C1_C01.fcs | Single Cells | 7783 |
| | efs1_B1_B01.fcs | Single Cells | 7984 |

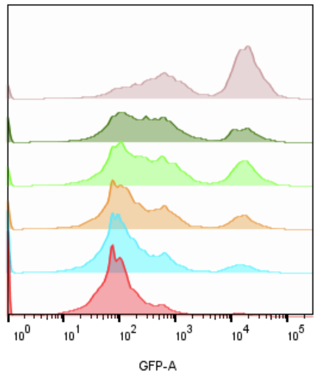


| | Sample Name | Subset Name | Count |
|---|---|---|---|
| | e3gp6_G7_G07.fcs | Single Cells | 7696 |
| | e3gp5_F7_F07.fcs | Single Cells | 6247 |
| | e3gp4_E7_E07.fcs | Single Cells | 8297 |
| | e3gp3_D7_D07.fcs | Single Cells | 7081 |
| | e3gp2_C7_C07.fcs | Single Cells | 7376 |
| | e3gp1_B7_B07.fcs | Single Cells | 6969 |

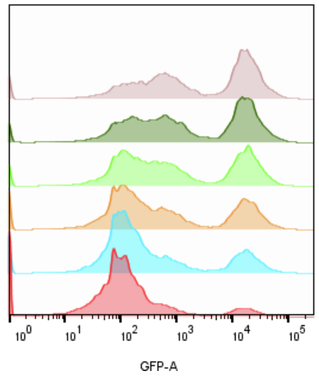


| | Sample Name | Subset Name | Count |
|---|---|---|---|
| | wt6_G1_G01.fcs | Single Cells | 7879 |
| | wt5_F1_F01.fcs | Single Cells | 8632 |
| | wt4_E1_E01.fcs | Single Cells | 8616 |
| | wt3_D1_D01.fcs | Single Cells | 8652 |
| | wt2_C1_C01.fcs | Single Cells | 8914 |
| | wt1_B3_B03.fcs | Single Cells | 8466 |

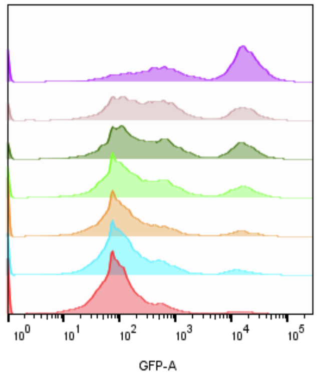


| | Sample Name | Subset Name | Count |
|---|---|---|---|
| | xlp6_G11_G11.fcs | Single Cells | 7158 |
| | xlp5_F10_F10.fcs | Single Cells | 7211 |
| | xlp4_E10_E10.fcs | Single Cells | 8217 |
| | xlp3_D11_D11.fcs | Single Cells | 8346 |
| | xlp2_C12_C12.fcs | Single Cells | 6890 |
| | xlp2_C10_C10.fcs | Single Cells | 8401 |
| | xlp1_B10_B10.fcs | Single Cells | 8340 |

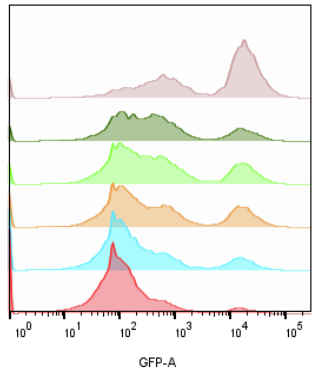


| | Sample Name | Subset Name | Count |
|---|---|---|---|
| | e3m6_G4_G04.fcs | Single Cells | 7788 |
| | e3m5_F4_F04.fcs | Single Cells | 6136 |
| | e3m4_E4_E04.fcs | Single Cells | 8315 |
| | e3m3_D4_D04.fcs | Single Cells | 7820 |
| | e3m2_C4_C04.fcs | Single Cells | 7678 |
| | e3m1_B4_B04.fcs | Single Cells | 7858 |

**Flow Cytometry gating strategy of for the NK cell cytotoxicity assay using K562s as described in Figure 5 panel C.**

**Figure S17:**

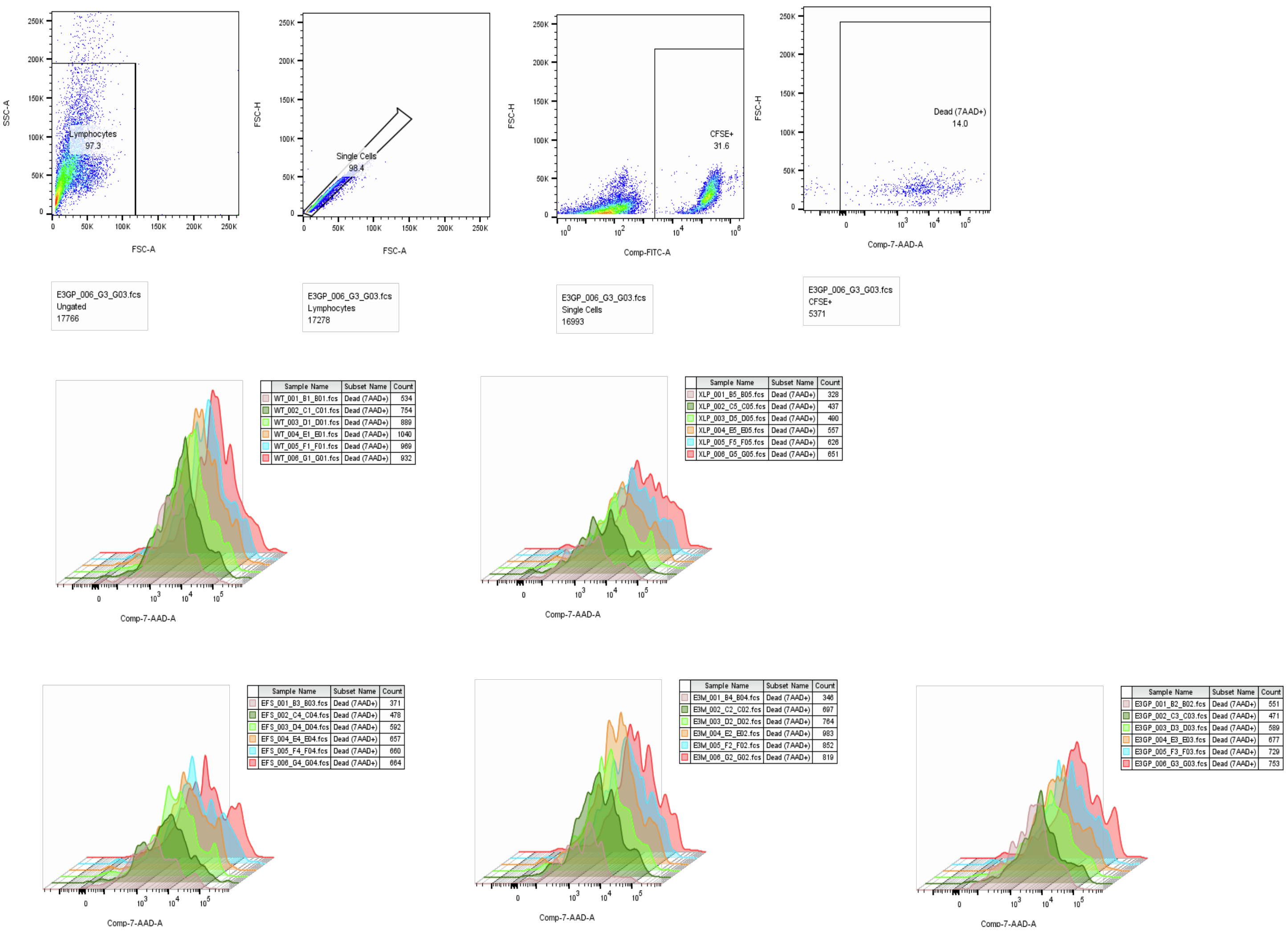


**Flow Cytometry gating strategy of for the NK cell cytotoxicity assay using K562s as described in Figure 5 panel D.**

**Table S1: Enhancer Sequences**

See Supplemental Table

**Table S2: The wildtype (WT) or codon optimized (GeneArt or JCAT) DNA sequence of SH2D1A.**

| Codon optimization | Sequence |
|---|---|
| WT | ATGGACGCAGTGGCTGTGTATCATGGCAAAATCAGCAGGGAAACCGGCGAGAAGCTCCTGCTTG<br>CCACTGGGCTGGATGGCAGCTATTTGCTGAGGGACAGCGAGAGCGTGCCAGGCGTGTACTGCC<br>TATGTGTGCTGTATCACGGTTACATTTATACATACCGAGTGTCCCAGACAGAAACAGGTTCTTGGA<br>GTGCTGAGACAGCACCTGGGGTACATAAAAGATATTTCCGGAAAATAAAAAATCTCATTTCAGCAT<br>TTCAGAAGCCAGATCAAGGCATTGTAATACCTCTGCAGTATCCAGTTGAGAAGAAGTCCTCAGCT<br>AGAAGTACACAAGGTACTACAGGGATAAGAGAAGATCCTGATGTCTGCCTGAAAGCCCCATGA |
| GeneArt | ATGGATGCCGTGGCCGTGTATCACGGCAAGATCAGCAGAGAGACAGGCGAGAAACTGCTGCTG<br>GCCACAGGCCTGGATGGCAGCTATCTGCTGAGAGACTCTGAGAGCGTGCCCGGCGTGTACTGT<br>CTGTGTGTGCTGTACCACGGCTACATCTACACCTACCGGGTGTCCCAGACCGAGACAGGATCTT<br>GGAGCGCCGAAACAGCTCCTGGCGTGCACAAGCGGTACTTCAGAAAGATCAAGAACCTGATCAG<br>CGCCTTCCAGAAGCCTGACCAGGGCATCGTGATCCCTCTGCAGTACCCCGTGGAAAAGAAGTCC<br>AGCGCCAGAAGCACCCAGGGCACCACAGGCATCAGAGAAGATCCCGACGTGTGCCTGAAGGCC<br>CCTTGA |
| JCAT | ATGGACGCCGTGGCCGTGTACCACGGCAAGATCAGCCGCGAGACCGGCGAGAAGCTGCTGCTG<br>GCCACCGGCCTGGACGGCAGCTACCTGCTGCGCGACAGCGAGAGCGTGCCCGGCGTGTACTG<br>CCTGTGCGTGCTGTACCACGGCTACATCTACACCTACCGCGTGAGCCAGACCGAGACCGGCAGC<br>TGGAGCGCCGAGACCGCCCCCGGCGTGCACAAGCGCTACTTCCGCAAGATCAAGAACCTGATC<br>AGCGCCTTCCAGAAGCCCGACCAGGGCATCGTGATCCCCCTGCAGTACCCCGTGGAGAAGAAG<br>AGCAGCGCCCGCAGCACCCAGGGCACCACCGGCATCCGCGAGGACCCCGACGTGTGCCTGAA<br>GGCCCCCTGA |

**Table S3 SH2D1A -/- Jurkat Western Blot Densitometry:**

| Lane | Condition | Control | Target | T/C Ratio | % of WT |
|---|---|---|---|---|---|
| 1 | WT Jurkat | 15322.255 | 10810.104 | 0.706 | 100% |
| 2 | SH2D1A-/-  Jurkat | 13984.669 | 423.698 | 0.030 | 4% |
| 3 | E3M VCN 1 | 14951.69 | 239.92 | 0.016 | 2% |
| 4 | E3M VCN 3 | 11348.619 | 1804.134 | 0.159 | 23% |
| 5 | E3M VCN 5 | 10183.104 | 2436.154 | 0.239 | 34% |
| 6 | E3GP VCN 1 | 7034.447 | 244.213 | 0.035 | 5% |
| 7 | E3GP VCN 3 | 16223.619 | 1200.234 | 0.074 | 10% |
| 8 | E3GP VCN 5 | 13031.912 | 2857.054 | 0.219 | 31% |

**Table S4: ddPCR Primer and Probe sets for detecting vector copy number of lentiviral vectors.**

| Name | Sequence | Modification |
|---|---|---|
| SDC4 Probe | CCCACCGAACCCAAGAAACTAGAGGAGAAT | HEX |
| PSI Probe | Ccctcagacccttttagtcagtgtggaaaatctctag | FAM |
| SDC4 Primer F | CAGGGTCTGGGAGCCAAGT | - |
| SDC4 Primer R | GCACAGTGCTGGACATTGACA | - |
| PSI Primer F | cactcccaacgaagacaaga | - |
| PSI Primer R | cctctggtttccctttcgct | - |

**Table S5: sgRNA sequences for knocking out *SH2D1A* in Jurkat and T cells to create SAP deficient cells.**

| **Name** | **Sequence** |
|---|---|
| XLP-sgRNA4-SH2D1Ako | GACGCAGTGGCTGTGTATCA |
| XLP-sgRNA7-SH2D1Ako | AACAGGTTCTTGGAGTGCTG |

**Table S6: Antibodies**

| Marker | Color | Website |
|---|---|---|
| SAP | PE | https://www.thermofisher.com/antibody/product/SAP-SLAM-Associated-Protein-Antibody-clone-XLP-1D12-Monoclonal/12-9787-42 |
| CD33 | BV711 | https://www.bdbiosciences.com/eu/applications/research/stem-cell-research/hematopoietic-stem-cell-markers/human/negative-markers/bv711-mouse-anti-human-cd33-wm53-also-known-as-wm-53/p/563171 |
| CD56 | FITC | https://www.biolegend.com/it-it/products/fitc-anti-human-cd56-ncam-antibody-3795 |
| CD16 | APC-CY7 | https://www.bdbiosciences.com/us/applications/research/stem-cell-research/cancer-research/human/apc-cy7-mouse-anti-human-cd16-3g8/p/557758 |
| CD19 | PE-Cy7 | https://www.bdbiosciences.com/us/applications/research/clinical-research/oncology-research/blood-cell-disorders/surface-markers/human/pe-cy7-mouse-anti-human-cd19-sj25c1/p/557835 |
| CD3 | PerCP Cy5.5 | https://www.bdbiosciences.com/us/applications/research/t-cell-immunology/th-1-cells/surface-markers/human/percp-cy55-mouse-anti-human-cd3-ucht1-also-known-as-ucht-1-ucht-1/p/560835 |
| TCR V*a* | APC | https://www.biolegend.com/en-us/products/apc-anti-human-tcr-valpha24-jalpha18-inkt-cell-antibody-6030 |
| CD14 | BV421 | https://www.bdbiosciences.com/us/reagents/research/antibodies-buffers/immunology-reagents/anti-human-antibodies/cell-surface-antigens/bv421-mouse-anti-human-cd14-m5e2/p/565283 |
| CD4 | BV605 | https://www.bdbiosciences.com/us/applications/research/t-cell-immunology/th-1-cells/surface-markers/human/bv605-mouse-anti-human-cd4-rpa-t4/p/562658 |
| CD8 | BV510 | https://www.bdbiosciences.com/us/reagents/research/antibodies-buffers/immunology-reagents/anti-non-human-primate-antibodies/cell-surface-antigens/bv510-mouse-anti-human-cd8-sk1/p/563919 |
| CD34 | Pe-Cy7 | PE/Cyanine7 anti-human CD34 Antibody anti-CD34 - 561 (biolegend.com) |
| CD117 | FITC | FITC anti-human CD117 c-kit Antibody anti-CD117 - 104D2 (biolegend.com) |
| CD122 | BV421 | BV421,Mouse,Anti-Human,CD122,Mik-β3,RUO - 562887 \| BD Biosciences-US |
| CD56 | APC-Cy7 | APC/Cyanine7 anti-human CD56 NCAM Antibody anti-CD56 (NCAM) - 5.1H11 (biolegend.com) |
| CD94 | PE | PE anti-human CD94 Antibody anti-CD94 - DX22 (biolegend.com) |
| NKp80 | AF750 | https://www.rndsystems.com/products/human-nkp80-klrf1-alexa-fluor-750-conjugated-antibody-239127_fab1900s |
| CD16 | BV650 | BV650,Mouse,Anti-Human,CD16,3G8,RUO - 563691 \| BD Biosciences-US |
| CD16 | BV510 | https://www.bdbiosciences.com/en-us/products/reagents/flow-cytometry-reagents/research-reagents/single-color-antibodies-ruo/bv510-mouse-anti-human-cd16.563830 |
| CD19 | APC | https://www.thermofisher.com/antibody/product/CD19-Antibody-clone-SJ25C1-Monoclonal/17-0198-42 |
| CD3 | PE-Texas Red | https://www.thermofisher.com/antibody/product/CD3-Antibody-clone-7D6-Monoclonal/MHCD0317 |
| CD5 | BV421 | https://biolegend.com/en-us/products/brilliant-violet-421-anti-human-cd5-antibody-15832 |
| TCRab | PECy7 | https://www.biolegend.com/en-us/products/pe-cyanine7-anti-human-tcr-alpha-beta-antibody-6731?GroupID=GROUP28 |
| CD34 | APC Cy7 | https://www.biolegend.com/en-us/search-results/apc-cyanine7-anti-human-cd34-antibody-6159?GroupID=BLG7551&gclid=Cj0KCQjw8NilBhDOARIsAHzpbLCaj4FNP3yWHPoqv4lwQTjoWuJsEJNeiplJyBfSU63E5--5OcJVLSYaAgAQEALw_wcB |

| | | |
|---|---|---|
| CD57 | PerCP Cy5.5 | https://www.biolegend.com/en-us/search-results/percp-cyanine5-5-anti-human-cd57-antibody-12127?GroupID=BLG8483&gclid=Cj0KCQjw8NilBhDOARIsAHzpbLDMTI7FbaVoxg847nDqC4lwuCx7_unezyYJZtQdAIVBNLivY84ZtrIaAi6rEALw_wcB |
| CD94 | PE Dazzle | https://www.biolegend.com/en-us/products/pe-dazzle-594-anti-human-cd94-antibody-16042 |
| CD19 | PE/Cy7 | https://www.biolegend.com/en-us/search-results/pe-cyanine7-anti-human-cd19-antibody-1911?gclid=Cj0KCQjw8NilBhDOARIsAHzpbLAEdWtO6QNY24GLob-tVN2VjUxNia9K5Zu8KJ5LXL-VIeljfa6PrfwaAv4SEALw_wcB |
| CD7 | PE/Cy7 | https://www.biolegend.com/en-us/products/pe-cyanine7-anti-human-cd7-antibody-21684 |
| CD14 | APC | https://www.biolegend.com/en-us/products/apc-anti-human-cd14-antibody-3953?GroupID=BLG4805 |
| CD56 | PE | https://www.bdbiosciences.com/en-us/products/reagents/flow-cytometry-reagents/research-reagents/single-color-antibodies-ruo/pe-mouse-anti-human-cd56.556647 |
| CD45 | BV650 | https://www.bdbiosciences.com/en-us/products/reagents/flow-cytometry-reagents/research-reagents/single-color-antibodies-ruo/bv650-mouse-anti-human-cd45.563717 |
| CD45 | APC | https://www.biolegend.com/en-us/products/apc-anti-human-cd45-antibody-705?GroupID=BLG5926 |
| CD45RA | PerCP Cy5.5 | https://www.biolegend.com/en-us/search-results/percp-cyanine5-5-anti-human-cd45ra-antibody-4241 |
| CD45RO | PE | https://www.bdbiosciences.com/en-us/products/reagents/flow-cytometry-reagents/research-reagents/single-color-antibodies-ruo/pe-mouse-anti-human-cd45ro.555493 |
| CD8a | BV711 | https://www.bdbiosciences.com/en-us/products/reagents/flow-cytometry-reagents/research-reagents/single-color-antibodies-ruo/bv711-mouse-anti-human-cd8.563677 |
| CD8b | APC | https://www.biolegend.com/fr-ch/products/apc-anti-human-cd8b-antibody-23661?GroupID=GROUP28 |
| Zombie | UV | https://www.biolegend.com/en-gb/products/zombie-uv-fixable-viability-kit-9336 |
| TCRab | PE-Dazzle | https://www.biolegend.com/en-us/products/pe-dazzle-594-anti-human-tcr-alpha-beta-antibody-12515 |
| CD56 | PE-Cy7 | https://www.biolegend.com/en-us/products/pe-cyanine7-anti-human-cd56-ncam-antibody-9959 |
| CD8b | APC | https://www.miltenyibiotec.com/US-en/products/cd8b-antibody-anti-human-reafinity-rea715.html#conjugate=apc:size=100-tests-in-200-ul |
| CD3 | APC-Cy7 | https://www.biolegend.com/en-us/products/apc-cyanine7-anti-human-cd3-antibody-3929 |
| CD45 | BV510 | https://www.biolegend.com/en-us/products/brilliant-violet-510-anti-human-cd45-antibody-8006 |
| CD4 | BV605 | https://www.biolegend.com/en-us/products/brilliant-violet-605-anti-human-cd4-antibody-10434 |
| CD8a | BV711 | https://www.biolegend.com/en-us/products/brilliant-violet-711-anti-human-cd8-antibody-10762 |
| CD45RO | BV785 | https://www.biolegend.com/en-us/products/brilliant-violet-785-anti-human-cd45ro-antibody-7973 |
| CD7 | PerCP Cy5.5 | https://www.biolegend.com/en-us/products/percp-cyanine5-5-anti-human-cd7-antibody-13918 |
| CD5 | APC | https://www.biolegend.com/en-us/products/apc-anti-human-cd5-antibody-868 |
| CD34 | APC-Cy7 | https://www.biolegend.com/en-us/products/apc-cyanine7-anti-human-cd34-antibody-12973 |
| CD3 | BV785 | https://www.biolegend.com/en-us/products/brilliant-violet-785-anti-human-cd3-antibody-14454 |
| Fcblock | | https://www.biolegend.com/en-us/products/human-trustain-fcx-fc-receptor-blocking-solution-6462 |
| DAPI | | https://www.thermofisher.com/order/catalog/product/D1306 |

**Table S7: Barcodes for next generation sequencing**

| Construct | Barcode |
|---|---|
| XLP1-E1-1-mCit-WPRE | AACAGACCGACAGGTTCTAC |
| XLP1-E1-2-mCit-WPRE | AACGTCCTTAAGGCCACTTC |
| XLP1-E2-1-mCit-WPRE | AACTACGAGGCGCAAGTGCT |
| XLP1-E2-2-mCit-WPRE | AAGTCGCATAATCCTTGGTC |
| XLP1-E3-1-mCit-WPRE | GATCTTGCGGCACCGAGATT |
| XLP1-E3-2-mCit-WPRE | GGTGCGTGTAAGAAGTTGTC |
| XLP1-E4-1-mCit-WPRE | ACCACCATAGTCACCATTGT |
| XLP1-E4-2-mCit-WPRE | ACCGAGTTAAGCCTATGCTG |
| XLP1-E5-1-mCit-WPRE | ACGAGTTACATATAGAGACC |
| XLP1-E5-2-mCit-WPRE | ACTAATCAGGTCATAAGCCT |
| XLP1-E6-1-mCit-WPRE | ACTGTGAACAACCATGTGGC |
| XLP1-E6-2-mCit-WPRE | AGCATACGAGTAGAGTTGGT |
| XLP1-E7-1-mCit-WPRE | AGGAGCATTACTGTAGATTC |
| XLP1-E7-2-mCit-WPRE | AGTGGTAGGATCCTGCACAC |
| XLP1-E8-1-mCit-WPRE | ATACACCAGAACCTAACAAC |
| XLP1-E8-2-mCit-WPRE | ATGACTCGGACCATTGCCAC |
| XLP1-E9-1-mCit-WPRE | ATGCCGTTCAACTGGCTATC |
| XLP1-E9-2-mCit-WPRE | ATGGAACCTACTCACACCTC |
| XLP1-E10-1-mCit-WPRE | ATGGATTGAGCGACGAACGT |
| XLP1-E10-2-mCit-WPRE | ATGTTCGTAGCGAATAGACT |
| XLP1-E11-1-mCit-WPRE | ATTCGATCGGCGGAGGCATT |
| XLP1-E11-2-mCit-WPRE | CAACACGGAACTTCGTCACG |
| XLP1-E12-1-mCit-WPRE | CAATGTGCGAGATTACGTAC |
| XLP1-E12-2-mCit-WPRE | CACGGTTGAAGTACTACGCG |
| XLP1-E13-1-mCit-WPRE | CACTAAGTCAGAGGACAATC |
| XLP1-E13-2-mCit-WPRE | CCACAGCTCAGTTGCTCATC |
| XLP1-E14-1-mCit-WPRE | GAGTCTCCAGTTATGCGTGT |
| XLP1-E14-2-mCit-WPRE | GAGTTGGTGAGGATACAGGC |
| XLP1-E16-1-mCit-WPRE | GTCTCAAGGTTGGCAGGCT |
| XLP1-E16-2-mCit-WPRE | GTTATGAGGATTCCAGTGAC |
| XLP1-E17-1-mCit-WPRE | GTTCGCCGTACATATGCGTC |
| XLP1-E17-2-mCit-WPRE | TACTTGGCAGGTCCAAGTGT |
| XLP1-E18-1-mCit-WPRE | CTTCAGACAGGCGATCTCGT |
| XLP1-E18-2-mCit-WPRE | GACGCAGCTACGATCTACTC |
| XLP1-E19-1-mCit-WPRE | CGTTAAGCGGCAAGTCTTCT |
| XLP1-E19-2-mCit-WPRE | CTAGGTATCATCCGCGAATC |
| XLP1-E20-1-mCit-WPRE | GAACCGGTAGTGCAGCAACT |
| XLP1-E20-2-mCit-WPRE | GAACGCTCCAGTCAAGCTCC |

| | |
|---|---|
| XLP1-E21-1-mCit-WPRE | GACATCGACAAGTCAGGTGC |
| XLP1-E21-2-mCit-WPRE | GACCATAGAGGTAGAGCGGT |
| XLP1-E22-1-mCit-WPRE | GGATTCGCAATGAGACCTCG |
| XLP1-E22-2-mCit-WPRE | GGATTGCAGGCCTAATACCT |
| XLP1-E23-1-mCit-WPRE | TCCTCCGTTAGCTATTGTTC |
| XLP1-E23-2-mCit-WPRE | TCGGATGTCAACGAAGGATC |
| XLP1-E25-1-mCit-WPRE | TCTCTGTAAGGTTGCCTTAT |
| XLP1-E25-2-mCit-WPRE | TGACAATGCAGAGTAGTGCC |
| XLP1-E26-1-mCit-WPRE | CTCCTAAGAATCCGTACTGG |
| XLP1-E26-2-mCit-WPRE | CTGTTCCACATTGCTGAACC |
| XLP1-E30-1-mCit-WPRE | CGCAGATAGGTAGCTCGCCT |
| XLP1-E30-2-mCit-WPRE | CGCGTTAATATAGCCTTATC |
| XLP1-E31-1-mCit-WPRE | CCGGTATGCAGGTCGTATCC |
| XLP1-E31-2-mCit-WPRE | CCTTGGTGGAAGTTGTCCAC |
| XLP1-Int1-1-mCit-WPRE | CGACAACAAGGCTGTGGCAT |
| XLP1-Int1-2-mCit-WPRE | CGATCCTAGAGTCTTCCGAC |
| XLP-5'Region-1-mCit-WPRE | GCATACTAAGCCTCTCGAGT |
| XLP-5'Region-2-mCit-WPRE | GGAAGAAGAAGCTGACATGG |
| XLP-5'RegionCore-1-mCit-WPRE | TAGATCAGAGATGCGCAGGT |
| XLP-5'RegionCore-2-mCit-WPRE | TCAGCTCAAGCATCCTTCAT |
| XLP-5'RegionUltraCore-1-mCit-WPRE | CGGTGTGAAGCGGTACACAT |
| XLP-5'RegionUltraCore-2-mCit-WPRE | CGTATCTCCACCGAACTTCC |
| XLP-3Region-1-mCit-WPRE | GGCGAACACATTGGAATTGC |
| XLP-3Region-2-mCit-WPRE | GGCGTTGTAGGCTCGATACT |
| XLP1-Pro-1-mCit-WPRE | TTATCGAGAGTACGTGAGGT |
| XLP1-Pro-2-mCit-WPRE | TTGCCACAGGATAGCGACCT |
| XLP1-EFS-1-mCit-WPRE | TGCCGAATAGTATGCTGTGT |
| XLP1-EFS-2-mCit-WPRE | TGTACCACGGAGTGGACTCT |
| XLP1-Cntrl-1-mCit-WPRE | ACAACGGTGAATAGTAAGGC |
| XLP1-Cntrl-2-mCit-WPRE | ACAAGAGAAGTGTTCGCAAT |

**Table S8: Primers used for cloning**

See Supplemental Table File
