# Supplementary material for: Lentiviral vectors for precise expression to treat X-linked lymphoproliferative disease
Source: Mol Ther Methods Clin Dev. 2024 Aug 20;32(4):101323. doi: 10.1016/j.omtm.2024.101323 (PMC11415656; doi:10.1016/j.omtm.2024.101323)
Supplement: Document S2. Article plus supplemental information [file mmc3.pdf]

Molecular Therapy
Methods & Clinical Development
Original Article

Lentiviral vectors for precise expression to treat X-linked lymphoproliferative disease

Paul G. Ayoub,[1] Julia Gensheimer,[3,7] Lindsay Lathrop,[1] Colin Juett,[2] Jason Quintos,[2] Kevin Tam,[2] Jack Reid,[2] Feiyang Ma,[2] Curtis Tam,[2] Grace E. McAuley,[2] Devin Brown,[2] Xiaomeng Wu,[2] Ruixue Zhang,[2] Kathryn Bradford,[3] Roger P. Hollis,[2] Gay M. Crooks,[3,4,5,6,7] and Donald B. Kohn[1,2,3,4,5]

[1]Department of Molecular & Medical Pharmacology, University of California, Los Angeles, Los Angeles, CA 90095, USA; [2]Department of Microbiology, Immunology & Molecular Genetics, University of California, Los Angeles, Los Angeles, CA 90095, USA; [3]David Geffen School of Medicine, University of California, Los Angeles, Los Angeles, CA 90095, USA; [4]Division of Pediatric Hematology-Oncology, University of California, Los Angeles, Los Angeles, CA 90095, USA; [5]Eli & Edythe Broad Center for Regenerative Medicine & Stem Cell Research, University of California, Los Angeles, Los Angeles, CA 90095, USA; [6]Jonsson Comprehensive Cancer Center, University of California, Los Angeles, Los Angeles, CA 90095, USA; [7]Department of Pathology & Laboratory Medicine, University of California, Los Angeles, Los Angeles, CA 90095, USA

X-linked lymphoproliferative disease (XLP1) results from *SH2D1A* gene mutations affecting the SLAM-associated protein (SAP). A regulated lentiviral vector (LV), XLP-SMART LV, designed to express SAP at therapeutic levels in T, NK, and NKT cells, is crucial for effective gene therapy. We experimentally identified 34 genomic regulatory elements of the *SH2D1A* gene and designed XLP-SMART LVs to emulate the lineage and stage-specific control of SAP. We screened them for their on-target enhancer activity in T, NK, and NKT cells and their off-target enhancer activity in B cell and myeloid populations. In combination, three enhancer elements increased SAP promoter expression up to 4-fold in on-target populations *in vitro*. NSG-Tg(Hu-IL15) xenograft studies with XLP-SMART LVs demonstrated up to 7-fold greater expression in on-target cells over a control EFS-LV, with no off-target expression. The XLP-SMART LVs exhibited stage-specific T and NK cell expression in peripheral blood, bone marrow, spleen, and thymic tissues (mimicking expression patterns of SAP). Transduction of XLP1 patient CD8+ T cells or BM CD34+ cells with XLP-SMART LVs restored restimulation-induced cell death and NK cytotoxicity to wild-type levels, respectively. These data demonstrate that it is feasible to create a lineage and stage-specific LV to restore the XLP1 phenotype by gene therapy.

INTRODUCTION

X-linked lymphoproliferative disease (XLP1), also known as Duncan disease, is an inborn error of immunity caused by mutations in the *SH2D1A* gene, affecting 1 in 1 million males.[1–5] The *SH2D1A* gene encodes the SLAM-associated protein (SAP), an adaptor molecule involved in the signaling of immune cell receptors of the SLAM family.[1–5] SAP binds to the intracellular domain of SLAM family signaling receptors, and supports the activation or inhibition of immune cell signaling.[2,4,6–8] SAP mRNA and protein expression are predominately expressed in human thymocytes, T cells, NK cells, and NKT cells.[2,4,6–12] Patients with XLP1 suffer from impairments in CD4+ T cell function, CD8+ T cell cytotoxicity, NK cell cytotoxicity, plasma cell and memory B cell generation, and NKT cell development.[13–16]

In over 90% of XLP1 cases, Epstein-Barr virus (EBV) primary infection is the major cause for clinical presentations of the disease.[1,2,17] After EBV infection, XLP1 patients mount a dysregulated immune response, with nearly 60% of patients developing hemophagocytic lymphohistiocytosis (HLH).[1,8] HLH treatment is highly immune suppressive and can be significantly toxic.[18] As such, the mortality associated with HLH presentation is greater than 60%.[1] Those that survive the EBV infection may develop malignant lymphoma, hypogammaglobulinemia, and lymphoproliferation, and are thus treated with continuous immunoglobulin replacement therapy (IRT) and immune suppression.[9] The expense and inconvenience of life-long IRT, with administration requiring intravenous or subcutaneous injections, are drawbacks, but are accepted due to the important clinical benefits IRT confers. The only curative treatment includes the use of an allogeneic hematopoietic stem cell (HSC) transplantation, in which CD34+ HSCs are taken from a healthy suitable-matched donor and transplanted into the patient to give rise to a fully functionally immune system.[19] Many patients, unfortunately, lack this treatment as a viable option due to poor donor availability and immunologic complications. Thus, a more effective treatment for XLP1 remains an unmet need, and—given the severe nature of the disease—an autologous HSC transplantation is a viable approach.[11] An autologous HSC transplant treats a patient's own HSCs to integrate a stable copy of the *SH2D1A ex vivo* with a lentiviral vector (LV).[11] This method of gene therapy may provide the same benefit as an allogeneic transplant, while eliminating any risks of graft rejection or graft-versus-host

Received 6 November 2023; accepted 15 August 2024;
https://doi.org/10.1016/j.omtm.2024.101323.

Correspondence: Donald B. Kohn, Department of Molecular & Medical Pharmacology, University of California, Los Angeles, Los Angeles, CA 90095, USA.
E-mail: dkohn1@mednet.ucla.edu

© 2024 The Author(s). Published by Elsevier Inc. on behalf of The American Society of Gene and Cell Therapy.
This is an open access article under the CC BY license (http://creativecommons.org/licenses/by/4.0/).

disease, since each patient serves as their own donor. A successful treatment by autologous HSC transplantation requires an LV capable of transducing a functional copy of *SH2D1A* into long-lived multipotent HSCs expressing within target leukocyte lineages.[1,11]

A LV was developed previously for gene therapy of XLP1 through LV-mediated gene transfer into autologous HSCs.[11] However, this LV did not utilize the endogenous regulatory elements of the encoded transgene like many of the current clinical LVs, thereby it did not recapitulate the precise expression pattern of the native *SH2D1A* gene.[11,20] Instead, a ubiquitously expressed promoter known as the elongation factor 1 α short promoter (EFS) was used. With this approach, the endogenous promoter alone is insufficient to drive proper physiological expression and regulation of the gene, as additional regulatory elements such as distant enhancer and silencers are also required.[21] Since SAP expression is tightly regulated within T, NK, and NKT cells, gene expression in off-target HSC populations may pose safety concerns including skewing of hematopoietic potential and dysregulated lymphocyte development and function.[11,22,23]

Not all groups utilize ubiquitously expressed promoters within LV gene therapies. Instead, some have employed natural regulatory elements to control promoter activity. This approach has been applied in developing LV gene therapies for conditions such as hemoglobinopathies and IPEX syndrome.[24,25] In these specific instances, decades of research were dedicated to identifying key native promoter/enhancer regions. This led to the discovery of the locus control region (LCR) and the conserved noncoding sequence (CNS) areas. The LCR was used to regulate the β-globin promoter in hemoglobinopathy treatments, and the CNS was applied to the FoxP3 promoter for IPEX syndrome therapies.[24–26] However, the elucidation of these regions was backed by extensive research, including studying the regulation of the globin genes (specifically *HBB*) and using mouse knockout models to explore and define the regulatory CNS elements within the FoxP3 locus (pertaining to *FOXP3*).[24–26] In contrast, our group has advanced this approach utilizing bioinformatics-assisted design to quickly identify and construct LVs driven by the endogenous promoter and regulatory elements necessary to treat inborn errors of immunity such as X-linked chronic granulomatous disease (X-CGD) and Wiskott-Aldrich syndrome.[27] The approach leveraged the publicly available bioinformatic tool GeneHancer database, which pools data from ENCODE, Ensembl, FANTOM, and VISTA. By integrating data, including histone modification, chromatin accessibility, bound transcription factors, and Hi-C interactions across multiple datasets, we identified various putative enhancer regions that assist with regulating their target gene.[27] Using this methodology, we recently described an LV with a superior lineage-specific expression pattern compared with an LV currently in clinical trials for X-CGD that uses non-endogenous regulatory elements from other myeloid lineage genes.[27]

We have since optimized this methodology for the efficient identification and testing of endogenous regulatory elements to generate rationally designed bioinformatics-assisted lentiviruses for the treatment of XLP1. Transduction of autologous CD34+ hematopoietic stem and progenitor cells (HSPCs) using LVs that contain regulatory elements of the *SH2D1A* gene can achieve lineage- and stage-specific expression of SAP for maximal therapeutic benefit, with minimal off-target expression in inappropriate cell types. Rationally designed LVs with *SH2D1A* gene-specific promoter/enhancers (XLP-SMART LVs) may achieve functional restoration of humoral and cytotoxic defects in XLP1 patients through gene therapy.

RESULTS

To elucidate the putative elements responsible for the lineage- and stage-specific expression of the endogenous *SH2D1A* locus, we employed a bioinformatics-guided approach utilizing GeneHancer, a bioinformatic tool that links over 285,000 candidate enhancer elements across the human genome to their respective target genes.[28] GeneHancer integrates four genome-wide enhancer databases (ENCODE, Ensembl, FANTOM, and VISTA) to generate a comprehensive list of putative regulatory elements for each gene. By integrating data, including histone modification, chromatin accessibility, bound transcription factors, and Hi-C interactions, we identified 34 potential regulatory elements of *SH2D1A*, located within a 200 kb window of the *SH2D1A* transcription start site (Table S1). We entered each genomic coordinate into the UCSC Genome Browser to examine the corresponding regulatory elements (Figure 1A).

We designed a series of LVs (XLP1-SMART LVs) to test each element's enhancer capability independently (Figure 1A). The elements were inserted upstream of a 600 bp *SH2D1A* promoter—identified via the Eukaryotic Promoter Database —to drive expression of an mCitrine (mCit) reporter cassette. The mCitrine gene is a variant of eGFP, with increased fluorescent intensity over the traditional GFP fluorophore.[27] Furthermore, each vector was designed with the woodchuck hepatitis virus post-transcriptional regulatory element (WPRE) replacing the endogenous 3′ UTR of *SH2D1A* to enhance expression of the transgene cassette. To multiplex the series of vectors, unique 20 bp barcodes were cloned directly upstream of the WPRE (Figure 1A). At this location, the barcode remains within the transcript and vector provirus genome for identification and normalization by RNA or DNA, respectively, but it will not be translated to affect any protein function. The barcodes were designed, each with a Hamming distance of 10, to tolerate accidental mutations.[29] Each of the 34 vectors was cloned twice—each with its own unique barcode—to further assess any potential biases from transduction, recombination, or PCR amplification. An LV used in pre-clinical XLP1 gene therapy studies, EFS-SAP, was cloned in duplicate as a control.[11] The *SH2D1A* gene was replaced with an mCit reporter and termed EFS-mCit.

To mimic the endogenous expression of SAP, we tested each LV construct for its ability to drive high-level expression in primary T cells, NKT cells, and NK cells, each isolated from healthy donor peripheral blood mononuclear cells (PBMCs). B lymphoblastoid cell lines (B-LCLs) were utilized to assess off-target B lymphoid expression of each regulatory element. We transduced each cell population with the 34 candidate vectors and the EFS control. Fourteen days

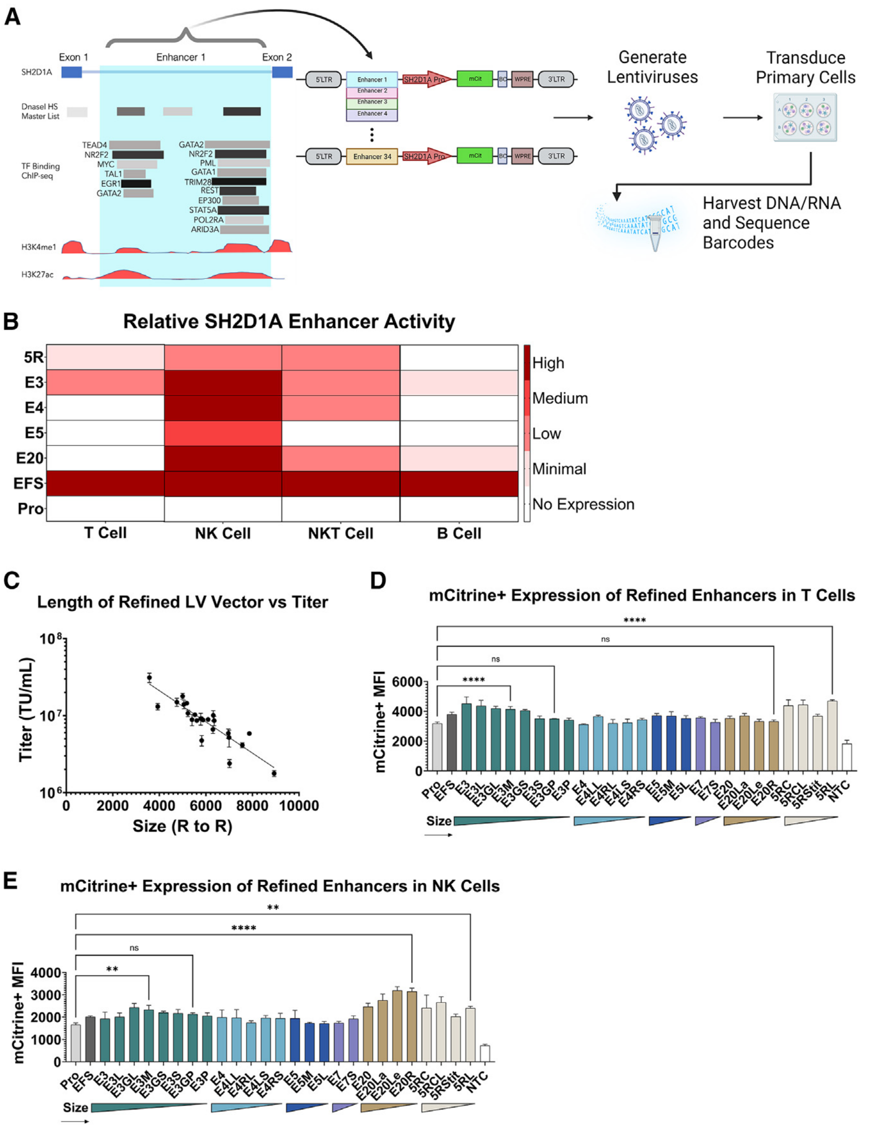

Figure 1. Functional Characterization and Optimization of SH2D1A Enhancers Across T, NK, and B Cell Lineages

(A) UCSC Genome Browser interface of the *SH2D1A* locus encoding the SAP protein. Blue shaded columns indicate putative regulatory regions. Distinctive DNase I hypersensitive sites (DHSs) are shown across different cell lineages. Transcription factor (TF) binding, along with peaks of bound H3K4me1, H3K27ac, and DHSs can be used to define the presence and boundaries of putative enhancer elements. Proviral maps of a series of XLP1 "SMART" lentiviral vector constructs each contain a putative regulatory element upstream of the endogenous *SH2D1A* promoter (*SH2D1A* Pro) that drive expression of an mCitrine (mCit) reporter cassette. A unique barcode (BC) is located upstream of the woodchuck hepatitis virus post-transcriptional regulatory element (WPRE) to identify the ability for each element to drive lineage and stage specific expression of the mCit reporter. We transduced primary T, NKT, and NK cells with a pool of raw viral supernatant containing each of the 34 candidate XLP-SMART LVs, in duplicate with different BC, and the EFS-SAP vector as control. B-LCLs were transduced to measure off-target expression in the B lymphocyte lineage. Fourteen days post-transduction, cells were harvested for their gDNA and RNA fractions to measure BC expression and presence in gDNA via next-generation sequencing. (B) Relative *SH2D1A* enhancer activity of the highest-expressing elements. The relative enhancer expression measured by next-generation sequencing of vector barcodes is shown. The highest-expressing 5 enhancers are depicted (see supplemental figures for the remaining 29), with the lowest level of expression (white) denoted as that of the *SH2D1A* promoter only (Pro). Elements of interest contain increased expression over the *SH2D1A* Pro (red). Minimal = 1- to 1.25-fold increase; low = 1.25- to 1.5-fold increase; medium = 1.5- to 2-fold increase; high = >2-fold increase. (C) Proviral size of refined XLP-SMART LV versus titer. Putative enhancers were cloned into the plasmid backbone of a lentiviral vector (pCCL-c-MNDU3-X [Addgene, plasmid no. 81071]) with the MNDU3 promoter first removed, and the vectors were packaged and titered head-to-head. The quantities of infectious particles were plotted as a function of proviral length (bp). Each point in the plot represents an average of three individual 10-cm plates of virus titered on HT-29 cells. Proviral length is defined as sequence length from the beginning of the 5′ long terminal repeat (LTR) U3 through the end of the 3′ LTR U5. $n = 3$ per arm. Linear regression analyses were used to determine the correlation between titer and proviral size ($R^2 = 0.78$). (D and E) Expression from LV with refined enhancer elements (via GFP mean fluorescence intensity [MFI]) in T and NK cells. Healthy donor CD3+ T cells or CD56+ NK cells were isolated from peripheral blood mononuclear cells (PBMCs). CD3+ T cells and CD56+ NK cells were transduced with each XLP-SMART LV to achieve a VCN ranging from 0.1 to 0.2. 14 days post-transduction (based on pre-determined titers), T cells were assessed for relative expression driven by each enhancer via mCitrine+ MFI using flow cytometry. Each enhancer was compared with basal *SH2D1A* promoter-driven expression (Pro) and the control LV (EFS). Data are represented as mean ± SD of biological triplicates from two experiments. Statistical significance was analyzed using a one-way ANOVA followed by multiple paired comparisons for normally distributed data (Tukey test). Statistical analysis was performed on all arms, but selected arms are shown. All statistical tests were two-tailed and a p value of <0.05 was deemed significant (ns, non-significant; *$p < 0.05$, **$p < 0.01$, ***$p < 0.001$, ****$p < 0.0001$).

post-transduction, cells were harvested to isolate their gDNA and mRNA fractions. Bulk vector copy number (VCN) of the transduced populations was assessed via droplet digital PCR (ddPCR). The barcode-containing DNA regions were PCR amplified from the gDNA fraction and assessed via next-generation sequencing. Furthermore, the barcode-containing regions were PCR amplified from the RNA fraction after cDNA conversion and assessed via next-generation sequencing. The RNA barcode counts are proportionate to the transcriptional activity of the enhancers within each lineage. The genomic (DNA) barcode count is used to normalize each barcode in the transcript to account for potential differences in transduction efficiency of the vectors. The number of RNA barcode reads normalized to

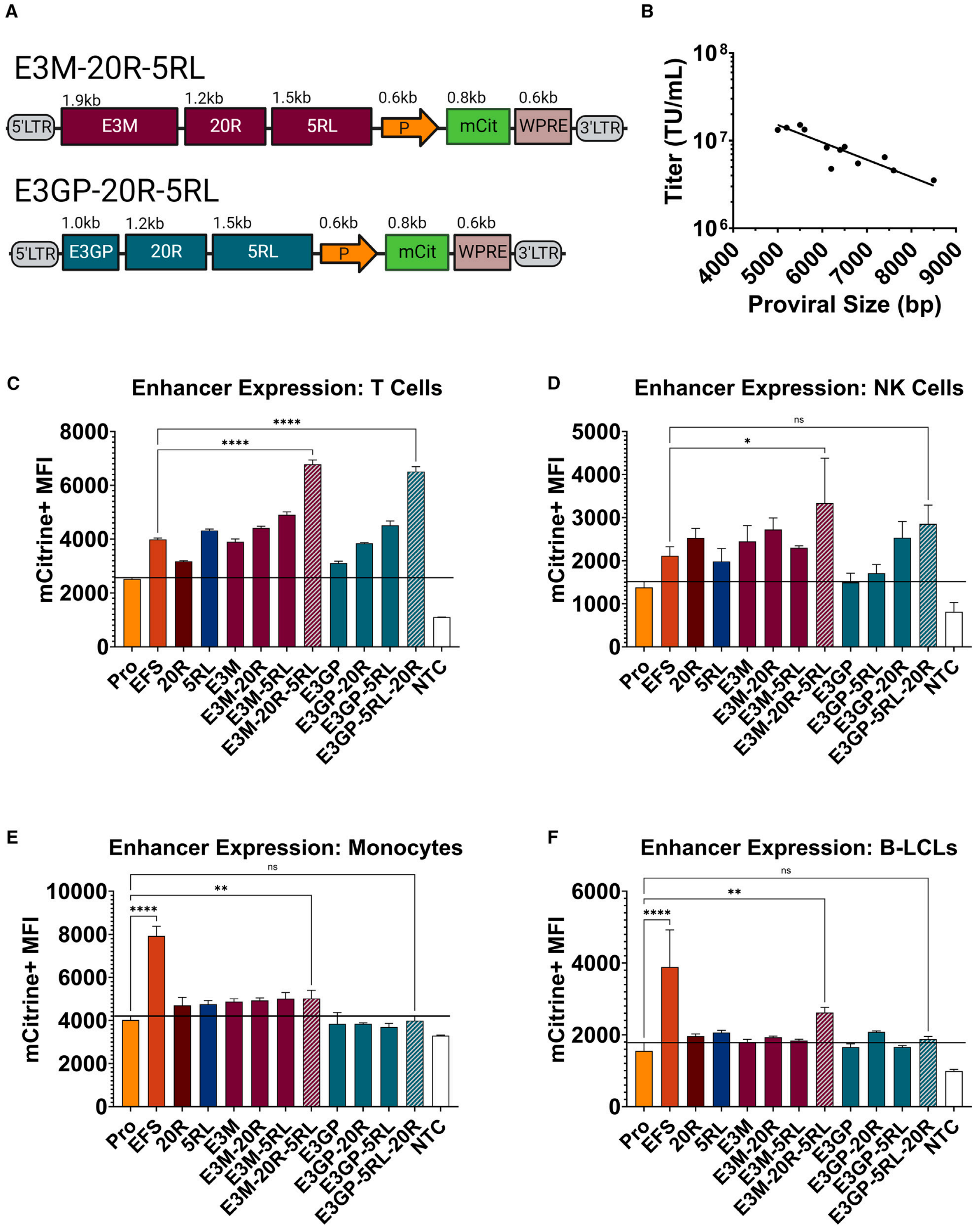

(legend on next page)

frequency of gDNA barcodes within each cell type determined the relative expression directed by each element (Figure 1A).

Of the 34 regulatory elements tested, elements 3 and 5R demonstrated T cell-specific enhancer expression (Figures 1B and S1A–S1C). Elements 3, 4, 5, 7, 20, and 5R demonstrated NK and NKT cell-specific enhancer expression (Figures 1B, and S1D–S1E). All 34 regulatory elements tested demonstrated minimal to no off-target expression in B-LCLs (Figures 1B and S1F).

Enhancers 3, 4, 5, 7, 20, and 5R were refined to decrease their size (thereby increasing the vector titers and gene transfer) with the goal of retaining high enhancer-driven function (Figures 1C–1E). LV plasmids were designed with the refined enhancer fragments and the 600 bp *SH2D1A* promoter driving expression of an mCit cassette. LV plasmids were packaged and titered head-to-head using methods previously described by our laboratory (Figure 1C).[26,27,30] Primary T and NK cells were transduced with the refined LVs to achieve equivalent VCNs ranging from ~0.10 to 0.20 to increase the probability of each transduced cell containing a single integrant.[27] The mean fluorescence intensity (MFI) of the mCit+ populations were assessed via flow cytometry to compare the ability of each refined enhancer element to drive mCit expression in T cells (Figure 1D) and NK cells (Figure 1E). Enhancers E3M and 5RL retained 90% and 100% of the full-length enhancer activity in T cells, while reducing the enhancer sizes by 3.5 and 4 kb, respectively, from the original elements (Figure 1D). Enhancers 3M, 3GP, 20R, and 5RL retained 120%, 110%, 130%, and 100% of the full-length enhancer activity in NK cells, while reducing the enhancer sizes by 3.5, 4.2, 1, and 4 kb, respectively (Figure 1E). Enhancers 4, 5, and 7 and their shortened iterations did not significantly increase expression in T cells or NK cells when compared with the promoter-only control and were thus removed from subsequent analyses (Figures 1D and 1E). After refining the enhancers, the elements were combined to assess additive and/or synergistic effects on expression.

Enhancer E3M was combined with either 20R, 5RL or both 20R and 5RL, and a 600 bp *SH2D1A* promoter to drive expression of an mCit cassette (Figure 2A). Similarly, LV plasmids were packaged and titered head-to-head using methods previously described by our lab (Figure 2B).[26,27,30] We transduced primary T and NK cells for evaluating on-target expression (Figures 2C and 2D). Transduced cord blood (CB) CD34+ cells were differentiated into monocytes and, along with B-LCLs, were used to determine off-target expression levels (Figures 2E and 2F).

Enhancers E3M/E3GP, 20R, and 5RL demonstrated additive expression levels when combined and evaluated in primary T and NK cells (Figures 2C and 2D). The combination vectors (XLP1-SMART LVs) increased mCit expression in T cells up to 4-fold compared with the

Figure 2. In Vitro Characterization of Composite XLP1-SMART Lentiviral Vectors: Proviral Size, Titer, and Cell Lineage-Specific Expression

(A) Schematic of composite XLP1-SMART lentiviral vectors. Diagrams of the two XLP-SMART LV composite constructs are shown with their sequence lengths (kb). 5′ LTR and 3′ LTR designate the 5’ and 3′ viral long terminal repeats (LTRs), respectively; E3M, E3GP, 20R, and 5RL are *SH2D1A* enhancer elements; P designates the *SH2D1A* promoter; mCit, mCitrine reporter cassette; WPRE, woodchuck hepatitis virus post-transcriptional regulatory element. (B) Proviral size of composite XLP-SMART LVs versus titer. Enhancers were cloned into the plasmid backbone of a therapeutic lentiviral vector (pCCL-c-MNDU3-X [Addgene, plasmid no. 81071]) (with the MNDU3 promoter removed), packaged, and titered head-to-head. The quantities of infectious particles were plotted as a function of proviral length (bp). Each point in the plot represents an average of three individual 10-cm plates of virus titered on HT-29 cells. Proviral length is defined as sequence length from the beginning of the 5′ long terminal repeat (LTR) U3 through the end of the 3′ LTR U5. $n = 3$ per arm. Linear regression analyses were used to determine the correlation between titer and proviral size ($R^2 = 0.79$). (C) On-target expression in T cells *in vitro* of composite XLP-SMART LVs. Healthy donor CD3+ T cells were isolated from PBMCs. CD3+ T cells were transduced with each XLP-SMART LV to achieve a VCN ranging from 0.1 to 0.2. At 14 days post-transduction, T cells were assessed for the relative expression driven by each enhancer via mCitrine+ MFI using flow cytometry. Each enhancer was compared with basal *SH2D1A* promoter expression (Pro), the control LV (EFS), and a non-transduced control (NTC). Data are represented as mean ± SD of biological triplicates from three experiments. Statistical significance was analyzed using a one-way ANOVA followed by multiple paired comparisons for normally distributed data (Tukey test). Statistical analysis was performed on all arms, but selected arms are shown. All statistical tests were two-tailed and a p value of <0.05 was deemed significant (ns, non-significant; *$p < 0.05$, **$p < 0.01$, ***$p < 0.001$, ****$p < 0.0001$). (D) On-target expression in NK cells *in vitro* of composite XLP-SMART LVs. Healthy donor CD56+ NK cells were isolated from PBMCs. CD56+ NK Cells were transduced with each XLP-SMART LV to achieve a VCN ranging from 0.1 to 0.2. At 14 days post-transduction, NK cells were assessed for the relative expression driven by each enhancer via mCitrine+ MFI using flow cytometry. Each enhancer was compared with basal *SH2D1A* promoter expression (Pro) and the control LV (EFS). Data are represented as mean ± SD of biological triplicates from three experiments. Statistical significance was analyzed using a one-way ANOVA followed by multiple paired comparisons for normally distributed data (Tukey test). Statistical analysis was performed on all arms, but selected arms are shown. All statistical tests were two-tailed and a p value of <0.05 was deemed significant (ns, non-significant; *$p < 0.05$, **$p < 0.01$, ***$p < 0.001$, ****$p < 0.0001$). (E) Off-target expression in CB CD34+ differentiated monocytes cells by composite XLP-SMART LVs. Healthy donor CB CD34+ cells were differentiated into monocytes as described.[31] Prior to differentiation, CB CD34+ cells were transduced with each XLP-SMART LV to achieve a VCN ranging from 0.1 to 0.2. At 14 days post-transduction and differentiation, CD14+CD16+ monocytes were assessed for the relative expression driven by each enhancer via mCitrine+ MFI using flow cytometry. Each enhancer was compared with basal *SH2D1A* promoter expression (Pro) and the control LV (EFS). Data are represented as mean ± SD of biological triplicates from one experiment. Statistical significance was analyzed using a one-way ANOVA followed by multiple paired comparisons for normally distributed data (Tukey test). Statistical analysis was performed on all arms, but selected arms are shown. All statistical tests were two-tailed and a p value of <0.05 was deemed significant (ns, non-significant; *$p < 0.05$, **$p < 0.01$, ***$p < 0.001$, ****$p < 0.0001$). (F) Off-target expression in B-LCLs by composite XLP-SMART LVs. B-LCLs, cultured in R10, were transduced with each XLP-SMART LV to achieve a VCN ranging from 0.1 to 0.2. At 14 days post-transduction, B-LCLs were assessed for the relative expression driven by each enhancer via mCitrine+ MFI using flow cytometry. Each enhancer was compared with basal *SH2D1A* promoter expression (Pro) and the control LV (EFS). Data are represented as mean ± SD of biological triplicates from three experiments. Statistical significance was analyzed using a one-way ANOVA followed by multiple paired comparisons for normally distributed data (Tukey test). Statistical analysis was performed on all arms, but selected arms are shown. All statistical tests were two-tailed and a p value of <0.05 was deemed significant (ns, non-significant; *$p < 0.05$, **$p < 0.01$, ***$p < 0.001$, ****$p < 0.0001$).

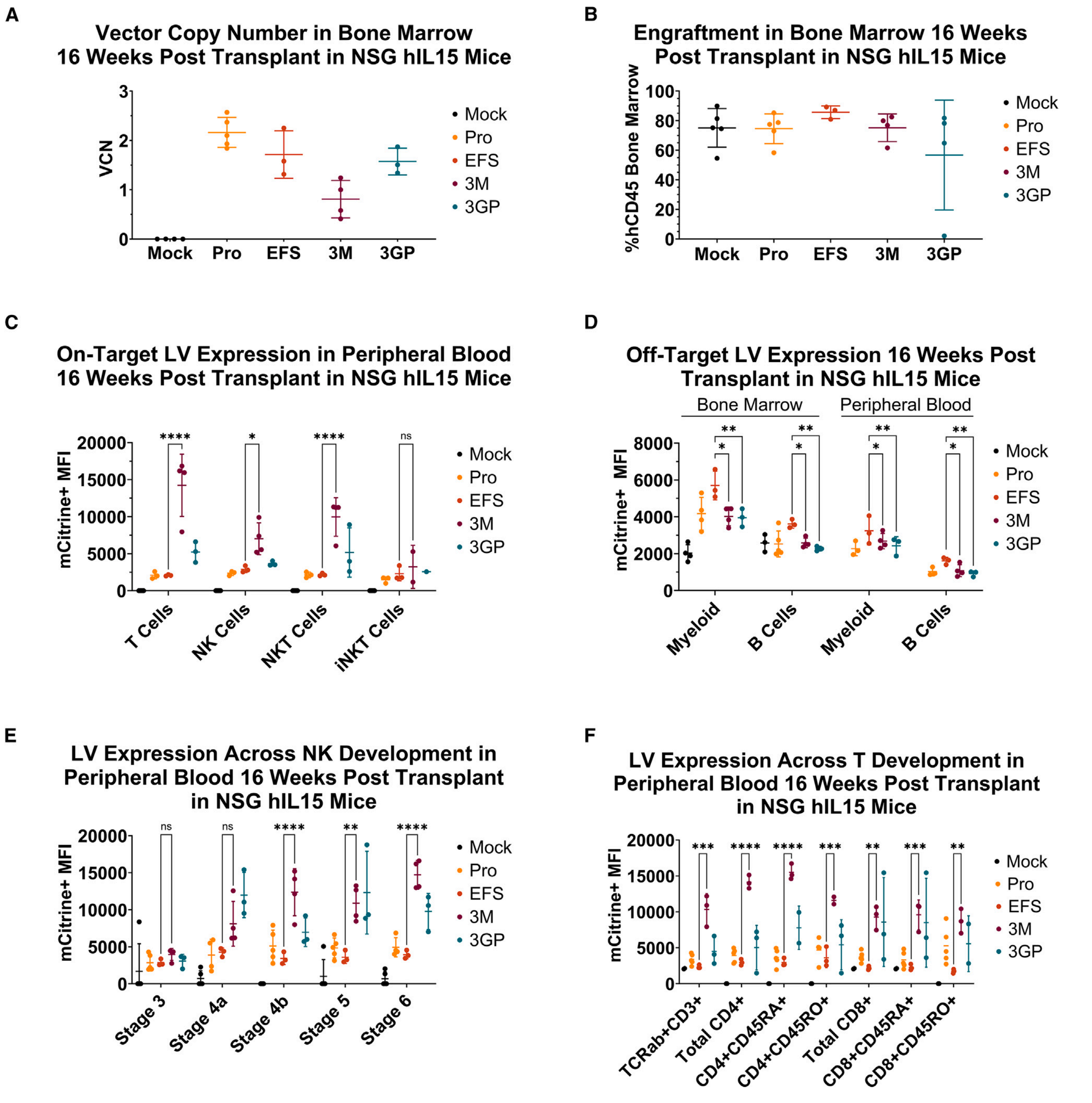

Figure 3. Lineage-Specific Expression of XLP-SMART Lentiviral Vectors in NSG-Tg(Hu-IL15) Mice

(A) Vector copy number 16 weeks post-transplant from NSG-Tg(Hu-IL15) mouse bone marrow (BM). Whole BM was taken from each mouse at time of euthanasia and processed into a single-cell suspension. Genomic DNA was extracted from the BM suspension and analyzed for vector copy number by ddPCR. *n* = 4, mock; *n* = 5, promoter only (Pro); *n* = 3, EFS-mCitrine (EFS); *n* = 4, E3M-20R-5RL-mCitrine (3M); *n* = 3, E3GP-20R-5RL-mCitrine (3GP). Data are represented as mean ± SD of biological replicates from one experiment. (B) Engraftment 16 weeks post-transplant from NSG-Tg(Hu-IL15) mouse BM. Whole BM was taken from each mouse at time of euthanasia and analyzed for engraftment by flow cytometry using an anti-hCD45 antibody. *n* = 5, Mock; *n* = 5, promoter only (Pro); *n* = 3, EFS-mCitrine (EFS); *n* = 4, E3M-20R-5RL-mCitrine (3M); *n* = 4, E3GP-20R-5RL-mCitrine (3GP). (C) On-target XLP-SMART LV expression in peripheral blood 16 weeks post-transplant in NSG-Tg(Hu-IL15) mice. Mice were bled at 16 weeks post-transplant to analyze peripheral blood for XLP-SMART LV expression. Lysed red blood cells were stained for various on-target lineages within the hCD45+ gate (T cells: hCD33–, hCD19–, hCD3+; NK cells: hCD33-, hCD3-, hCD19-, hCD56+; NKT cells: hCD33-, hCD19-, hCD3+, hCD56+; and iNKT cells: hCD33–, hCD19–, hCD3+, hCD56+, hVα24+). Each LV's relative expression was measured in on-target lineages via mCitrine+ MFI using flow cytometry. Each enhancer was

(legend continued on next page)

promoter-only control and up to 1.8-fold greater than the control EFS vector (Figure 2C). Furthermore, the combination vectors increased mCit expression in NK cells 2-fold greater than the promoter-only control and 1.5-fold greater than the EFS preclinical vector (Figure 2D). XLP1-SMART LVs that included E3M showed minor levels of off-target expression, with the E3M combination XLP-SMART LV (E3M-20R-5RL-mCit) harboring a 1.25- or 1.67-fold increase in mCit expression levels in monocytes and B cells, respectively, over the promoter-only control. However, the E3GP combination XLP-SMART LV (E3GP-20R-5RL-mCit) showed no significant levels of off-target expression (Figures 2E and 2F).

To determine the lineage- and stage-specific expression of the XLP1-SMART LVs, we tested the ability of each LV to drive mCit reporter expression *in vivo* in NSG-Tg(Hu-IL15)-immunodeficient mice. Healthy human donor CB CD34+ HSCs were pre-stimulated for 24 h in 50 ng/mL of human hSCF, hTPO, and hFT3L before transduction with 2e–7 TU/mL of our XLP-SMART LVs. The E3M-20R-5RL and E3GP-20R-5RL LVs were compared with both mock transduced cells and to CB CD34+ HSCs transduced with 2e–7 TU/mL of the control EFS-mCit vector. We transplanted the transduced cells via intra-hepatic injection into sub-lethally irradiated NOD.Cg-*Prkdc*^{scid} *Il2rg*^{tm1Wjl} Tg(IL15)1Sz/SzJ (NSG-Tg [HuIL15]) neonatal pups receiving 150 rads of irradiation. These NSG-Tg(HuIL15) mice express human IL-15 to help enhance the development of human NK cells in mice engrafted with human CD34+ cells.[32] Furthermore, the transplantation of NSG neonatal pups supports more efficient human T cell development after HSC injection than adult NSG mice.[33]

Mice were sacrificed 16 weeks post-transplantation to assess engraftment and vector-mediated gene expression in multiple human hematopoietic cell lineages. Bone marrow, thymus, and spleen were processed into single-cell suspensions and peripheral blood was collected. The VCN was determined for the transplanted cells within the bone marrow of each mouse (Figure 3A) and the bone marrow engraftment (Figure 3B) of each mouse was quantified via the percent of hCD45+ human cells via flow cytometry. Average VCNs for promoter-only (Pro), EFS-mCit (EFS), E3M-20R-5RL-mCit (3M), and E3GP-20R-5RL-mCit (3GP) in the bone marrow compartment of NSG-Tg(Hu-IL15) mice were 2.17, 1.72, 0.81, and 1.57, respectively. With exception of one mouse that did not engraft in the E3GP-20R-5RL-mCit group, average human cell engraftment was 75% for all groups.

We evaluated lineage-specific expression of the XLP-SMART LVs by analyzing the MFI of mCit+ expression in different on- and off-target populations of hCD45+ cells. Mice transplanted with CB CD34+ HSCs transduced with the E3M-20R-5RL-mCit LV contained 7.1-, 2.8-, and 4.3-fold brighter mCit+ expression than the EFS-mCit vector in T cells, NK cells, and NKT cells, respectively (Figure 3C). Mice transplanted with CB CD34+ HSCs transduced with the E3GP-20R-5RL-mCit LV contained 2.0-, 1.6-, and 2.0-fold brighter mCit+ expression than the EFS-mCit vector in T cells, NK cells, and NKT cells, respectively (Figure 3C). Furthermore, mice transplanted with CB CD34+ HSCs transduced with E3M-20R-5RL-mCit or E3GP-20R-5RL-mCit LVs contained no off-target expression in myeloid cells or B cells in both the bone marrow and peripheral blood compartments, unlike the EFS-mCit counterpart (Figure 3D).

compared with basal *SH2D1A* promoter-driven expression (Pro) and the control LV (EFS). Data are represented as mean ± SD of biological triplicates from one experiment. Statistical significance was analyzed using a two-way ANOVA followed by multiple paired comparisons for normally distributed data (Tukey test). Statistical analysis was performed on all arms, but selected arms are shown. All statistical tests were two-tailed and a p value of <0.05 was deemed significant (ns, non-significant; *$p < 0.05$, **$p < 0.01$, ***$p < 0.001$, ****$p < 0.0001$). (D) Off-target XLP-SMART LV expression in BM and peripheral blood (PB) 16 weeks post-transplant in NSG-Tg(Hu-IL15) mice. Whole BM was taken from each mouse at time of euthanasia and mice were bled at 16 weeks post-transplant to analyze PB for XLP-SMART LV expression. Whole BM, processed into a single-cell suspension, and red blood cell-lysed PB were stained for various off-target lineages within the hCD45+ gate (myeloid cells: hCD33+; B cells: hCD33–, hCD19+, hCD3–). Each LV's relative expression was measured in off-target lineages via mCitrine+ MFI using flow cytometry. Each enhancer was compared with basal *SH2D1A* promoter expression (Pro) and the control LV (EFS). Data are represented as mean ± SD of biological triplicates from one experiment. Statistical significance was analyzed using a two-way ANOVA followed by multiple paired comparisons for normally distributed data (Tukey test). Statistical analysis was performed on all arms, but selected arms are shown. All statistical tests were two-tailed and a p value of <0.05 was deemed significant (ns, non-significant; *$p < 0.05$, **$p < 0.01$, ***$p < 0.001$, ****$p < 0.0001$). (E) XLP-SMART LV expression across NK cell development in PB 16 weeks post-transplant in NSG-Tg(Hu-IL15) mice. Mice were bled at 16 weeks to analyze PB for XLP-SMART LV expression after red blood cell lysis. Cells were stained for various stages of NK cell differentiation within the hCD45+CD33– gate (stage 1 [data not shown]: hCD34+; stage 2a [data not shown]: hCD34+, hCD117+, hCD122–; stage 2b [data not shown]: hCD34+, hCD117+, hCD122+; stage 3: hCD34–, hCD117+, hCD122+, hCD56–; stage 4a: hCD34–, hCD117+, hCD122+, hCD56+, hCD94+; stage 4b: hCD34–, hCD117–, hCD122+, hCD56+, hCD94+, hNKp80+; stage 5: hCD34–, hCD117–, hCD122+, hCD56+, hCD94+, hNKp80+, hCD16+; and stage 6: hCD34–, hCD117–, CD122+, hCD56+, hCD94+, hNKp80+, hCD16+, hCD57+). Each LV's relative expression was measured across NK cell subpopulations via mCitrine+ MFI using flow cytometry. Each enhancer was compared with basal *SH2D1A* promoter expression (Pro) and the control LV (EFS). Data are represented as mean ± SD of biological triplicates from one experiment. Statistical significance was analyzed using a two-way ANOVA followed by multiple paired comparisons for normally distributed data (Tukey test). Statistical analysis was performed on all arms, but selected arms are shown. All statistical tests were two-tailed and a p value of <0.05 was deemed significant (ns, non-significant; *$p < 0.05$, **$p < 0.01$, ***$p < 0.001$, ****$p < 0.0001$). (F) XLP-SMART LV expression across T cell development in PB 16 weeks post-transplant in NSG-Tg(Hu-IL15) mice. Mice were bled at 16 weeks to analyze PB for XLP-SMART LV expression after red blood cell lysis. Cells were stained for various stages of mature T cell populations within the hCD45+ hCD34– hCD14– hCD19– hCD56– hCD5+ hCD7+ TCRab+ CD3+ gate (total CD4: hCD4+, hCD8–; CD4+CD45RA+: hCD4+, hCD8–, hCD45RA+, hCD45RO–; CD4+CD45RO+: hCD4+, hCD8–, hCD45RA–, hCD45RO+; total CD8: hCD4–, hCD8+; CD8+CD45RA+: hCD4–, hCD8+, hCD45RA+, hCD45RO–; CD8+CD45RO+: hCD4–, hCD8+, hCD45RA–, hCD45RO+). Each LV's relative expression was measured in mature T cell subsets via mCitrine+ MFI using flow cytometry. Each enhancer was compared with basal *SH2D1A* promoter expression (Pro) and the control LV (EFS), both harboring an mCitrine reporter cassette. Data are represented as mean ± SD of biological triplicates from one experiment. Statistical significance was analyzed using a two-way ANOVA followed by multiple paired comparisons for normally distributed data (Tukey test). Statistical analysis was performed on all arms, but selected arms are shown. All statistical tests were two-tailed and a p value of <0.05 was deemed significant (ns, non-significant; *$p < 0.05$, **$p < 0.01$, ***$p < 0.001$, ****$p < 0.0001$).

To evaluate the stage-specific expression of the XLP1-SMART LVs, we stained the bone marrow, thymus, and spleen cell suspensions with an NK cell or T cell differentiation antibody panel and evaluated their mCit+ expression via flow cytometry (Figures 3E, 3F, and S2A–2D). Mice transplanted with CB CD34+ HSCs transduced with the E3M-20R-5RL-mCit LV contained 1.7- to 3.7-fold brighter mCit+ expression than the EFS-mCit vector in mature NK lineages (stage 4a to stage 6; Figure 3E). Mice transplanted with CB CD34+ HSCs transduced with the E3GP-20R-5RL-mCit LV contained 2.1- to 3.5-fold brighter mCit+ expression than the EFS-mCit vector in mature NK lineages (stage 4a to stage 6; Figure 3E). Furthermore, the mCit+ expression in mature NK lineages from mice treated with E3M-20R-5RL-mCit LV mirrors the natural SAP expression seen in mature NK lineages of a healthy donor's peripheral blood (Figures S3B and S3D). The mice with the E3M-20R-5RL-mCit LV had 4.7- to 5.0-fold brighter mCit+ expression than cells in mice receiving the EFS-mCit vector in mature T lineages (Figures 3F, S2A, and S2B). The mice with the E3GP-20R-5RL-mCit LV had 2.0- to 4.4-fold brighter mCit+ expression than cells in mice receiving the EFS-mCit vector in mature T lineages (Figures 3F, S2A, and S2B). In addition, the mCit+ expression measured across mature T lineages in the thymus of mice with the E3M-20R-5RL-mCit LV mirrored the pattern of SAP expression across T development from a heathy donor thymus (Figures S3A and S3C).

To further test the stage specificity of the XLP1-SMART LVs, we utilized the artificial thymic organoid (ATO) system, which fully recapitulates thymopoiesis from multiple stem cell sources.[34,35] Human CD34+ mobilized peripheral blood (mPB) cells were transduced with E3M-20R-5RL-mCit LV, E3GP-20R-5RL-mCit LV, and EFS-mCit at equivalent VCN. Transduced mPB cells and a mock non-transduced control were combined with the MS5-hDLL4 stromal cell line which constitutively expresses human Notch delta-like ligand 4 (DLL4). Cell count and T cell differentiation kinetics were measured at weeks 3, 7, and 12 using flow cytometry, and the percentage of mCitrine expression was compared with endogenous SAP expression to assess the temporal specificity of the XLP1-SMART LVs across multiple stages of T cell differentiation. Thymocytes from ATOs with XLP1-SMART LVs expressed mCitrine at all stages of T cell development, consistent with the presence of SAP expression throughout T cell differentiation from uncommitted to mature thymocytes (Figures S4A and S4B).

To assess both the clonogenic potential of human hematopoietic progenitor cells after transduction with the LV and the functional restoration of the XLP1 phenotype, the mCitrine open reading frame of the XLP1-SMART LVs was replaced with the coding region of human *SH2D1A*, codon optimized using the JCat codon optimization algorithm (Figures S5A and S5B).[36] Both JCat and GeneArt algorithms were tested, changing the codons of wild-type *SH2D1A* with synonymous human codon changes to thus increase protein production (Table S2). The algorithms differ based on their considerations of codons that assist in ribosome stalling, mRNA translation, mRNA stability, and premature termination of translation.[36,37] Both optimizations were tested in *SH2D1A*−/− Jurkat cells for their relative protein production via western blotting, with JCat producing the most protein per VCN (Figures S5A and S5B).

After determining the increased *SH2D1A* expression using a JCat codon optimization, in comparison with wild-type *SH2D1A*, codon optimized XLP1-SMART LV plasmids were packaged and titered head-to-head using methods described previously.[26,27,30]

To measure the efficacy of XLP1-SMART LVs to produce SH2D1A protein, we conducted a dose response in the *SH2D1A*−/− Jurkat cells, transducing the cells with the JCat codon optimized XLP1-SMART LVs to achieve VCNs of 1, 3, and 5. At equal protein concentrations, XLP1-SMART LVs harboring the E3M enhancer demonstrated detectable levels of SH2D1A protein at VCNs of 3 and 5, reaching approximately 23% and 34% wild-type SAP protein, respectively (Figures S5C and S5D; Table S3). Furthermore, XLP1-SMART LVs harboring the E3GP enhancer demonstrated detectable levels of SH2D1A protein at VCNs of 3 and 5, reaching approximately 10% and 31% wild-type SAP protein, respectively (Figures S5C and S5D; Table S3).

To assess the viability of XLP1-SMART LVs as a gene therapy tool for XLP1 patients, we tested the XLP1-SMART LVs for their functional correction of XLP1 patient cells. Bone marrow (BM) CD34+ cells were obtained from XLP1 patients after their informed consent (UCLA IRB no. 10-001399). XLP1 patient CD34+ cells were pre-stimulated for 24 h in 50 ng/mL of human hSCF, hTPO, and hFT3L before transduction with the XLP1-SMART LVs. Cells were transduced to achieve equivalent VCNs of ∼1.1 and ∼2.4 (EFS = 1.1 and 3.0; E3M = 0.9 and 1.7; E3GP = 1.2 and 2.52) (based on prior dose-response testing of VCN produced by each LV across a range of concentrations). Transduced CD34+ cells were used to conduct a colony-forming unit (CFU) assay for progenitor cell clonogenic potential and were also differentiated to NK cells to assess XLP1 functional restoration via an NK cell cytotoxicity assay.

Transduced XLP1 patient cells were seeded in semi-solid methylcellulose medium and cultured for 14 days before quantifying vector effect on clonogenicity and generation of hematopoietic progenitor colonies (Figures 4A–4C). At a VCN of 1, XLP1-SMART LVs demonstrated no changes to clonogenicity or hematopoietic lineage skewing in comparison with a healthy donor control (Figures 4A–4C). Similarly, patient cells transduced with the EFS-SAP vector, at a VCN of 1.1, demonstrated no significant skewing ($p < 0.05$) into the myeloid lineage when compared with a healthy donor control (Figures 4B and 4C). At a VCN of 1.7, E3M-20R-5RL-SAP (3M-SAP) XLP1-SMART LVs still demonstrated no changes to clonogenicity or hematopoietic lineage skewing in comparison with a healthy donor control (Figures 4D–4F). Conversely, patient cells transduced with the EFS-SAP vector at a VCN of 3.0 demonstrated significant skewing ($p < 0.05$) into the myeloid lineage, with a 1.3-fold increase in granulocyte-macrophage (GM) colonies compared with a healthy donor control (Figures 4E and 4F). At a VCN of 2.52,

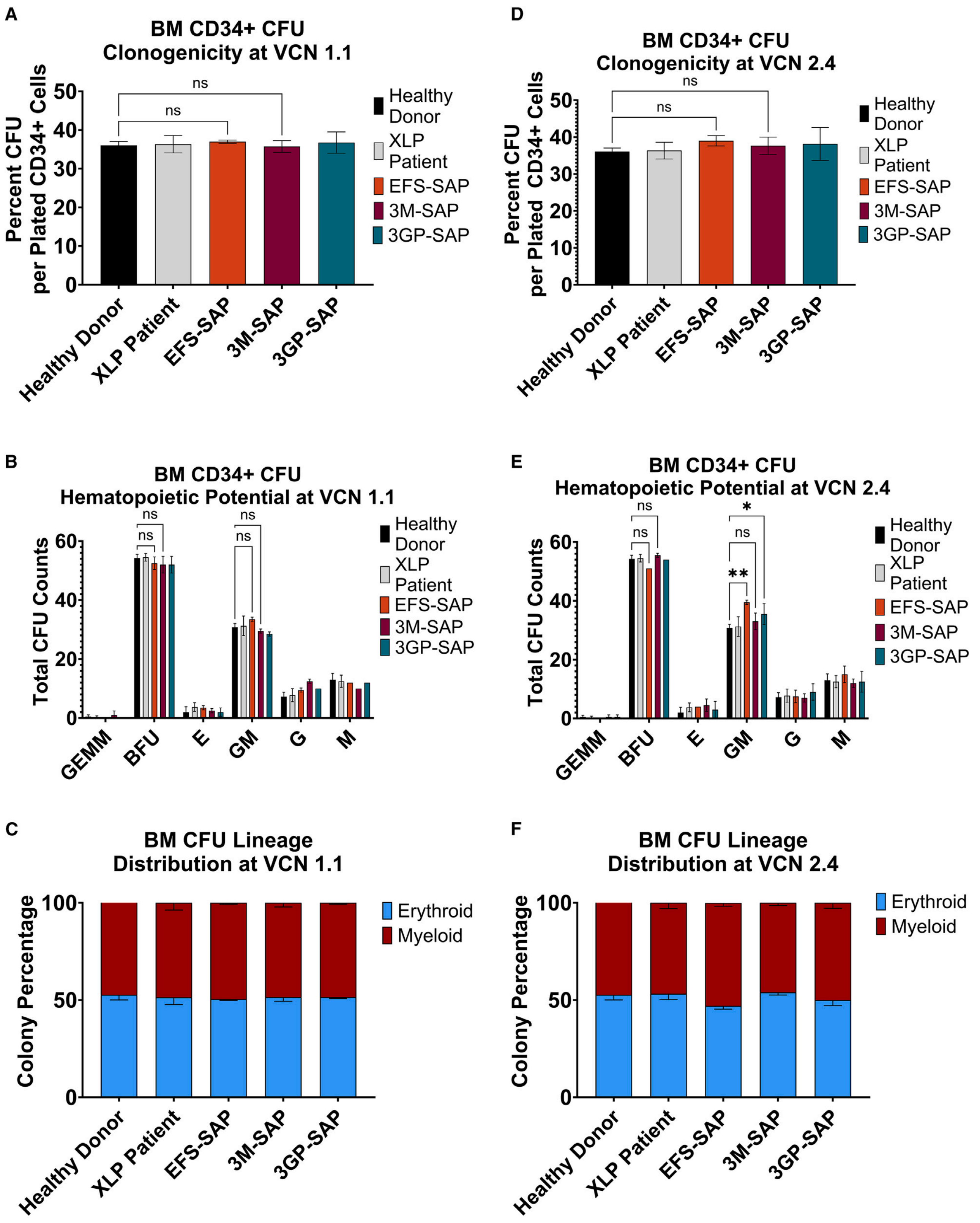

(legend on next page)

E3GP-20R-5RL-SAP (3GP-SAP) also demonstrated significant skewing into the myeloid lineage with a 1.15-fold increase in GM colonies compared with a healthy donor control (Figures 4E and 4F).

To measure levels of rescued SAP expression in XLP1 patient cells, we compared SAP expression after transduction by XLP1-SMART LVs to wild-type SAP expression from healthy donor CD8+ T cells via flow cytometry (Figure 5A). In brief, transduced XLP1 patient CD8+ T cells were fixed, permeabilized, and stained for SAP protein using an anti-SAP monoclonal antibody to assess the rescue of SAP protein expression via flow cytometry. At a VCN of 1, XLP1-SMART LVs restored SAP expression in XLP1 patient CD8+ T cells to healthy donor levels (Figure 5A).

It has also been demonstrated that patients with XLP1 are resistant to apoptosis-mediated T cell receptor (TCR) restimulation, as SAP expression is required for TCR-induced apoptosis.[38] As such, XLP1 patient peripheral blood CD8+ T cells were transduced with XLP1-SMART LVs at equivalent VCNs to assess functional restoration using a restimulation-induced cell death (RICD) assay.[39,40] Fourteen days post-transduction, transduced patient CD8+ T cells were quantified for recovery of RICD by flow cytometry. Transduced XLP1 patient T cells with the EFS-SAP, E3M-SAP, and E3GP-SAP vectors restored T cell RICD activity to healthy donor levels at an equal VCN of 1 (Figure 5B).

Healthy donor human CD8+ cells were also transfected using the CRISPR-Cas9 system to knock out the *SH2D1A* gene. Knockout T cells were transduced with XLP1-SMART LVs to assess their RICD activity. The E3M-20R-5RL-SAP LV at a VCN of 2 restored RICD activity to healthy donor levels. Conversely, EFS-SAP and E3GP-20R-5RL-SAP LV transduced *SH2D1A* knockout T cells did not restore T cell RICD activity to healthy donor levels at an equivalent VCN of 2 (Figure S6).

Transduced XLP1 patient BM CD34+ cells were also differentiated into NK cells and tested for functional recovery of NK cell cytotoxicity in a tumor killing assay with NK-sensitive target cells that express SLAM family receptors (Raji) and those that do not (K562).[41,42] As expected, MHC-independent killing was observed in K562 cells, with equal levels of killing of K562 by healthy donor and non-transduced/transduced XLP1 patient cells (VCN: EFS = 6, E3M = 0.9, and E3GP = 1.2) (Figure 5C). In a parallel assay with SLAM-expressing Raji cells, E3M-20R-5RL-SAP-transduced XLP1 patient BM CD34+ cells restored NK cell cytotoxicity to near healthy donor levels (VCN: EFS = 1, E3M = 1.7, E3GP = 2.52) (Figure 5D). Conversely, E3GP-20R-5RL-SAP- and EFS-SAP-transduced XLP1 patient BM CD34+ cells did not fully restore NK cell cytotoxicity to healthy donor levels (Figure 5D).

DISCUSSION

Autologous HSC gene therapy could provide a safe and effective curative treatment for XLP1 if methods can achieve precise, regulated SAP expression in the correct lineages, at therapeutic levels, at the right developmental stage. There are also far fewer risks with autologous gene therapy that hamper allogeneic hematopoietic stem cell transplantation (HSCT), such as graft versus host disease and graft rejection. For the treatment of XLP1, a gene therapy candidate that can restore appropriate *SH2D1A* gene expression after an autologous HSCT in the appropriate hematopoietic cell lineages is required since SAP expression is tightly regulated in T, NK, and NKT cells. There are various gene therapy efforts underway to target XLP1 including gene editing and viral vector-mediated approaches. Recently, a group from the University College London has attempted to combat XLP1 through gene editing, specifically through the use of TALEN, CRISPR-Cas9, and CRISPR-12a nucleases.[39] The benefit of gene editing for XLP1 is the ability to use endogenous regulatory elements of the *SH2D1A* gene to ensure the tight control necessary for appropriate SAP expression. However, the delivery of these gene-editing reagents utilizes an adeno-associated virus serotype 6-based vector, which can cause issues with immunogenicity and toxicity in treated patients.[43,44] Furthermore, the utilization of nucleases and the induction of double-stranded breaks (DSBs) is a safety-profile concern, specifically due to off-target effects, chromosomal translocation, and chromothripsis.[45,46] Even newer nuclease-based technologies that overcome these safety concerns such as base-editing and prime editing have disadvantages.[47] Base-editing is restricted to single-nucleotide changes, so diseases such as XLP1 with multiple mutations are ineligible for this treatment. Prime editing, although capable of inserting or deleting larger fragments of DNA without the need for DSBs, lacks the repair efficiency required to provide a therapeutic advantage. Consequently, the utilization of an LV-based therapy for XLP1 is a promising alternative that may offer a stronger safety profile, so long as the designed LV maintains the tight regulation of *SH2D1A*.

The use of LV gene therapy to treat XLP1 has been attempted previously.[11] However, this method utilized a ubiquitously active

Figure 4. Colony Forming Unit (CFU) Assay of XLP-SMART Lentiviral Vectors in Healthy Donor and XLP1 Patient Bone Marrow CD34+ Cells
BM CD34+ cells from a healthy donor and an XLP1 patient were prestimulated for 24 h with 50 ng/mL each of human stem cell factor (hSCF), human thrombopoietin (hTPO), and human FMS-like tyrosine kinase 3 ligand (hFlt3-L) before transduction with XLP1-SMART LVs. 24 h after transduction, 100, 300, and 900 BM CD34+ HSPCs per replicate were plated in MethoCult. After 14 days of culture at 5% CO_2, 37°C, and humidified atmosphere, the number of mature colonies were scored under the microscope for total colony-forming units (CFU) at a VCN of 1.1 (A) or 2.4 (D); total hematopoietic progenitor cell counts at a VCN of 1.1 (B) or 2.4 (E), denoted as myeloid (G/M/GM), erythroid (BFU/E), or mixed (GEMM); and, finally, percentage of total myeloid or erythroid lineage distribution for cells at a VCN of 1.1 (C) or 2.4 (F). Data are represented as mean ± SD of biological duplicates from one experiment. Clonogenicity was analyzed for statistical significance using a one-way ANOVA followed by multiple paired comparisons for normally distributed data (Tukey test). CFU hematopoietic potential was analyzed for statistical significance using a two-way ANOVA followed by multiple paired comparisons for normally distributed data (Tukey test). Statistical analysis was performed on all arms, but selected arms are shown. All statistical tests were two-tailed and a p value of <0.05 was deemed significant (ns, non-significant;*$p < 0.05$, **$p < 0.01$, ***$p < 0.001$, ****$p < 0.0001$).

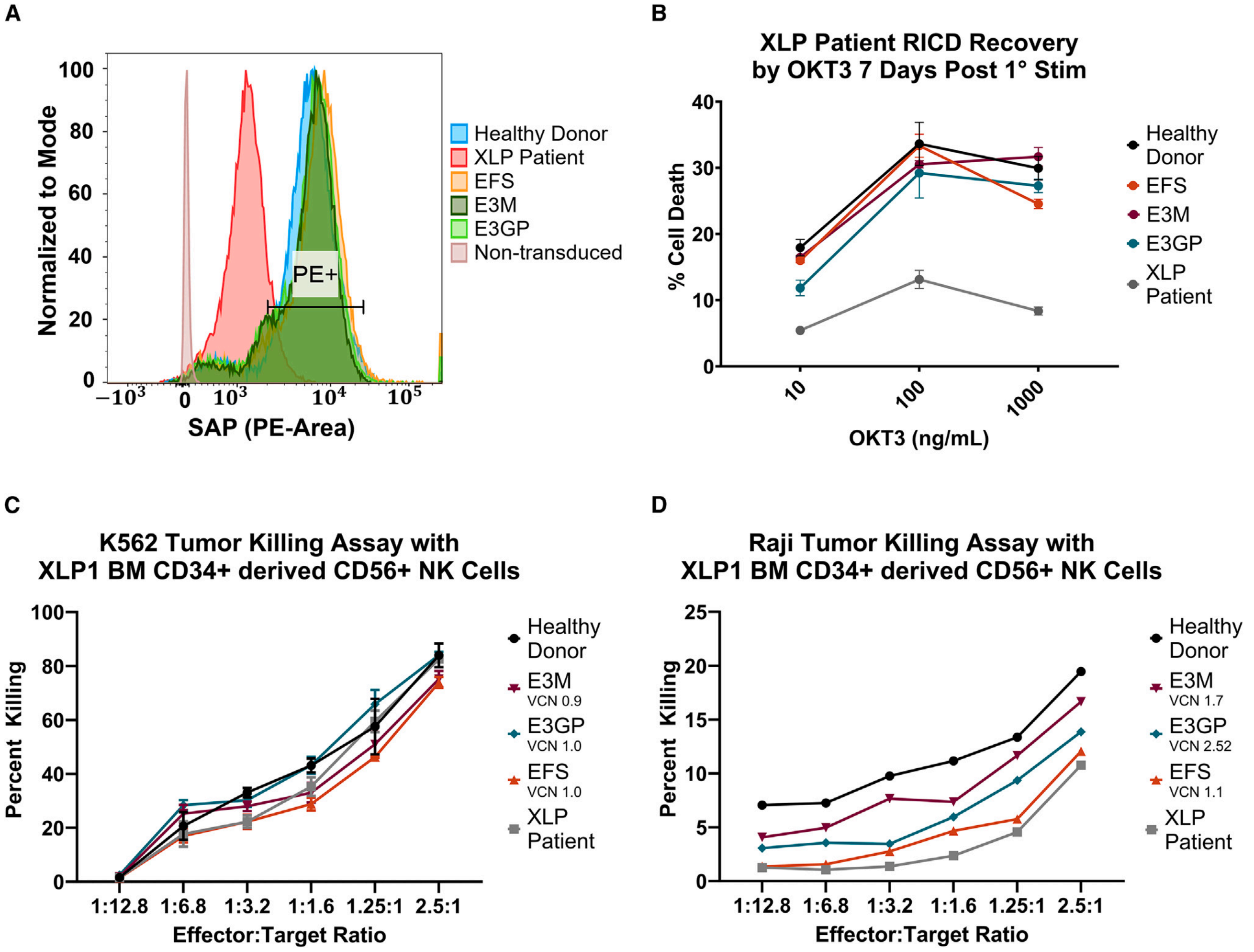

Figure 5. Functional Restoration and Cytotoxic Activity of XLP1-SMART Lentiviral Vectors in CD8+ T Cells and NK Cells from XLP1 Patients

(A) FACS representation of SAP protein restoration in XLP1 patient CD8+ T cells. CD8+ T cells from a healthy donor (HD) and an XLP1 patient were isolated from PBMCs and transduced with XLP-SMART LVs. Ten days after transduction, cells were fixed, permeabilized, and stained for SAP protein using an anti-SAP monoclonal antibody. Stained cells were then assessed for their SAP expression via total SAP MFI within each target subpopulation using flow cytometry. (B) T cell restimulation-induced cell death (RICD) assay of XLP1 patient CD8+ T cells transduced with XLP-SMART LVs. CD8+ T cells from a HD and an XLP1 patient were isolated from PBMCs and transduced with XLP-SMART LVs. Ten days after transduction, cells were plated for RICD assay in OKT3 at final concentrations of 1,000, 100, and 10 ng/mL. After 24 h, XLP-SMART LV transduced cells were taken to measure the recovery of RICD in comparison with an EFS-SAP transduced condition and an HD control. The number of live cells (PI–) in stimulated controls were compared with unstimulated controls to measure the percent cell loss = [1 – (no. of PI– restimulated cells/no. of PI– untreated cells)] $\times$ 100. Data are represented as mean ± SD of biological triplicates from one experiment. Statistical significance was analyzed using a two-way ANOVA followed by multiple paired comparisons for normally distributed data (Tukey test). All statistical tests were two-tailed and a p value of <0.05 was deemed significant (ns, non-significant; *$p < 0.05$, **$p < 0.01$, ***$p < 0.001$, ****$p < 0.0001$). Compared with SH2D1A knockout (KO) T cells, the EFS, E3M, and E3GP conditions were deemed significant with a p value < 0.0001 (data not shown). (C) K562 NK cell cytotoxicity assay of XLP1 patient BM CD34+ cells transduced with XLP-SMART LVs. BM CD34+ cells from a HD and an XLP1 patient were transduced with XLP-SMART LVs and differentiated into CD56+ NK cells using the StemSpan NK Cell Generation Kit. On day 28 of differentiation, CD56+ NK cells were enriched using magnetic bead isolation and serially diluted with target cells at various effector to target (K562) ratios: 2.5:1, 1.25:1, 1:1.6, 1:3.2, 1:6.8, and 1:12.8. After 18 h of incubation, GFP+ tumor cells were counted via FACS to assess NK cytotoxicity and normalized to target only control wells. Due to limited XLP1 patient cells, data are represented as single replicates from one experiment. Statistical significance was analyzed using a two-way ANOVA followed by multiple paired comparisons for normally distributed data (Tukey test). All statistical tests were two-tailed and a p value of <0.05 was deemed significant (ns non-significant; *$p < 0.05$, **$p < 0.01$, ***$p < 0.001$, ****$p < 0.0001$). Compared with XLP1 patient samples, Healthy donor was deemed significant at a p value < 0.05; EFS was deemed significant with a p value < 0.001; E3M was deemed not significant; and E3GP was deemed significant with a p value < 0.0001. (D): Raji NK cell cytotoxicity assay of XLP1 patient BM CD34+ cells transduced with XLP-SMART LVs. BM CD34+ cells from a HD and an XLP1 patient were transduced with XLP-SMART LVs and differentiated into CD56+ NK cells using the StemSpan NK Cell Generation Kit. On day 28 of differentiation, CD56+ NK cells were enriched using magnetic bead isolation and serially diluted with target cells at various effector to target (Raji) ratios: 2.5:1, 1.25:1, 1:1.6, 1:3.2, 1:6.8, and 1:12.8. After 18 h of incubation, 7AAD+ CFSE+ tumor cells were counted via FACS to assess NK cytotoxicity and normalized to target only control wells. Due to insufficient patient cells, data are represented as single replicates from one experiment. Statistical significance was analyzed using a two-way ANOVA followed by multiple paired comparisons for normally distributed data (Tukey test). Compared with XLP1 patient samples, EFS was deemed not significant; E3M was deemed significant with a p value < 0.0001; and E3GP was deemed significant with a p value < 0.0001.

elongation factor 1 α short promoter to drive expression of the SAP protein (EFS-SAP). One concern with this approach lies in the non-physiological expression of SAP in all hematopoietic cell populations. Since SAP expression is tightly regulated within T, NK, and NKT cells, gene expression in off-target cell populations such as myeloid and B cells may pose safety concerns, including improper signaling of factors associated with autoimmune diseases and an elevated apoptotic response to DNA damage.[23,48] Previous researchers have demonstrated significant skewing induced by the EFS-SAP vector of hematopoietic potential in the bone marrow compartment via a CFU assay.[8,11] As a result, we rationalized that an XLP1 LV therapy requires the incorporation of endogenous enhancer elements to regulate the expression of the *SH2D1A* transgene.

We implemented a bioinformatics-guided approach to develop highly regulated LVs driven by endogenous regulatory elements of the *SH2D1A* gene. Analysis of the topologically associated domain of *SH2D1A* revealed three genomic elements responsible for the physiological expression pattern of the *SH2D1A* gene. Element 5R, located 3 kb directly downstream of the *SH2D1A* promoter, was shown to be a T-, NK-, and NKT-specific enhancer, lacking activity in B cell and myeloid cell lineages. Chromatin immunoprecipitation sequencing (ChIP-seq) data from ENCODE revealed various lymphoid-associated transcription factor binding sites such as MEIS2, TAL1, and SPI1 within element 5R.[28] These transcription factors are known to regulate expression of NK cell-activating ligands, T cell decision fate, and homeostasis of lymphoid cells.[49,50] Element 3, located 125 kb upstream of the *SH2D1A* promoter, presented as a T-, NK-, and NKT-specific enhancer, lacking activity in B cell and myeloid cell lineages. ChIP-seq transcription factor analysis revealed various lymphoid-associated transcription factor binding sites within element 3, such as FOXA1, RUNX3, GATA2, and JUND. These transcription factors are important in T cell development, expression of T and NK cytotoxic lymphocytes, proliferation and maintenance of HSPCs, and modulation of expression of T cell differentiation and activation.[51] Finally, element 20, located within intron 1 of the *SH2D1A* gene, presented as an NK- and NKT-specific enhancer lacking activity in T cell, B cell, and myeloid cell lineages. ChIP-seq transcription factor analysis revealed various lymphoid-associated transcription factor binding sites within element 20, such as SPI1, GATA2, and BRD4. These transcription factors are known to regulate lymphocyte homeostasis and HSPC differentiation, proliferation, and maintenance.[49,51,52] Taken together, these three enhancers can recapitulate the expression of SAP in all physiologically expressed lineages.

The therapeutic potential of an LV is greatly influenced by both gene transfer and expression. Enhancing gene transfer not only increases the percentage of transduced cells, but also results in more integrated LV copies within each cell, amplifying the overall cellular expression. Conversely, boosting expression directly results in a higher production of the therapeutic protein for every integrated copy. Previous studies have also demonstrated a negative correlation between titer/gene transfer and proviral length.[26] To address this, we made systematic deletions in each of the genomic elements to reduce the proviral length of our vector. As a result, we observed a 5- to 10-fold enhancement in titer and gene transfer.[26,27] Enhancer E3M was further reduced in size from 1.9 to 1 kb to generate the E3GP enhancer. These reductions in enhancer element lengths increased titer by 2-fold and gene transfer by 1.5-fold. Such improvements in titer and gene transfer are of significant value for the clinical application of gene therapy because they substantially decrease the cost of vector production and reduce the volume of LV production lots needed for each patient's treatment. With these modifications, there is a potential concern about the regulation and expression being compromised. However, even after these adjustments, over 90% of the intended MFI was maintained, and any unintended activity remained minimal. Further modifications to refine the enhancer elements and retain vector expression can be made.

We next strung multiple enhancers together within a single LV to measure the additive effects on mCitrine expression in on-target and off-target cells. While enhancer 3 demonstrated minimal off-target expression in B-LCLs and monocytes, when combined with enhancer 5RL alone or 5RL-20R, off-target expression was abrogated, most likely a result of repressor elements within the 5R enhancer region. This result was further confirmed in our *in vivo* studies, in which the XLP1-SMART LVs contained no significant off-target expression when compared with the promoter-only control in both the bone marrow and peripheral blood compartments. The effects of this decrease in off-target expression warrants further studies in SAP-deficient mouse models.

Using reporter gene experiments in transduced human CB CD34+ cells that were then transplanted into NSG-Tg(Hu-IL15) neonates, we observed that the XLP1-SMART LVs were predominantly expressed in mature human T, NK, and NKT lineages. Importantly, there was no detectable expression in the B and myeloid cell lineages. Furthermore, the E3GP-20R-5RL XLP1-SMART LV exhibited lower levels of mCitrine expression in T, NK, and NKT cells compared with the E3M-20R-5RL XLP1-SMART LV.

We next assessed if the XLP1-SMART LVs will affect hematopoietic skewing. While the E3M-20R-5RL-SAP XLP-SMART LV demonstrated no lineage skewing at VCNs of 1 and 1.7, the EFS-SAP LVs illustrated significant HSC skewing into the myeloid lineage, further indicating the potential adverse effects of ectopic SAP expression within hematopoietic cells. The lineage skewing demonstrated in the CFU assay may be attributed to the increased SAP expression within off-target lineages. The skewing into the myeloid lineage of the E3GP-20R-5RL XLP-SMART LV may be attributed to the differences in enhancer sequences of enhancer 3. Alternatively, the lack of skewing in the E3M-20R-5RL-SAP XLP-SMART LV may be due to VCN differences between that sample and the EFS and E3GP counterparts (EFS = 3, E3M = 1.7, E3GP = 2.52). Further studies are necessary to determine the cause of this skewing and its effects on the XLP1 phenotype.

The dysregulated immune response seen in XLP1 patients can be attributed to their reductions in T cell function and NK cell

cytotoxicity. We began by assessing the XLP-SMART LVs efficacy in an alternative disease model: SH2D1A −/− Jurkat cell lines. Our dose response, ranging from a VCN of 1 to 5, demonstrated that at VCN of 3 and 5, the E3M and E3GP vectors reached ~30% of wild-type SH2D1A protein levels. Future studies are required to determine if this decrease in SH2D1A protein expression will affect function and treatment outcomes. That said, it is possible that wild-type levels are not necessary to alleviate the XLP1 phenotype, as evidenced by the fact that female carriers of XLP are healthy.[53]

We then looked to assess the ability of XLP1 patient cells transduced with XLP-SMART LVs to restore T cell and NK cell function to healthy donor levels. E3M-20R-5RL-SAP (E3M)- and EFS-SAP-transduced XLP1 patient CD8+ T cells restored RICD activity to HD levels at an average VCN of 1; in contrast, studies conducted in healthy donor samples—in which the *SH2D1A* was knocked out with CRISPR-Cas9 technology—demonstrated larger discrepancies of RICD restoration between the EFS, E3M, and E3GP vectors at equal VCN (e.g., EFS and E3GP vectors were not capable of restoring RICD levels to healthy donor levels at a VCN of 2). While these variations may be due to donor variability, further studies in XLP1 patient T cells or in a viable XLP1 *in vivo* model are necessary to confirm the E3M or EFS vector's ability to restore T cell RICD function.

When assessing restoration of NK cytotoxic activity in XLP1 patient cells, the XLP1-SMART LVs demonstrated greater cytolytic activity than the EFS-SAP vector. This phenotype is likely due to the increased SAP expression in NK cells with the E3M/GP, E5RL, and E20R enhancers. The reduction of NK expression in the E3GP sample compared with E3M may be due to core transcription factor binding sites and epigenetic modifications that were removed in the interest of decreasing vector size for an increased titer and gene transfer. The lack of EFS-SAP vector NK cytolytic activity is potentially a result of reduced SAP expression within NK cells due to a lower VCN seen in the EFS-transduced populations compared with the E3M and E3GP counterparts (VCNs were EFS = 1.1, E3M = 1.7, E3GP = 2.52). The limited access to more XLP1 patient CD34+ cells and PBMCs prevented repeated studies at equal VCNs. Future *in vivo* studies utilizing the C57BL/6 *SH2D1A*−/− mouse model will help elucidate the ability for XLP-SMART LVs to restore the humoral defects and the NKT developmental block seen in XLP1 patients.

While future studies are needed to assess the safety and efficacy of the XLP-SMART LVs (e.g., *in vitro* immortalization assay and C57BL/6 *SH2D1A*−/− mouse studies), the outcomes of this study have elucidated a streamlined approach for the identification and incorporation of key elements into a vector cassette that can properly regulate a target gene (SMART LVs). The rational generation of regulated LVs offers a novel, effective, and efficient approach for enhanced expression and regulation of transgenes required to correct various inborn errors of immunity, as evidenced by these highly specific XLP-SMART LVs for the treatment of XLP1. In conjunction with these studies, the regulated expression by enhancers 3, 20, and 5R within the T, NK, and NKT lineages may provide a useful approach toward a lentiviral gene therapy for hemophagocytic lymphohistiocytosis (HLH) disorders, such as perforin deficiency, which share similar expression profiles and regulation to that of XLP1. Ultimately by iterative process, we developed two candidate vectors that express SAP protein in a lineage- and stage-specific manner at levels similar to the endogenous *SH2D1A* gene.

MATERIALS AND METHODS

Elucidation of putative enhancer elements

Genomic regions containing putative regulatory elements of the *SH2D1A* gene were compiled using data from ENCODE, Ensembl, FANTOM, and VISTA. Functional boundaries of the putative enhancer elements were defined using lineage-specific DNase I accessibility, transcription factor binding, epigenetic histone modification, and vertebrate sequence conservation. Primers were then designed to amplify these putative enhancer regions from human genomic DNA for downstream cloning into plasmid lentiviral transgene cassettes.

Vector packaging and titration

Lentiviruses were packaged by transient transfection of PKR−/− 293T cells with fixed amounts of HIV Gag/Pol, Rev, and VSV-G envelope expression plasmids and equimolar amounts of transfer plasmid using TransIT-293 (Mirus Bio, Madison, WI) as described in Cooper et al.[30,54] Viral supernatants were then directly used for titer determination or concentrated by tangential flow filtration. To titer the lentivirus, 1×10^5 HT-29 cells per sample were plated in 2 mL of culture medium in 6-well plates (no. 3516; Corning, Corning, NY). Twenty-four hours after plating, cells were transduced with a 1:10 dilution of viral supernatant in 1 mL of culture medium. Twenty-four hours after transduction, culture medium was refreshed on all wells. Seventy-two hours after transduction, cells were harvested to determine VCNs by ddPCR. Vector titer (TU/mL) was calculated as TU = VCN × (cell count at day of transduction) × virus dilution. Cell counts were measured with a Vi-CELL XR automated cell counter (Beckman Coulter, Brea, CA).

LV transduction

Primary human T, NK, and NKT cells (5×10^4 per sample) isolated from PBMCs were plated on retronectin (20 μg/mL) (Takara Bio, Kusatsu, Shiga, Japan)-coated plates with XVIVO15 medium (Lonza Biosciences, Basel, Switzerland) supplemented with 1 mg/mL poloxamer synperonic F108 (Kolliphor P338; BASF Pharma, Ludwigshafen, Germany) for 24 h during lentiviral transduction. Cell counts were measured with a Vi-CELL XR automated cell counter. For transduction of CD34+ cells, cells were prestimulated with 50 ng/mL each of human stem cell factor (hSCF), human thrombopoietin (hTPO), and human FMS-like tyrosine kinase 3 ligand (hFlt3-L) (Peprotech, Rocky Hill, NJ) for 24 h before lentiviral transduction. During LV transduction of CD34+ cells, 10 μM prostaglandin E2 (PGE2) was also added for 24 h. LV supernatant (raw or concentrated) was added to the culture medium for 24 h at various concentrations to achieve the necessary VCN.

ddPCR for VCN and titer quantification

Genomic DNA from transduced cells was extracted using a PureLink Genomic DNA Mini Kit (K182002; Invitrogen, Waltham, MA). VCN was calculated by using the vector HIV-1 PSI gene primers (oPAF-PSI, oPAR-PSI) and probes (oPAP-PSI) and an endogenous human diploid gene control (SCD4; Human Syndecan 4) primers (oPAF-SDC4 and oPAR-SDC4) and probe (oPAP-SDC4) as a reference (Table S4). Reaction mixtures of 22 μL volume, comprising 1× ddPCR Master Mix (no. 1863010; Bio-Rad, Hercules, CA), 400 nmol/L primers, and 100 nmol/L probe for each set, 40 U DraI (R0129S; New England Biolabs, Ipswich, MA) and 30–100 g of the gDNA were prepared and incubated at 37°C for 1 h. Droplet generation was performed as described in Hindson et al. with 20 μL of each reaction mixture.[55] The droplet emulsion was then transferred with a multichannel pipette to a 96-well twin.tec real-time PCR Plate (Eppendorf, Hamburg, Germany), heat sealed with foil, and amplified in a conventional thermal cycler (T100 Thermal Cycler; Bio-Rad). Thermal cycling conditions consisted of 95°C for 10 min (1 cycle), 94°C for 30 s, and 60°C for 1 min (55 cycles), 98°C for 10 min (1 cycle), and 12°C hold. After PCR, the 96-well plate was transferred to a droplet reader (Bio-Rad). Acquisition and analysis of the ddPCR data was performed with the QuantaSoft software (Bio-Rad), provided with the droplet reader. Vector titer (TU/mL) was calculated as TU = VCN × (cell count at day of transduction) × virus dilution.

NSG-Tg(hu-IL15) xenografts

Transduced human CB CD34+ cells were washed and incubated with 1 μg/100 μL of OKT3 (Tonbo Biosciences, San Diego, CA) for 30 min at 4°C to prevent contaminating T cell-derived graft-versus-host disease. Immediately before transplant, 1- to 3-day-old neonatal NSG-Tg(hu-IL-15) mice (NOD.Cg-*Prkdc*^{scid} *Il2rg*^{tm1Wjl} Tg(IL15)1Sz/SzJ, strain no. 030890; The Jackson Laboratory, Bar Harbor, ME) were irradiated at a dose of 150 rads with a cesium-137 source. Each mouse was injected intrahepatically with 1×10^5 to 5×10^5 cells. At 16 weeks post-transplant, mouse bone marrow, spleen, thymus, and peripheral blood were harvested into single-cell suspensions for downstream flow cytometry analysis. The mice were maintained at UCLA under an approved protocol by the UCLA Animal Research Committee under the Division of Laboratory Medicine.

Plasmid generation

All LVs were cloned into an empty pCCL backbone.[56] Fragments of transcriptional regulatory elements were synthesized as gBlocks (Integrated DNA Technologies, Coralville, IA) or amplified from genomic DNA by polymerase chain reaction with compatible ends to be cloned using an NEBuilder HiFi DNA Assembly Kit (New England Biolabs). Enhancer elements were inserted upstream of a 600 bp *SH2D1A* promoter to drive expression of the SAP or an mCit reporter cassette. Furthermore, each vector contains the WPRE in replacement of the endogenous *SH2D1A* 3′ UTR.

Cell culture

CD34+ cells were cultured in X-VIVO15 medium (Lonza Biosciences) supplemented with 1× penicillin-streptomycin-glutamine (P/S/G), with 50 ng/mL each of hSCF, hTPO, and hFlt3-L for 24 h before LV transduction. CD3+ T cells were isolated from PBMCs through CD3+-positive magnetic selection (Miltenyi Biotec, Bergisch Gladbach, Germany). Isolated cells were then activated using anti-CD3/CD28 Immunocult (STEMCELL Technologies, Vancouver, Canada) and cultured in X-VIVO15 medium (Lonza Biosciences), 5% human serum, and 100 U/mL of human recombinant IL-2. NKT cells were isolated from PBMCs through iNKT Vα24+-positive magnetic selection (Miltenyi Biotec). NKT cells were cultured in RPMI 1640, 1% P/S, 10% fetal bovine serum (FBS), 1% MEM non-essential amino acids, 10 mM HEPES, 1 mM sodium pyruvate, and 50 μM 2-mercaptoethanol. NKT cells were stimulated at a ratio of 1:1 with autologous PBMCs loaded with 5 ng/mL of α-GalCer (Cayman Chemical, Ann Arbor, MI, cat, no. 158021-47-7) and irradiated at 6,000 rpm. The cocultured cells were subsequently cultured with 10 ng/mL of IL-15 and IL-7. NK cells were enriched from PBMCs using the CD56+-positive magnetic selection (Miltenyi Biotec). Isolated NK cells were cultured in NK MACS medium (Miltenyi Biotech) supplemented with 5% human AB serum, 1% P/S, 10 ng/mL hIL-15, and 500 U/mL of hIL-2 at 5% CO_2 and 37°C humidified atmosphere.

ATO generation

ATOs were generated from human mPB stem cells as described previously.[34,35] Previously frozen MS5-hDLL4 cells were thawed and resuspended in serum-free ATO culture medium (RB27) composed of RPMI 1640 (Corning, Manassas, VA), 4% B27 supplement (Thermo Fisher Scientific, Grand Island, NY), 30 μM L-ascorbic acid 2-phosphate sesquimagnesium salt hydrate (Sigma-Aldrich, St. Louis, MO) reconstituted in PBS, 1% P/S (GeminiBio products, West Sacramento, CA), and 2% GlutaMAXx (Thermo Fisher Scientific). RB27 lasts 3 weeks at 4°C. MS5-hDLL4 cells and CD34+ mPB cells edited with XLP1-SMART LVs or mock control were combined in Eppendorf tubes at a concentration of 150k MS5-hDLL4 and 5,000 mPB per ATO to make 24 ATOs per group. Cells were centrifuged at 300 × *g* for 5 min at 4°C in a swinging bucket centrifuge. Supernatants were carefully removed, and the cell pellet was resuspended in 5 μL RB27 per ATO and mixed by brief vortexing. ATOs were plated on 0.4 μm Millicell transwell inserts (EMD Millipore, Billerica, MA, cat. no. PICM0RG50) in 6-well plates containing 1 mL complete RB27 (RB27 with the addition of 5 ng/mL rhFLT3L, 2.5 ng/mL rhIL-7, and 5 ng/mL hSCF (Peprotech) per well. Two ATOs were plated per insert. Medium was changed completely every 3–4 days by aspiration from around the cell insert followed by replacement with 1 mL of fresh RB27/cytokines. hSCF is only added for the first week of culture. ATOs are kept in an incubator at 37°C with 5% CO_2. At weeks 3, 7, and 12, six ATOs (three inserts) per group per time point were harvested by adding FACS buffer (PBS/0.5% bovine serum albumin/2 mM EDTA) to each well and briefly disaggregating the ATO by pipetting, followed by passage through a 50 μm nylon cell strainer. Cells were stained in 96-well plates. At weeks 3 and 7, 200k cells per ATO were stained without fixation to determine mCitrine levels and T cell differentiation kinetics, and the remaining cells (no more than 2 million cells per stained sample) were fixed and

permeabilized to assess intracellular SAP expression and T cell differentiation. At week 12, half of the ATO cells were stained without fixation and half were stained with fixation (no more than 2 million cells per stained sample).

Acquisition of XLP1 patient cells

XLP1 patient cells were acquired after patient and parental informed consent (UCLA IRB no. 10-001399). Collection of BM CD34+ cells after bone marrow aspirate and acquisition of PBMCs from peripheral blood were procured under Institutional Review Board-approved protocol at the David Geffen School of Medicine at UCLA, protocol no. 10-001399. The XLP1 patient harbored a pathogenic missense mutation in exon 1 of the *SH2D1A* gene.

CFU assay

One hundred, 300, and 900 BM CD34+ HSPCs per replicate were plated in MethoCult (cat. no. 04445; STMECELL Technologies) 24 h after LV transduction. After 14 days of culture at 5% CO_2, 37°C, and humidified atmosphere, the number of mature colonies were counted and scored under the microscope based on their specific morphology.

Generation of SH2D1A knockout cell lines

To generate an XLP1 model cell line, Jurkat T cells were modified to knockout *SH2D1A* by electroporation of SpCas9 recombinant protein (QB3 Macrolab; UC Berkeley, Berkeley, CA) complexed to sgRNA (Table S5) (Synthego, Redwood City, CA) and FACS single-cell sorted and cultured in R20 (RPMI 1640 [Gibco, Grand Island, NY]/20% FBS [Gibco]/1× P/S/G [GeminiBio products]). Primers for amplification of the *SH2D1A* locus were used to confirm knockout (oPAF605 [TCCTATGAATGCAATGACACCA] and oPAR340 [TGTGGCA ATTTTCAGGAGTTCAC]) by Synthego ICE. Absence of SAP expression was confirmed by western blot analysis. Cells were cultured in R10 at 37°C with 5% CO_2.

Western blot

For immunoblots, cells were lysed in RIPA lysis and extraction buffer (cat. no. 89901; Thermo Fisher Scientific) with added HALT protease inhibitor (cat. no. 87786; Thermo Fisher Scientific) at a 1× concentration following the manufacturer's protocols. Lysate concentrations were determined using the Pierce BCA protein assay (cat. no. 23227; Thermo Fisher Scientific) following the manufacturer's protocol. Samples were treated for sodium dodecyl sulfate-polyacrylamide gel electrophoresis with NuPAGE LDS Sample Buffer (cat. no. NP0007; Thermo Fisher Scientific) and NuPAGE Sample Reducing Agent (cat. no. NP0009; Thermo Fisher Scientific), each to a 1× concentration. Lysates were diluted to contain equivalent amounts of total protein for immunoblot gel loading, using lysate from the SAP-deficient Jurkat cells to keep the total amount of protein loaded per lane constant to allow for valid loading controls. Wild-type Jurkat lysate was used as a control to indicate the relative expression levels of the *SH2D1A* codon optimized XLP1 LVs. SAP levels were detected using Abnova monoclonal antibody, clone 1C9 (cat. no. H00004068-M01). Protein quantification was assessed through densitometry via the ImageJ software. SAP protein levels were normalized to the actin protein levels after quantification.

Flow cytometry

Intracellular staining of SAP was performed using the eBioscience Foxp3/Transcription Factor Staining Buffer Set (Invitrogen) using the manufacturer's protocol. The primary antibody was rat anti-human SAP antibody, PE (cat. no. 12-9787-42; Invitrogen). To discern the various hematopoietic cell lineages, cell populations were gated as follows: NK cell: CD56+, CD16−; NKT: CD3+, CD56+; iNKT: CD3+, TCR Va24+, CD56+; B cell: CD19+, CD3−; T cell: CD3+, CD19−, CD4+/CD8+; monocytes: CD33−, CD16−, CD14+. To discern the various T cell subpopulations and thymocyte populations in ATOs, we used the following monoclonal antibodies for staining: hCD45, hCD56, hCD34, hCD5, hCD7, hTCRab, hCD4, hCD8a, hCD8b, hCD3, hCD45RA, and hCD45RO. To discern the various NK cell subpopulations, we used the following monoclonal antibodies for staining: hCD122, hCD16, hCD117, hCD45, hNKP80, hCD56, hCD34, hCD94, and hCD57. Live/dead dyes used for staining include DAPI and Zombie Fixable Viability Dye (BioLegend, San Diego, CA) for unfixed and fixed cells, respectively. See supplemental figures for more information (Table S6).

Next-generation sequencing library preparation

Fourteen days after transduction, genomic DNA and mRNA were harvested from the primary T cells, primary NK cells, primary NKT cells, and the B-LCLs. For library preparation, an initial PCR was completed to amplify the barcodes (Table S7) using primers oPAF255 – XLP Barcode Amp for NGS (Table S8) and oPAR119 – XLP Barcode Amp for NGS (Table S8). A second PCR was completed to add Illumina adapters and indexes. Following Illumina barcoding, PCR products were pooled at equal concentrations, purified twice using AMPure XP beads (Beckman Coulter), and then quantified by ddPCR (QX 200; Bio-Rad). The high-throughput sequencing was performed at the UCLA Technology Center for Genomics & Bioinformatics (TCGB) using an Illumina MiSeq instrument 2 × 150 paired-end reads (Illumina, San Diego, CA). For flow cytometry gating strategies, please see Figures S7–S17.

RICD

RICD was assessed using an established protocol.[40,57] LV modified or unmodified T cells were cultured for 10 days, before plating at 5×10^4 cells/well in a 96-well plate in 100 μL medium. Dilutions of OKT3 antibody (Tonbo Biosciences, San Diego, CA) were prepared at 2,000 , 200, and 20 ng/mL, and 100 μL added to the cells to make final concentrations of 1,000, 100, and 10 ng/mL OKT3 in the wells. After 24 h, a final concentration of 1 μg/mL of propidium iodide (PI) was added before running a fixed volume of cell suspension from each well for flow cytometry. The numbers of live cells (PI−) in stimulated controls were compared with unstimulated controls to measure the percent cell loss = [1 − (no. of PI− restimulated cells/no. of PI− untreated cells)] × 100.

NK differentiation

Untransduced or transduced healthy donor and XLP1 patient BM CD34+ cells were differentiated into CD56+ NK cells over 28 days of culture using the StemSpan NK Cell Generation Kit (cat. no. 09960; STEMCELL Technologies) following the manufacturer's protocol. BM CD34+ cells (1×10^4) were plated on non-tissue culture-treated plates coated with StemSpan Lymphoid Differentiation Coating Material. Half-volume medium changes were performed using StemSpan Lymphoid Progenitor Expansion Medium until day 14. The cells were then replated at 1×10^5 cells/mL in StemSpan NK Cell Differentiation Medium and cultured for another 14 days. On day 28, the cells were harvested and enriched using CD56+-positive magnetic selection (Miltenyi Biotec). The differentiated NK cells were then assessed for cytotoxic capabilities in further assays.

NK killing assay

BM CD34+ cells differentiated into CD56+ NK cells were counted and resuspended in NK medium at 5×10^4 cells per 200 μL and added to row B on a 96-well plate. One hundred microliters of NK medium was added to rows C through G. Six serial dilutions were performed of the resuspended NK cells using 100 μL multichannel pipette from rows B to G (from 2.5×10^4 cells per well to 800 cells per well). Target cells were either K562 cells expressing one copy of a GFP reporter cassette or Raji cells labeled with CellTrace CFSE (C34554; Invitrogen) resuspended in R10 (RPMI 1640, 10% FBS, 1% P/S/G) at 1×10^4 cells per 100 μSL. Of the target cells, 100 μL was added to rows A through G to generate the following effector to target ratios: 2.5:1, 1.25:1, 0.625:1, 1:3, 1:6, and 1:12.5. The plate was incubated for 18 h at 5% CO_2, 37°C. After incubation, GFP+ tumor cells were counted via FACS using a BD FACSCelesta Cell Analyzer (BD Biosciences, Franklin Lakes, NJ) and normalized to target only control wells (rows A and G) to normalize the killing percentage.[41]

Monocyte differentiation

CB CD34+ HSCs were cultured in X-VIVO15 medium (Lonza Biosciences), 4% FBS (Gibco), 1× P/S/G (GeminiBio products), 50 ng hSCF, 15 ng/mL hTPO, 30 ng/mL hIL3, and 30 ng/mL hFlt3-L (all cytokines: Peprotech) for 9 days. After 9 days of culture, the CB CD34+ cells were differentiated in the following medium for 7 days: STEMSpan II (STEMCELL Technologies), 20% FBS (Gibco), 1× P/S/G (GeminiBio products), 25 ng hSCF, 30 ng/mL M-CSF, 30 ng/mL hIL3, and 30 ng/mL hFlt3-L (all cytokines: Peprotech). On day 16 of culture, cells were confirmed for NK differentiation and purity by flow cytometry using anti-CD14 and anti-CD16 antibodies.

T cell electroporation to knock out SH2D1A

After isolation from PBMCs, CD3+ primary T cells were counted by hemocytometer via trypan blue exclusion prior to electroporation. Per condition, 1×10^6 cells were centrifuged at $300 \times g$ for 10 min at room temperature (RT), resuspended in 20 μL of P3 electroporation buffer (Lonza Biosciences). SpCas9 recombinant protein (100 pmol) (QB3 Macrolab, UC Berkeley) was combined with 120 pmol of each sgRNA to *SH2D1A* (sgRNA4: 5′-GACGCAGTGGCTGTG TATCA-3'; sgRNA7: 5′-AACAGGTTCTTGGAGTGCTG-3′ both from Synthego, Redwood City, CA) for 15 min at RT for RNP complex formation. The cell and RNP mixtures were combined and electroporated using the EH-100 setting on the Amaxa 4D Nucleofector X Unit (Lonza Biosciences). Cells were rested in 16-well electroporation strips (Lonza Biosciences) for 10 min at RT and then recovered with 480 μL of T cell medium. Twenty-four hours after electroporation, *SH2D1A* knockout T cells were transduced with XLP-SMART LVs and used for an RICD assay 10 days post-electroporation.

Statistical analysis

All data are reported as mean ± SD unless otherwise stated. All statistical analyses were carried out using GraphPad Prism version 10.0.0 (GraphPad Software, San Diego, CA). The statistical significance between two averages was established using unpaired t tests. When the statistical significance between three or more averages was evaluated, a one-way ANOVA was applied, followed by multiple paired comparisons for normally distributed data (Tukey's test). When the statistical significance between two or more categorical variables was evaluated, a two-way ANOVA was applied, followed by multiple paired comparisons for normally distributed data (Tukey's test). Linear regression analyses were used to determine the correlation between titer and proviral size. All statistical tests were two-tailed and a p value of <0.05 was deemed significant (ns, non-significant; $*p < 0.05$, $**p < 0.01$, $***p < 0.001$, $****p < 0.0001$). Details of statistical tests used, including all p values, are indicated in the relevant figure legend.

DATA AND CODE AVAILABILITY

The data generated in this study are available within the article and its supplemental information. Any additional materials and protocols will be distributed to researchers for non-commercial and academic purposes.

ACKNOWLEDGMENTS

Dr. Lili Yang (UCLA) and Charlie Yan-Ruide Li (UCLA) assisted with NKT expansion and activation. This work was funded through the use of unrestricted funds. J.G. is supported by the UCLA Tumor Cell Biology Training Program (NCI T32CA009056) and the UCLA-Caltech Medical Scientist Training Program (NIGMS T32GM008042). The Flow Cytometry Core of the UCLA Eli and Edythe Broad Center of Regenerative Medicine and Stem Cell Research, the Imaging Core of the UCLA Eli and Edythe Broad Center of Regenerative Medicine and Stem Cell Research, and the Virology Core of the UCLA Center for AIDS Research (CFAR) were used to support studies.

AUTHOR CONTRIBUTIONS

P.G.A. conceived of the project, performed bioinformatic analyses to identify putative enhancer elements, designed the vectors, led the *in vitro* and *in vivo* studies of vector activity, and wrote the manuscript. R.P.H. participated in design and analysis of the SMART LV and edited the manuscript. J.G. performed the ATO experiments and edited the manuscript. L.L., C.J., K.T., J.R., C.T., J.Q., and G.E.M. performed laboratory work in support of the studies. K.B. provided the XLP1 patient cells after acquiring patient consent. D.B., X.W., and R.Z. performed murine transplant work. J.G. and G.M.C. designed and performed studies in the Artificial Thymic Organoid system. F.Y.M. performed bioinformatic analyses. D.B.K. oversaw the studies and edited the manuscript.

DECLARATION OF INTERESTS

The authors declare no competing interests.

SUPPLEMENTAL INFORMATION

Supplemental information can be found online at https://doi.org/10.1016/j.omtm.2024.101323.

REFERENCES

1. Booth, C., Gilmour, K.C., Veys, P., Gennery, A.R., Slatter, M.A., Chapel, H., Heath, P.T., Steward, C.G., Smith, O., O'Meara, A., et al. (2011). X-linked lymphoproliferative disease due to SAP/SH2D1A deficiency: a multicenter study on the manifestations, management and outcome of the disease. Blood *117*, 53–62. https://doi.org/10.1182/blood-2010-06-284935.
2. Ma, C.S., Nichols, K.E., and Tangye, S.G. (2007). Regulation of cellular and humoral immune responses by the SLAM and SAP families of molecules. Annu. Rev. Immunol. *25*, 337–379. https://doi.org/10.1146/annurev.immunol.25.022106.141651.
3. Coffey, A.J., Brooksbank, R.A., Brandau, O., Oohashi, T., Howell, G.R., Bye, J.M., Cahn, A.P., Durham, J., Heath, P., Wray, P., et al. (1998). Host response to EBV infection in X-linked lymphoproliferative disease results from mutations in an SH2-domain encoding gene. Nat. Genet. *20*, 129–135. https://doi.org/10.1038/2424.
4. Latour, S., Gish, G., Helgason, C.D., Humphries, R.K., Pawson, T., and Veillette, A. (2001). Regulation of SLAM-mediated signal transduction by SAP, the X-linked lymphoproliferative gene product. Nat. Immunol. *2*, 681–690. https://doi.org/10.1038/90615.
5. Sayos, J., Wu, C., Morra, M., Wang, N., Zhang, X., Allen, D., Van Schaik, S., Notarangelo, L., Geha, R., Roncarolo, M.G., et al. (1998). The X-linked lymphoproliferative-disease gene product SAP regulates signals induced through the co-receptor SLAM. Nature *395*, 462–469. https://doi.org/10.1038/26683.
6. Dupré, L., Andolfi, G., Tangye, S.G., Clementi, R., Locatelli, F., Aricò, M., Aiuti, A., and Roncarolo, M.-G. (2005). SAP controls the cytolytic activity of CD8+ T cells against EBV-infected cells. Blood *105*, 4383–4389. https://doi.org/10.1182/blood-2004-08-3269.
7. Crotty, S., Kersh, E.N., Cannons, J., Schwartzberg, P.L., and Ahmed, R. (2003). SAP is required for generating long-term humoral immunity. Nature *421*, 282–287. https://doi.org/10.1038/nature01318.
8. Panchal, N., Houghton, B., Diez, B., Ghosh, S., Ricciardelli, I., Thrasher, A.J., Gaspar, H.B., and Booth, C. (2018). Transfer of gene-corrected T cells corrects humoral and cytotoxic defects in patients with X-linked lymphoproliferative disease. J. Allergy Clin. Immunol. *142*, 235–245.e6. https://doi.org/10.1016/j.jaci.2018.02.053.
9. Veillette, A., Zhang, S., Shi, X., Dong, Z., Davidson, D., and Zhong, M.-C. (2008). SAP expression in T cells, not in B cells, is required for humoral immunity. Proc. Natl. Acad. Sci. USA *105*, 1273–1278. https://doi.org/10.1073/pnas.0710698105.
10. Sullivan, J.L., Byron, K.S., Brewster, F.E., Baker, S.M., and Ochs, H.D. (1983). X-linked lymphoproliferative syndrome. Natural history of the immunodeficiency. J. Clin. Invest. *71*, 1765–1778. https://doi.org/10.1172/jci110932.
11. Rivat, C., Booth, C., Alonso-Ferrero, M., Blundell, M., Sebire, N.J., Thrasher, A.J., and Gaspar, H.B. (2013). SAP gene transfer restores cellular and humoral immune function in a murine model of X-linked lymphoproliferative disease. Blood *121*, 1073–1076. https://doi.org/10.1182/blood-2012-07-445858.
12. Tangye, S.G., Cherwinski, H., Lanier, L.L., and Phillips, J.H. (2000). 2B4-mediated activation of human natural killer cells. Mol. Immunol. *37*, 493–501. https://doi.org/10.1016/S0161-5890(00)00076-6.
13. Sharifi, R., Sinclair, J.C., Gilmour, K.C., Arkwright, P.D., Kinnon, C., Thrasher, A.J., and Gaspar, H.B. (2004). SAP mediates specific cytotoxic T-cell functions in X-linked lymphoproliferative disease. Blood *103*, 3821–3827. https://doi.org/10.1182/blood-2003-09-3359.
14. Parolini, S., Bottino, C., Falco, M., Augugliaro, R., Giliani, S., Franceschini, R., Ochs, H.D., Wolf, H., Bonnefoy, J.-Y., Biassoni, R., et al. (2000). X-Linked Lymphoproliferative Disease. J. Exp. Med. *192*, 337–346. https://doi.org/10.1084/jem.192.3.337.
15. Ma, C.S., Hare, N.J., Nichols, K.E., Dupré, L., Andolfi, G., Roncarolo, M.-G., Adelstein, S., Hodgkin, P.D., and Tangye, S.G. (2005). Impaired humoral immunity in X-linked lymphoproliferative disease is associated with defective IL-10 production by CD4+ T cells. J. Clin. Invest. *115*, 1049–1059. https://doi.org/10.1172/jci23139.
16. Qi, H., Cannons, J.L., Klauschen, F., Schwartzberg, P.L., and Germain, R.N. (2008). SAP-controlled T–B cell interactions underlie germinal centre formation. Nature *455*, 764–769. https://doi.org/10.1038/nature07345.
17. Priatel, J.J., Chung, B.K., Tsai, K., and Tan, R. (2014). Natural killer T cell strategies to combat Epstein–Barr virus infection. OncoImmunology *3*, e28329. https://doi.org/10.4161/onci.28329.
18. Sankararaman, S., Riel-Romero, R.M., Jeroudi, M., and Gonzalez-Toledo, E. (2014). Epstein-Barr virus induced hemophagocytic lymphohistiocytosis in X-linked lymphoproliferative disease. J. Neurosci. Rural Pract. *5*, 171–174. https://doi.org/10.4103/0976-3147.131669.
19. Lankester, A.C., Visser, L.F.A., Hartwig, N.G., Bredius, R.G.M., Gaspar, H.B., van der Burg, M., van Tol, M.J.D., Gross, T.G., and Egeler, R.M. (2005). Allogeneic stem cell transplantation in X-linked lymphoproliferative disease: two cases in one family and review of the literature. Bone Marrow Transplant. *36*, 99–105. https://doi.org/10.1038/sj.bmt.1705016.
20. Poletti, V., and Mavilio, F. (2021). Designing Lentiviral Vectors for Gene Therapy of Genetic Diseases. Viruses *13*, 1526. https://doi.org/10.3390/v13081526.
21. Catarino, R.R., and Stark, A. (2018). Assessing sufficiency and necessity of enhancer activities for gene expression and the mechanisms of transcription activation. Genes Dev. *32*, 202–223. https://doi.org/10.1101/gad.310367.117.
22. Nagy, N., Matskova, L., Kis, L.L., Hellman, U., Klein, G., and Klein, E. (2009). The proapoptotic function of SAP provides a clue to the clinical picture of X-linked lymphoproliferative disease. Proc. Natl. Acad. Sci. USA *106*, 11966–11971. https://doi.org/10.1073/pnas.0905691106.
23. Gartshteyn, Y., Askanase, A.D., and Mor, A. (2021). SLAM Associated Protein Signaling in T Cells: Tilting the Balance Toward Autoimmunity. Front. Immunol. *12*, 654839. https://doi.org/10.3389/fimmu.2021.654839.
24. Passerini, L., Santoni de Sio, F.R., Roncarolo, M.G., and Bacchetta, R. (2014). Forkhead box P3: the peacekeeper of the immune system. Int. Rev. Immunol. *33*, 129–145. https://doi.org/10.3109/08830185.2013.863303.
25. Santilli, G., Almarza, E., Brendel, C., Choi, U., Beilin, C., Blundell, M.P., Haria, S., Parsley, K.L., Kinnon, C., Malech, H.L., et al. (2011). Biochemical correction of X-CGD by a novel chimeric promoter regulating high levels of transgene expression in myeloid cells. Mol. Ther. *19*, 122–132. https://doi.org/10.1038/mt.2010.226.
26. Morgan, R.A., Unti, M.J., Aleshe, B., Brown, D., Osborne, K.S., Koziol, C., Ayoub, P.G., Smith, O.B., O'Brien, R., Tam, C., et al. (2020). Improved Titer and Gene Transfer by Lentiviral Vectors Using Novel, Small β-Globin Locus Control Region Elements. Mol. Ther. *28*, 328–340. https://doi.org/10.1016/j.ymthe.2019.09.020.
27. Wong, R.L., Sackey, S., Brown, D., Senadheera, S., Masiuk, K., Quintos, J.P., Colindres, N., Riggan, L., Morgan, R.A., Malech, H.L., et al. (2023). Lentiviral gene therapy for X-linked chronic granulomatous disease recapitulates endogenous CYBB regulation and expression. Blood *141*, 1007–1022. https://doi.org/10.1182/blood.2022016074.
28. Fishilevich, S., Nudel, R., Rappaport, N., Hadar, R., Plaschkes, I., Iny Stein, T., Rosen, N., Kohn, A., Twik, M., Safran, M., et al. (2017). GeneHancer: genome-wide integration of enhancers and target genes in GeneCards. Database *2017*, bax028. https://doi.org/10.1093/database/bax028.
29. Bystrykh, L.V. (2012). Generalized DNA barcode design based on Hamming codes. PLoS One *7*, e36852. https://doi.org/10.1371/journal.pone.0036852.
30. Cooper, A.R., Patel, S., Senadheera, S., Plath, K., Kohn, D.B., and Hollis, R.P. (2011). Highly efficient large-scale lentiviral vector concentration by tandem tangential flow filtration. J. Virol. Methods *177*, 1–9. https://doi.org/10.1016/j.jviromet.2011.06.019.
31. Stec, M., Weglarczyk, K., Baran, J., Zuba, E., Mytar, B., Pryjma, J., and Zembala, M. (2007). Expansion and differentiation of CD14+CD16(-) and CD14+ +CD16+ human monocyte subsets from cord blood CD34+ hematopoietic progenitors. J. Leukoc. Biol. *82*, 594–602. https://doi.org/10.1189/jlb.0207117.
32. Aryee, K.E., Burzenski, L.M., Yao, L.C., Keck, J.G., Greiner, D.L., Shultz, L.D., and Brehm, M.A. (2022). Enhanced development of functional human NK cells in NOD-scid-IL2rg(null) mice expressing human IL15. Faseb. J. *36*, e22476. https://doi.org/10.1096/fj.202200045R.
33. Brehm, M.A., Cuthbert, A., Yang, C., Miller, D.M., DiIorio, P., Laning, J., Burzenski, L., Gott, B., Foreman, O., Kavirayani, A., et al. (2010). Parameters for establishing humanized mouse models to study human immunity: analysis of human hematopoietic

stem cell engraftment in three immunodeficient strains of mice bearing the IL2rgamma(null) mutation. Clin. Immunol. *135*, 84–98. https://doi.org/10.1016/j.clim.2009.12.008.

34. Montel-Hagen, A., Seet, C.S., Li, S., Chick, B., Zhu, Y., Chang, P., Tsai, S., Sun, V., Lopez, S., Chen, H.C., et al. (2019). Organoid-Induced Differentiation of Conventional T Cells from Human Pluripotent Stem Cells. Cell Stem Cell *24*, 376–389.e8. https://doi.org/10.1016/j.stem.2018.12.011.

35. Seet, C.S., He, C., Bethune, M.T., Li, S., Chick, B., Gschweng, E.H., Zhu, Y., Kim, K., Kohn, D.B., Baltimore, D., et al. (2017). Generation of mature T cells from human hematopoietic stem and progenitor cells in artificial thymic organoids. Nat. Methods *14*, 521–530. https://doi.org/10.1038/nmeth.4237.

36. Grote, A., Hiller, K., Scheer, M., Münch, R., Nörtemann, B., Hempel, D.C., and Jahn, D. (2005). JCat: a novel tool to adapt codon usage of a target gene to its potential expression host. Nucleic Acids Res. *33*, W526–W531. https://doi.org/10.1093/nar/gki376.

37. Raab, D., Graf, M., Notka, F., Schödl, T., and Wagner, R. (2010). The GeneOptimizer Algorithm: using a sliding window approach to cope with the vast sequence space in multiparameter DNA sequence optimization. Syst. Synth. Biol. *4*, 215–225. https://doi.org/10.1007/s11693-010-9062-3.

38. Snow, A.L., Marsh, R.A., Krummey, S.M., Roehrs, P., Young, L.R., Zhang, K., van Hoff, J., Dhar, D., Nichols, K.E., Filipovich, A.H., et al. (2009). Restimulation-induced apoptosis of T cells is impaired in patients with X-linked lymphoproliferative disease caused by SAP deficiency. J. Clin. Invest. *119*, 2976–2989. https://doi.org/10.1172/jci39518.

39. Houghton, B.C., Panchal, N., Haas, S.A., Chmielewski, K.O., Hildenbeutel, M., Whittaker, T., Mussolino, C., Cathomen, T., Thrasher, A.J., and Booth, C. (2022). Genome Editing With TALEN, CRISPR-Cas9 and CRISPR-Cas12a in Combination With AAV6 Homology Donor Restores T Cell Function for XLP. Front Genome Ed. *4*, 828489. https://doi.org/10.3389/fgeed.2022.828489.

40. Pohida, K., Lake, C.M., Yee, D., and Snow, A.L. (2022). Restimulation-Induced Cell Death (RICD): Methods for Modeling, Investigating, and Quantifying RICD Sensitivity in Primary Human T Cells via Flow Cytometric Analysis. Bio. Protoc. *12*, e4326. https://doi.org/10.21769/BioProtoc.4326.

41. Kandarian, F., Sunga, G.M., Arango-Saenz, D., and Rossetti, M. (2017). A Flow Cytometry-Based Cytotoxicity Assay for the Assessment of Human NK Cell Activity. J. Vis. Exp. 56191. https://doi.org/10.3791/56191.

42. Aoukaty, A., and Tan, R. (2002). Association of the X-linked lymphoproliferative disease gene product SAP/SH2D1A with 2B4, a natural killer cell-activating molecule, is dependent on phosphoinositide 3-kinase. J. Biol. Chem. *277*, 13331–13337. https://doi.org/10.1074/jbc.M112029200.

43. Shen, W., Liu, S., and Ou, L. (2022). rAAV immunogenicity, toxicity, and durability in 255 clinical trials: A meta-analysis. Front. Immunol. *13*, 1001263. https://doi.org/10.3389/fimmu.2022.1001263.

44. Ertl, H.C.J. (2022). Immunogenicity and toxicity of AAV gene therapy. Front. Immunol. *13*, 975803. https://doi.org/10.3389/fimmu.2022.975803.

45. Brunet, E., and Jasin, M. (2018). Induction of Chromosomal Translocations with CRISPR-Cas9 and Other Nucleases: Understanding the Repair Mechanisms That Give Rise to Translocations. Adv. Exp. Med. Biol. *1044*, 15–25. https://doi.org/10.1007/978-981-13-0593-1_2.

46. Leibowitz, M.L., Papathanasiou, S., Doerfler, P.A., Blaine, L.J., Sun, L., Yao, Y., Zhang, C.-Z., Weiss, M.J., and Pellman, D. (2021). Chromothripsis as an on-target consequence of CRISPR–Cas9 genome editing. Nat. Genet. *53*, 895–905. https://doi.org/10.1038/s41588-021-00838-7.

47. Kantor, A., McClements, M.E., and MacLaren, R.E. (2020). CRISPR-Cas9 DNA Base-Editing and Prime-Editing. Int. J. Mol. Sci. *21*, 6240. https://doi.org/10.3390/ijms21176240.

48. Xiang, Q.M., Jiang, N., Liu, Y.F., Wang, Y.B., Mu, D.A., Liu, R., Sun, L.Y., Zhang, W., Guo, Q., and Li, K. (2023). Overexpression of SH2D1A promotes cancer progression and is associated with immune cell infiltration in hepatocellular carcinoma via bioinformatics and in vitro study. BMC Cancer *23*, 1005. https://doi.org/10.1186/s12885-023-11315-1.

49. Rothenberg, E.V., Hosokawa, H., and Ungerbäck, J. (2019). Mechanisms of Action of Hematopoietic Transcription Factor PU.1 in Initiation of T-Cell Development. Front. Immunol. *10*, 228. https://doi.org/10.3389/fimmu.2019.00228.

50. Wang, M., Wang, H., Wen, Y., Chen, X., Liu, X., Gao, J., Su, P., Xu, Y., Zhou, W., Shi, L., and Zhou, J. (2018). MEIS2 regulates endothelial to hematopoietic transition of human embryonic stem cells by targeting TAL1. Stem Cell Res. Ther. *9*, 340. https://doi.org/10.1186/s13287-018-1074-z.

51. Duddu, S., Chakrabarti, R., Ghosh, A., and Shukla, P.C. (2020). Hematopoietic Stem Cell Transcription Factors in Cardiovascular Pathology. Front. Genet. *11*, 588602. https://doi.org/10.3389/fgene.2020.588602.

52. Peng, Z., Zhang, Y., Ma, X., Zhou, M., Wu, S., Song, Z., Yuan, Y., Chen, Y., Li, Y., Wang, G., et al. (2021). Brd4 Regulates the Homeostasis of CD8(+) T-Lymphocytes and Their Proliferation in Response to Antigen Stimulation. Front. Immunol. *12*, 728082. https://doi.org/10.3389/fimmu.2021.728082.

53. Palendira, U., Low, C., Chan, A., Hislop, A.D., Ho, E., Phan, T.G., Deenick, E., Cook, M.C., Riminton, D.S., Choo, S., et al. (2011). Molecular pathogenesis of EBV susceptibility in XLP as revealed by analysis of female carriers with heterozygous expression of SAP. PLoS Biol. *9*, e1001187. https://doi.org/10.1371/journal.pbio.1001187.

54. Han, J., Tam, K., Tam, C., Hollis, R.P., and Kohn, D.B. (2021). Improved lentiviral vector titers from a multi-gene knockout packaging line. Mol. Ther. Oncolytics *23*, 582–592. https://doi.org/10.1016/j.omto.2021.11.012.

55. Hindson, B.J., Ness, K.D., Masquelier, D.A., Belgrader, P., Heredia, N.J., Makarewicz, A.J., Bright, I.J., Lucero, M.Y., Hiddessen, A.L., Legler, T.C., et al. (2011). High-throughput droplet digital PCR system for absolute quantitation of DNA copy number. Anal. Chem. *83*, 8604–8610. https://doi.org/10.1021/ac202028g.

56. Zufferey, R., Dull, T., Mandel, R.J., Bukovsky, A., Quiroz, D., Naldini, L., and Trono, D. (1998). Self-inactivating lentivirus vector for safe and efficient in vivo gene delivery. J. Virol. *72*, 9873–9880. https://doi.org/10.1128/jvi.72.12.9873-9880.1998.

57. Katz, G., Voss, K., Yan, T.F., Kim, Y.C., Kortum, R.L., Scott, D.W., and Snow, A.L. (2018). FOXP3 renders activated human regulatory T cells resistant to restimulation-induced cell death by suppressing SAP expression. Cell. Immunol. *327*, 54–61. https://doi.org/10.1016/j.cellimm.2018.02.007.

OMTM, Volume *32*

Supplemental information

Lentiviral vectors for precise expression to treat X-linked lymphoproliferative disease

Paul G. Ayoub, Julia Gensheimer, Lindsay Lathrop, Colin Juett, Jason Quintos, Kevin Tam, Jack Reid, Feiyang Ma, Curtis Tam, Grace E. McAuley, Devin Brown, Xiaomeng Wu, Ruixue Zhang, Kathryn Bradford, Roger P. Hollis, Gay M. Crooks, and Donald B. Kohn

Figure S1:

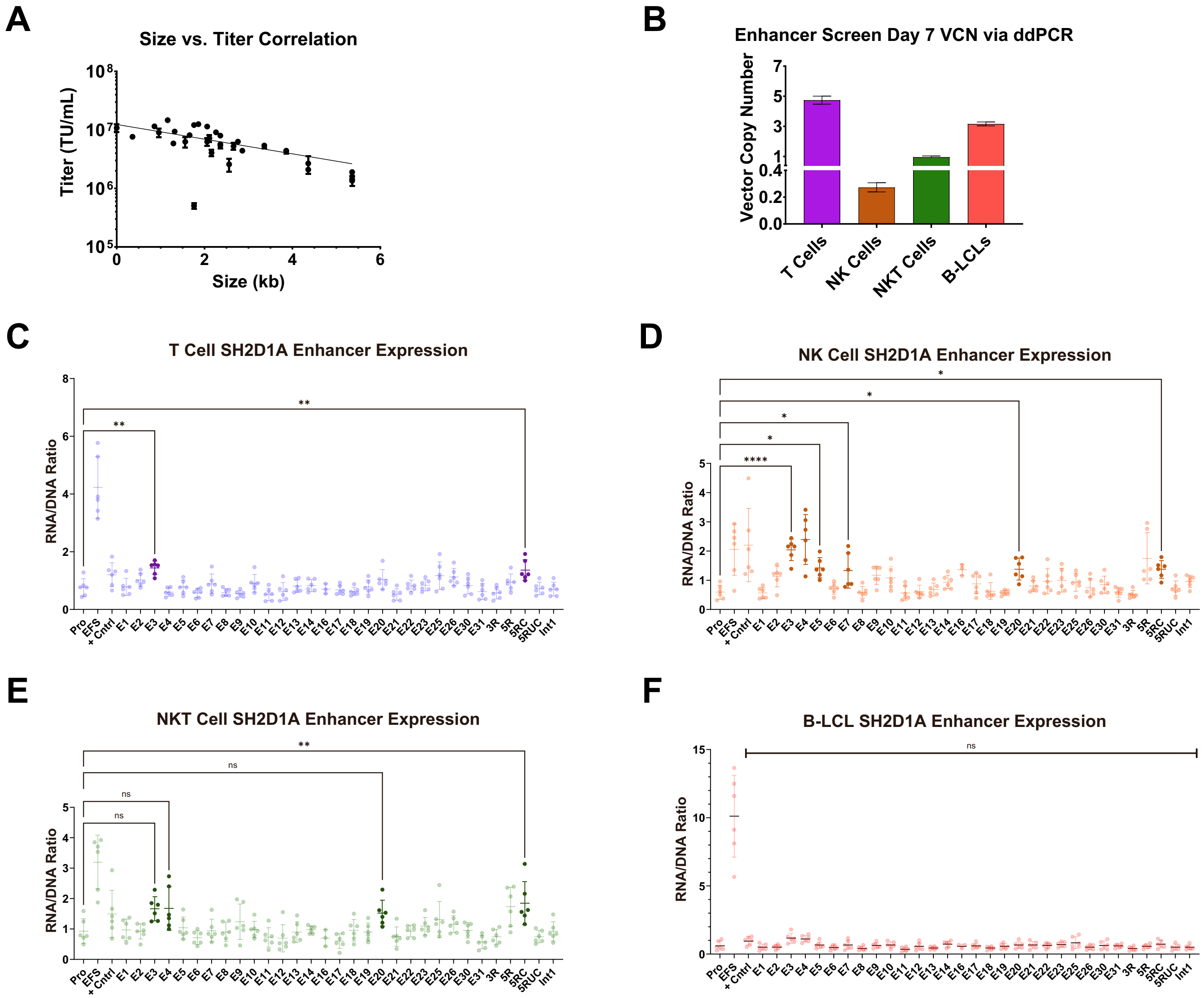

A: Effect of Lentivirus Proviral Size on Titer. Putative enhancers were cloned into the plasmid backbone of a therapeutic lentiviral vector (pCCL-c-MNDU3-X [Addgene Plasmid #81071]), packaged, titered head-to-head, and the quantity of infectious particles was plotted as a function of proviral length (bp). Each point in the plot represents an average of three individual 10-cm plates of virus titered on HT-29 cells. Proviral length is defined as sequence length from the beginning of the 5′ long terminal repeat (LTR) U3 through the end of the 3′ LTR U5. n = 3 per arm. Linear regression analyses were used to determine the correlation between titer and proviral size (R^2=0.51).

B: Enhancer Screen Bulk Vector Copy Number. We transduced primary T, NKT, and NK cells with a pool of raw viral supernatant containing $5x10^4$ TU/mL of the 34 candidate XLP1-SMART-LVs and the EFS-mCitrine vector. B-LCLs were transduced to measure off-target expression. 14 days post-transduction, cells were harvested for their gDNA fraction to measure vector copy number using digital droplet PCR. Data are represented as mean ± SD of biological triplicates from one experiment.

C-F: Relative SH2D1A Enhancer Activity in T, NK, NKT, and B-LCL Cells. We transduced primary T, NKT, and NK cells with a pool of raw viral supernatant containing each of the 34 candidate XLP1-SMART-LVs, in duplicate, and the EFS-mCit vector. Each candidate LV was cloned with two unique barcodes. B-LCLs were transduced to measure off-target expression. 14 days post-transduction, cells were harvested for their gDNA and RNA fractions to measure barcode expression via next-generation sequencing. The RNA barcode counts identify active enhancers within each lineage whereas each genomic barcode count is used normalize each barcode in the transcript to the genome. The number of RNA barcode reads, normalized to frequency of gDNA barcodes within each cell type, determined the relative expression of each element. Data are represented as mean ± SD of biological triplicates from two experiments. We analyzed statistical significance using a one-way ANOVA followed by multiple paired comparisons for normally distributed data (Tukey test). All statistical tests were two-tailed and a p value of < 0.05 was deemed significant (ns non-significant, *P < 0.05, **P < 0.01, ***P < 0.001, ****P < 0.0001).

Figure S2:

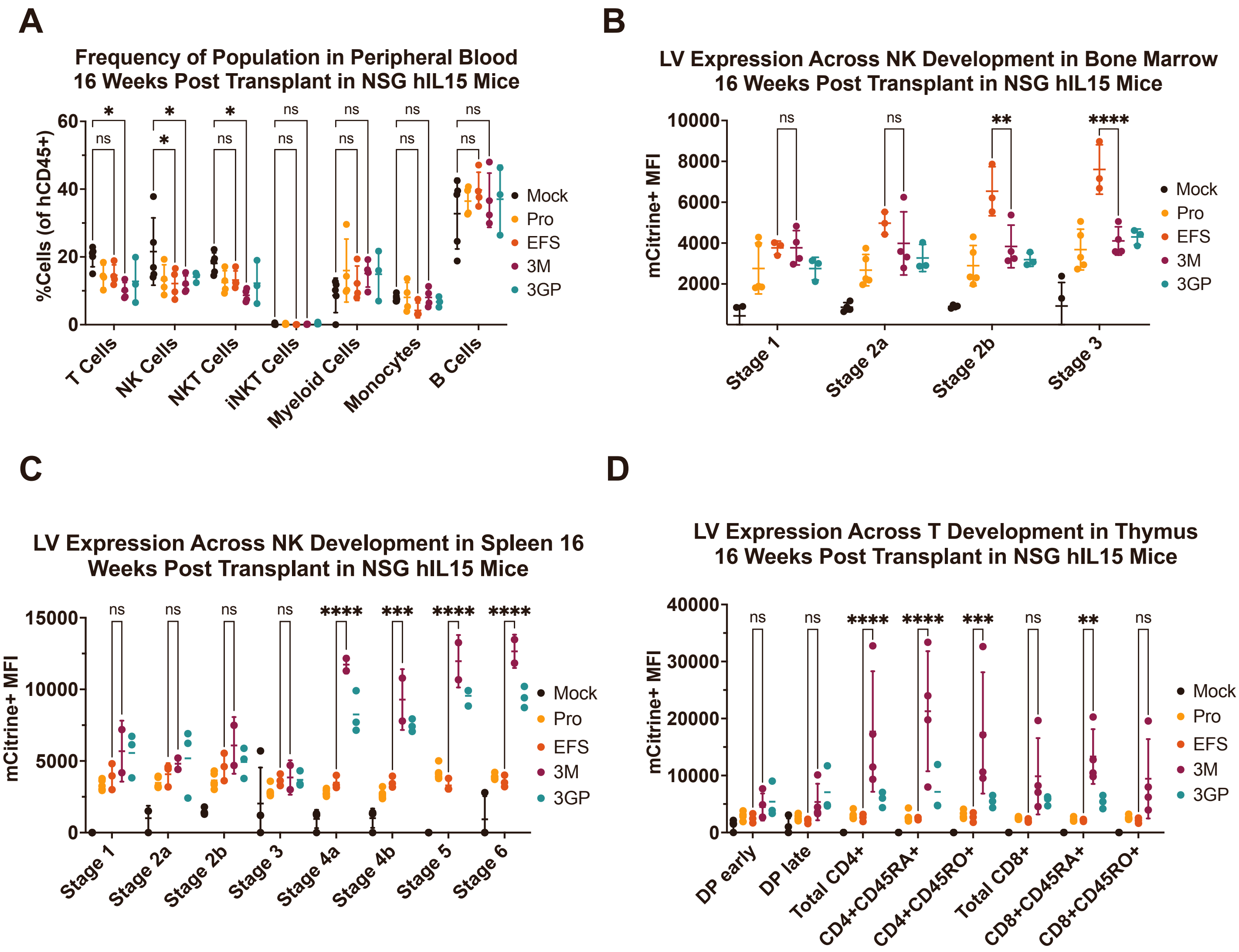

A: Frequency of Hematopoietic Populations in Peripheral Blood 16 Weeks Post Transplant in hIL15 NSG Mice. Mice were bled at 16 weeks to analyze peripheral blood for XLP1-SMART-LV expression. Lysed red blood cells were stained for various lineages within the hCD45+ gate (T Cells: hCD33- hCD19- hCD3+; NK Cells: hCD33- hCD3- hCD19- hCD56+; NKT Cells: hCD33- hCD19- hCD3+ hCD56+; iNKT cells: hCD33- hCD19- hCD3+ hCD56+ hVα24+; Myeloid Cells: hCD33+; Monocytes: hCD33+, CD14+, CD16-; B Cells: hCD33- hCD19+ hCD3-). Frequency of each population is plotted as a percentage of total hCD45+ cells using flow cytometry. Data are represented as mean ± SD of biological triplicates from one experiment. We analyzed statistical significance using a two-way ANOVA followed by multiple paired comparisons for normally distributed data (Tukey test). All statistical tests were two-tailed and a p value of < 0.05 was deemed significant (ns non-significant, *P < 0.05, **P < 0.01, ***P < 0.001, ****P < 0.0001).

B: XLP1-SMART-LV Expression across NK cell Development in Bone Marrow 16 Weeks Post Transplant in hIL15 NSG Mice. Whole bone marrow (BM) was taken from each mouse at time of euthanasia and processed into a single cell suspension. Single cells were stained for various stages of NK cell differentiation within the hCD45+hCD33- gate (Stage 1: hCD34+; Stage 2a: hCD34+ hCD117+ hCD122-; Stage 2b: hCD34+ hCD117+ hCD122+; Stage 3: hCD34- hCD117+ hCD122+ hCD56-). Each LV's relative expression was measured via mCitrine+ MFI using flow cytometry. Each enhancer was compared to basal SH2D1A promoter expression (Pro) and the preclinical vector (EFS). Data are represented as mean ± SD of biological triplicates from one experiment. We analyzed statistical significance using a two-way ANOVA followed by multiple paired comparisons for normally distributed data (Tukey test). All statistical tests were two-tailed and a p value of < 0.05 was deemed significant (ns non-significant, *P < 0.05, **P < 0.01, ***P < 0.001, ****P < 0.0001).

C: XLP1-SMART-LV Expression across NK cell Development in Spleen 16 Weeks Post Transplant in hIL15 NSG Mice. The spleen was taken from each mouse at time of euthanasia, processed into a single cell suspension, and lysed. Lysed splenic single cells were stained for various stages of NK cell differentiation within the hCD45+hCD33- gate (Stage 1: hCD34+; Stage 2a: hCD34+ hCD117+ hCD122-; Stage 2b: hCD34+ hCD117+ hCD122+; Stage 3: hCD34- hCD117+ hCD122+ hCD56-; Stage 4a: hCD34- hCD117+ hCD122+ hCD56+ hCD94+; Stage 4b: hCD34- hCD117- hCD122+ hCD56+ hCD94+ hNKp80+; Stage 5: hCD34- hCD117- hCD122+ hCD56+ hCD94+ hNKp80+ hCD16+; and Stage 6: hCD34- hCD117- CD122+ hCD56+ hCD94+ hNKp80+ hCD16+ hCD57). Each LV's relative expression was measured via mCitrine+ MFI using flow cytometry. Each enhancer was compared to basal SH2D1A promoter expression (Pro) and the preclinical vector (EFS). Data are represented as mean ± SD of biological triplicates from one experiment. We analyzed statistical significance using a two-way ANOVA followed by multiple paired comparisons for normally distributed data (Tukey test). All statistical tests were two-tailed and a p value of < 0.05 was deemed significant (ns non-significant, *P < 0.05, **P < 0.01, ***P < 0.001, ****P < 0.0001).

D: XLP1-SMART-LV Expression across T cell Development in Thymus 16 Weeks Post Transplant in hIL15 NSG Mice. The thymus was taken from each mouse at time of euthanasia and processed into a single cell suspension. The single cell suspension was stained for mature stages of T cell differentiation within the hCD45+ hCD34- hCD14- hCD19- hCD56- hCD5+ hCD7+ TCRab+ CD3+ gate (DP early: TCRab-CD3-CD8+CD4+; DP late: hCD4+ hCD8+; total CD4+: hCD4+ hCD8-; CD4+CD45RA+: hCD4+ hCD8- hCD45RA+ hCD45RO-; CD4+CD45RO+: hCD4+ hCD8- hCD45RA- hCD45RO+; total CD8+: hCD4- hCD8+; CD8+CD45RA+: hCD4- hCD8+ hCD45RA+ hCD45RO-; CD8+CD45RO+: hCD4- hCD8+ hCD45RA- hCD45RO+). Each LV's relative expression was measured via mCitrine+ MFI using flow cytometry. Each enhancer was compared to basal SH2D1A promoter expression (Pro) and the preclinical vector (EFS), both

harboring an mCitrine reporter cassette. Data are represented as mean ± SD of biological triplicates from one experiment. We analyzed statistical significance using a two-way ANOVA followed by multiple paired comparisons for normally distributed data (Tukey test). All statistical tests were two-tailed and a p value of < 0.05 was deemed significant (ns non-significant, *$P < 0.05$, **$P < 0.01$, ***$P < 0.001$, ****$P < 0.0001$.).

Figure S3:

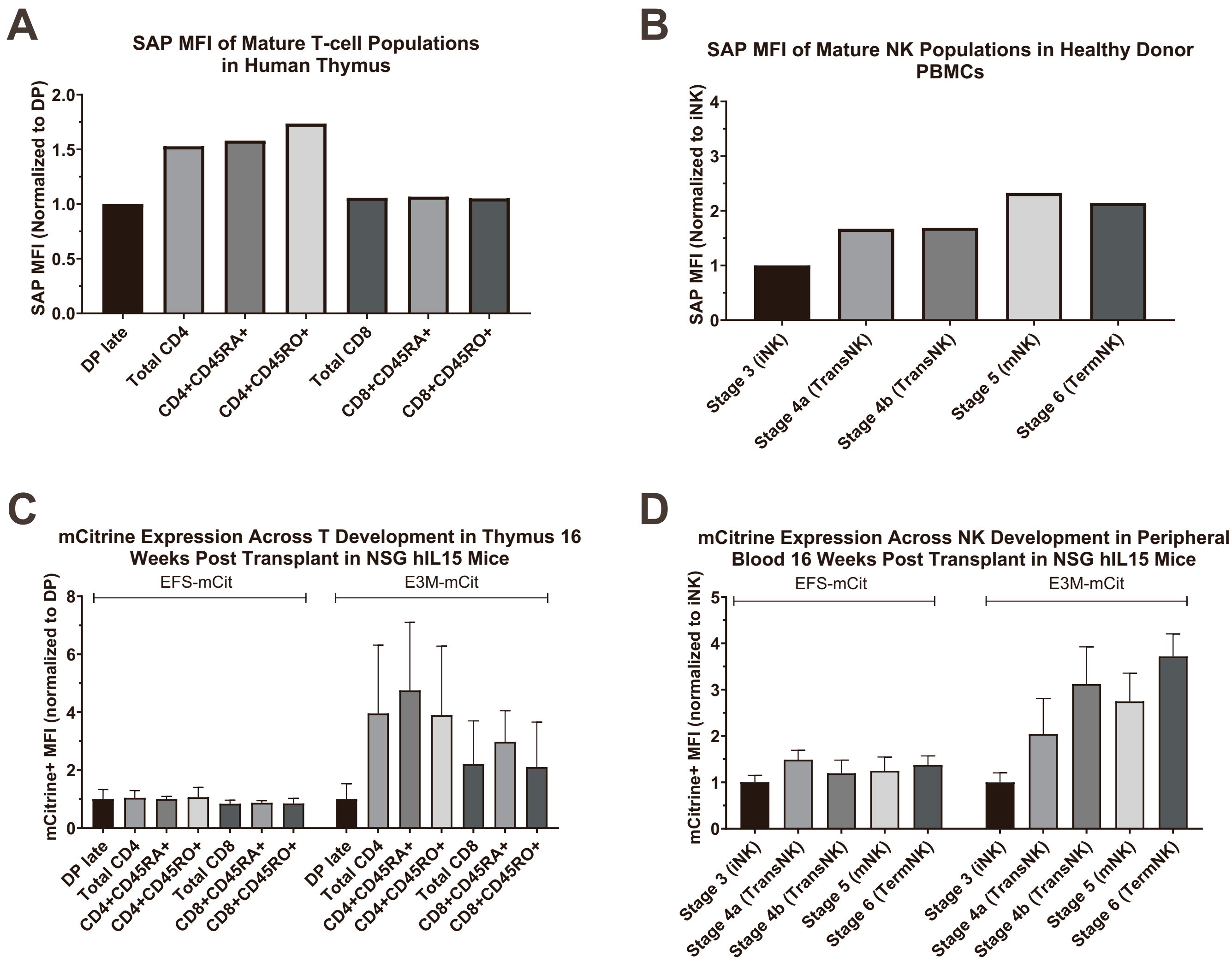

A: SAP Mean Fluorescence Intensity of T cell Populations in Human Thymus: Healthy donor human thymic tissue was processed into a single cell suspension. The single cell suspension was stained for mature stages of T cell differentiation within the hCD45+ hCD34- hCD14- hCD19- hCD56- hCD5+ hCD7+ TCRab+ CD3+ gate (DP: hCD4+ hCD8+; total CD4+: hCD4+ hCD8-; CD4+CD45RA+: hCD4+ hCD8- hCD45RA+ hCD45RO-; CD4+CD45RO+: hCD4+ hCD8- hCD45RA- hCD45RO+; total CD8+: hCD4- hCD8+; CD8+CD45RA+: hCD4- hCD8+ hCD45RA+ hCD45RO-; CD8+CD45RO+: hCD4- hCD8+ hCD45RA- hCD45RO+). Stained cells were then fixed, permeabilized and stained for SAP using an anti-SAP monoclonal antibody. SAP expression was determined via total SAP MFI within each target population using flow cytometry.

B: SAP Mean Fluorescence Intensity of Mature NK cell Populations in Three Healthy Donor PBMC Samples: CD56+ NK cells from (n=3) healthy donors (HD) were isolated from PBMCs. Cells were stained with monoclonal antibodies for various stages of NK development (Stage 3: hCD34- hCD117+ hCD122+ hCD56-; Stage 4a: hCD34- hCD117+ hCD122+ hCD56+ hCD94+; Stage 4b: hCD34- hCD117- hCD122+ hCD56+ hCD94+ hNKp80+; Stage 5: hCD34- hCD117- hCD122+ hCD56+ hCD94+ hNKp80+ hCD16+; and Stage 6: hCD34- hCD117- CD122+ hCD56+ hCD94+ hNKp80+ hCD16+ hCD57). Stained cells were then fixed, permeabilized and stained for SAP using an anti-SAP monoclonal antibody. SAP expression was determined via total SAP MFI within each target population using flow cytometry.

C-D: XLP1-SMART-LV Expression across T cell and NK cell Development 16 Weeks Post Transplant in hIL15 NSG mice. The following graphs are representative data shown in Figure 3, panels E and F. They depict XLP1-SMART-LV expression across T cell development (C) and NK cell development (D) 16 weeks post transplant in hIL15 NSG mice. mCitrine+ MFI was detected in T and NK cell subpopulations to assess relative XLP1-SMART-LV patterns of expression in comparison to patterns of SAP expression shown in panels A and B.

Figure S4:

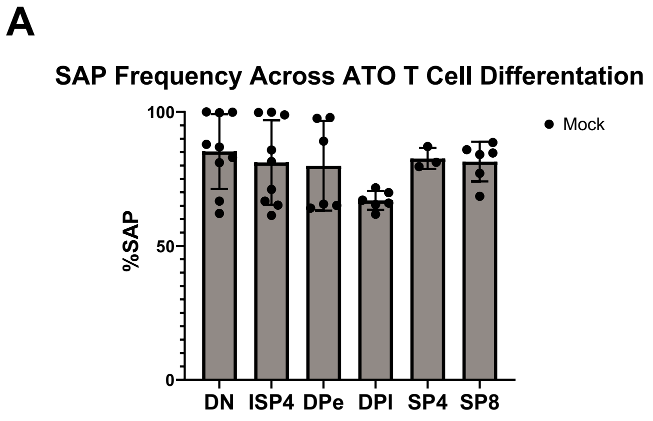

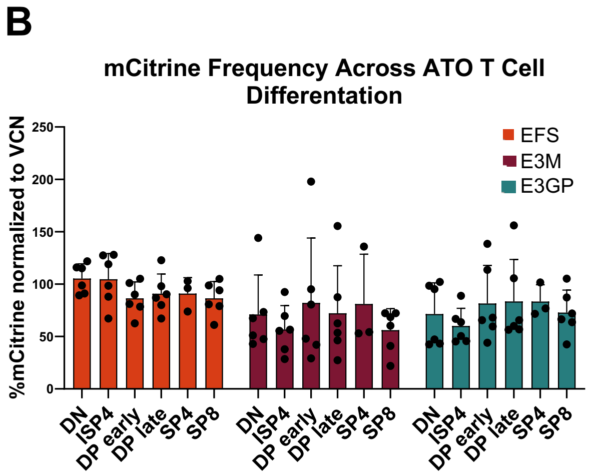

A-B: Representative SAP and mCitrine Expression within Healthy Donor PBSC CD34+ Derived Artificial Thymic Organoid Cultures. To determine SAP expression and SMART-LV expression across T cell maturation, healthy donor mobilized peripheral blood CD34+ cells were transduced with XLP1-SMART-LVs harboring an mCitrine reporter cassette and differentiated into T cells using the 3D artificial thymic organoid (ATO) system. At weeks 3, 7, and 12 of differentiation, ATOs were harvested and stained to measure their relative **(A)** SAP expression (after fixation and permeabilization) or **(B)** mCitrine expression using flow cytometry. ATOs were stained to differentiate the following stages of T cell development: double negative (DN): hTCRab-hCD3-hCD4-hCD8-; immature single positive 4 (ISP4): hTCRab-hCD3-hCD4+hCD8-; double positive (DP) early: hTCRab-hCD3-hCD4+hCD8+; DP late: hTCRab+hCD3+hCD4+hCD8+; single positive (SP) 4: hTCRab+hCD3+hCD4+hCD8-; and SP8: hTCRab+hCD3+hCD4-hCD8+. A representative plot of the frequency of SAP+ cells **(A)** and the frequency of mCitrine+ cells **(B)** across weeks 3, 7, and 12 is shown, in which data were normalized to the VCN of the bulk ATO populations. VCNs were as follows: EFS = 0.39, E3M = 0.184, E3GP = 0.25. Data are represented as mean ± SD of biological triplicates from one experiment across three timepoints (weeks 3, 7, and 12).

Figure S5:

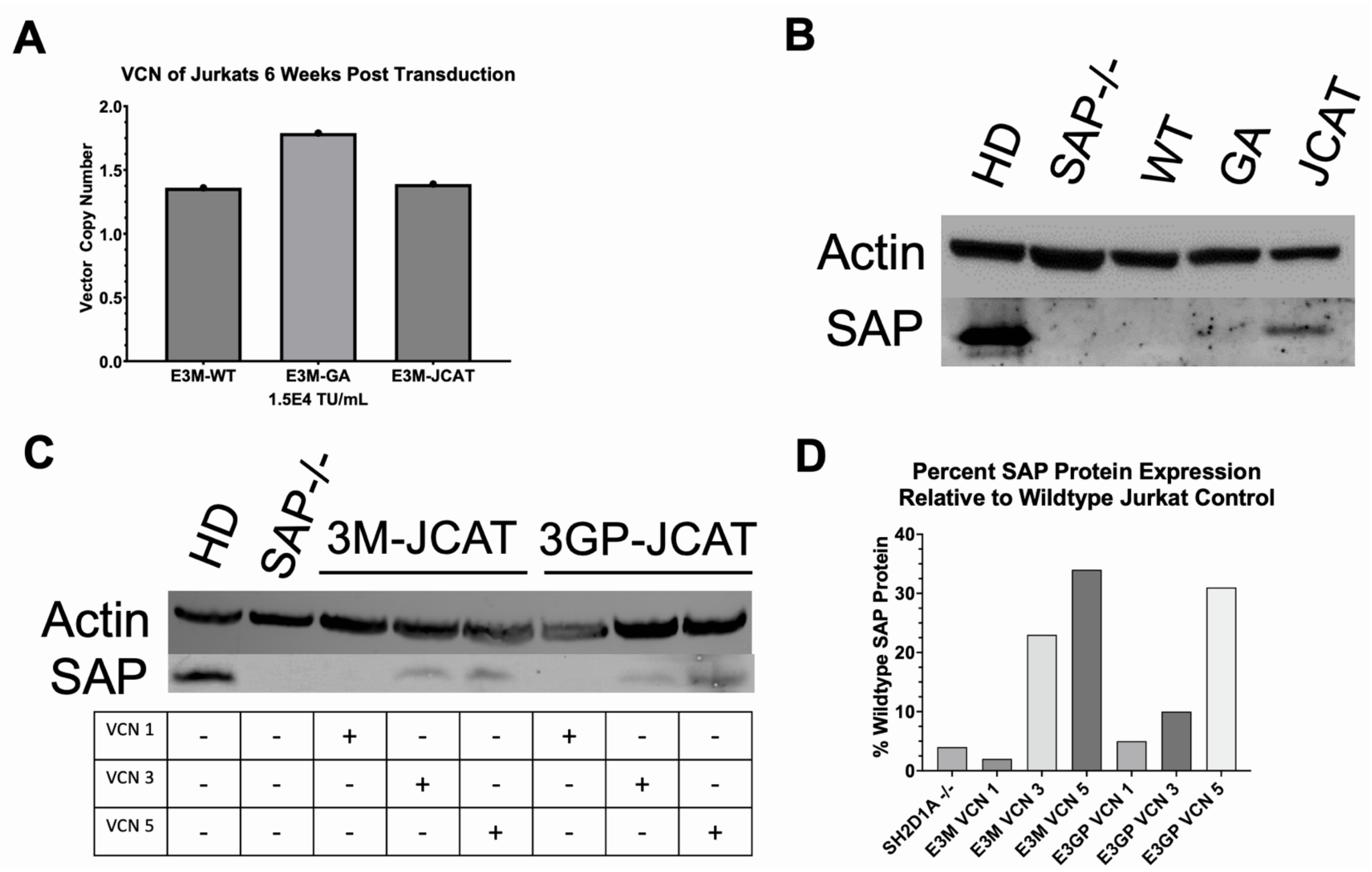

A: VCN of Jurkat cells 6 weeks post transduction with XLP-SMART-LV. SH2D1A -/- Jurkat cells were transduced at an equivalent vector copy number (VCN) with the E3M-E20R-5RL lentiviral vector harboring an SH2D1A cDNA cassette containing wildtype codons, GeneArt (GA), or Java Codon Adaptation Tool (JCAT) codon optimizations. 14 days after transduction, cells were harvested for VCN measurement by ddPCR.

B: Western blot of cells transduced with XLP-SMART-LV. Protein was extracted from the transduced populations shown in panel A. The reconstitution of SAP protein after transduction was measured by western blot using an anti-SAP antibody (clone 1C9; Abnova) with a 1:1000 dilution of primary antibody.

C: Western blot for dose response transduction of Jurkat cells transduced with JCat codon optimized XLP1-SMART-LVs. SH2D1A -/- Jurkat cells were transduced with the JCat codon optimized E3M-E20R-5RL or E3GP-E20R-5RL lentiviral vectors to achieve VCNs of 1,3, and 5. 14 days after transduction, cells were harvested for VCN

measurement by ddPCR. Protein was extracted from the transduced populations and measured by western blot using an anti-SAP antibody (clone 1C9; Abnova).

D: Densitometry quantification of Western Blot dose response. The western blot images produced in panel C were quantified for total Actin and SAP protein using densitometry via the ImageJ image processing and analysis tool. After quantification, SAP protein was normalized to the actin loading control. Relative SAP protein expression was determined by measuring the ratio of normalized SAP protein in the experimental condition to the normalized SAP protein of the wildtype Jurkat control.

Figure S6:

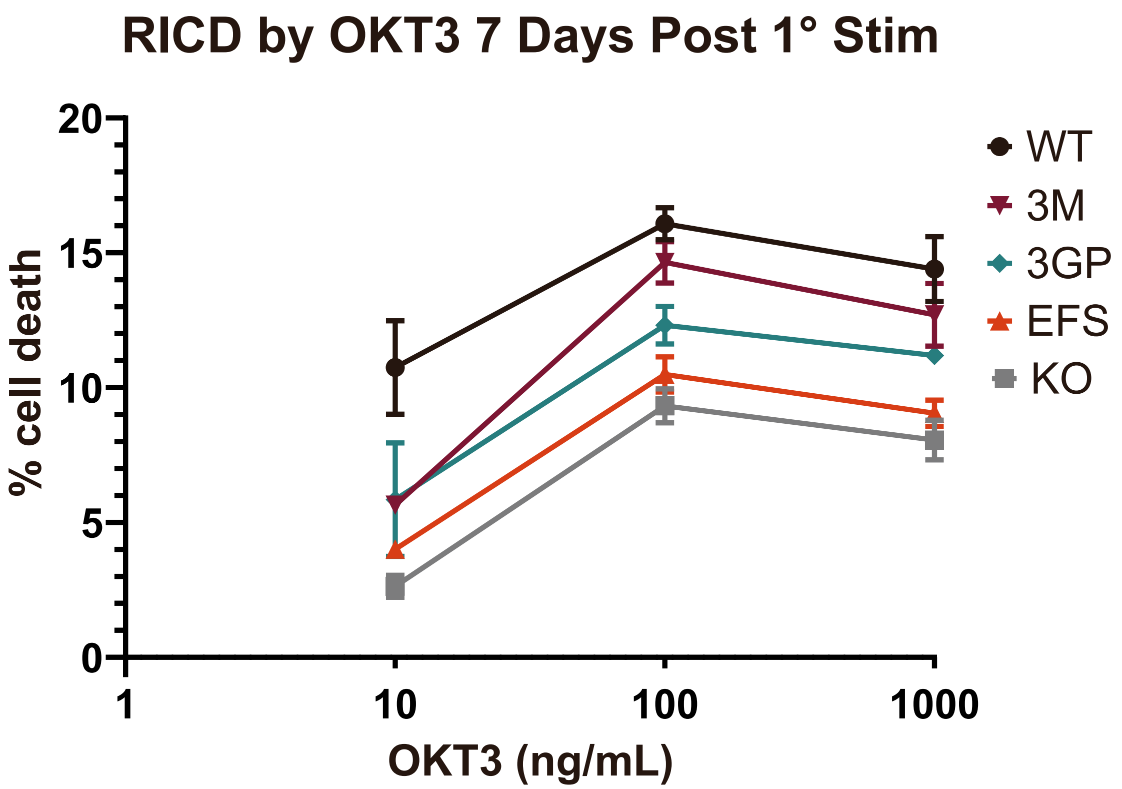

A: T Cell Restimulation Induced Cell Death (RICD) Assay of SH2D1A-/- CD8+ T Cells Transduced with XLP1-SMART-LVs. CD8+ T cells from a healthy donor (HD) and were isolated from PBMCs and transfected with sgRNAs targeting Exon 2 of the SH2D1A gene to knockout SAP expression. SH2D1A -/- T cells were transduced with XLP1-SMART-LVs (VCNs are EFS = 2.79, E3M = 2.5, E3GP = 2.46). 10 days after transduction, cells were plated for RICD in OKT3 at final concentrations of 1000 ng/ml, 100 ng/ml and 10 ng/ml. After 24 hours, the recovery of RICD in XLP1-SMART-LV transduced cells was compared to a HD control. The number of live cells (PI-) in stimulated controls were compared to unstimulated controls to measure the % cell loss = [1-(# PI- restimulated cells/# PI- untreated cells)]x100. Data are represented as mean ± SD of biological triplicates from one experiment. We analyzed statistical significance using a two-way ANOVA followed by multiple paired comparisons for normally distributed data (Tukey test). All statistical tests were two-tailed and a p value of < 0.05 was deemed significant (ns non-significant, *P < 0.05, **P < 0.01, ***P < 0.001, ****P < 0.0001). Compared to XLP knockout samples, EFS was deemed significant with a p value <0.05; E3M was deemed significant with a p value <0.0001; and E3GP was deemed significant with a p value <0.0001.

Figure S7:

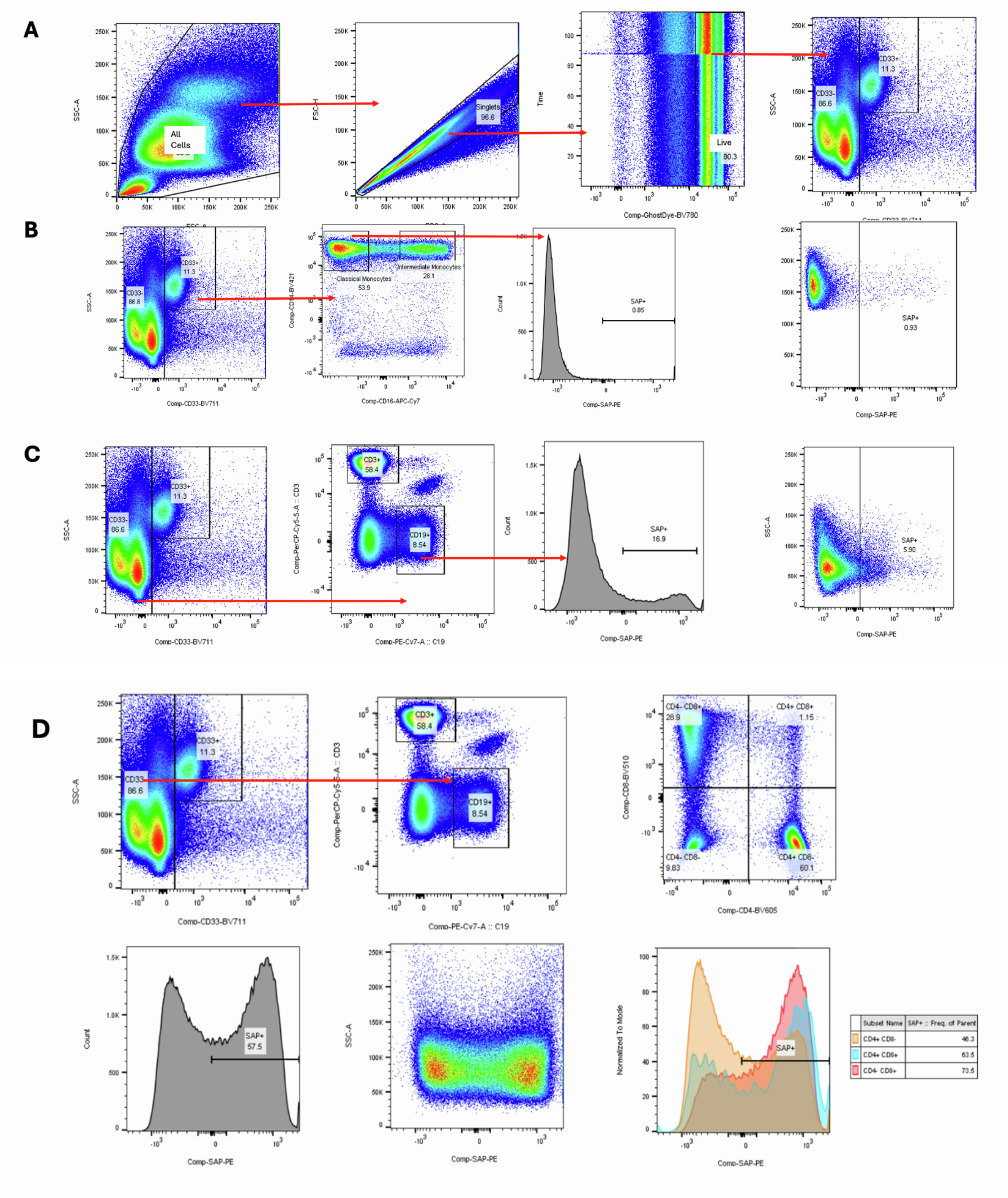

A
All Cells
SSC-A
FSC-H
Singlets
96.6
Time
Live
80.3
Comp-GhostDye-BV780
CD33+
11.3
CD33-
86.6
B
Comp-CD33-BV711
Comp-CD14-BV421
Classical Monocytes
53.9
Intermediate Monocytes
28.1
Comp-CD16-APC-Cy7
Count
SAP+
0.85
Comp-SAP-PE
SAP+
0.93
C
Comp-PerCP-Cy5-5-A :: CD3
CD3+
58.4
CD19+
8.54
Comp-PE-Cy7-A :: C19
SAP+
16.9
SAP+
5.90
D
CD4- CD8+
28.9
CD4+ CD8+
1.15
CD4- CD8-
9.83
CD4+ CD8-
60.1
Comp-CD8-BV510
Comp-CD4-BV605
SAP+
57.5
Normalized To Mode
SAP+
Subset Name | SAP+ :: Freq. of Parent
CD4+ CD8- | 46.3
CD4+ CD8+ | 63.5
CD4- CD8+ | 73.5

E

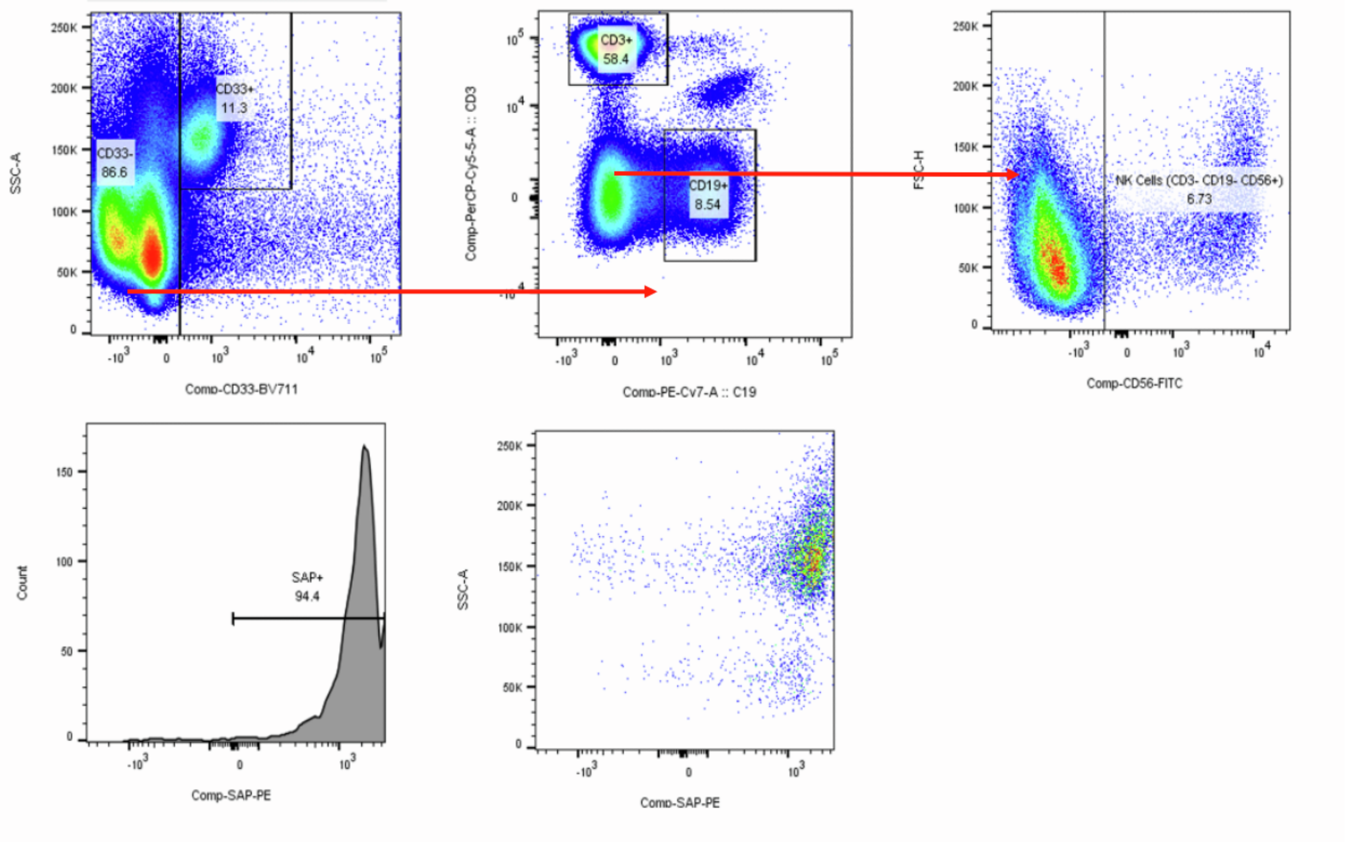
SSC-A
Comp-CD33-BV711
CD33-
86.6
CD33+
11.3
Comp-PerCP-Cy5-5-A :: CD3
Comp-PE-Cy7-A :: C19
CD3+
58.4
CD19+
8.54
FSC-H
Comp-CD56-FITC
NK Cells (CD3- CD19- CD56+)
6.73
Count
Comp-SAP-PE
SAP+
94.4
SSC-A
Comp-SAP-PE

F

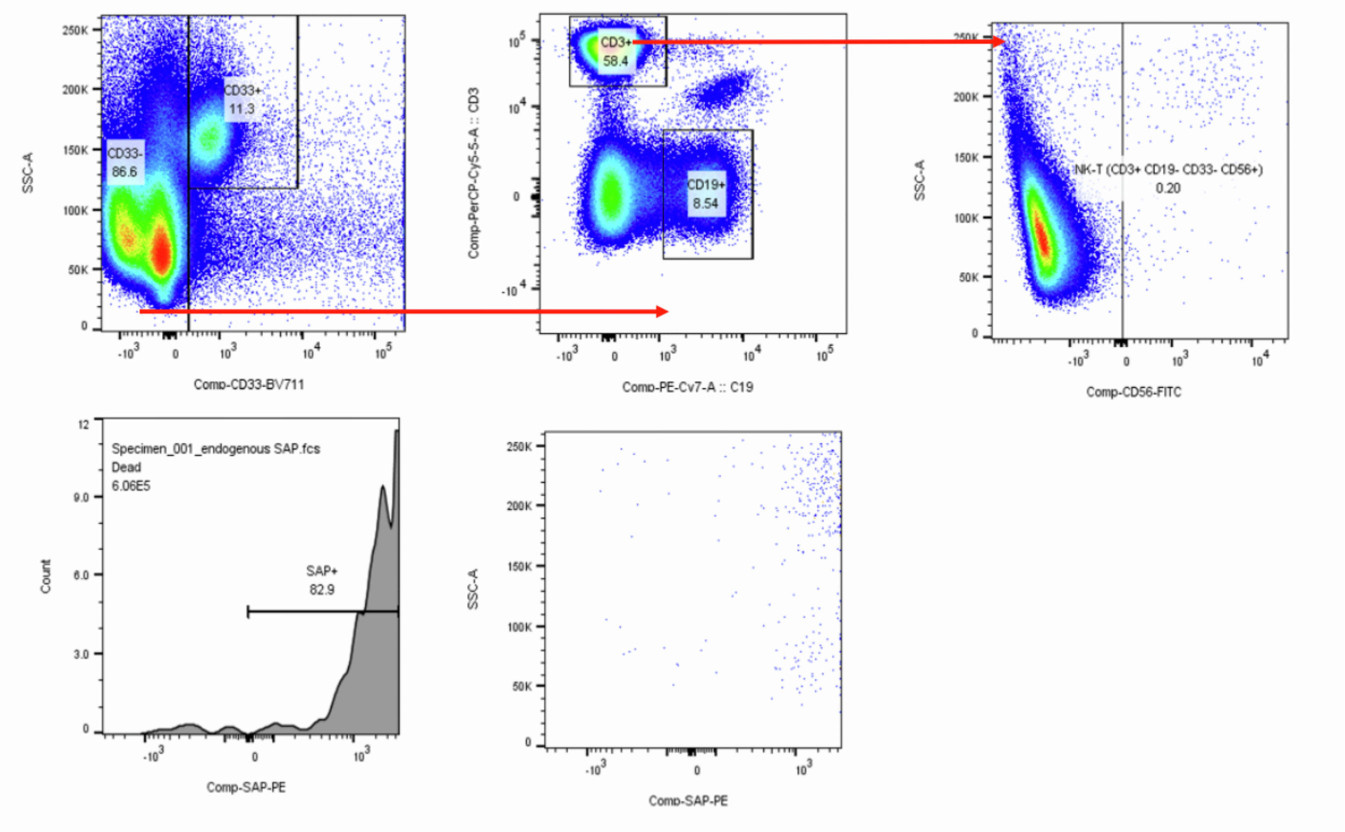
SSC-A
Comp-CD33-BV711
CD33-
86.6
CD33+
11.3
Comp-PerCP-Cy5-5-A :: CD3
Comp-PE-Cy7-A :: C19
CD3+
58.4
CD19+
8.54
SSC-A
Comp-CD56-FITC
NK-T (CD3+ CD19- CD33- CD56+)
0.20
Specimen_001_endogenous SAP.fcs
Dead
6.06E5
Count
Comp-SAP-PE
SAP+
82.9
SSC-A
Comp-SAP-PE

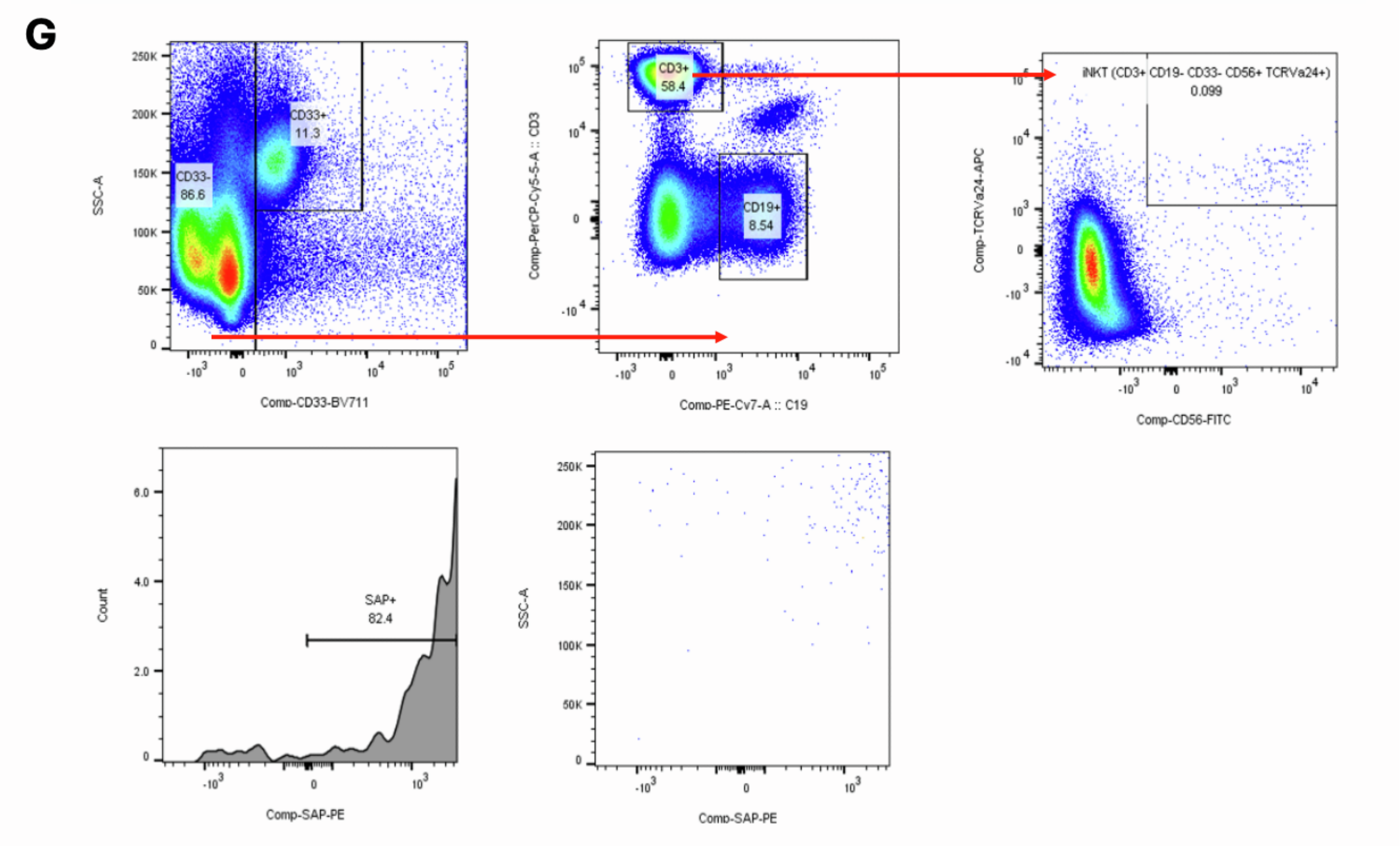

Flow Gating for SAP quantification within PBMC Lineages. (A) Parent Gating; (B) Monocytes; (C) B Cells; (D) T Cells; (E) NK Cells; (F) NKT Cells; (G) iNKT Cells.

Figure S8:

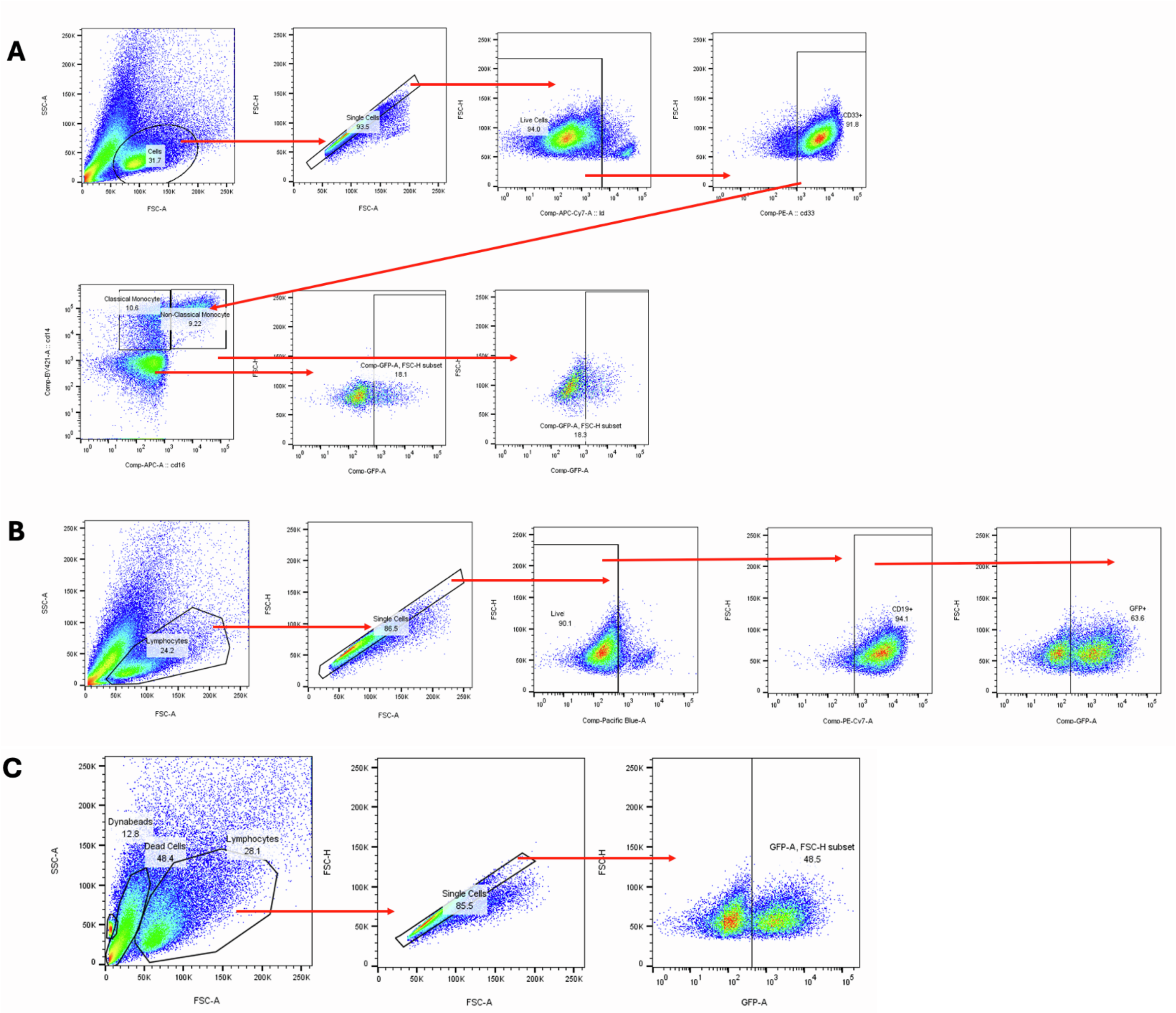

mCitrine MFI Flow Cytometry Gating for Enhancers in T Cells, Monocytes, and B-LCLs. (A) Monocyte gating; (B) B-LCL gating; (C) T Cell gating.

Figure S9:

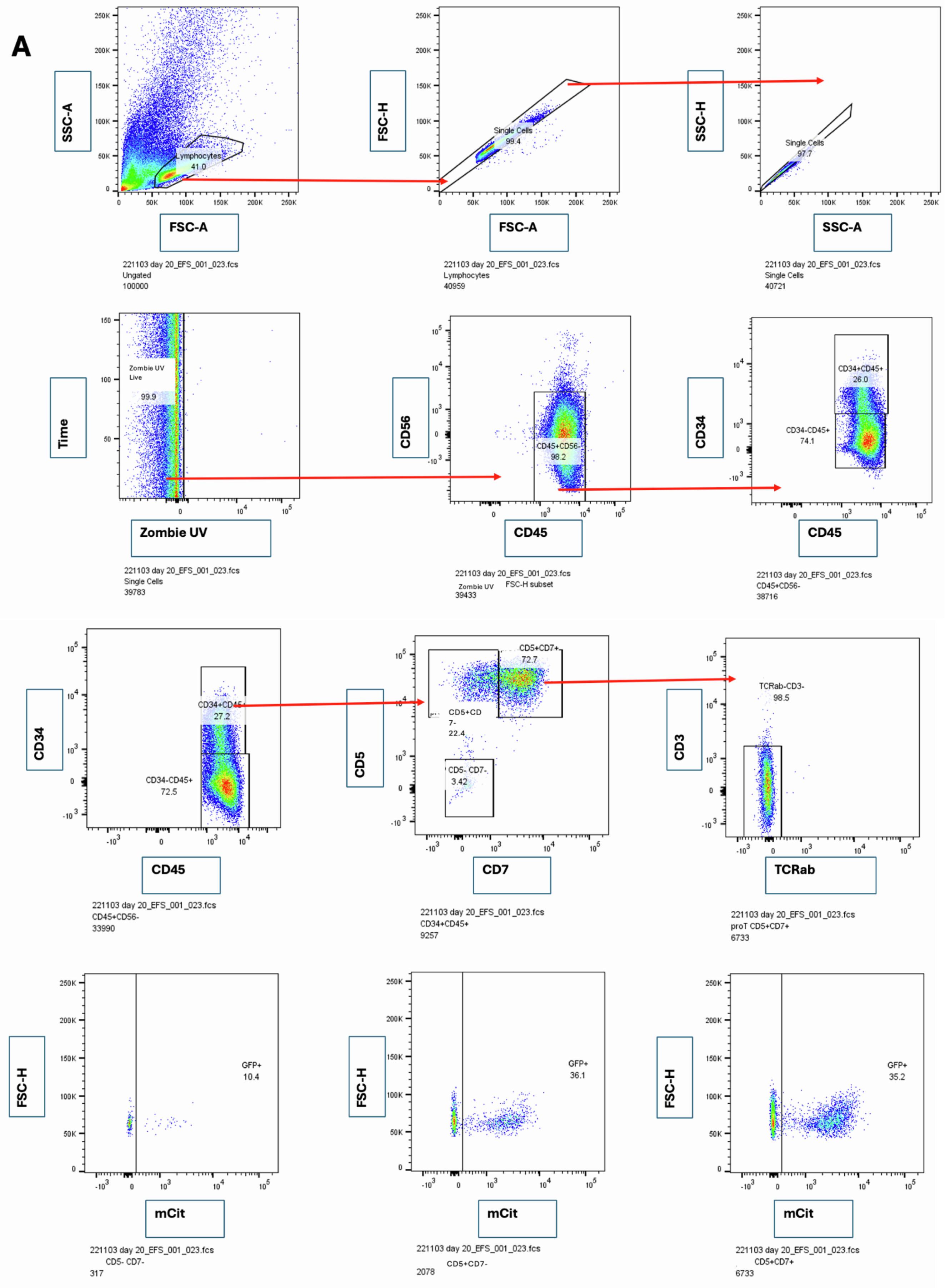
A
SSC-A
FSC-A
Lymphocytes
41.0
221103 day 20_EFS_001_023.fcs
Ungated
100000
FSC-H
FSC-A
Single Cells
99.4
221103 day 20_EFS_001_023.fcs
Lymphocytes
40959
SSC-H
SSC-A
Single Cells
97.7
221103 day 20_EFS_001_023.fcs
Single Cells
40721
Time
Zombie UV
Zombie UV Live
99.9
221103 day 20_EFS_001_023.fcs
Single Cells
39783
CD56
CD45
CD45+CD56-
98.2
221103 day 20_EFS_001_023.fcs
Zombie UV FSC-H subset
39433
CD34
CD45
CD34+CD45+
26.0
CD34-CD45+
74.1
221103 day 20_EFS_001_023.fcs
CD45+CD56-
38716
CD34
CD45
CD34+CD45+
27.2
CD34-CD45+
72.5
221103 day 20_EFS_001_023.fcs
CD45+CD56-
33990
CD5
CD7
CD5+CD7+
72.7
CD5+CD7-
22.4
CD5- CD7-
3.42
221103 day 20_EFS_001_023.fcs
CD34+CD45+
9257
CD3
TCRab
TCRab-CD3-
98.5
221103 day 20_EFS_001_023.fcs
proT CD5+CD7+
6733
FSC-H
mCit
GFP+
10.4
221103 day 20_EFS_001_023.fcs
CD5- CD7-
317
FSC-H
mCit
GFP+
36.1
221103 day 20_EFS_001_023.fcs
CD5+CD7-
2078
FSC-H
mCit
GFP+
35.2
221103 day 20_EFS_001_023.fcs
CD5+CD7+
6733

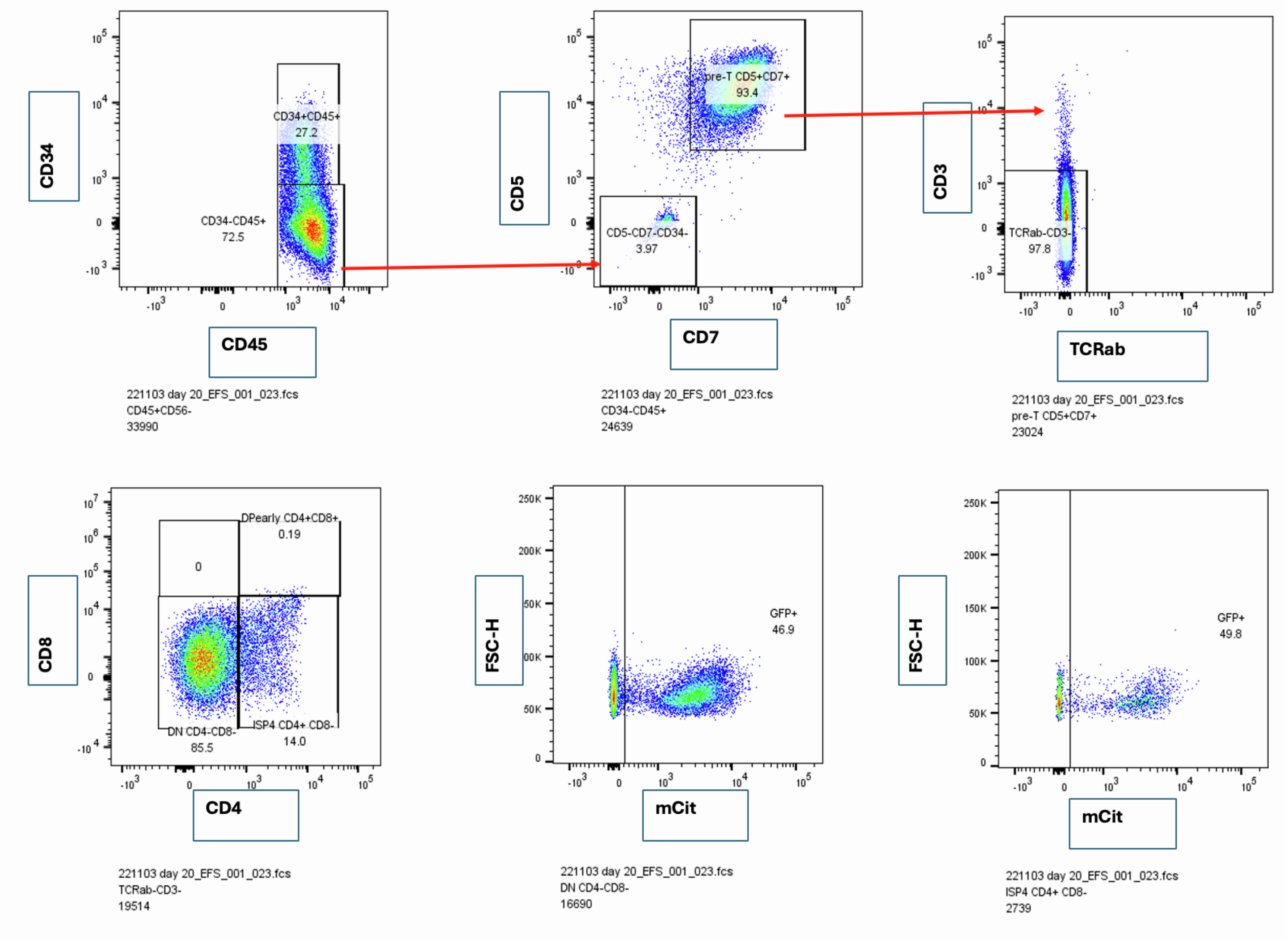
CD34
CD34+CD45+
27.2
CD34-CD45+
72.5
CD45
221103 day 20_EFS_001_023.fcs
CD45+CD56-
33990
CD5
pre-T CD5+CD7+
93.4
CD5-CD7-CD34-
3.97
CD7
221103 day 20_EFS_001_023.fcs
CD34-CD45+
24639
CD3
TCRab-CD3-
97.8
TCRab
221103 day 20_EFS_001_023.fcs
pre-T CD5+CD7+
23024
CD8
DPearly CD4+CD8+
0.19
0
DN CD4-CD8-
85.5
ISP4 CD4+ CD8-
14.0
CD4
221103 day 20_EFS_001_023.fcs
TCRab-CD3-
19514
FSC-H
GFP+
46.9
mCit
221103 day 20_EFS_001_023.fcs
DN CD4-CD8-
16690
FSC-H
GFP+
49.8
mCit
221103 day 20_EFS_001_023.fcs
ISP4 CD4+ CD8-
2739

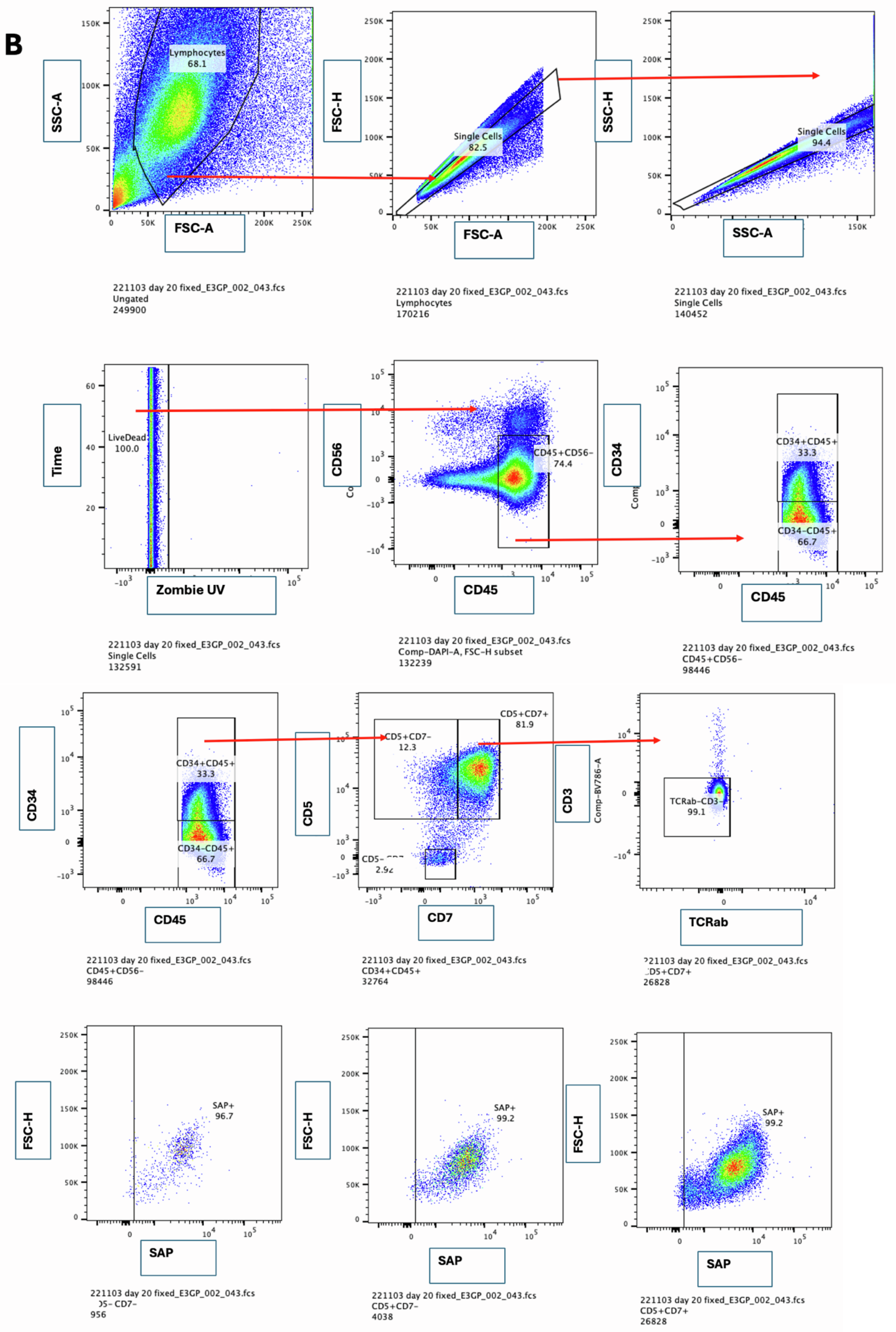

B
SSC-A
FSC-A
Lymphocytes
68.1
221103 day 20 fixed_E3GP_002_043.fcs
Ungated
249900
FSC-H
FSC-A
Single Cells
82.5
221103 day 20 fixed_E3GP_002_043.fcs
Lymphocytes
170216
SSC-H
SSC-A
Single Cells
94.4
221103 day 20 fixed_E3GP_002_043.fcs
Single Cells
140452
Time
Zombie UV
LiveDead
100.0
221103 day 20 fixed_E3GP_002_043.fcs
Single Cells
132591
CD56
CD45
CD45+CD56-
74.4
221103 day 20 fixed_E3GP_002_043.fcs
Comp-DAPI-A, FSC-H subset
132239
CD34
CD45
CD34+CD45+
33.3
CD34-CD45+
66.7
221103 day 20 fixed_E3GP_002_043.fcs
CD45+CD56-
98446
CD34
CD45
CD34+CD45+
33.3
CD34-CD45+
66.7
221103 day 20 fixed_E3GP_002_043.fcs
CD45+CD56-
98446
CD5
CD7
CD5+CD7-
12.3
CD5+CD7+
81.9
2.92
221103 day 20 fixed_E3GP_002_043.fcs
CD34+CD45+
32764
CD3
Comp-BV786-A
TCRab
TCRab-CD3-
99.1
221103 day 20 fixed_E3GP_002_043.fcs
CD5+CD7+
26828
FSC-H
SAP
SAP+
96.7
221103 day 20 fixed_E3GP_002_043.fcs
CD7-
956
FSC-H
SAP
SAP+
99.2
221103 day 20 fixed_E3GP_002_043.fcs
CD5+CD7-
4038
FSC-H
SAP
SAP+
99.2
221103 day 20 fixed_E3GP_002_043.fcs
CD5+CD7+
26828

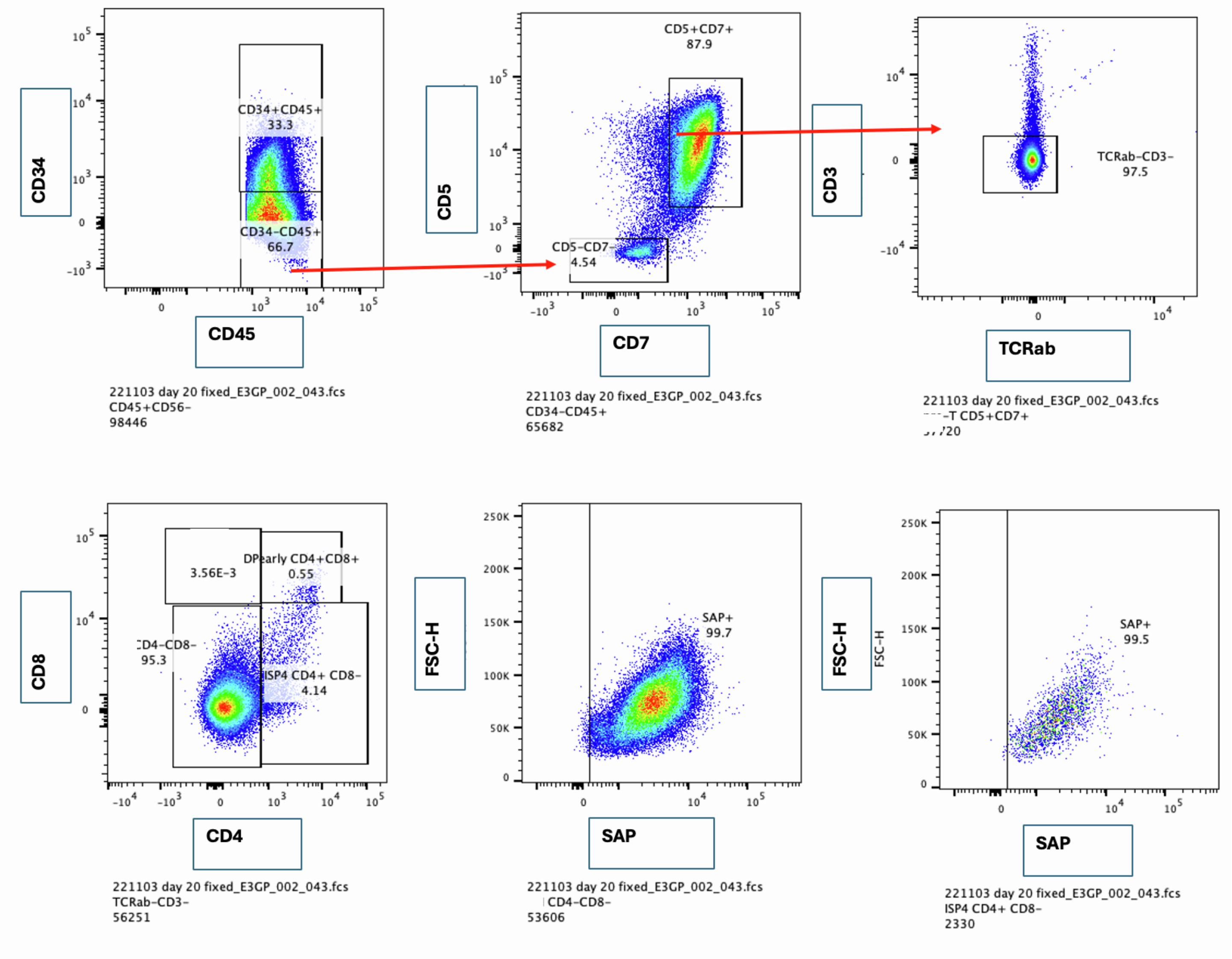
CD34
CD45
CD34+CD45+
33.3
CD34-CD45+
66.7
221103 day 20 fixed_E3GP_002_043.fcs
CD45+CD56-
98446
CD5
CD7
CD5+CD7+
87.9
CD5-CD7-
4.54
221103 day 20 fixed_E3GP_002_043.fcs
CD34-CD45+
65682
CD3
TCRab
TCRab-CD3-
97.5
221103 day 20 fixed_E3GP_002_043.fcs
CD5+CD7+
CD8
CD4
3.56E-3
DPearly CD4+CD8+
0.55
95.3
ISP4 CD4+ CD8-
4.14
221103 day 20 fixed_E3GP_002_043.fcs
TCRab-CD3-
56251
FSC-H
SAP
SAP+
99.7
221103 day 20 fixed_E3GP_002_043.fcs
CD4-CD8-
53606
FSC-H
SAP
SAP+
99.5
221103 day 20 fixed_E3GP_002_043.fcs
ISP4 CD4+ CD8-
2330

c

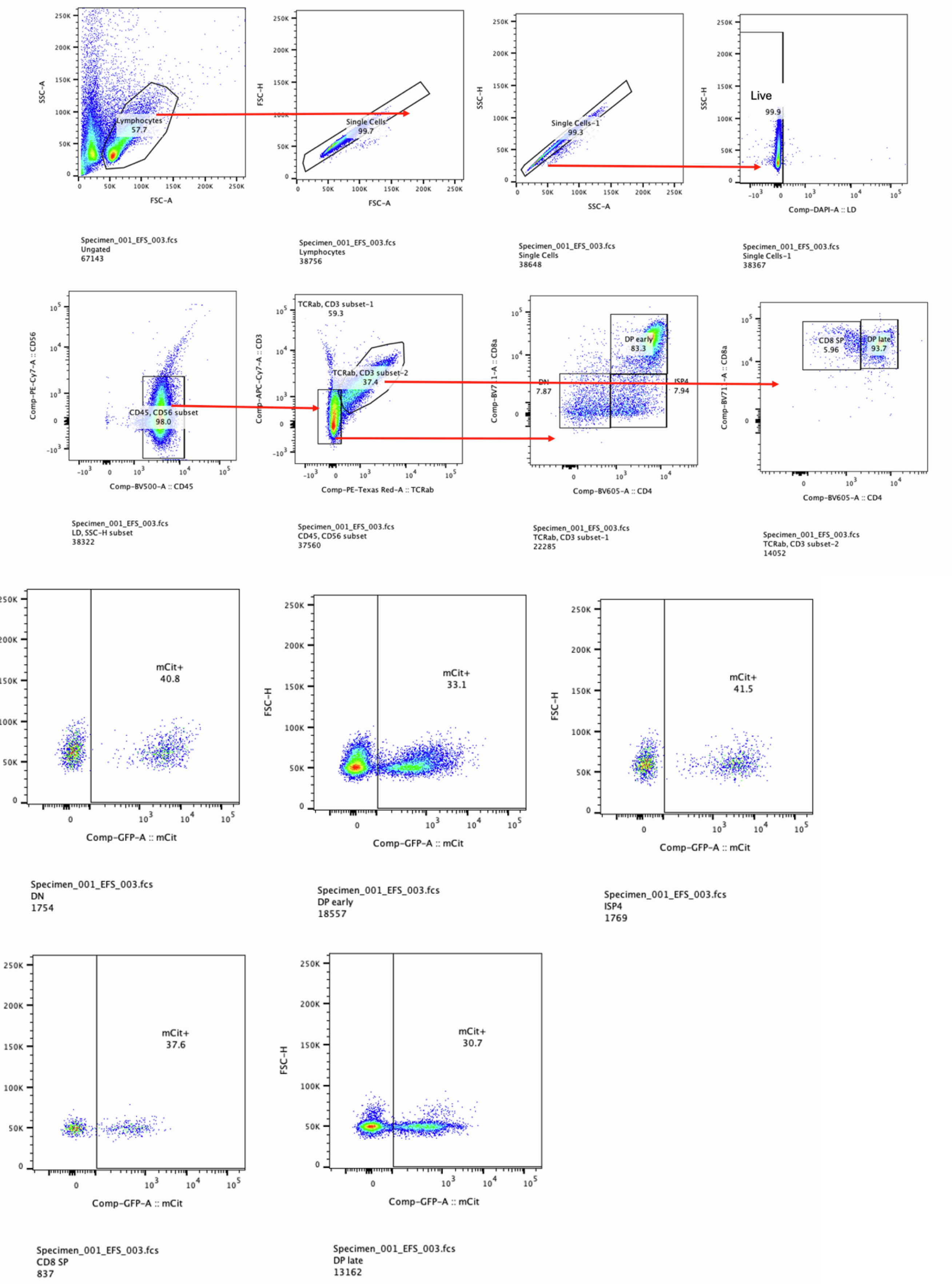
Lymphocytes
57.7
SSC-A
FSC-A
Specimen_001_EFS_003.fcs
Ungated
67143
Single Cells
99.7
FSC-H
FSC-A
Specimen_001_EFS_003.fcs
Lymphocytes
38756
Single Cells-1
99.3
SSC-H
SSC-A
Specimen_001_EFS_003.fcs
Single Cells
38648
Live
99.9
SSC-H
Comp-DAPI-A :: LD
Specimen_001_EFS_003.fcs
Single Cells-1
38367
CD45, CD56 subset
98.0
Comp-PE-Cy7-A :: CD56
Comp-BV500-A :: CD45
Specimen_001_EFS_003.fcs
LD, SSC-H subset
38322
TCRab, CD3 subset-1
59.3
TCRab, CD3 subset-2
37.4
Comp-APC-Cy7-A :: CD3
Comp-PE-Texas Red-A :: TCRab
Specimen_001_EFS_003.fcs
CD45, CD56 subset
37560
DP early
83.3
DN
7.87
ISP4
7.94
Comp-BV711-A :: CD8a
Comp-BV605-A :: CD4
Specimen_001_EFS_003.fcs
TCRab, CD3 subset-1
22285
CD8 SP
5.96
DP late
93.7
Comp-BV711-A :: CD8a
Comp-BV605-A :: CD4
Specimen_001_EFS_003.fcs
TCRab, CD3 subset-2
14052
mCit+
40.8
FSC-H
Comp-GFP-A :: mCit
Specimen_001_EFS_003.fcs
DN
1754
mCit+
33.1
FSC-H
Comp-GFP-A :: mCit
Specimen_001_EFS_003.fcs
DP early
18557
mCit+
41.5
FSC-H
Comp-GFP-A :: mCit
Specimen_001_EFS_003.fcs
ISP4
1769
mCit+
37.6
FSC-H
Comp-GFP-A :: mCit
Specimen_001_EFS_003.fcs
CD8 SP
837
mCit+
30.7
FSC-H
Comp-GFP-A :: mCit
Specimen_001_EFS_003.fcs
DP late
13162

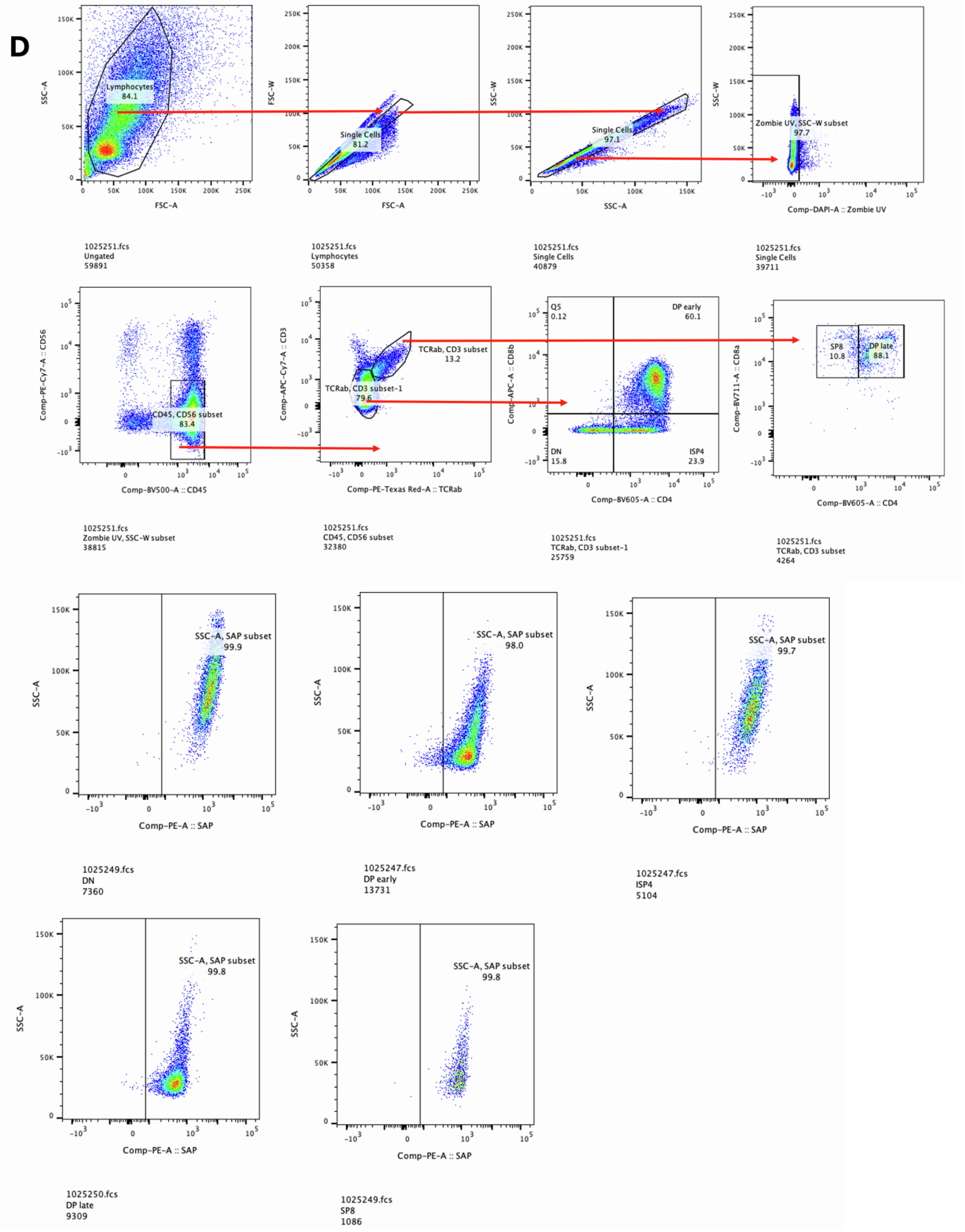
D
Lymphocytes
84.1
SSC-A
FSC-A
1025251.fcs
Ungated
59891
Single Cells
81.2
FSC-W
FSC-A
1025251.fcs
Lymphocytes
50358
Single Cells
97.1
SSC-W
SSC-A
1025251.fcs
Single Cells
40879
Zombie UV, SSC-W subset
97.7
SSC-W
Comp-DAPI-A :: Zombie UV
1025251.fcs
Single Cells
39711
CD45, CD56 subset
83.4
Comp-PE-Cy7-A :: CD56
Comp-BV500-A :: CD45
1025251.fcs
Zombie UV, SSC-W subset
38815
TCRab, CD3 subset
13.2
TCRab, CD3 subset-1
79.6
Comp-APC-Cy7-A :: CD3
Comp-PE-Texas Red-A :: TCRab
1025251.fcs
CD45, CD56 subset
32380
Q5
0.12
DP early
60.1
DN
15.8
ISP4
23.9
Comp-APC-A :: CD8b
Comp-BV605-A :: CD4
1025251.fcs
TCRab, CD3 subset-1
25759
SP8
10.8
DP late
88.1
Comp-BV711-A :: CD8a
Comp-BV605-A :: CD4
1025251.fcs
TCRab, CD3 subset
4264
SSC-A, SAP subset
99.9
SSC-A
Comp-PE-A :: SAP
1025249.fcs
DN
7360
SSC-A, SAP subset
98.0
SSC-A
Comp-PE-A :: SAP
1025247.fcs
DP early
13731
SSC-A, SAP subset
99.7
SSC-A
Comp-PE-A :: SAP
1025247.fcs
ISP4
5104
SSC-A, SAP subset
99.8
SSC-A
Comp-PE-A :: SAP
1025250.fcs
DP late
9309
SSC-A, SAP subset
99.8
SSC-A
Comp-PE-A :: SAP
1025249.fcs
SP8
1086

E

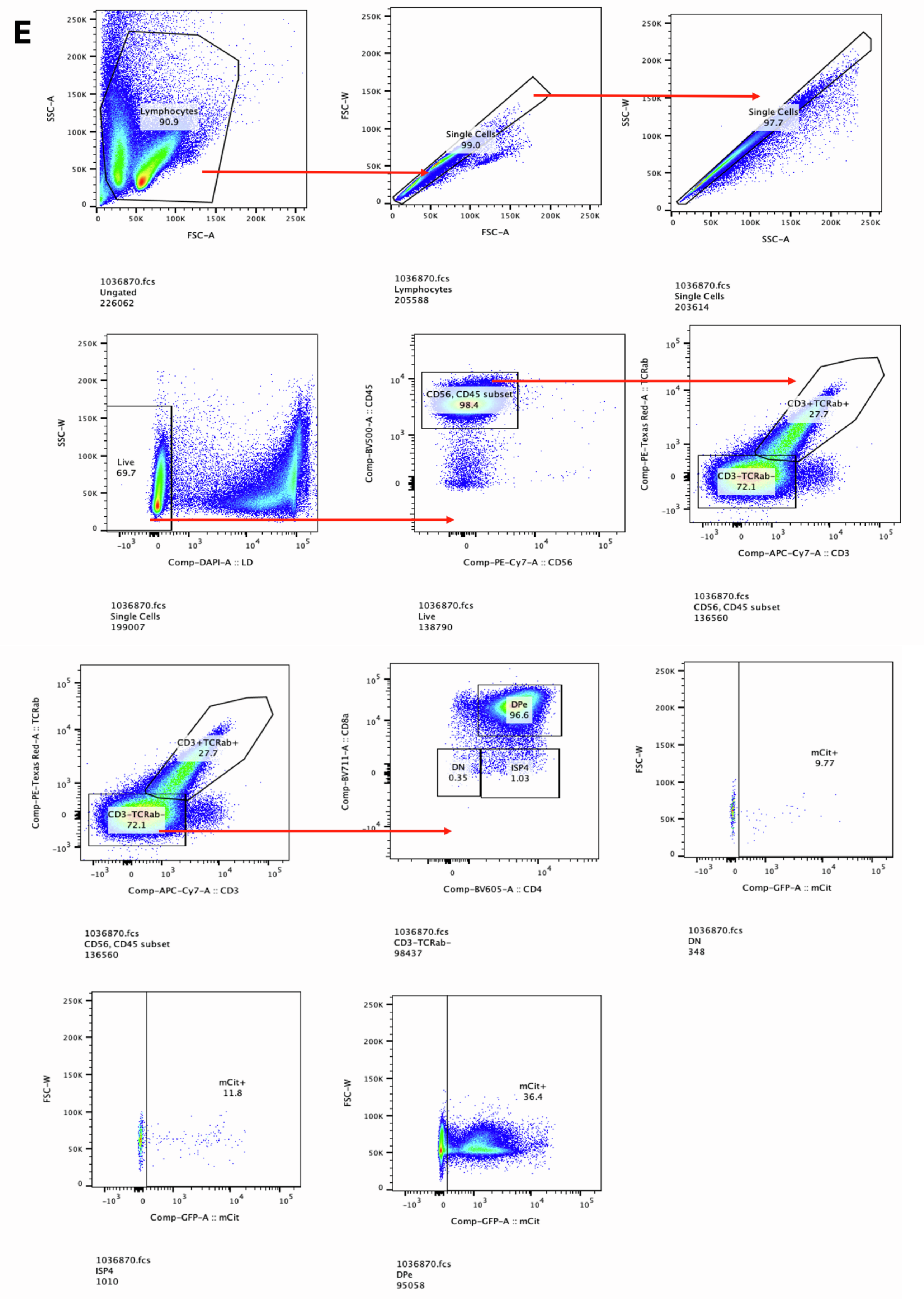
Lymphocytes
90.9
SSC-A
FSC-A
1036870.fcs
Ungated
226062
Single Cells
99.0
FSC-W
FSC-A
1036870.fcs
Lymphocytes
205588
Single Cells
97.7
SSC-W
SSC-A
1036870.fcs
Single Cells
203614
Live
69.7
SSC-W
Comp-DAPI-A :: LD
1036870.fcs
Single Cells
199007
CD56, CD45 subset
98.4
Comp-BV500-A :: CD45
Comp-PE-Cy7-A :: CD56
1036870.fcs
Live
138790
CD3+TCRab+
27.7
CD3-TCRab-
72.1
Comp-PE-Texas Red-A :: TCRab
Comp-APC-Cy7-A :: CD3
1036870.fcs
CD56, CD45 subset
136560
CD3+TCRab+
27.7
CD3-TCRab-
72.1
Comp-PE-Texas Red-A :: TCRab
Comp-APC-Cy7-A :: CD3
1036870.fcs
CD56, CD45 subset
136560
DPe
96.6
DN
0.35
ISP4
1.03
Comp-BV711-A :: CD8a
Comp-BV605-A :: CD4
1036870.fcs
CD3-TCRab-
98437
mCit+
9.77
FSC-W
Comp-GFP-A :: mCit
1036870.fcs
DN
348
mCit+
11.8
FSC-W
Comp-GFP-A :: mCit
1036870.fcs
ISP4
1010
mCit+
36.4
FSC-W
Comp-GFP-A :: mCit
1036870.fcs
DPe
95058

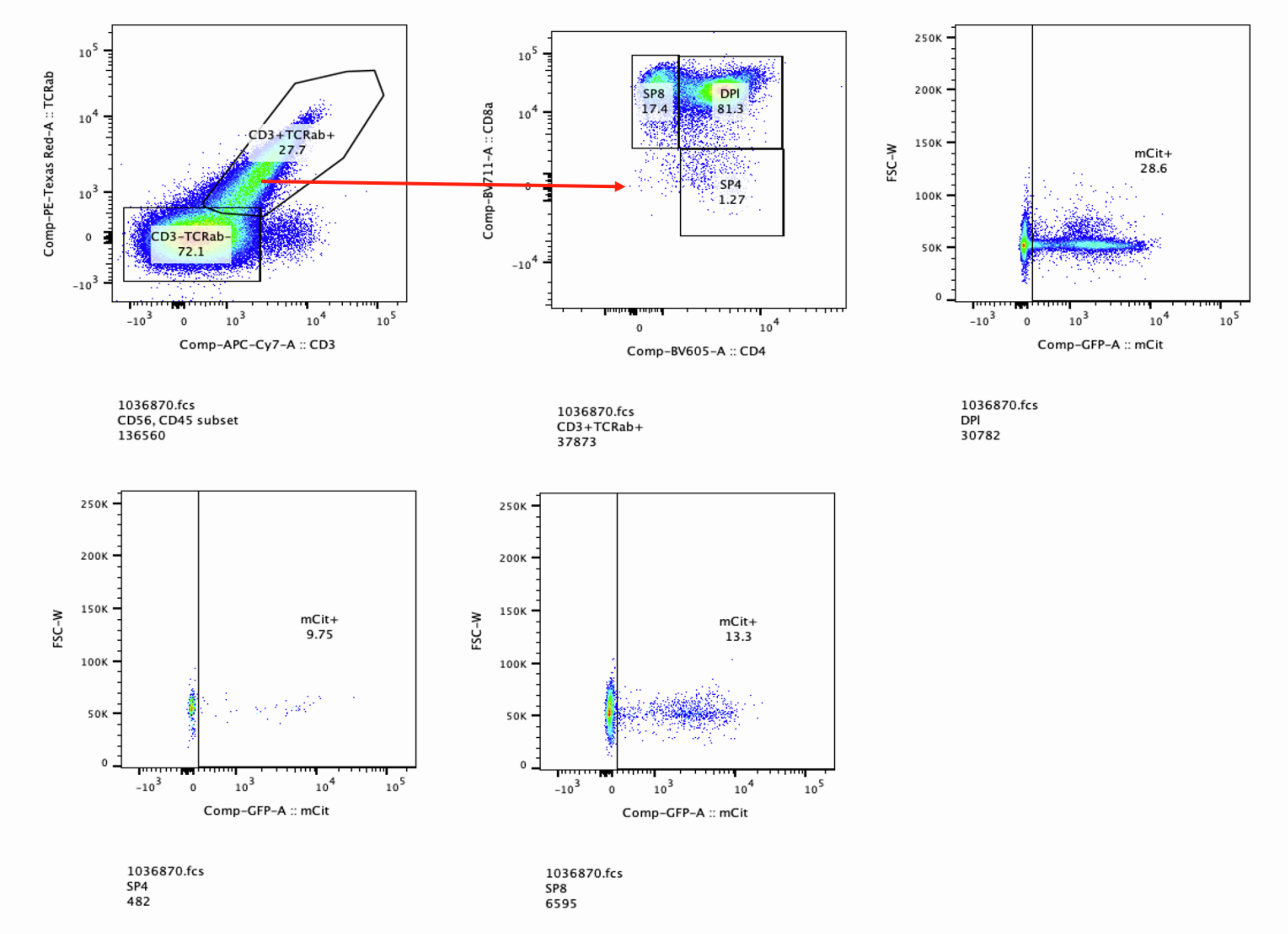
CD3+TCRab+
27.7
CD3-TCRab-
72.1
Comp-PE-Texas Red-A :: TCRab
Comp-APC-Cy7-A :: CD3
1036870.fcs
CD56, CD45 subset
136560
SP8
17.4
DPl
81.3
SP4
1.27
Comp-BV711-A :: CD8a
Comp-BV605-A :: CD4
1036870.fcs
CD3+TCRab+
37873
mCit+
28.6
FSC-W
Comp-GFP-A :: mCit
1036870.fcs
DPl
30782
mCit+
9.75
FSC-W
Comp-GFP-A :: mCit
1036870.fcs
SP4
482
mCit+
13.3
FSC-W
Comp-GFP-A :: mCit
1036870.fcs
SP8
6595

F

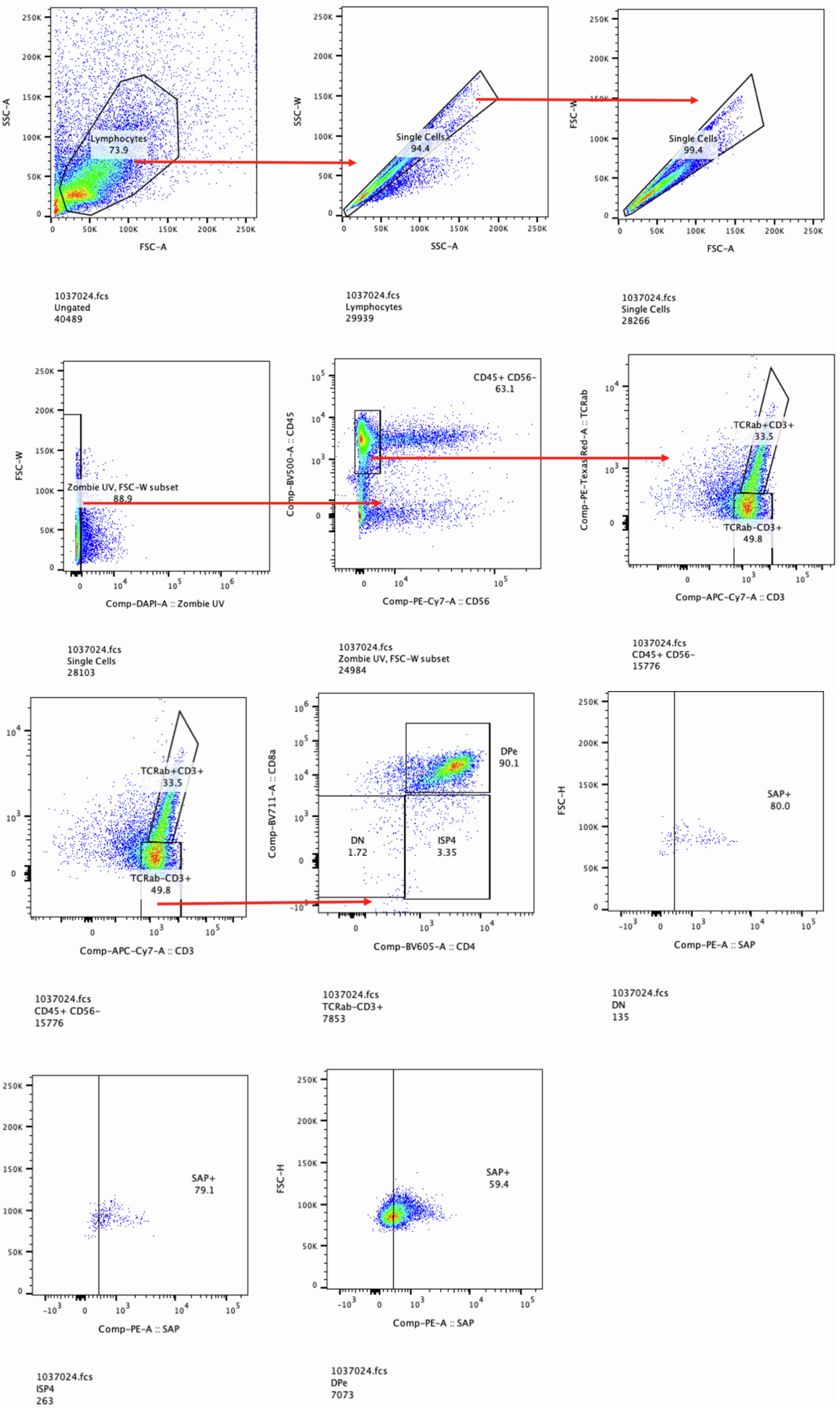

SSC-A
FSC-A
Lymphocytes
73.9
1037024.fcs
Ungated
40489
SSC-W
SSC-A
Single Cells
94.4
1037024.fcs
Lymphocytes
29939
FSC-W
FSC-A
Single Cells
99.4
1037024.fcs
Single Cells
28266
FSC-W
Comp-DAPI-A :: Zombie UV
Zombie UV, FSC-W subset
88.9
1037024.fcs
Single Cells
28103
Comp-BV500-A :: CD45
Comp-PE-Cy7-A :: CD56
CD45+ CD56-
63.1
1037024.fcs
Zombie UV, FSC-W subset
24984
Comp-PE-Texas Red-A :: TCRab
Comp-APC-Cy7-A :: CD3
TCRab+CD3+
33.5
TCRab-CD3+
49.8
1037024.fcs
CD45+ CD56-
15776
Comp-PE-Texas Red-A :: TCRab
Comp-APC-Cy7-A :: CD3
TCRab+CD3+
33.5
TCRab-CD3+
49.8
1037024.fcs
CD45+ CD56-
15776
Comp-BV711-A :: CD8a
Comp-BV605-A :: CD4
DPe
90.1
DN
1.72
ISP4
3.35
1037024.fcs
TCRab-CD3+
7853
FSC-H
Comp-PE-A :: SAP
SAP+
80.0
1037024.fcs
DN
135
FSC-H
Comp-PE-A :: SAP
SAP+
79.1
1037024.fcs
ISP4
263
FSC-H
Comp-PE-A :: SAP
SAP+
59.4
1037024.fcs
DPe
7073

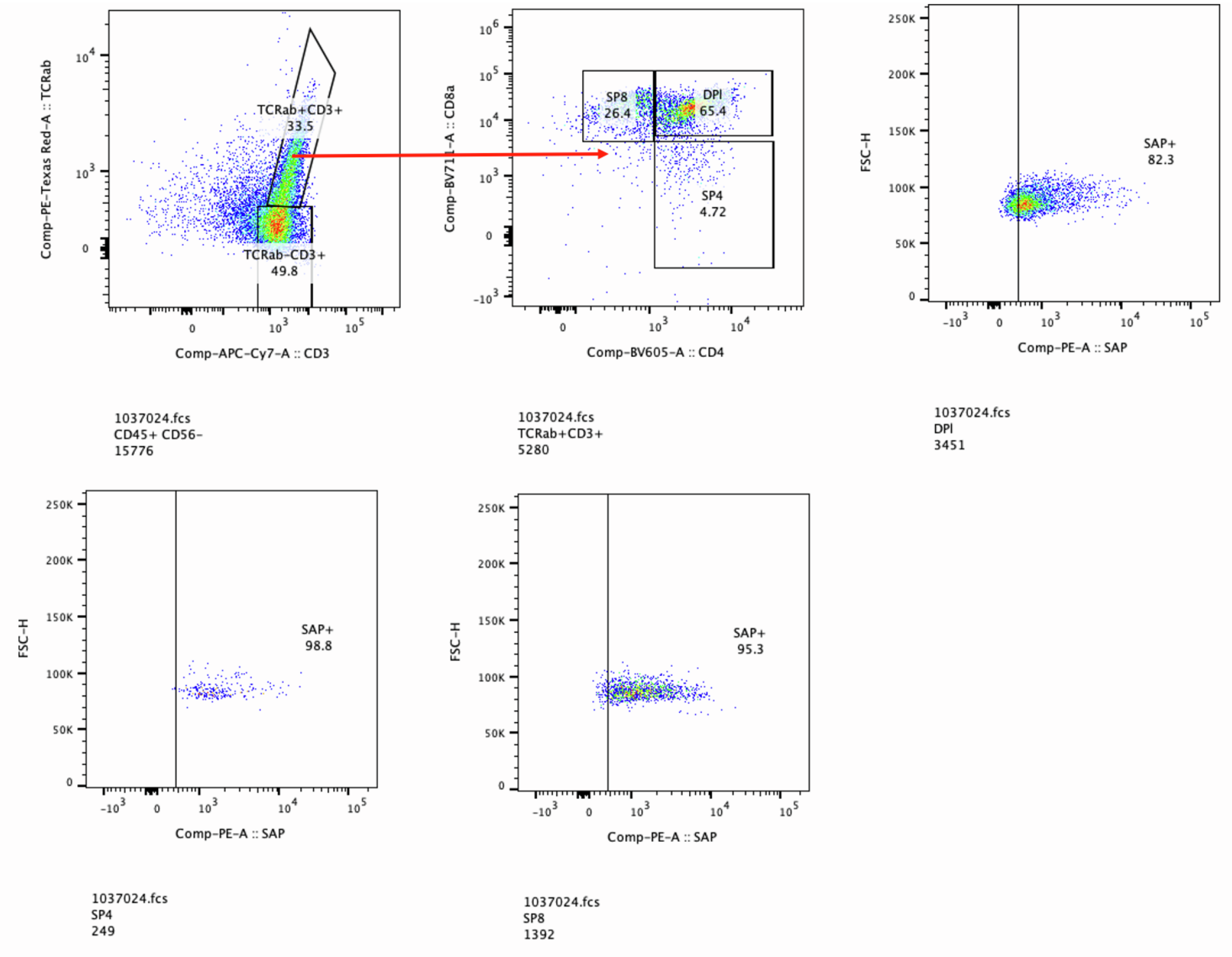

ATO Flow Cytometry SAP and mCitrine staining across differentiation. ATOs were stained to differentiate the following stages of T cell development: double negative (DN): hTCRab-hCD3-hCD4-hCD8-; immature single positive 4 (ISP4): hTCRab-hCD3-hCD4+hCD8-; double positive (DP) early: hTCRab-hCD3-hCD4+hCD8+; DP late: hTCRab+hCD3+hCD4+hCD8+; single positive (SP) 4: hTCRab+hCD3+hCD4+hCD8-; and SP8: hTCRab+hCD3+hCD4-hCD8+. Representative flow plots are shown above for mCitrine week 3 (A), SAP week 3 (B), mCitrine week 7 (C), SAP week 7 (D), mCitrine week 12 (E), and SAP week 12 (F).

Figure S9:

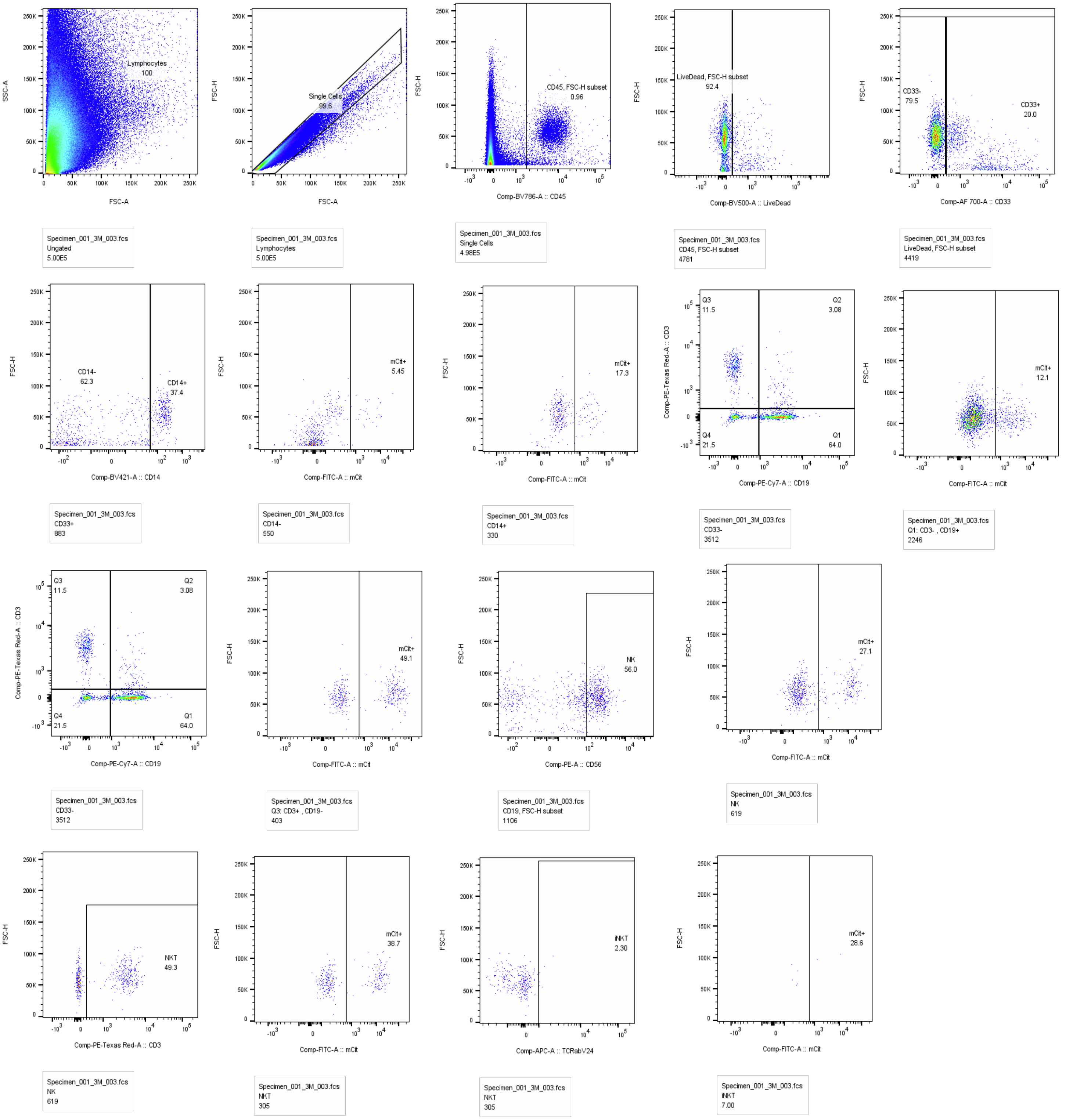

Representative flow cytometry gating strategy of peripheral blood lineages in hIL15-NSG mice.

Figure S10:

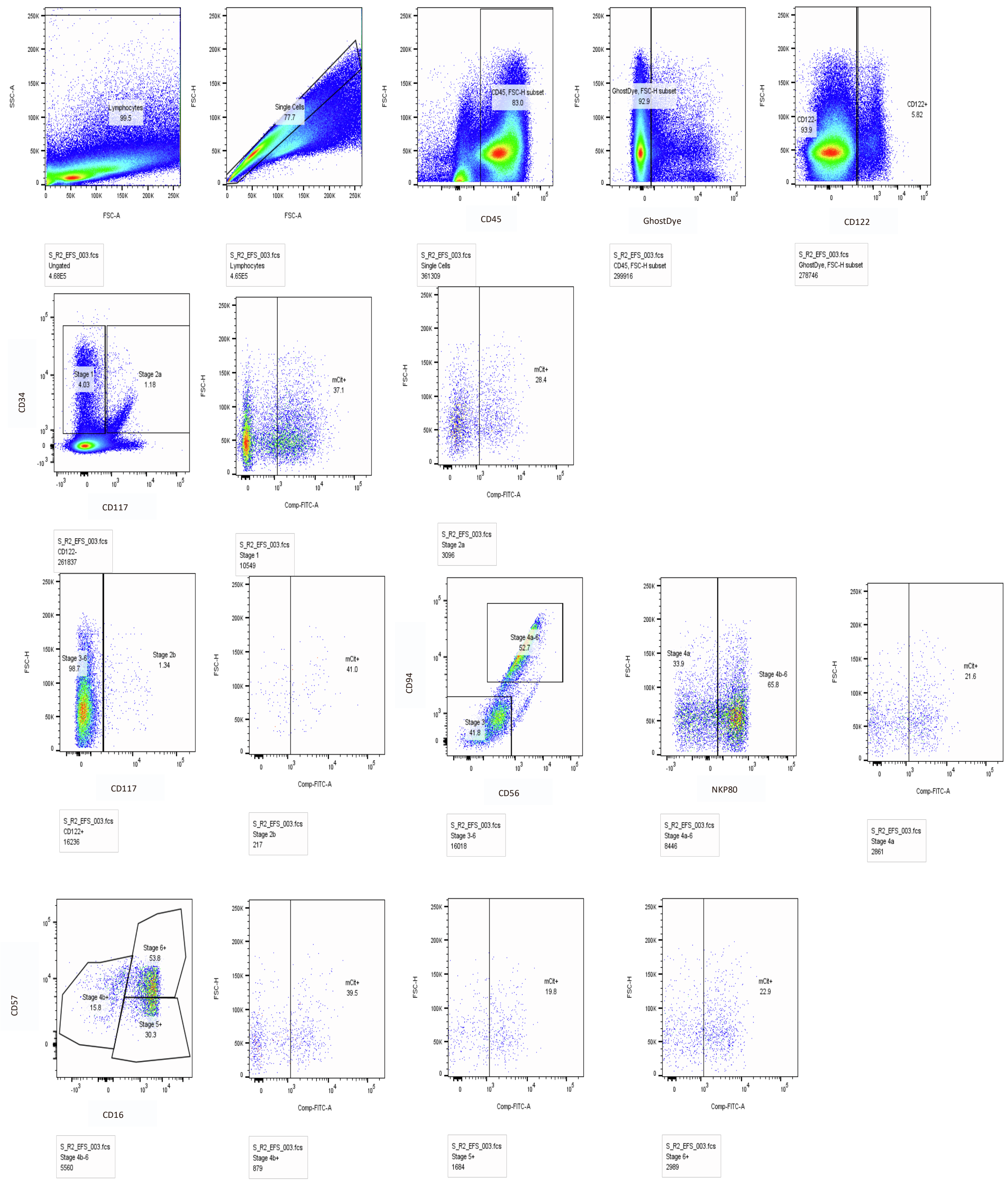

Representative flow cytometry gating strategy of NK cells in the spleen of hIL15-NSG mice.

Figure S11:

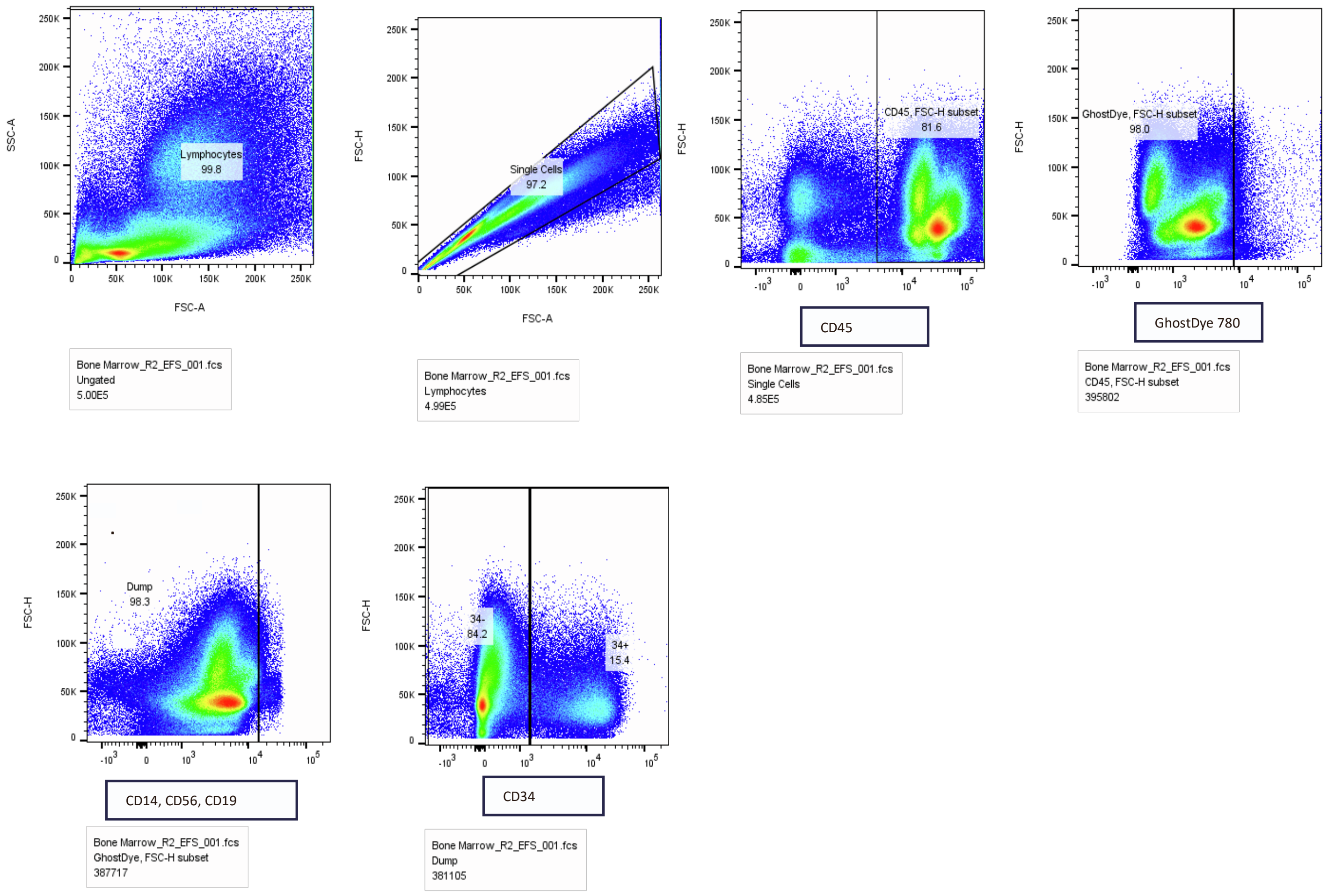

Lymphocytes
99.8
SSC-A
FSC-A
Bone Marrow_R2_EFS_001.fcs
Ungated
5.00E5
Single Cells
97.2
FSC-H
FSC-A
Bone Marrow_R2_EFS_001.fcs
Lymphocytes
4.99E5
CD45, FSC-H subset
81.6
FSC-H
CD45
Bone Marrow_R2_EFS_001.fcs
Single Cells
4.85E5
GhostDye, FSC-H subset
98.0
FSC-H
GhostDye 780
Bone Marrow_R2_EFS_001.fcs
CD45, FSC-H subset
395802
Dump
98.3
FSC-H
CD14, CD56, CD19
Bone Marrow_R2_EFS_001.fcs
GhostDye, FSC-H subset
387717
34-
84.2
34+
15.4
FSC-H
CD34
Bone Marrow_R2_EFS_001.fcs
Dump
381105

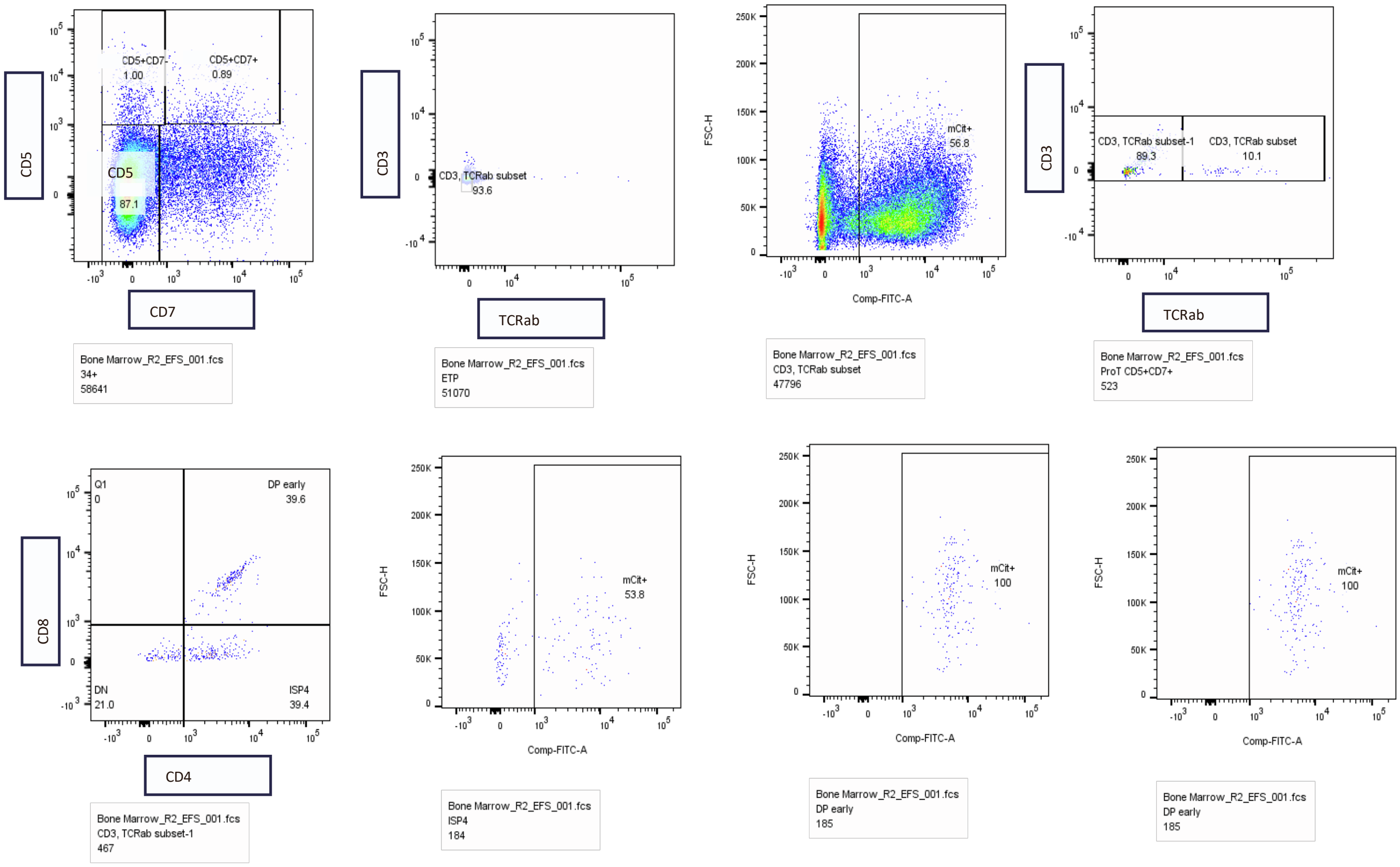
CD5
CD7
CD5+CD7-
1.00
CD5+CD7+
0.89
CD5
87.1
Bone Marrow_R2_EFS_001.fcs
34+
58641
CD3
TCRab
CD3, TCRab subset
93.6
Bone Marrow_R2_EFS_001.fcs
ETP
51070
FSC-H
Comp-FITC-A
mCit+
56.8
Bone Marrow_R2_EFS_001.fcs
CD3, TCRab subset
47796
CD3
TCRab
CD3, TCRab subset-1
89.3
CD3, TCRab subset
10.1
Bone Marrow_R2_EFS_001.fcs
ProT CD5+CD7+
523
CD8
CD4
Q1
0
DP early
39.6
DN
21.0
ISP4
39.4
Bone Marrow_R2_EFS_001.fcs
CD3, TCRab subset-1
467
FSC-H
Comp-FITC-A
mCit+
53.8
Bone Marrow_R2_EFS_001.fcs
ISP4
184
FSC-H
Comp-FITC-A
mCit+
100
Bone Marrow_R2_EFS_001.fcs
DP early
185
FSC-H
Comp-FITC-A
mCit+
100
Bone Marrow_R2_EFS_001.fcs
DP early
185

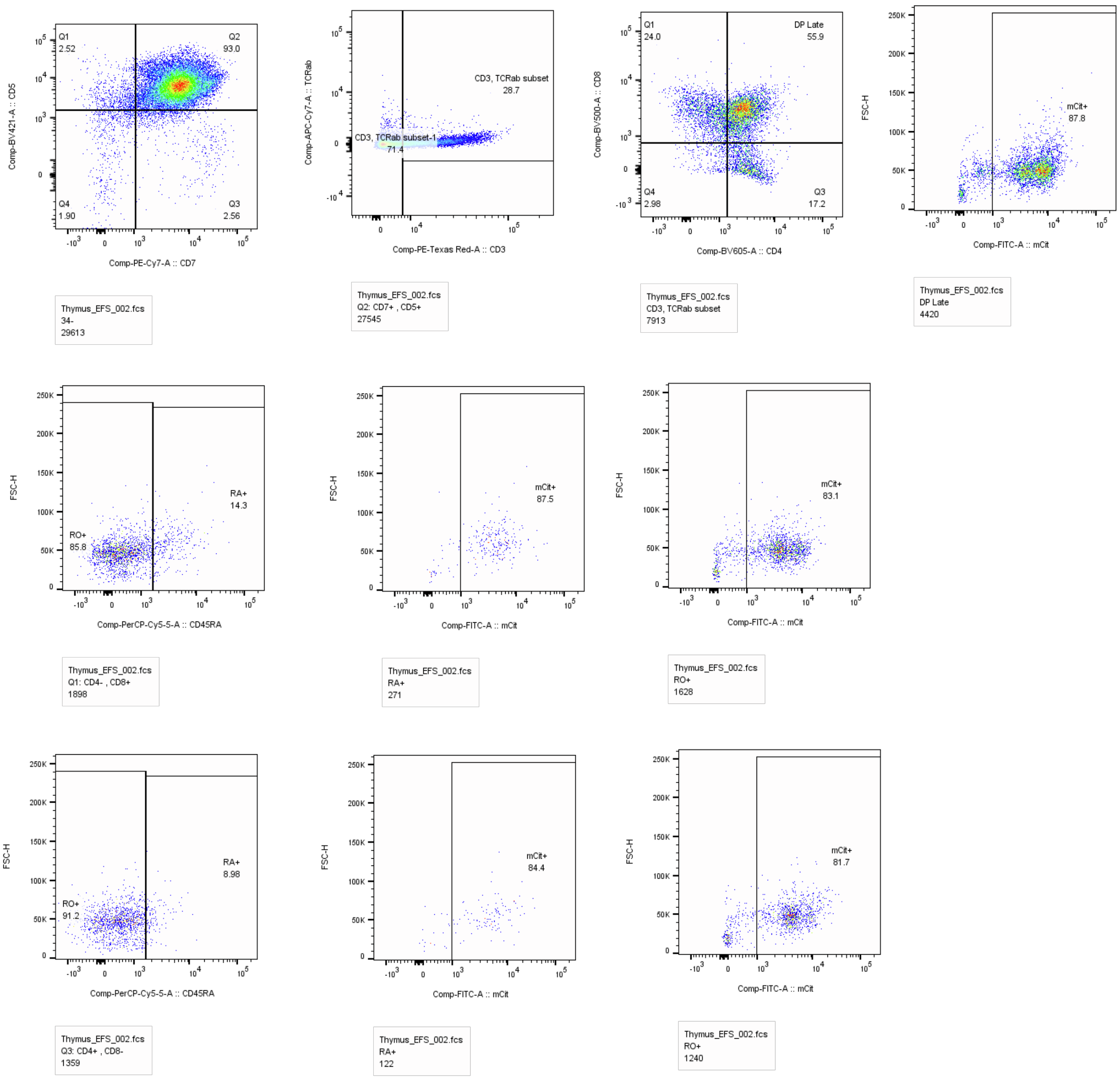

Representative flow cytometry gating strategy of T cells in the bone marrow and thymus of hIL15-NSG mice.

Figure S12:

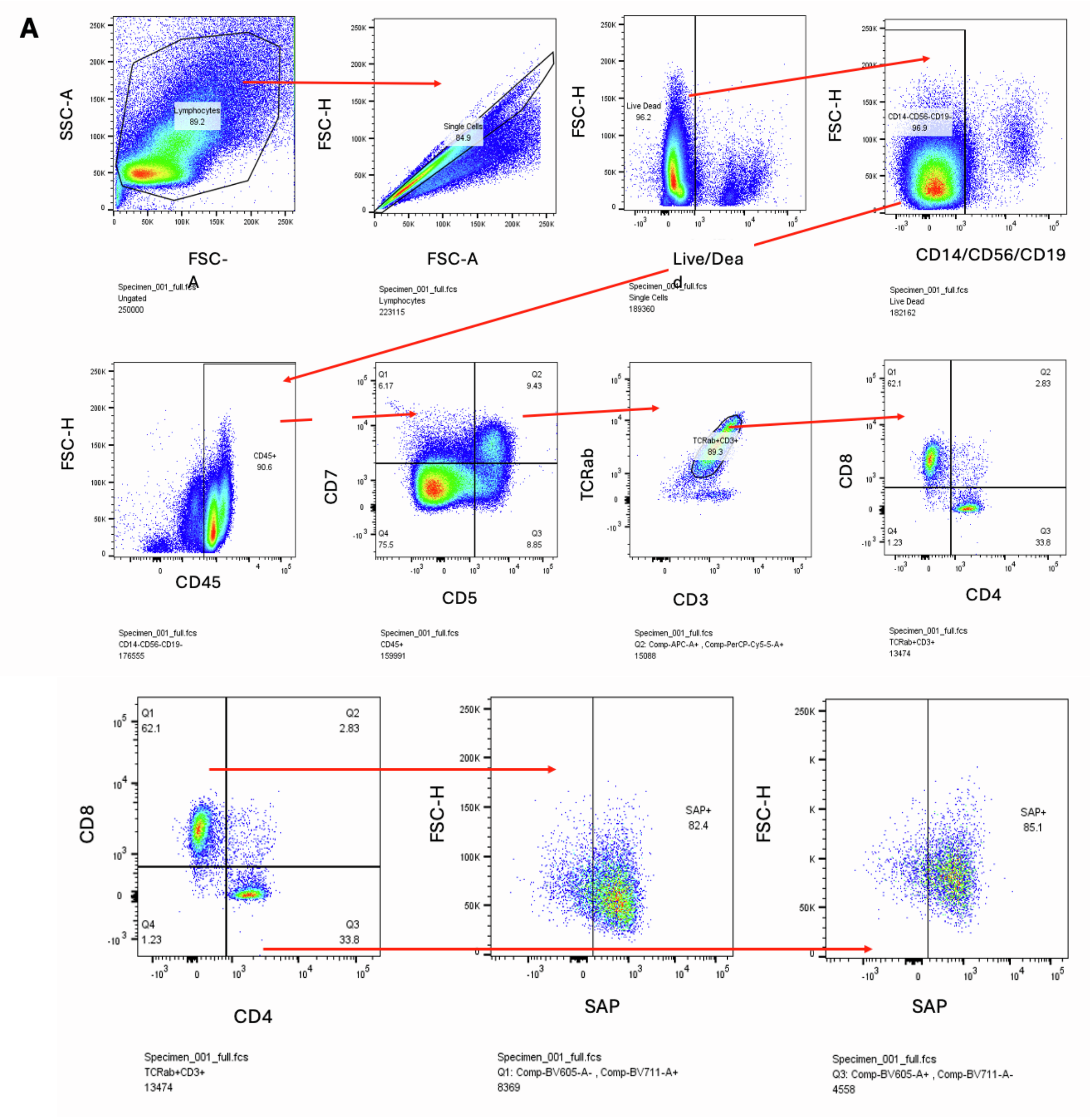
A
Lymphocytes 89.2
SSC-A
FSC-A
Specimen_001_full.fcs
Ungated
250000
Single Cells 84.9
FSC-H
FSC-A
Specimen_001_full.fcs
Lymphocytes
223115
Live Dead 96.2
FSC-H
Live/Dead
Specimen_001_full.fcs
Single Cells
189360
CD14-CD56-CD19- 96.9
FSC-H
CD14/CD56/CD19
Specimen_001_full.fcs
Live Dead
182162
CD45+ 90.6
FSC-H
CD45
Specimen_001_full.fcs
CD14-CD56-CD19-
176555
Q1 6.17
Q2 9.43
Q4 75.5
Q3 8.85
CD7
CD5
Specimen_001_full.fcs
CD45+
159991
TCRab+CD3+ 89.3
TCRab
CD3
Specimen_001_full.fcs
Q2: Comp-APC-A+ , Comp-PerCP-Cy5-5-A+
15088
Q1 62.1
Q2 2.83
Q4 1.23
Q3 33.8
CD8
CD4
Specimen_001_full.fcs
TCRab+CD3+
13474
Q1 62.1
Q2 2.83
Q4 1.23
Q3 33.8
CD8
CD4
Specimen_001_full.fcs
TCRab+CD3+
13474
SAP+ 82.4
FSC-H
SAP
Specimen_001_full.fcs
Q1: Comp-BV605-A- , Comp-BV711-A+
8369
SAP+ 85.1
FSC-H
SAP
Specimen_001_full.fcs
Q3: Comp-BV605-A+ , Comp-BV711-A-
4558

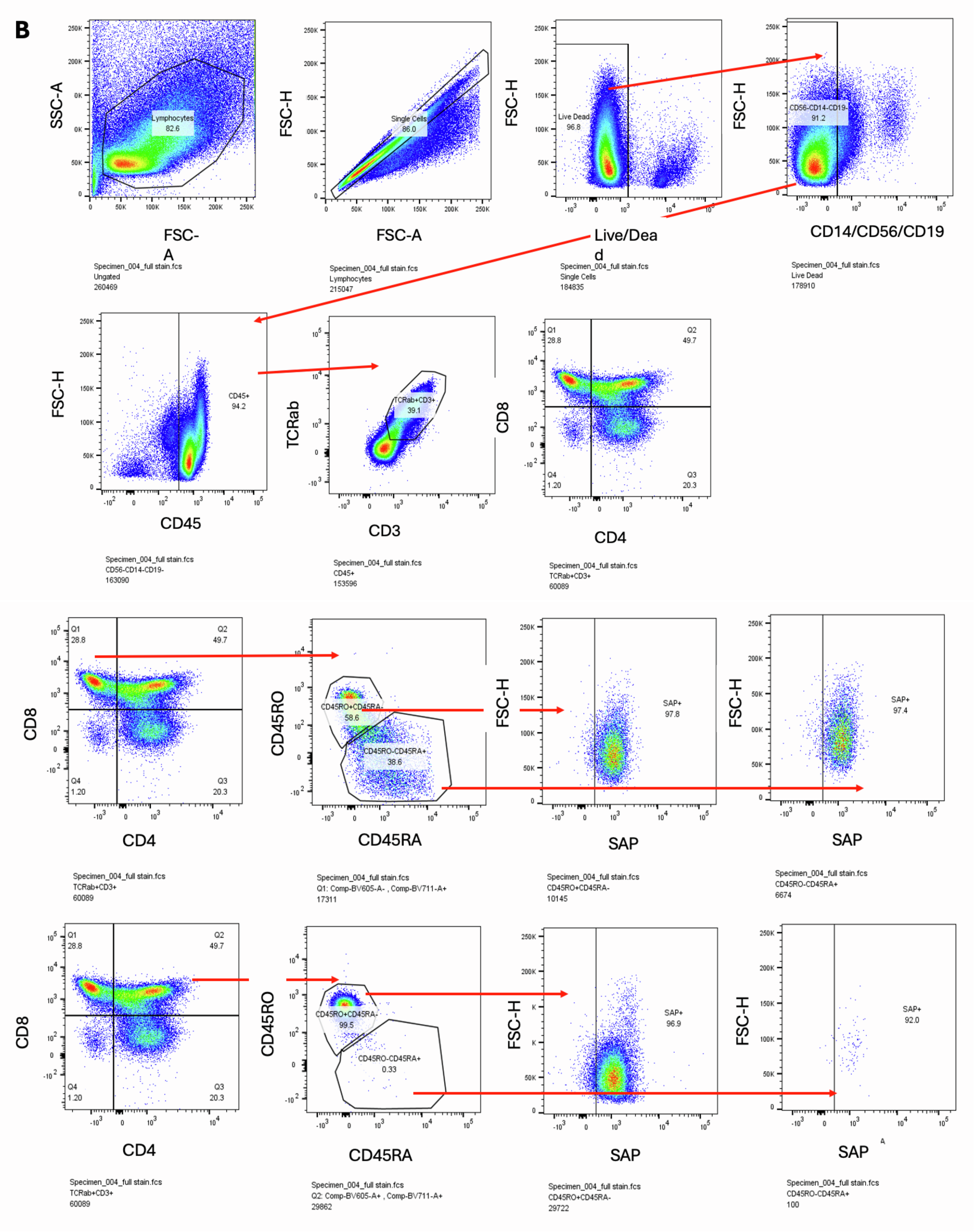
B
Lymphocytes 82.6
Single Cells 86.0
Live Dead 96.8
CD56-CD14-CD19- 91.2
SSC-A
FSC-A
FSC-H
Live/Dead
CD14/CD56/CD19
Specimen_004_full stain.fcs Ungated 260469
Specimen_004_full stain.fcs Lymphocytes 215047
Specimen_004_full stain.fcs Single Cells 184835
Specimen_004_full stain.fcs Live Dead 178910
CD45+ 94.2
TCRab+CD3+ 39.1
Q1 28.8
Q2 49.7
Q3 20.3
Q4 1.20
CD45
TCRab
CD3
CD8
CD4
Specimen_004_full stain.fcs CD56-CD14-CD19- 163090
Specimen_004_full stain.fcs CD45+ 153596
Specimen_004_full stain.fcs TCRab+CD3+ 60089
CD45RO+CD45RA- 58.6
CD45RO-CD45RA+ 38.6
SAP+ 97.8
SAP+ 97.4
CD45RO
CD45RA
SAP
Specimen_004_full stain.fcs TCRab+CD3+ 60089
Specimen_004_full stain.fcs Q1: Comp-BV605-A- , Comp-BV711-A+ 17311
Specimen_004_full stain.fcs CD45RO+CD45RA- 10145
Specimen_004_full stain.fcs CD45RO-CD45RA+ 6674
CD45RO+CD45RA- 99.5
CD45RO-CD45RA+ 0.33
SAP+ 96.9
SAP+ 92.0
Specimen_004_full stain.fcs TCRab+CD3+ 60089
Specimen_004_full stain.fcs Q2: Comp-BV605-A+ , Comp-BV711-A+ 29862
Specimen_004_full stain.fcs CD45RO+CD45RA- 29722
Specimen_004_full stain.fcs CD45RO-CD45RA+ 100

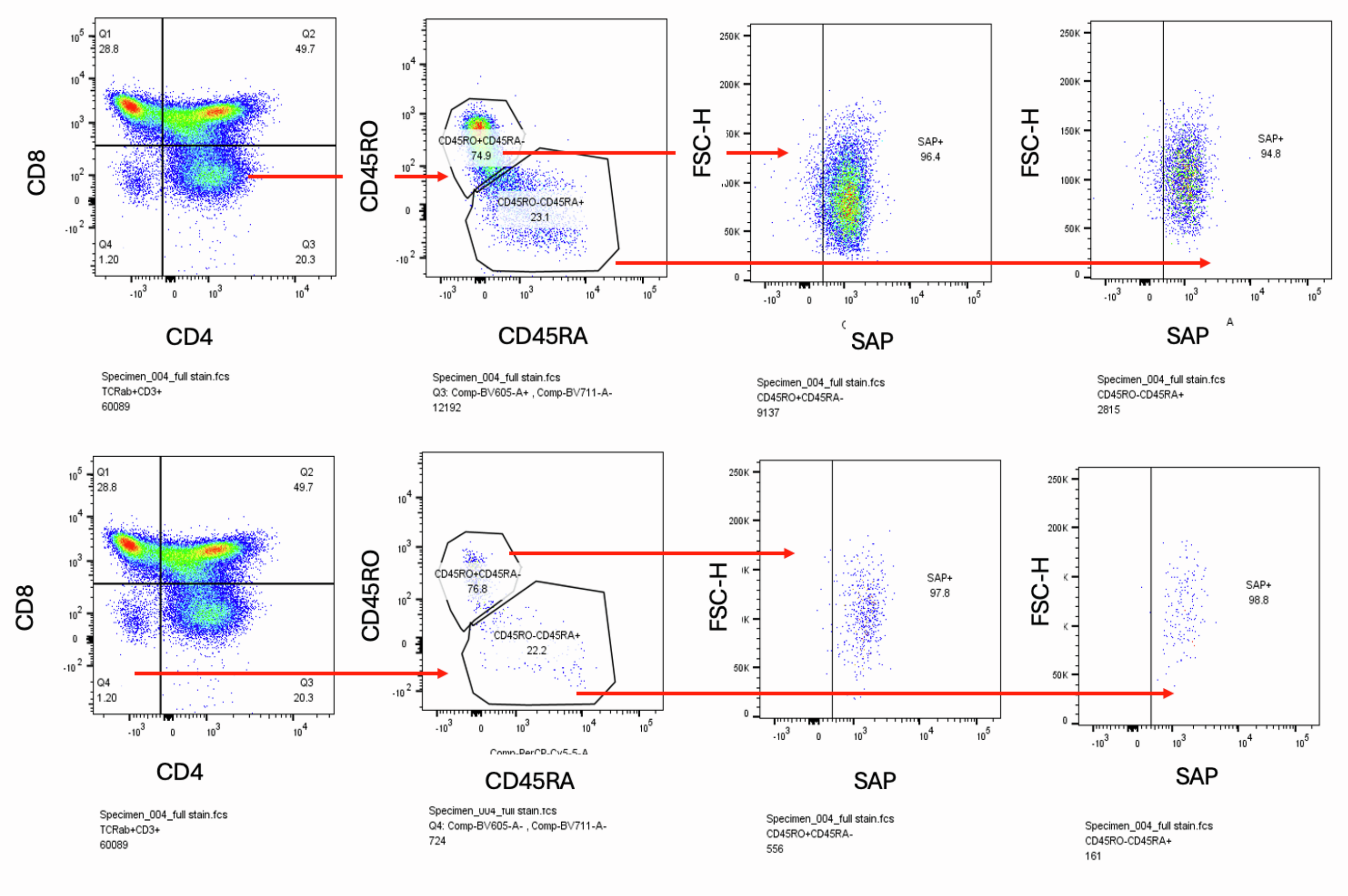

Representative flow gating strategy of T cell populations in a human thymus as described in Figure S3. Panel A illustrates flow gating strategy for human thymus within early development. Panel B illustrates the flow gating strategy for human thymus within mature development.

Figure S13:

A

				NKPs	Immature NKs		Mature NKs					
				NKP	Pre-NK cell		iNK cell	CD56bright NK Cell		CD56dim NK Cell		
**Markers**	**Colors**	**Laser**	**Filter**	**Stage 1**	**Stage 2a**	**Stage 2b**	**Stage 3**	**Stage 4a**	**Stage 4b**	**Stage 5**	**Stage 6**	
**CD34**	PE-Cy7	Yellow	780/60	+	+	+	-	-	-	-	-	
**CD117**	FITC	Blue	530/30	-	+	+	+	+ (low)	-	-	-	
**CD122**	BV650	Violet	610/20	-	-	+	+	+	+	+	+	
**CD56**	APC-Cy7	Red	780/60	-	-	- (low)	- (low)	+ (bright)	+ (bright)	+ (dim)	+ (dim)	
**CD94**	PE-Cy7	Yellow	575/26	-	-	-	-	+	+	(-/+)	(-/+)	
**NKp80**	APC-Cy7	Red	660/20	-	-	-	-	-	+	+	+	
**CD16**	BV421	Bviolet	450/40	-	-	-	-	-	-	+	+	
**CD57**	PerCP-Cy5.5	Blue	695/40	-	-	-	-	-	-	-	+	
**Ghost Dye**	UV	UV	450/50									

B

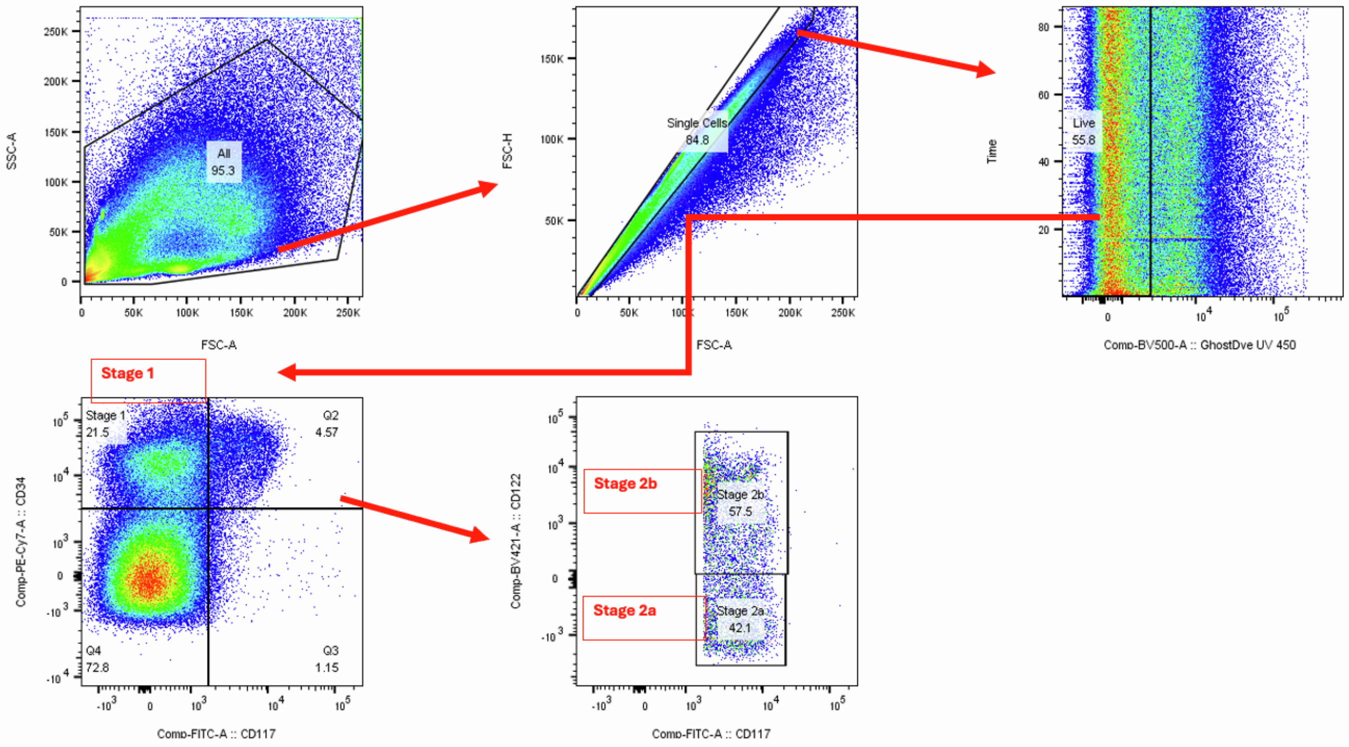

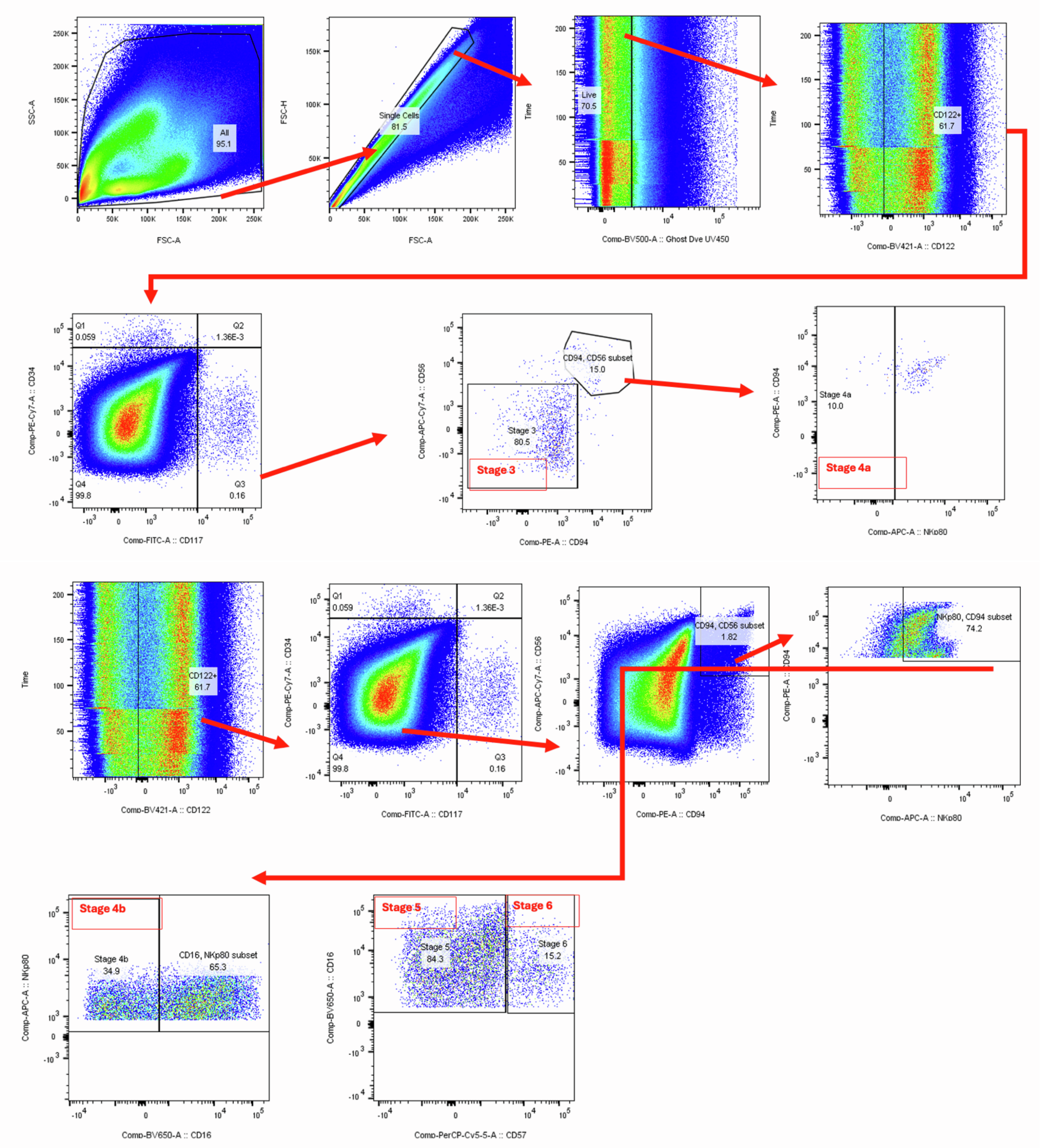

Representative flow gating strategy of NK cell populations in human peripheral blood as described in Figure S3. Panel A illustrates the flow panel used to differentiate NK developmental markers across development. Panel B illustrates the flow gating strategy for human NK cells across development in peripheral blood.

Figure S14:

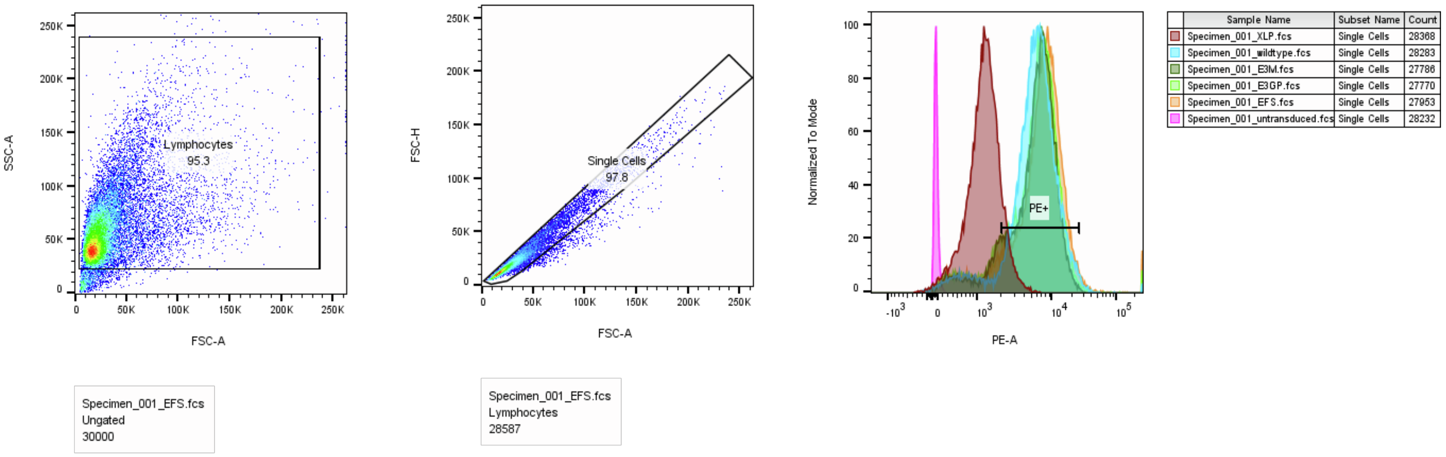

Flow Cytometry gating strategy of SAP Expression of XLP1 Patient T cells transduced with XLP1-SMART LVs as described in Figure 5 panel A.

Figure S15:

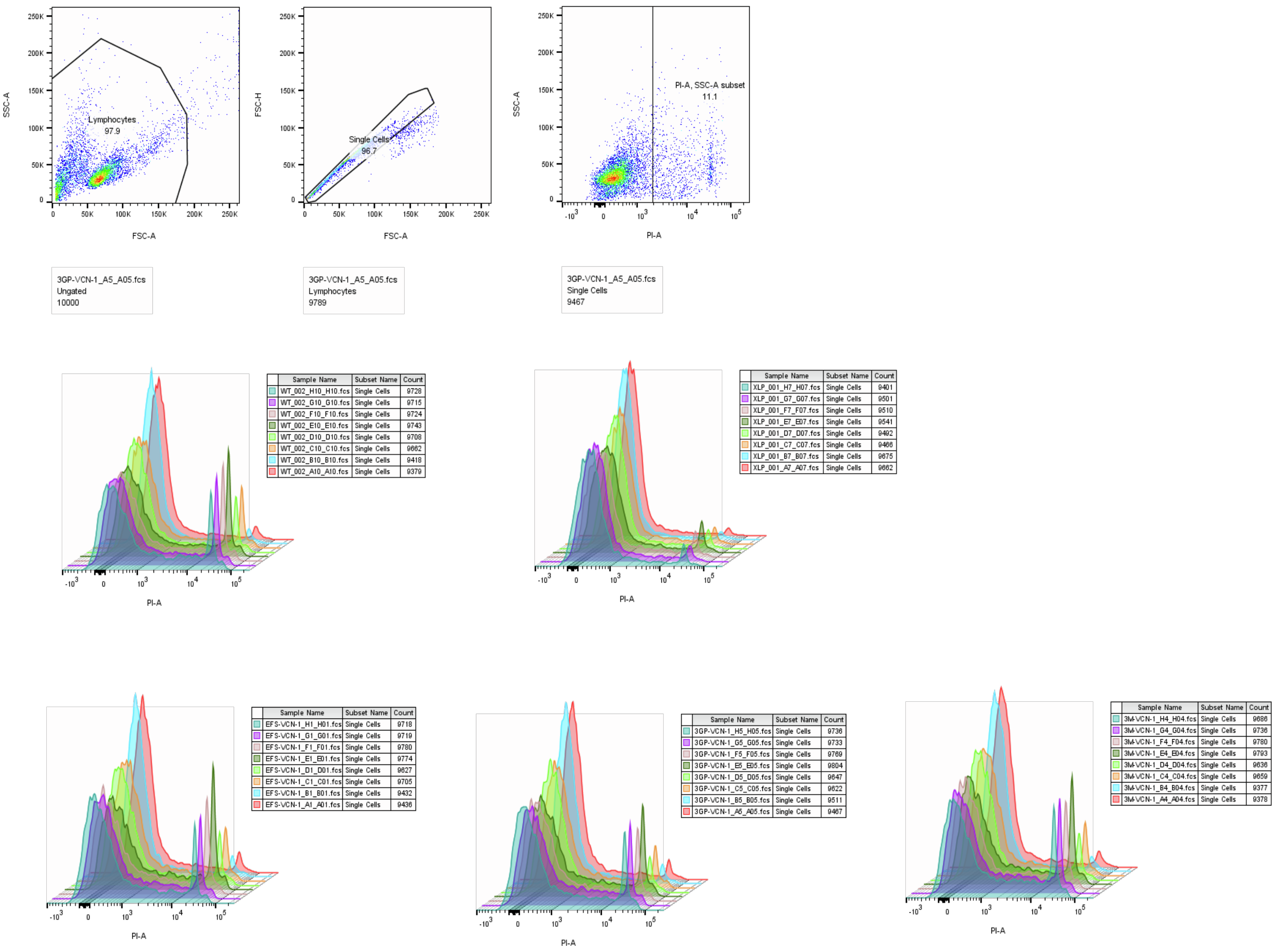

Flow cytometry gating strategy of T Cell RICD as described in Figure 5 panel B.

Figure S16:

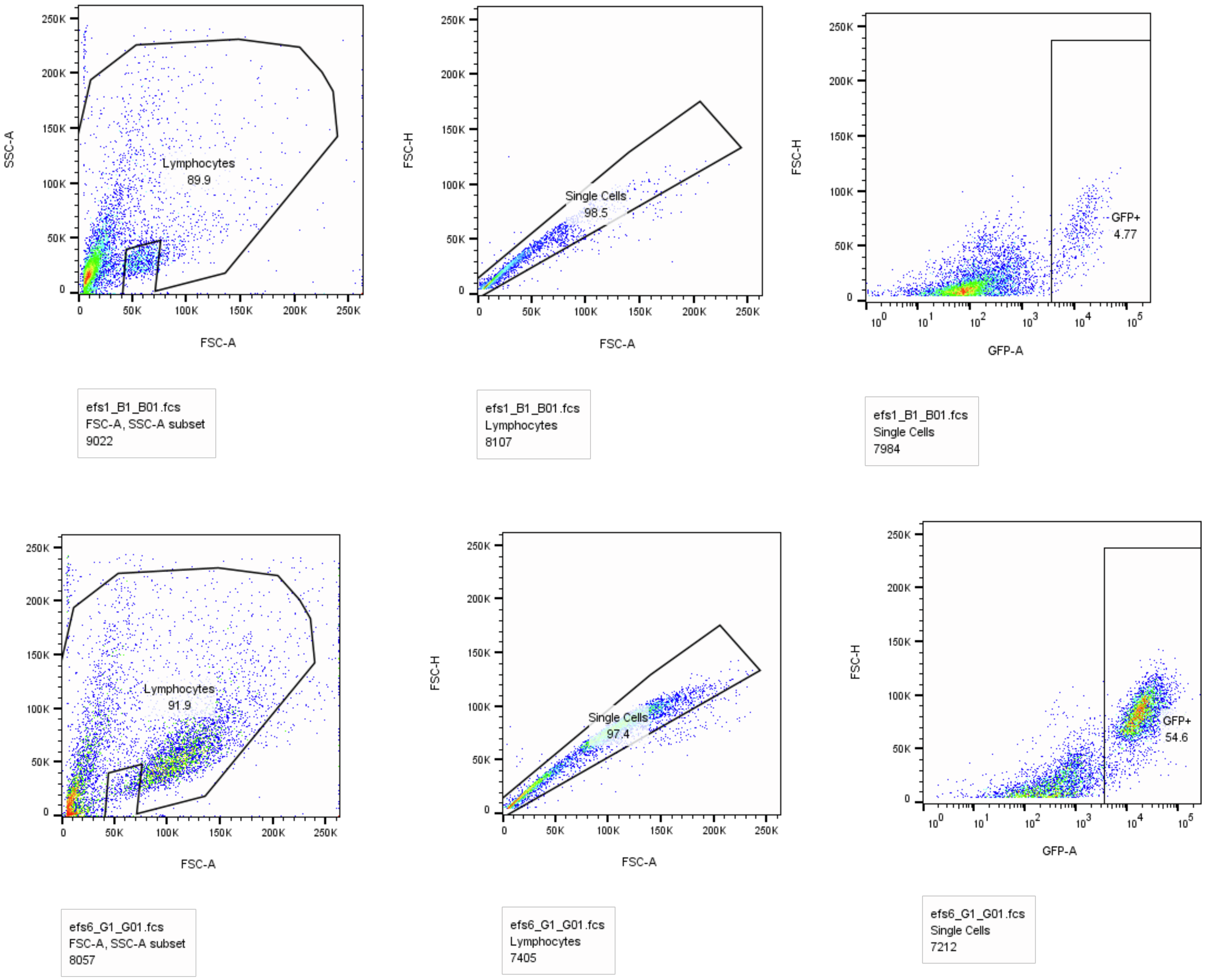
SSC-A
FSC-A
Lymphocytes
89.9
FSC-H
Single Cells
98.5
GFP-A
GFP+
4.77
efs1_B1_B01.fcs
FSC-A, SSC-A subset
9022
efs1_B1_B01.fcs
Lymphocytes
8107
efs1_B1_B01.fcs
Single Cells
7984
Lymphocytes
91.9
Single Cells
97.4
GFP+
54.6
efs6_G1_G01.fcs
FSC-A, SSC-A subset
8057
efs6_G1_G01.fcs
Lymphocytes
7405
efs6_G1_G01.fcs
Single Cells
7212

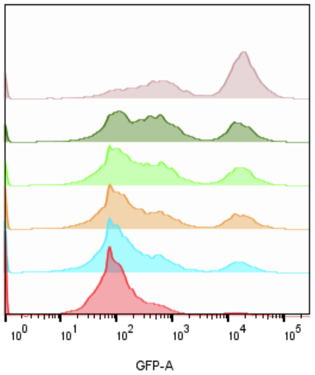

	Sample Name	Subset Name	Count
	efs6_G1_G01.fcs	Single Cells	7212
	efs5_F1_F01.fcs	Single Cells	7535
	efs4_E1_E01.fcs	Single Cells	7967
	efs3_D1_D01.fcs	Single Cells	7435
	efs2_C1_C01.fcs	Single Cells	7783
	efs1_B1_B01.fcs	Single Cells	7984

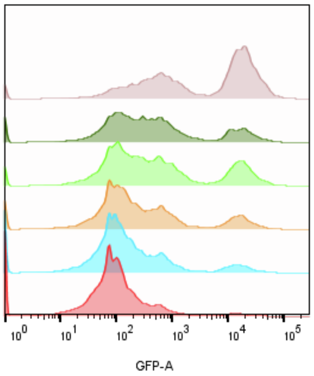

	Sample Name	Subset Name	Count
	e3gp6_G7_G07.fcs	Single Cells	7696
	e3gp5_F7_F07.fcs	Single Cells	6247
	e3gp4_E7_E07.fcs	Single Cells	8297
	e3gp3_D7_D07.fcs	Single Cells	7081
	e3gp2_C7_C07.fcs	Single Cells	7376
	e3gp1_B7_B07.fcs	Single Cells	6969

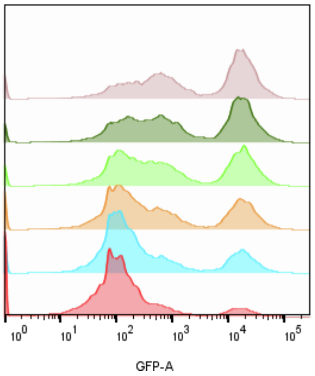

	Sample Name	Subset Name	Count
	wt6_G1_G01.fcs	Single Cells	7879
	wt5_F1_F01.fcs	Single Cells	8632
	wt4_E1_E01.fcs	Single Cells	8616
	wt3_D1_D01.fcs	Single Cells	8652
	wt2_C1_C01.fcs	Single Cells	8914
	wt1_B3_B03.fcs	Single Cells	8466

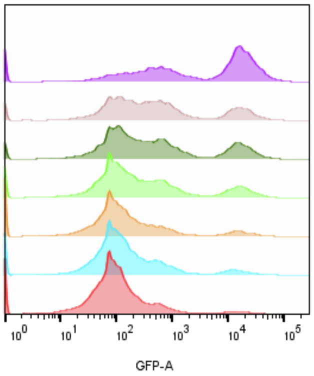

	Sample Name	Subset Name	Count
	xlp6_G11_G11.fcs	Single Cells	7158
	xlp5_F10_F10.fcs	Single Cells	7211
	xlp4_E10_E10.fcs	Single Cells	8217
	xlp3_D11_D11.fcs	Single Cells	8346
	xlp2_C12_C12.fcs	Single Cells	6890
	xlp2_C10_C10.fcs	Single Cells	8401
	xlp1_B10_B10.fcs	Single Cells	8340

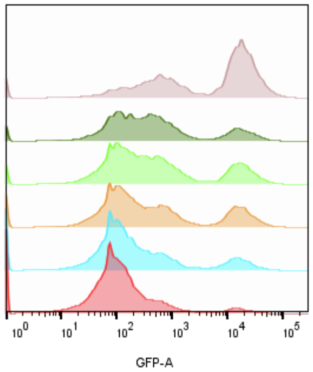

	Sample Name	Subset Name	Count
	e3m6_G4_G04.fcs	Single Cells	7788
	e3m5_F4_F04.fcs	Single Cells	6136
	e3m4_E4_E04.fcs	Single Cells	8315
	e3m3_D4_D04.fcs	Single Cells	7820
	e3m2_C4_C04.fcs	Single Cells	7678
	e3m1_B4_B04.fcs	Single Cells	7858

Flow Cytometry gating strategy of for the NK cell cytotoxicity assay using K562s as described in Figure 5 panel C.

Figure S17:

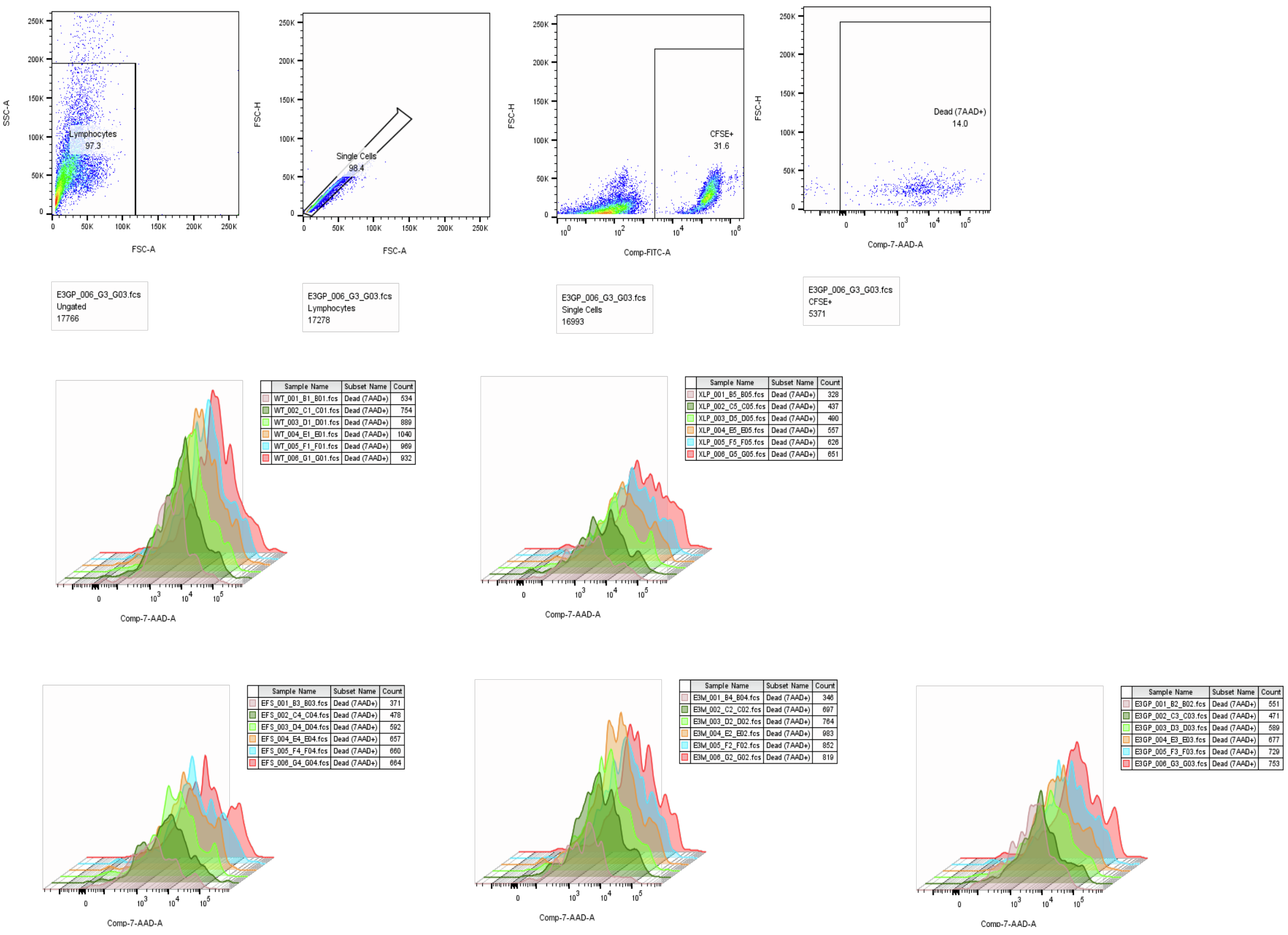

Flow Cytometry gating strategy of for the NK cell cytotoxicity assay using K562s as described in Figure 5 panel D.

Table S1: Enhancer Sequences

See Supplemental Table

Table S2: The wildtype (WT) or codon optimized (GeneArt or JCAT) DNA sequence of SH2D1A.

Codon optimization	Sequence
WT	ATGGACGCAGTGGCTGTGTATCATGGCAAAATCAGCAGGGAAACCGGCGAGAAGCTCCTGCTTG CCACTGGGCTGGATGGCAGCTATTTGCTGAGGGACAGCGAGAGCGTGCCAGGCGTGTACTGCC TATGTGTGCTGTATCACGGTTACATTTATACATACCGAGTGTCCCAGACAGAAACAGGTTCTTGGA GTGCTGAGACAGCACCTGGGGTACATAAAAGATATTTCCGGAAAATAAAAAATCTCATTTCAGCAT TTCAGAAGCCAGATCAAGGCATTGTAATACCTCTGCAGTATCCAGTTGAGAAGAAGTCCTCAGCT AGAAGTACACAAGGTACTACAGGGATAAGAGAAGATCCTGATGTCTGCCTGAAAGCCCCATGA
GeneArt	ATGGATGCCGTGGCCGTGTATCACGGCAAGATCAGCAGAGAGACAGGCGAGAAACTGCTGCTG GCCACAGGCCTGGATGGCAGCTATCTGCTGAGAGACTCTGAGAGCGTGCCCGGCGTGTACTGT CTGTGTGTGCTGTACCACGGCTACATCTACACCTACCGGGTGTCCCAGACCGAGACAGGATCTT GGAGCGCCGAAACAGCTCCTGGCGTGCACAAGCGGTACTTCAGAAAGATCAAGAACCTGATCAG CGCCTTCCAGAAGCCTGACCAGGGCATCGTGATCCCTCTGCAGTACCCCGTGGAAAAGAAGTCC AGCGCCAGAAGCACCCAGGGCACCACAGGCATCAGAGAAGATCCCGACGTGTGCCTGAAGGCC CCTTGA
JCAT	ATGGACGCCGTGGCCGTGTACCACGGCAAGATCAGCCGCGAGACCGGCGAGAAGCTGCTGCTG GCCACCGGCCTGGACGGCAGCTACCTGCTGCGCGACAGCGAGAGCGTGCCCGGCGTGTACTG CCTGTGCGTGCTGTACCACGGCTACATCTACACCTACCGCGTGAGCCAGACCGAGACCGGCAGC TGGAGCGCCGAGACCGCCCCCGGCGTGCACAAGCGCTACTTCCGCAAGATCAAGAACCTGATC AGCGCCTTCCAGAAGCCCGACCAGGGCATCGTGATCCCCCTGCAGTACCCCGTGGAGAAGAAG AGCAGCGCCCGCAGCACCCAGGGCACCACCGGCATCCGCGAGGACCCCGACGTGTGCCTGAA GGCCCCCTGA

Table S3 SH2D1A -/- Jurkat Western Blot Densitometry:

Lane	Condition	Control	Target	T/C Ratio	% of WT
1	WT Jurkat	15322.255	10810.104	0.706	100%
2	SH2D1A-/- Jurkat	13984.669	423.698	0.030	4%
3	E3M VCN 1	14951.69	239.92	0.016	2%
4	E3M VCN 3	11348.619	1804.134	0.159	23%
5	E3M VCN 5	10183.104	2436.154	0.239	34%
6	E3GP VCN 1	7034.447	244.213	0.035	5%
7	E3GP VCN 3	16223.619	1200.234	0.074	10%
8	E3GP VCN 5	13031.912	2857.054	0.219	31%

Table S4: ddPCR Primer and Probe sets for detecting vector copy number of lentiviral vectors.

Name	Sequence	Modification
SDC4 Probe	CCCACCGAACCCAAGAAACTAGAGGAGAAT	HEX
PSI Probe	Ccctcagacccttttagtcagtgtggaaaatctctag	FAM
SDC4 Primer F	CAGGGTCTGGGAGCCAAGT	-
SDC4 Primer R	GCACAGTGCTGGACATTGACA	-
PSI Primer F	cactcccaacgaagacaaga	-
PSI Primer R	cctctggtttccctttcgct	-

Table S5: sgRNA sequences for knocking out *SH2D1A* in Jurkat and T cells to create SAP deficient cells.

Name	Sequence
XLP-sgRNA4-SH2D1Ako	GACGCAGTGGCTGTGTATCA
XLP-sgRNA7-SH2D1Ako	AACAGGTTCTTGGAGTGCTG

Table S6: Antibodies

Marker	Color	Website
SAP	PE	https://www.thermofisher.com/antibody/product/SAP-SLAM-Associated-Protein-Antibody-clone-XLP-1D12-Monoclonal/12-9787-42
CD33	BV711	https://www.bdbiosciences.com/eu/applications/research/stem-cell-research/hematopoietic-stem-cell-markers/human/negative-markers/bv711-mouse-anti-human-cd33-wm53-also-known-as-wm-53/p/563171
CD56	FITC	https://www.biolegend.com/it-it/products/fitc-anti-human-cd56-ncam-antibody-3795
CD16	APC-CY7	https://www.bdbiosciences.com/us/applications/research/stem-cell-research/cancer-research/human/apc-cy7-mouse-anti-human-cd16-3g8/p/557758
CD19	PE-Cy7	https://www.bdbiosciences.com/us/applications/research/clinical-research/oncology-research/blood-cell-disorders/surface-markers/human/pe-cy7-mouse-anti-human-cd19-sj25c1/p/557835
CD3	PerCP Cy5.5	https://www.bdbiosciences.com/us/applications/research/t-cell-immunology/th-1-cells/surface-markers/human/percp-cy55-mouse-anti-human-cd3-ucht1-also-known-as-ucht-1-ucht-1/p/560835
TCR V*a*	APC	https://www.biolegend.com/en-us/products/apc-anti-human-tcr-valpha24-jalpha18-inkt-cell-antibody-6030
CD14	BV421	https://www.bdbiosciences.com/us/reagents/research/antibodies-buffers/immunology-reagents/anti-human-antibodies/cell-surface-antigens/bv421-mouse-anti-human-cd14-m5e2/p/565283
CD4	BV605	https://www.bdbiosciences.com/us/applications/research/t-cell-immunology/th-1-cells/surface-markers/human/bv605-mouse-anti-human-cd4-rpa-t4/p/562658
CD8	BV510	https://www.bdbiosciences.com/us/reagents/research/antibodies-buffers/immunology-reagents/anti-non-human-primate-antibodies/cell-surface-antigens/bv510-mouse-anti-human-cd8-sk1/p/563919
CD34	Pe-Cy7	PE/Cyanine7 anti-human CD34 Antibody anti-CD34 - 561 (biolegend.com)
CD117	FITC	FITC anti-human CD117 c-kit Antibody anti-CD117 - 104D2 (biolegend.com)
CD122	BV421	BV421,Mouse,Anti-Human,CD122,Mik-β3,RUO - 562887 | BD Biosciences-US
CD56	APC-Cy7	APC/Cyanine7 anti-human CD56 NCAM Antibody anti-CD56 (NCAM) - 5.1H11 (biolegend.com)
CD94	PE	PE anti-human CD94 Antibody anti-CD94 - DX22 (biolegend.com)
NKp80	AF750	https://www.rndsystems.com/products/human-nkp80-klrf1-alexa-fluor-750-conjugated-antibody-239127_fab1900s
CD16	BV650	BV650,Mouse,Anti-Human,CD16,3G8,RUO - 563691 | BD Biosciences-US
CD16	BV510	https://www.bdbiosciences.com/en-us/products/reagents/flow-cytometry-reagents/research-reagents/single-color-antibodies-ruo/bv510-mouse-anti-human-cd16.563830
CD19	APC	https://www.thermofisher.com/antibody/product/CD19-Antibody-clone-SJ25C1-Monoclonal/17-0198-42
CD3	PE-Texas Red	https://www.thermofisher.com/antibody/product/CD3-Antibody-clone-7D6-Monoclonal/MHCD0317
CD5	BV421	https://biolegend.com/en-us/products/brilliant-violet-421-anti-human-cd5-antibody-15832
TCRab	PECy7	https://www.biolegend.com/en-us/products/pe-cyanine7-anti-human-tcr-alpha-beta-antibody-6731?GroupID=GROUP28
CD34	APC Cy7	https://www.biolegend.com/en-us/search-results/apc-cyanine7-anti-human-cd34-antibody-6159?GroupID=BLG7551&gclid=Cj0KCQjw8NilBhDOARIsAHzpbLCaj4FNP3yWHPoqv4lwQTjoWuJsEJNeiplJyBfSU63E5--5OcJVLSYaAgAQEALw_wcB

CD57	PerCP Cy5.5	https://www.biolegend.com/en-us/search-results/percp-cyanine5-5-anti-human-cd57-antibody-12127?GroupID=BLG8483&gclid=Cj0KCQjw8NilBhDOARIsAHzpbLDMTI7FbaVoxg847nDqC4lwuCx7_unezyYJZtQdAIVBNLivY84ZtrIaAi6rEALw_wcB
CD94	PE Dazzle	https://www.biolegend.com/en-us/products/pe-dazzle-594-anti-human-cd94-antibody-16042
CD19	PE/Cy7	https://www.biolegend.com/en-us/search-results/pe-cyanine7-anti-human-cd19-antibody-1911?gclid=Cj0KCQjw8NilBhDOARIsAHzpbLAEdWtO6QNY24GLob-tVN2VjUxNia9K5Zu8KJ5LXL-Vleljfa6PrfwaAv4SEALw_wcB
CD7	PE/Cy7	https://www.biolegend.com/en-us/products/pe-cyanine7-anti-human-cd7-antibody-21684
CD14	APC	https://www.biolegend.com/en-us/products/apc-anti-human-cd14-antibody-3953?GroupID=BLG4805
CD56	PE	https://www.bdbiosciences.com/en-us/products/reagents/flow-cytometry-reagents/research-reagents/single-color-antibodies-ruo/pe-mouse-anti-human-cd56.556647
CD45	BV650	https://www.bdbiosciences.com/en-us/products/reagents/flow-cytometry-reagents/research-reagents/single-color-antibodies-ruo/bv650-mouse-anti-human-cd45.563717
CD45	APC	https://www.biolegend.com/en-us/products/apc-anti-human-cd45-antibody-705?GroupID=BLG5926
CD45RA	PerCP Cy5.5	https://www.biolegend.com/en-us/search-results/percp-cyanine5-5-anti-human-cd45ra-antibody-4241
CD45RO	PE	https://www.bdbiosciences.com/en-us/products/reagents/flow-cytometry-reagents/research-reagents/single-color-antibodies-ruo/pe-mouse-anti-human-cd45ro.555493
CD8a	BV711	https://www.bdbiosciences.com/en-us/products/reagents/flow-cytometry-reagents/research-reagents/single-color-antibodies-ruo/bv711-mouse-anti-human-cd8.563677
CD8b	APC	https://www.biolegend.com/fr-ch/products/apc-anti-human-cd8b-antibody-23661?GroupID=GROUP28
Zombie	UV	https://www.biolegend.com/en-gb/products/zombie-uv-fixable-viability-kit-9336
TCRab	PE-Dazzle	https://www.biolegend.com/en-us/products/pe-dazzle-594-anti-human-tcr-alpha-beta-antibody-12515
CD56	PE-Cy7	https://www.biolegend.com/en-us/products/pe-cyanine7-anti-human-cd56-ncam-antibody-9959
CD8b	APC	https://www.miltenyibiotec.com/US-en/products/cd8b-antibody-anti-human-reafinity-rea715.html#conjugate=apc:size=100-tests-in-200-ul
CD3	APC-Cy7	https://www.biolegend.com/en-us/products/apc-cyanine7-anti-human-cd3-antibody-3929
CD45	BV510	https://www.biolegend.com/en-us/products/brilliant-violet-510-anti-human-cd45-antibody-8006
CD4	BV605	https://www.biolegend.com/en-us/products/brilliant-violet-605-anti-human-cd4-antibody-10434
CD8a	BV711	https://www.biolegend.com/en-us/products/brilliant-violet-711-anti-human-cd8-antibody-10762
CD45RO	BV785	https://www.biolegend.com/en-us/products/brilliant-violet-785-anti-human-cd45ro-antibody-7973
CD7	PerCP Cy5.5	https://www.biolegend.com/en-us/products/percp-cyanine5-5-anti-human-cd7-antibody-13918
CD5	APC	https://www.biolegend.com/en-us/products/apc-anti-human-cd5-antibody-868
CD34	APC-Cy7	https://www.biolegend.com/en-us/products/apc-cyanine7-anti-human-cd34-antibody-12973
CD3	BV785	https://www.biolegend.com/en-us/products/brilliant-violet-785-anti-human-cd3-antibody-14454
Fcblock		https://www.biolegend.com/en-us/products/human-trustain-fcx-fc-receptor-blocking-solution-6462
DAPI		https://www.thermofisher.com/order/catalog/product/D1306

Table S7: Barcodes for next generation sequencing

Construct	Barcode
XLP1-E1-1-mCit-WPRE	AACAGACCGACAGGTTCTAC
XLP1-E1-2-mCit-WPRE	AACGTCCTTAAGGCCACTTC
XLP1-E2-1-mCit-WPRE	AACTACGAGGCGCAAGTGCT
XLP1-E2-2-mCit-WPRE	AAGTCGCATAATCCTTGGTC
XLP1-E3-1-mCit-WPRE	GATCTTGCGGCACCGAGATT
XLP1-E3-2-mCit-WPRE	GGTGCGTGTAAGAAGTTGTC
XLP1-E4-1-mCit-WPRE	ACCACCATAGTCACCATTGT
XLP1-E4-2-mCit-WPRE	ACCGAGTTAAGCCTATGCTG
XLP1-E5-1-mCit-WPRE	ACGAGTTACATATAGAGACC
XLP1-E5-2-mCit-WPRE	ACTAATCAGGTCATAAGCCT
XLP1-E6-1-mCit-WPRE	ACTGTGAACAACCATGTGGC
XLP1-E6-2-mCit-WPRE	AGCATACGAGTAGAGTTGGT
XLP1-E7-1-mCit-WPRE	AGGAGCATTACTGTAGATTC
XLP1-E7-2-mCit-WPRE	AGTGGTAGGATCCTGCACAC
XLP1-E8-1-mCit-WPRE	ATACACCAGAACCTAACAAC
XLP1-E8-2-mCit-WPRE	ATGACTCGGACCATTGCCAC
XLP1-E9-1-mCit-WPRE	ATGCCGTTCAACTGGCTATC
XLP1-E9-2-mCit-WPRE	ATGGAACCTACTCACACCTC
XLP1-E10-1-mCit-WPRE	ATGGATTGAGCGACGAACGT
XLP1-E10-2-mCit-WPRE	ATGTTCGTAGCGAATAGACT
XLP1-E11-1-mCit-WPRE	ATTCGATCGGCGGAGGCATT
XLP1-E11-2-mCit-WPRE	CAACACGGAACTTCGTCACG
XLP1-E12-1-mCit-WPRE	CAATGTGCGAGATTACGTAC
XLP1-E12-2-mCit-WPRE	CACGGTTGAAGTACTACGCG
XLP1-E13-1-mCit-WPRE	CACTAAGTCAGAGGACAATC
XLP1-E13-2-mCit-WPRE	CCACAGCTCAGTTGCTCATC
XLP1-E14-1-mCit-WPRE	GAGTCTCCAGTTATGCGTGT
XLP1-E14-2-mCit-WPRE	GAGTTGGTGAGGATACAGGC
XLP1-E16-1-mCit-WPRE	GTCTCAAGGTTGGCAGGCT
XLP1-E16-2-mCit-WPRE	GTTATGAGGATTCCAGTGAC
XLP1-E17-1-mCit-WPRE	GTTCGCCGTACATATGCGTC
XLP1-E17-2-mCit-WPRE	TACTTGGCAGGTCCAAGTGT
XLP1-E18-1-mCit-WPRE	CTTCAGACAGGCGATCTCGT
XLP1-E18-2-mCit-WPRE	GACGCAGCTACGATCTACTC
XLP1-E19-1-mCit-WPRE	CGTTAAGCGGCAAGTCTTCT
XLP1-E19-2-mCit-WPRE	CTAGGTATCATCCGCGAATC
XLP1-E20-1-mCit-WPRE	GAACCGGTAGTGCAGCAACT
XLP1-E20-2-mCit-WPRE	GAACGCTCCAGTCAAGCTCC

XLP1-E21-1-mCit-WPRE	GACATCGACAAGTCAGGTGC
XLP1-E21-2-mCit-WPRE	GACCATAGAGGTAGAGCGGT
XLP1-E22-1-mCit-WPRE	GGATTCGCAATGAGACCTCG
XLP1-E22-2-mCit-WPRE	GGATTGCAGGCCTAATACCT
XLP1-E23-1-mCit-WPRE	TCCTCCGTTAGCTATTGTTC
XLP1-E23-2-mCit-WPRE	TCGGATGTCAACGAAGGATC
XLP1-E25-1-mCit-WPRE	TCTCTGTAAGGTTGCCTTAT
XLP1-E25-2-mCit-WPRE	TGACAATGCAGAGTAGTGCC
XLP1-E26-1-mCit-WPRE	CTCCTAAGAATCCGTACTGG
XLP1-E26-2-mCit-WPRE	CTGTTCCACATTGCTGAACC
XLP1-E30-1-mCit-WPRE	CGCAGATAGGTAGCTCGCCT
XLP1-E30-2-mCit-WPRE	CGCGTTAATATAGCCTTATC
XLP1-E31-1-mCit-WPRE	CCGGTATGCAGGTCGTATCC
XLP1-E31-2-mCit-WPRE	CCTTGGTGGAAGTTGTCCAC
XLP1-Int1-1-mCit-WPRE	CGACAACAAGGCTGTGGCAT
XLP1-Int1-2-mCit-WPRE	CGATCCTAGAGTCTTCCGAC
XLP-5'Region-1-mCit-WPRE	GCATACTAAGCCTCTCGAGT
XLP-5'Region-2-mCit-WPRE	GGAAGAAGAAGCTGACATGG
XLP-5'RegionCore-1-mCit-WPRE	TAGATCAGAGATGCGCAGGT
XLP-5'RegionCore-2-mCit-WPRE	TCAGCTCAAGCATCCTTCAT
XLP-5'RegionUltraCore-1-mCit-WPRE	CGGTGTGAAGCGGTACACAT
XLP-5'RegionUltraCore-2-mCit-WPRE	CGTATCTCCACCGAACTTCC
XLP-3Region-1-mCit-WPRE	GGCGAACACATTGGAATTGC
XLP-3Region-2-mCit-WPRE	GGCGTTGTAGGCTCGATACT
XLP1-Pro-1-mCit-WPRE	TTATCGAGAGTACGTGAGGT
XLP1-Pro-2-mCit-WPRE	TTGCCACAGGATAGCGACCT
XLP1-EFS-1-mCit-WPRE	TGCCGAATAGTATGCTGTGT
XLP1-EFS-2-mCit-WPRE	TGTACCACGGAGTGGACTCT
XLP1-Cntrl-1-mCit-WPRE	ACAACGGTGAATAGTAAGGC
XLP1-Cntrl-2-mCit-WPRE	ACAAGAGAAGTGTTCGCAAT

Table S8: Primers used for cloning

See Supplemental Table File
